# A telomere-to-telomere gapless genome reveals SlPRR1 control of circadian rhythm and photoperiodic flowering in tomato

**DOI:** 10.1093/gigascience/giaf058

**Published:** 2025-07-02

**Authors:** Hui Liu, Jia-Qi Zhang, Jian-Ping Tao, Chen Chen, Li-Yao Su, Jin-Song Xiong, Ai-Sheng Xiong

**Affiliations:** State Key Laboratory of Crop Genetics & Germplasm Enhancement and Utilization, Ministry of Agriculture and Rural Affairs Key Laboratory of Biology and Germplasm Enhancement of Horticultural Crops in East China, College of Horticulture, Nanjing Agricultural University, Nanjing, Jiangsu 210095, China; State Key Laboratory of Crop Genetics & Germplasm Enhancement and Utilization, Ministry of Agriculture and Rural Affairs Key Laboratory of Biology and Germplasm Enhancement of Horticultural Crops in East China, College of Horticulture, Nanjing Agricultural University, Nanjing, Jiangsu 210095, China; State Key Laboratory of Crop Genetics & Germplasm Enhancement and Utilization, Ministry of Agriculture and Rural Affairs Key Laboratory of Biology and Germplasm Enhancement of Horticultural Crops in East China, College of Horticulture, Nanjing Agricultural University, Nanjing, Jiangsu 210095, China; State Key Laboratory of Crop Genetics & Germplasm Enhancement and Utilization, Ministry of Agriculture and Rural Affairs Key Laboratory of Biology and Germplasm Enhancement of Horticultural Crops in East China, College of Horticulture, Nanjing Agricultural University, Nanjing, Jiangsu 210095, China; State Key Laboratory of Crop Genetics & Germplasm Enhancement and Utilization, Ministry of Agriculture and Rural Affairs Key Laboratory of Biology and Germplasm Enhancement of Horticultural Crops in East China, College of Horticulture, Nanjing Agricultural University, Nanjing, Jiangsu 210095, China; State Key Laboratory of Crop Genetics & Germplasm Enhancement and Utilization, Ministry of Agriculture and Rural Affairs Key Laboratory of Biology and Germplasm Enhancement of Horticultural Crops in East China, College of Horticulture, Nanjing Agricultural University, Nanjing, Jiangsu 210095, China; State Key Laboratory of Crop Genetics & Germplasm Enhancement and Utilization, Ministry of Agriculture and Rural Affairs Key Laboratory of Biology and Germplasm Enhancement of Horticultural Crops in East China, College of Horticulture, Nanjing Agricultural University, Nanjing, Jiangsu 210095, China

**Keywords:** cultivated tomato T2T genome, photoperiod, flowering time, chlorophyll biosynthesis, SlPRR1

## Abstract

Cultivated tomato (*Solanum lycopersicum*) is a major vegetable crop of high economic value that serves as an important model for studying flowering time in day-neutral plants. A complete, continuous, and gapless genome of cultivated tomato is essential for genetic research and breeding programs. Here, we report the construction of a telomere-to-telomere (T2T) gap-free genome of *S. lycopersicum* cv. VF36 using a combination of sequencing technologies. The 815.27-Mb T2T “VF36” genome contained 600.23 Mb of transposable elements. Through comparative genomics and phylogenetic analysis, we identified structural variations between the “VF36” and “Heinz 1706” genomes and found no evidence of a recent species-specific whole-genome duplication in the “VF36” tomato. Furthermore, a core circadian oscillator, SlPRR1, was identified, which peaked at night in a circadian rhythm. CRISPR/Cas9-mediated knockdown of *SlPRR1* in tomatoes demonstrated that *slprr1* mutant lines exhibited significantly earlier flowering under long-day condition than wild type. We present a hypothetical model of how SlPRR1 regulates flowering time and chlorophyll biosynthesis in response to photoperiod. This T2T genomic resource will accelerate the genetic improvement of large-fruited tomatoes, and the SlPRR1-related hypothetical model will enhance our understanding of the photoperiodic response in cultivated tomatoes, revealing a regulatory mechanism for manipulating flowering time.

## Introduction

Flowering is essential for the transition of plants from vegetative to reproductive growth and usually regulated by day length (or photoperiod). Long-day (LD) plants flower when the number of daylight hours exceeds a critical value, whereas short-day (SD) plants exhibit the opposite effect [1, 2]. In the LD plant *Arabidopsis thaliana*, the major photoperiodic flowering regulator *CONSTANS* (*CO*) peaks before dusk on long days in response to light and the circadian clock [3]. CO promotes flowering by activating the expression of *FLOWERING LOCUS T* (*FT*) and *SUPPRESSOR OF OVEREXPRESSION OF CO1* (*SOC1*) [4]. Photoperiod responses rely on crosstalk between light perception and the circadian clock, which together control the expression of the flowering hormone florigen [5]. The florigen gene *FT* is induced by the CO protein in the light [6, 7]. In contrast, tomato (*Solanum lycopersicum*, 2n = 24; NCBI:txid4081) is a day-neutral (ND) plant that flowers regardless of the day length. Tomatoes are an important cash crop cultivated worldwide. They are characterized by a sympodial growth habit with scorpioid cymose inflorescence [8]. Flowering time in tomatoes is regulated by several genes and is an important factor for tomato adaptability and genetic improvement. *EARLY FLOWERING* (*ELF*) has been characterized to develop flowers much earlier than parental controls [8]. Several classical genes involved in the control of flowering time in tomatoes, such as *SINGLE FLOWER TRUSS* (*SFT*), *JOINTLESS* (*J*), and *FALSIFLORA* (*FA*), promote flowering, whereas *SELF PRUNING 5 G* (*SP5G*) and *TERMINATING FLOWER* (*TMF*) delay flowering [9]. *SP5G*, an *FT* paralog, contributes to the loss of day length–sensitive flowering by reducing the LD response in tomato cultivars [5].

Metabolic day-length measurement systems have been reported to rely on the circadian clock–controlled balance in a photoperiodic manner [10]. The circadian clock is a molecular timing device that regulates various physiological and developmental processes via endogenous rhythm [11]. Flowering time is a phenological event regulated by clock output pathways [5]. *EARLY FLOWERING 3* (*ELF3*) is a circadian clock–associated gene that rhythmically inhibits the activity of the light input pathways around dusk by reducing clock sensitivity to light during this phase. It acts as a transcriptional regulator that controls the period of flowering time [12]. Mutations in *ELF3* cause early flowering, possibly because of the increased accumulation of *CONSTANS* (*CO*) transcripts and arrhythmic expression of the morning-specific clock-regulated gene *CHLOROPHYLL A/B BINDING 2* (*CAB2*) and the oscillator component *LATE ELONGATED HYPOCOTYL* (*LHY*) [13]. The *PSEUDO RESPONSE REGULATOR1* (*PRR1*) gene, also known as *TIMING OF CAB EXPRESSION1* (*TOC1*), belongs to the PRR family and was the first member discovered to play a central role in the regulation of circadian rhythms [14, 15]. *PRR1* mutants exhibit a faster-running clock, and misexpression of *PRR1* can lead to hypocotyl growth and early flowering [16]. PRR1 represses *LHY* and *CIRCADIAN CLOCK ASSOCIATED1* (*CCA1*) expression in *Arabidopsis* by directly binding to their promoters. However, the flowering regulatory pathways associated with the circadian clock oscillator are less well studied in cultivated tomatoes than in *Arabidopsis*.

Chlorophyll (Chl) plays a central role in harvesting light and transforming it into chemical energy via through photosynthesis during tomato fruit development [17, 18]. Chl is the primary photosynthetically active pigment in plants. Chl metabolism in tomato leaves contributes to photosynthesis [19], and Chl content modulates many metabolic processes that affect fruit quality of fruit. However, it remains unclear which enzymes degrade Chl in the tomato leaf photosystems under LD and SD conditions. Chl synthesis involves 4 main steps: 5-aminolevulinic acid (ALA), protoporphyrin IX, chlorophyll a (Chl a), and chlorophyll b (Chl b) synthesis. Previous studies have reported that *SlCHLH* and *SlCHLI* are important for Chl accumulation in tomato leaves [20]. Transcription factors such as GOLDEN2-LIKE 1 and 2 (GLK1 and 2) have been reported in many species, and their overexpression increases chlorophyll content and can lead to chloroplast development in tissues [21].

Cultivated tomatoes (*Solanum lycopersicum*) are distributed worldwide and are economically significant in the vegetable industry. Tomatoes are good model for horticultural plants, especially for studying fleshy fruit biology [22]. Although several tomato reference genomes have been published [23, 24], a high-accuracy reference genome has reported 31 gaps in SL5.0 [25]. However, a gapless reference genome is unavailable for the large-fruited tomato. Furthermore, centromeres, which are essential for maintaining chromosomal integrity during cell division and ensuring the fidelity of inheritance, remain largely underexplored in plants [26]. Recently, several horticultural species, including carrot, grape, kiwifruit, lemon, and strawberry, have been sequenced with telomere-to-telomere (T2T) assemblies using PacBio high-fidelity (HiFi), Oxford Nanopore Technology (ONT) ultra-long, and high-throughput chromosome conformation capture (Hi-C) technology [27–31]. However, T2T gap-free genomes have not been reported in large-fruited tomatoes.

To improve the completeness of the cultivated tomato reference genome, we assembled a T2T gap-free genome sequence for large-fruited tomato “VF36” using a combination of PacBio-HiFi, ONT ultra-long, and Hi-C technologies. “VF36” tomato is an important variety [32], and it remains unclear whether the core circadian oscillator SlPRR1 regulates flowering time and chlorophyll biosynthesis during different photoperiods. In the present study, we demonstrated that the knockdown of *SlPRR1* in tomatoes caused early flowering under LD conditions and delayed flowering under SD conditions. In this study, we proposed a hypothetical model for the *SlPRR1* regulation of flowering time and chlorophyll biosynthesis in response photoperiod.

## Results

### A T2T gap-free tomato reference genome for “VF36”

Based on *k*-mer analysis with 48.86-Gb Illumina reads, the genome size of the “VF36” tomato was estimated to be 774.22 Mb, with a heterozygosity rate of 0.71% and a duplication ratio of 38.84% (Supplementary Figs. S1–S2 and Supplementary Table S1).

**Figure 1:**
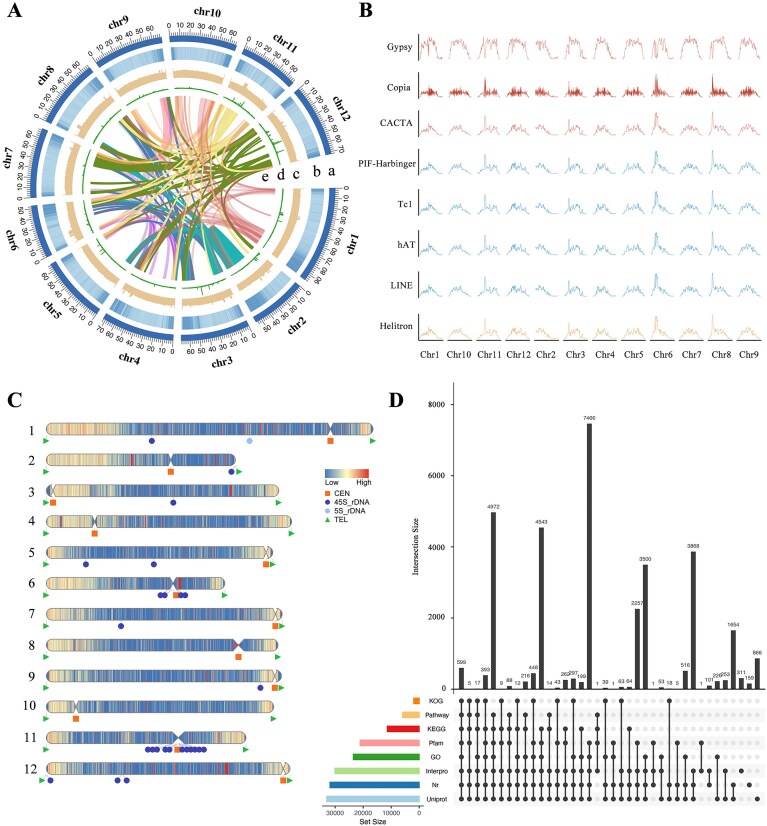
Complete genome assembly and annotation of the VF36 tomato. (A) Circos plot of VF36 genome annotation. Quantitative tracks are aggregated in a 10-kb window. Track a, chromosomes information. Track b, gene density. Track c, GC content. Track d, repeat coverage. Track e, collinearity information. (B) Track displaying density of *Gypsy, Copia, CACTA, PIF-Harbinger, Tc1, hAT, LINE*, and *Helitron* elements. (C) Map of centromere prediction for VF36 genome. (D) Distribution of VF36 genomic features.

To develop a high-quality genome assembly for the “VF36” tomato, different sequencing platforms were employed. A total of 62 Gb of PacBio HiFi reads and 169.51 Gb of ONT ultra-long reads were generated to preassemble the genome. The *N*_50_ length of the HiFi reads was 17.74 kb, and the *N*_50_ length of the ONT reads was 55.07 kb (Supplementary Table S2). NextDenovo was used to assemble the ONT data, forming 26 contigs with an *N*_50_ size of 55.68 Mb. For the PacBio HiFi combined with ONT reads, a genome with a contig *N*_50_ size of 68.44 Mb was assembled (Supplementary Table S3), an increase of approximately 1.6-fold compared with the previous build SL5.0 [25] and 3.8-fold compared with the “Heinz 1706” build SLT1.0 [33]. Moreover, a total of 812.37 Mb Hi-C reads were anchored into 12 pseudochromosomes by assistance with assembly correction (Fig. 1A and Supplementary Fig. S3). After filling all remaining gaps, a gap-less reference genome of “VF36” was generated, with a total length of 815,269,421 bp (Table 1 and Supplementary Table S4).

**Table 1:** Statistics for genome assembly and annotation of VF36 and Heinz 1706 genomes

Genomic feature	VF36 (this study)	Heinz 1706 (SLT1.0)	Heinz 1706 (SL5.0)
Total size of assembly contigs (Mb)	815.27	799.09	801.81
Number of contigs (gaps)	12 (0)	12 (210)	12 (31)
Number of telomeres	24	0	0
Number of centromeres	12	0	0
Number of gene models	34,783	34,384	36,648
Total size of TEs (Mb)	600.23	558.49	491.27
Annotation BUSCOs (%)	98.2	98.2	94.8
Genome BUSCOs (%)	98.27%	97.70%	97.60%

Using telomeric repeats (CCCTAAA at the 5′ end or TTTAGGG at the 3′ end) as sequence queries, we identified 24 telomeric regions among the 12 chromosomes. Plant centromeres typically have a high tandem repeat (TR) density and low gene density. Based on this sequence structure, we identified 9 regions of the tandem repeat clusters (TRCs) on each chromosome that were continuous and occupied the majority of the chromosome (Supplementary Fig. S4). In total, 12 centromeric regions were estimated, with lengths ranging from 0.48 to 3.63 Mb (Supplementary Table S5).

Finally, a gap-free “VF36” tomato genome consisting of 12 T2T chromosomes with 12 centromeric regions was generated (Fig. 1B-1C). BUSCO assessment indicated that 1,586 of the core conserved plant genes (98.27% of 1,614 BUSCOs) were complete in the “VF36” tomato assembly. We assessed the base accuracy of the genome using *k*-mer quality estimation, and the quality values (QVs) ranged from 41.82 to 62.72 for each chromosome. The long terminal repeat (LTR) assembly index (LAI) was used to evaluate genome assembly continuity, which was found to be 12.71. These results indicated the completeness, continuity, and accuracy of the genome assembly.

### “VF36” tomato genome annotation

A total of 600,225,913 bp transposable elements (TEs) were identified, accounting for 73.28% of the assembled “VF36” tomato genome, with LTR elements being the major component at 429.64 Mb. Gypsy-type LTRs (246.25 Mb) were much more abundant than Copia-type LTRs (68.01 Mb) (Fig. 1B and Supplementary Table S6). Moreover, we predicted protein-coding genes from the assembly, resulting in a “VF36” tomato annotation with 34,783 genes using a combination of *ab initio*, homology-based, and transcriptome-based search methods. The average mRNA length was 5.12 kb with 4.8 exons per gene in the “VF36” genome (Supplementary Table S7). The BUSCO assessment indicated that the completeness of the gene set in the annotated genome was 98.2% (Supplementary Table S8).

In total, 33,540 genes (96.43%) were functionally annotated using the National Center for Biotechnology Information nonredundant (NR), UniProt, InterPro, Pfam, Gene Ontology (GO), and Kyoto Encyclopedia of Genes and Genomes (KEGG) databases (Fig. 1D and Supplementary Table S9). We identified 413 microRNAs (miRNAs), 1,049 transfer RNAs (tRNAs), 2,938 ribosomal RNAs (rRNAs), and 582 small nuclear RNAs (snRNAs) in the “VF36” tomato genome (Supplementary Table S10).

### Global comparison of “VF36” and “Heinz 1706” genomes

In comparison to the “Heinz 1706” and the “MicroTom” tomato genome assemblies, the “VF36” genome assembly displayed a greater length than the “Heinz 1706” but was shorter than the “MicroTom” (Table 1) [34]. Notably, 12 T2T chromosomes with 12 centromeric regions were predicted in the “VF36” genome, whereas no telomeres or centromeric regions were identified in the “Heinz 1706” (SLT1.0 and SL5.0) and “MicroTom” (SLM_r2.0) genome assemblies. The SLM_r2.0 genome had 16,700 gaps, SLT1.0 genome version had 210 gaps, and even the substantially more complete SL5.0 version still had 31 gaps, whereas no gaps remained in the “VF36” genome. In addition, BUSCO analysis showed that an average of 98.3% of single-copy genes were completely assembled in the “VF36” genome, which was slightly higher than that in SLT1.0 (97.7%) and SL5.0 (96.2%). Taken together, the “VF36” genome assembly demonstrated higher completeness and accuracy than the “Heinz 1706” and the “MicroTom” assemblies.

**Figure 2:**
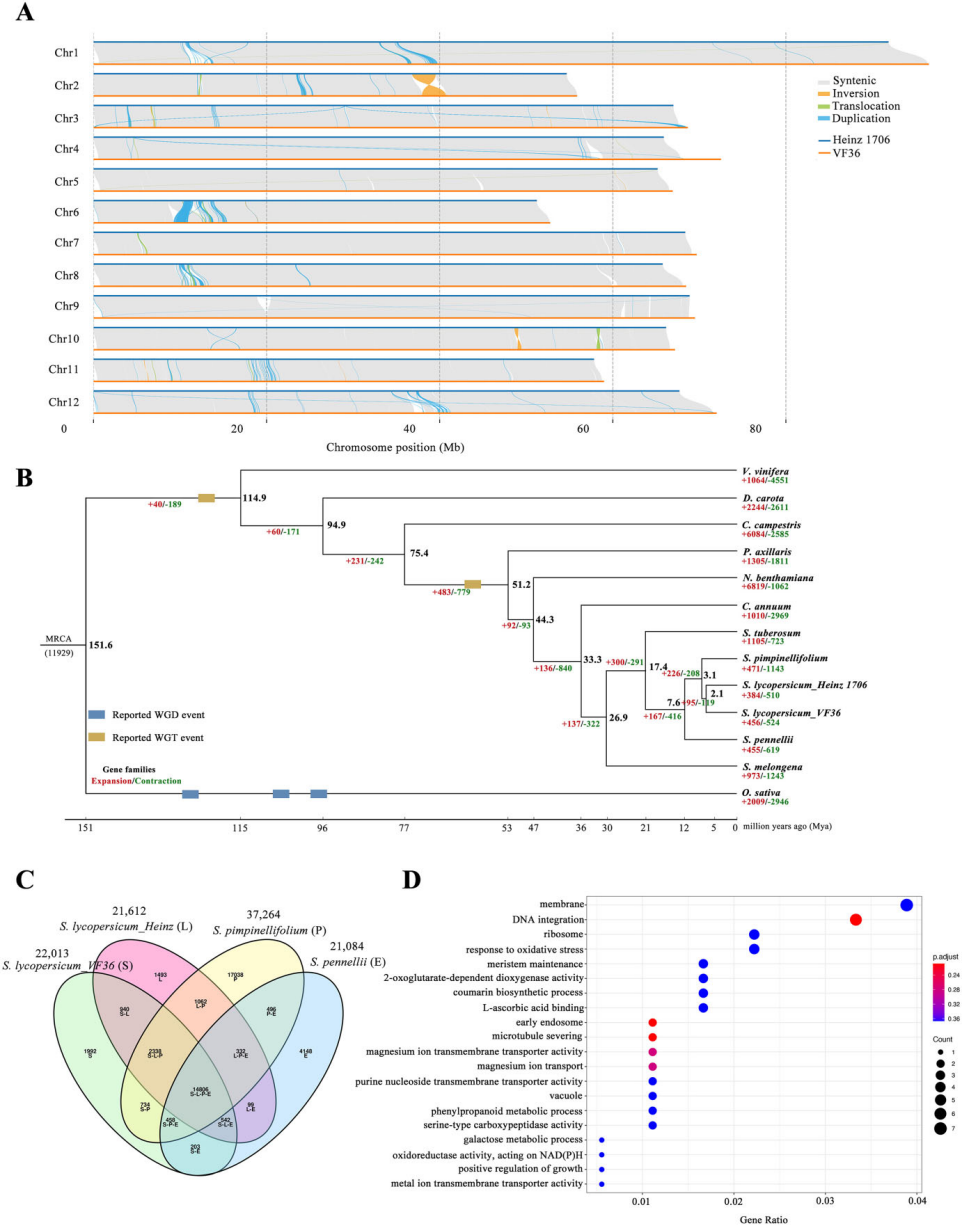
Comparative genomic analysis of the VF36 genome. (A) Structural variations between Heinz_1706 and VF36 genomes. (B) Estimation of divergence time and gene family expansion/contraction. The blue blocks represent the published whole-genome duplication (WGD) events. The dark yellow blocks represent the published whole-genome triplication events. (C) Venn diagram of gene family clustering. (D) The GO enrichment on the genes in SV regions.

Collinearity analysis between the “VF36” and “Heinz 1706” genomes revealed that 99.72% of the “Heinz 1706” genome could be mapped to the “VF36” genome (Fig. 2A). We performed single nucleotide variation (SNV) and short insertion/deletion (indel) analyses, which identified 289,116 SNPs and 103,826 indels through a comparison of the “VF36” and “Heinz 1706” assemblies. Most SNPs (210,357) and indels (74,011) were located in the intergenic regions (Supplementary Table S11). Abundant genetic variations, particularly structural variations (SVs), were detected between the 2 genomes. We predicted 1,807 SVs distributed throughout the genome, with deletions (DELs) and insertions (INSs) accounting for 817 and 705 events, respectively. These DELs and INSs affected 587 functional genes and contributed to the divergence of these 2 accessions. Furthermore, 60.55% of the SVs (1,076) were located in intergenic regions, whereas only 4.76% (86) were located in the coding regions. Among these SVs, 2 large inversions (INVs) (>400 kb) were observed between the 2 genomes. INV299, with a length of 2.78 Mb (chr02: 37,961,912–40,734,631), was predicted between “VF36” and “Heinz 1706” and was annotated as pectate lyase (Supplementary Table S12). Among the identified SV regions, a total of 259 genes were functionally annotated. These genes were involved in a variety of biological processes, including metabolism, environmental information processing, and genetic information processing (Supplementary Table S13). GO enrichment analysis of the genes in the SV regions indicated that the enriched terms were immune response, DNA integration, and metal ion binding (Fig. 2D).

### Phylogenetic analysis

The identification of homologous genes was critical for “VF36” tomato evolutionary analysis. We predicted homologous genes among 13 genomes, including “VF36,” “Heinz 1706,” *Solanum pimpinellifolium, Solanum pennellii, Solanum tuberosum, Solanum melongena, Capsicum annuum, Nicotiana benthamiana, Petunia axillaris, Cuscuta campestris, Daucus carota, Vitis vinifera*, and the outgroup *Oryza sativa* (Supplementary Table S14). As expected, “VF36” clustered together with “Heinz 1706” (Fig. 2B). A total of 527,829 homologous genes were identified and classified into 86,939 gene families. Among these genes, 195,190 were distributed among the 7,593 gene families shared by all 13 genomes (Supplementary Table S15). Based on 52 characterized single-copy and low-copy gene families, we constructed a phylogenetic tree with divergence times. Our findings inferred that “VF36” diverged from “Heinz 1706” tomato 2.1 million years ago (Mya). To provide additional evidence, we analyzed the synonymous substitutions per synonymous site (${K}_S$) between collinear homologous genes between the 2 tomato varieties by analyzing SNP-identified genomic regions. It showed a ${K}_S$ peak of approximately 0.005 between the genome of the “VF36” and the “Heinz 1706” (Supplementary Fig. S5). Using the formula *T* = ${K}_S$/2*r*, we estimated the divergence time between the 2 tomato varieties to be approximately at 1.55 Mya. *C. annuum* was the sister group to the *Solanum* species, and the divergence time of the *C. annuum*–*Solanum* lineage was approximately 33.3 Mya (Fig. 2B).

Gene family expansion and contraction were examined using CAFE. The expanded and unique gene families in the “VF36” tomato were identified as 456/1,786. GO analysis revealed that these expanded genes were involved in the response to auxin, oxidoreductase activity, photosystem II, and protein serine/threonine/tyrosine kinase activity (Supplementary Fig. S6). Unique genes were enriched in transferase activity, protein kinase activity, and methylation (Supplementary Fig. S7).

**Figure 3:**
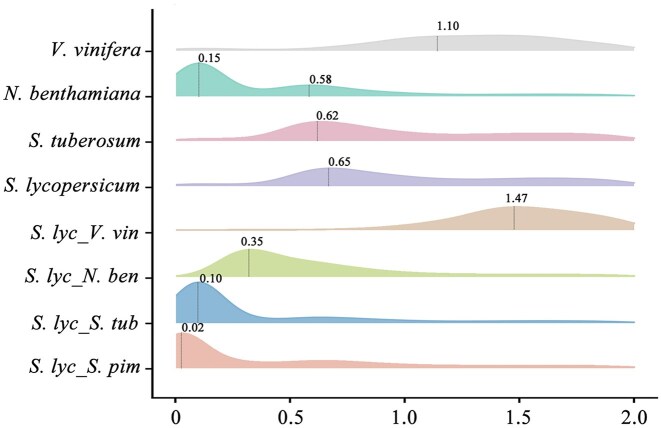
Gene duplication and evolution. ${K}_S$ distribution from orthologs and paralogs among *S. pimpinellifolium, S. tuberosum, N. benthamiana, V. vinifera*, and *S. lycopersicum*.

The distribution of ${K}_S$ between collinear homologous genes was determined. We observed a ${K}_S$ peak of approximately 0.65 in “VF36” tomato, which was also present in other Solanaceae species *S. tuberosum* and *N. benthamiana*, corresponding to the time of the shared family-specific Solanaceae-α hexaploidy event [35] (Supplementary Table S16). A substantial recent ${K}_S$ peak at approximately 0.15 was observed in *N. benthamiana*, consistent with previous studies [36, 37] (Fig. 3). Furthermore, intergenomic gene collinearity between *V. vinifera* and the “VF36” tomato was investigated using a 6:1 syntenic depth ratio (Supplementary Fig. S8). A ratio of 2:1 between *N. benthamiana*–“VF36” tomato and *N. benthamiana*–*S. tuberosum* was characterized (Supplementary Fig. S9). The dot plot of syntenic analysis showed that each fragment in the “VF36” tomato could be identified with the 2 most related syntenic fragments in *N. benthamiana*. Taken together, these results indicated that the recent species-specific whole-genome duplication (WGD) event did not occur in the “VF36” tomato.

### A core circadian oscillator SlPRR1 repressed flowering in tomato

**Figure 4:**
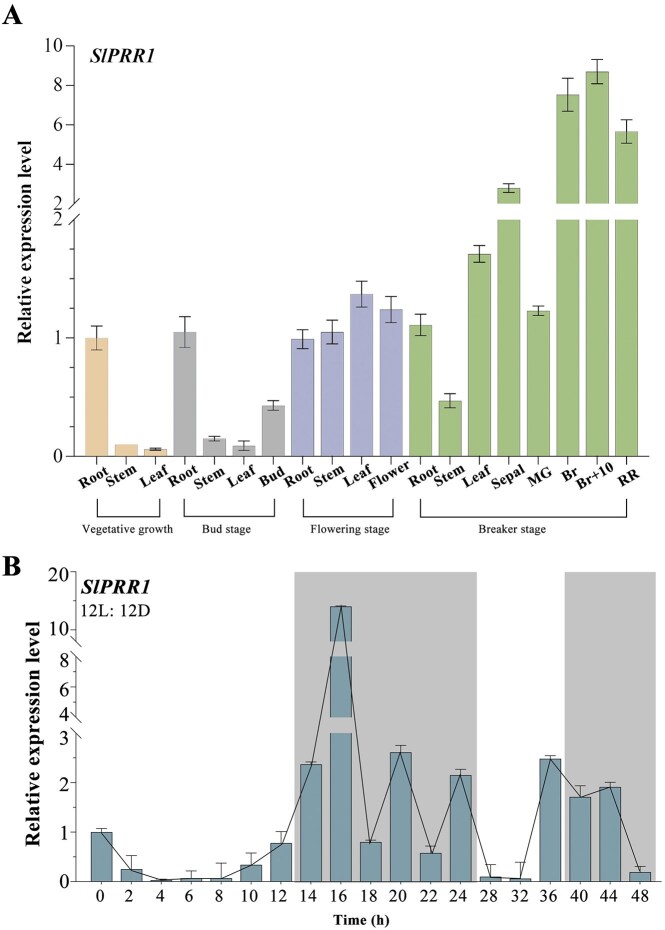
Expression and functional analysis of *SlPRR1* in circadian rhythm. (A) Expression of the *SlPRR1* gene in various stages of tomato tissues. (B) Expression of *SlPRR1* gene from tomato plants grown in 12L/12D. Shading indicates the dark period.

Flowering time is a vital trait in the reproductive success of tomato plants. Photoperiod (day length) regulates plant growth and flowering. PRR1 is a core circadian oscillator essential for plant growth and development. A previous study has shown that SlPRR1 expression has a robust light-dependent circadian rhythm with night-peaking [38]. We have conducted a sequence comparison of the SlPRR1 between the “VF36” and the “Heinz 1706,” and it exhibited very few variations. We identified a nonsynonymous substitution at position 1074, resulting in an amino acid alteration, and a synonymous substitution at position 1608 located in the C-terminal regulatory region (Supplementary Fig. S10). In this study, the expression profiles of *SlPRR1* were analyzed using quantitative reverse transcription PCR, which showed that it was highly expressed in roots and fruits (Fig. 4A). We observed that the expression of *SlPRR1* peaked at night and exhibited a circadian rhythm (Fig. 4B).

**Figure 5:**
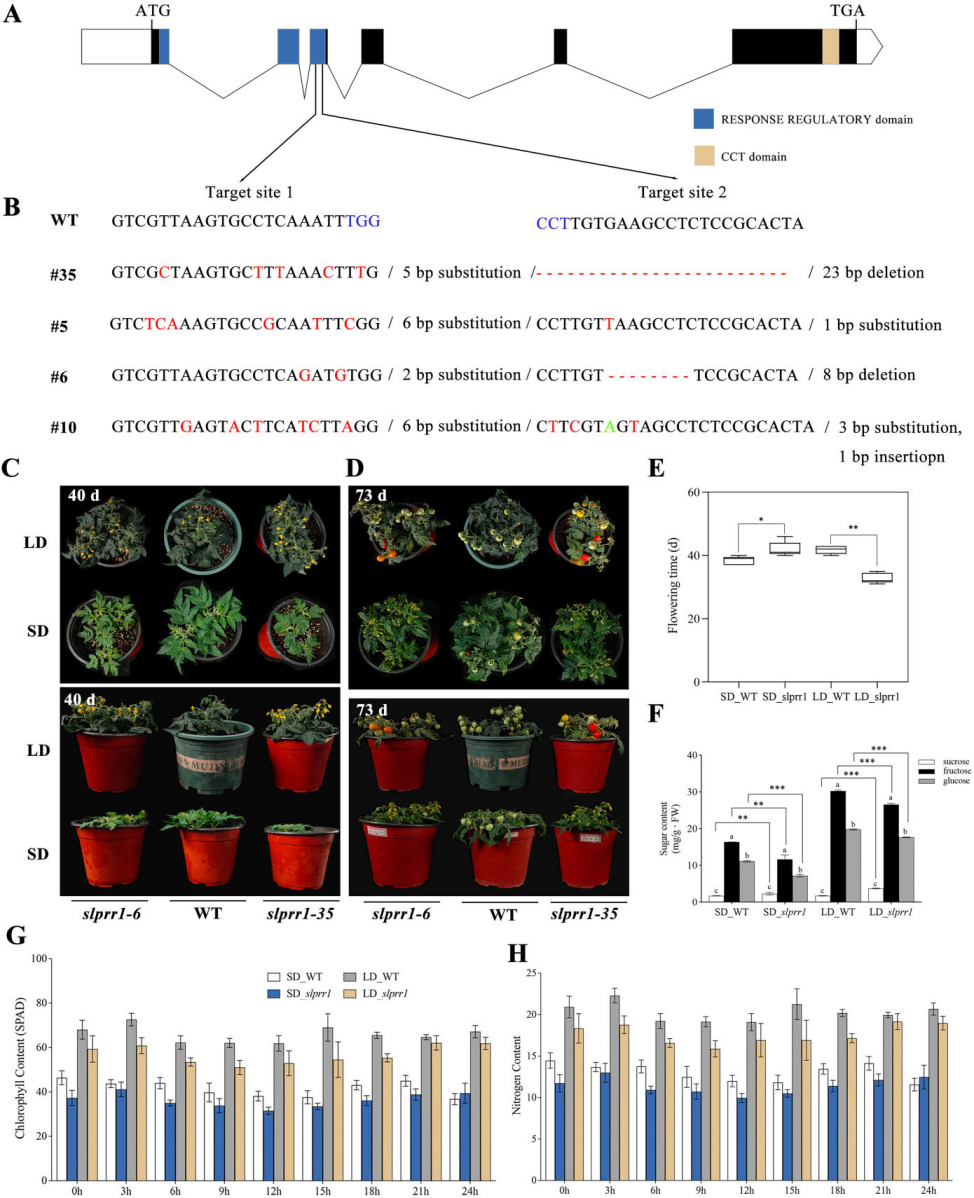
Functional analysis of *SlPRR1* in the regulation of tomato flowering time. (A) The 2 sgRNA target sites in *SlPRR1* locus used for the CRISPR/Cas9 gene-editing system. (B) The mutation types of *SlPRR1* in *slprr1*-35, *slprr1*-5, *slprr1*-6, and *slprr1*-10 line. Red letters indicate the substitution sites, green letters indicate the insertion sites, and blue letters indicate the PAM. (C–E) Flowering phenotype from 40-day-old (C) and 73-day-old (D) tomato plants and flowering time (E) of the WT, *slprr1*-6, and *slprr1*-35 lines under LD (16L/8D) and SD (8L/16D) conditions. (F) The content of sucrose, fructose, and glucose of red ripening tomato fruits in WT and *slprr1* lines under SD or LD conditions. (G, H) The chlorophyll (G) and nitrogen (H) content of tomato leaves in WT and *slprr1* lines under SD or LD conditions during the photoperiod.

To determine the biological role of *SlPRR1* in flowering time, genetic evidence was obtained using the CRISPR/Cas9 gene-editing system. We designed 2 single-guide RNAs (sgRNAs) targeting the third exon of *SlPRR1* (Fig. 5A). Four homozygous mutant lines, designated as *slprr1-35, slprr1-5, slprr1-6*, and *slprr1-10*, were used for further analysis (Fig. 5B). To better investigate the role of *SlPRR1* in day-length responses, we measured the flowering time of control plants (wild type [WT]) and *slprr1* mutant lines under LD and SD conditions. Compared to WT tomatoes, *slprr1* mutant lines showed significantly earlier flowering under LD conditions. In contrast, the *slprr1* mutant lines showed delayed flowering compared to WT plants under SD conditions (Fig. 5C–E).

The *slprr1* mutation caused early flowering under LD conditions and affected the expression of *CO/COL*. We identified 13 *CO/COL* genes in tomatoes, the expression of which increased in *slprr1* mutations, except for *SlCO1, SlCOL10b*, and *SlCOL16b* (Fig. 8K). COs are thought to mediate the circadian clock and control flowering [39]. CO promoted flowering by activating *FT* and *SOC1* expression. We surveyed the expression of a group of genes implicated in the control of flowering time in tomatoes, including *FTL1, J, SFT, SP5G, BOP, TMF, SOC1*, and *FA* (Fig. 8A–J). Among them, the tomato *SP5G* gene, an *FT* paralog, was significantly more highly expressed in LD than in SD. However, transcription of another *FT* paralog, *FTL1*, decreased under LD conditions. These results indicate that SP5G regulates flowering under LD conditions, whereas FTL1 specifically responds to SD. Furthermore, the expression of *SOC1* was significantly downregulated in the *slprr1* mutants under both LD and SD conditions. The expression of *J* and *FUL2* was significantly different between the *slprr1* mutant and WT plants. These data suggest a model for the regulation of flowering time under LD or SD conditions and provide evidence that the core circadian clock gene, *SlPRR1*, regulates flowering time in tomatoes (Fig. 9).

### Knockout of *SlPRR1* affected sugar accumulation in tomato fruit

The sugar content of red ripening fruits, which plays a decisive role in tomato quality, was analyzed in WT and gene-edited fruits under LD and SD conditions [24]. The *slprr1* mutant fruits showed significantly lower fructose and glucose contents under both LD and SD conditions. The sucrose content was significantly higher in *slprr1* mutant fruits than in WT fruits under both LD and SD conditions, although the sucrose content was much lower than that of fructose and glucose in the tomato fruits (Fig. 5F). These results indicated that the knockout of *SlPRR1* promoted flowering under LD conditions and affected fruit flavor in tomatoes.

*LIN5*, a tomato *cell-wall-invertase* gene (*CWIN*), was mapped to a major quantitative trait locus (QTL) determining fruit sugar level [40]. Additionally, *SUCROSE TRANSPORTER 1* (*SUT1*) gene, which was responsible for loading and transporting sucrose from source-to-sink organs, encoded an enzyme involved in tomato sucrose metabolism [41]. The expression levels of both *LIN5* and *SUT1* were significantly higher in *slprr1* mutant lines compared to WT lines under the LD condition (Supplementary Fig. S11A, B). Sugar will eventually be exported transporters (SWEETs) have been verified to mediate sugar transport, with subfamily III members being preferentially explored to transport sucrose in tomato [42]. We surveyed *SWEET*s of subfamily III in tomato, and the expression of *SWEET10b, 11a, 11c*, and *12a* increased in *slprr1* mutations under the LD condition (Supplementary Fig. S11C–L).

### Proposed chlorophyll biosynthesis pathway in tomato leaves

To explore the chlorophyll biosynthesis pathway in tomato leaves, we measured the chlorophyll content in WT and gene-edited plants under LD and SD conditions. Compared to both WT and gene-edited plants grown under SD conditions, tomato leaves exhibited significantly higher chlorophyll contents under LD conditions. The *slprr1* mutants showed slightly lower chlorophyll content than WT plants under both LD and SD conditions.

**Figure 6:**
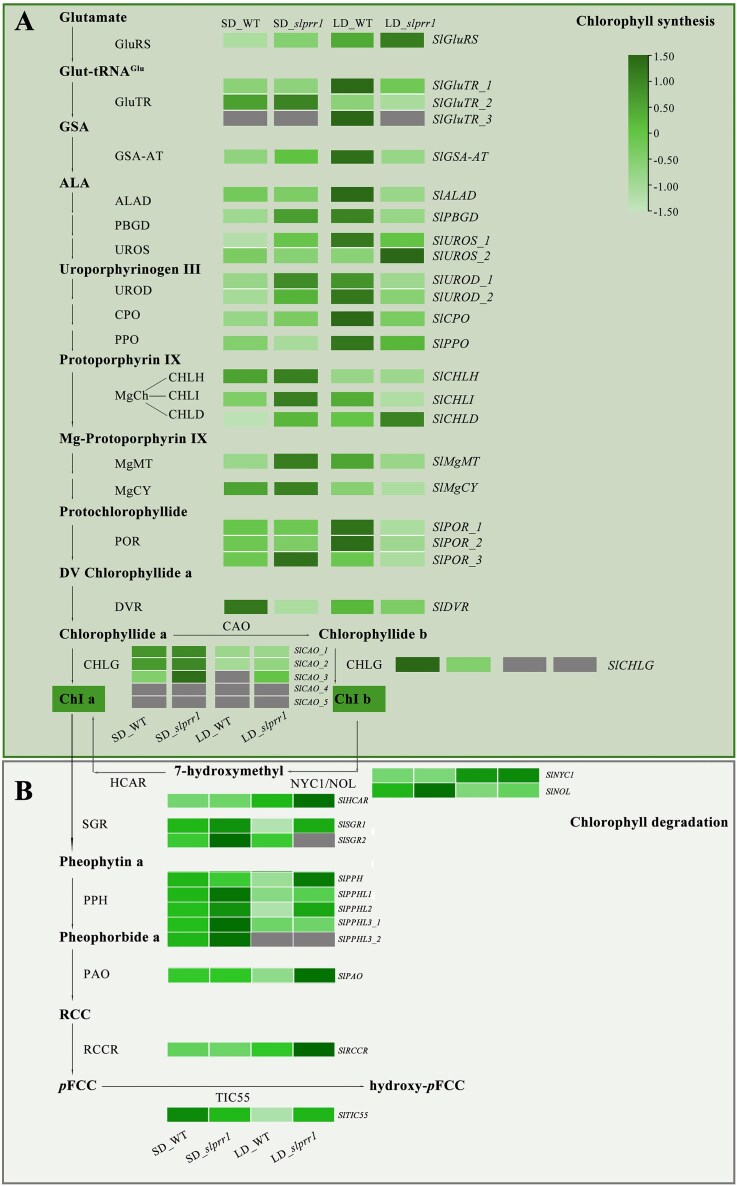
Chlorophyll synthesis and degradation of tomato leaves in WT and *slprr1* lines under SD or LD conditions. (A) The potential simplified chlorophyll synthesis pathway in tomato leaves is depicted. (B) The potential simplified chlorophyll degradation pathway in tomato leaves is depicted.

**Figure 7:**
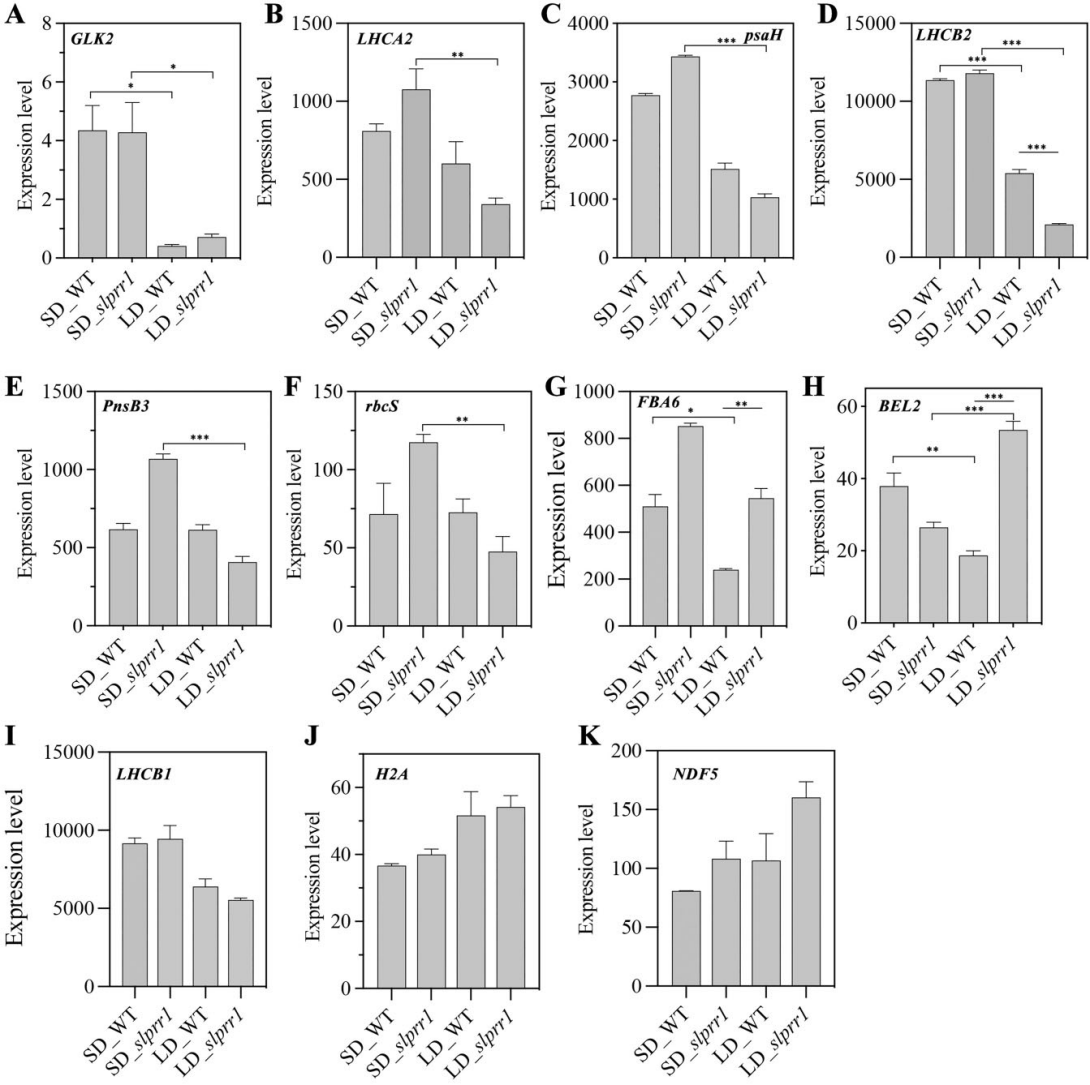
Relative expression levels of photosynthesis and chlorophyll synthesis-related genes in tomato leaves. Error bars represent the averages of 3 biological replicates ± SD. Asterisks indicate statistical significance (*P < 0.05, **P < 0.01, Student’s *t*-test).

The chlorophyll biosynthesis pathway was elucidated, and the enzymes genes were surveyed (Fig. 6A). Among these genes, *SlGluTR_1, SlPPO, SlPOR1*, and *SlPOR2* were expressed at significantly higher levels in WT plants under LD conditions according to transcriptome analysis. To infer the transcription factors (TFs) that modulate the transcription of genes related to chlorophyll metabolism in tomatoes, we analyzed the expression of *SlH2A* and *SlNDF5* (upregulated) and *SlGLK2, SlLHCB*, and *SlpsaH* (downregulated) under LD conditions (Fig. 7A–K). This suggests that *SlH2A* and *SlNDF5* positively regulate the activity of candidate enzymes, whereas *SlGLK2, SlLHCB*, and *SlpsaH* negatively regulate *SlGluTR_1, SlPPO, SlPOR1*, and *SlPOR2*under LD conditions (Fig. 9).

**Figure 8:**
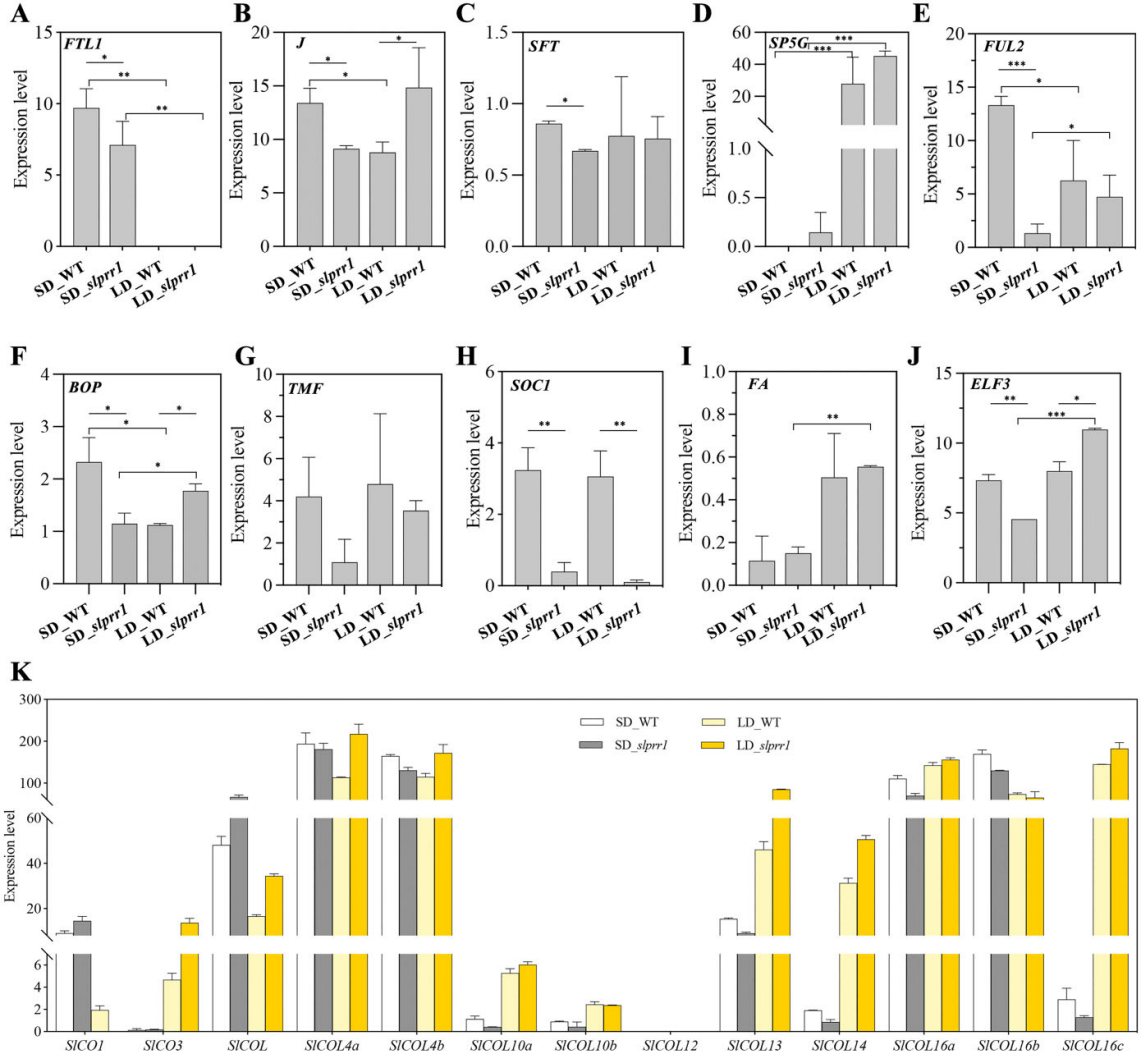
Relative expression levels of flowering-related genes in tomato. Error bars represent the averages of 3 biological replicates ± SD. Asterisks indicate statistical significance (*P < 0.05, **P < 0.01, Student’s *t*-test).

**Figure 9:**
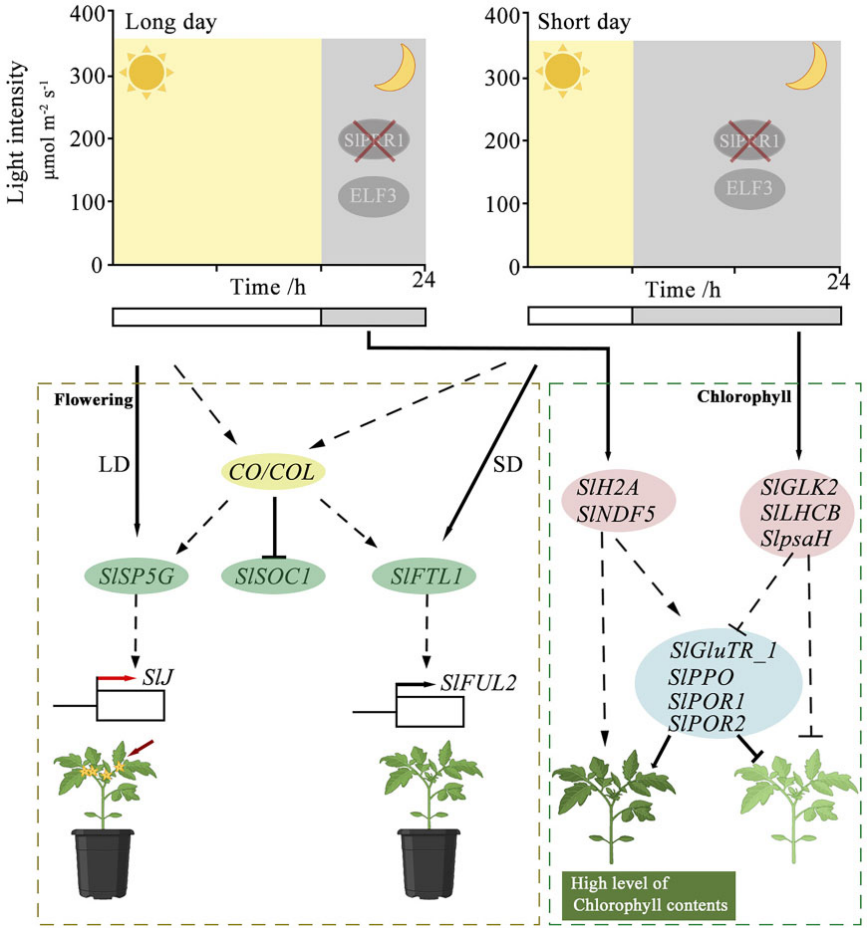
Model for the regulation of flowering time and chlorophyll synthesis by knockout *SlPRR1* in tomato under LD and SD conditions.

## Discussion

A high-quality reference genome is indispensable for identifying traits and facilitating genetic improvements. In this study, we publicly released a T2T gap-free genome of cultivated tomato “VF36,” comprising 815.27 Mb of sequence and 34,783 protein-coding genes. The complete genome sequence of cultivated tomato is the largest reported to date, surpassing several previously released tomato reference genomes [23–25, 43]. The combination of ONT ultra-long, PacBio HiFi, and Hi-C sequencing technologies has overcome the assembly challenges, including the 31 gaps remaining in the SL5.0 version and centromeres. In our cultivated tomato “VF36,” we have successfully corrected numerous misassemblies and filled all chromosomal gaps. The “VF36” displayed more completeness and continuity of the genome assembly than “Heinz 1706,” despite the annotation of a greater number of genes in the SL5.0 version. This discrepancy might be attributed to differences in gene annotation methods, which could lead to variations in the number of detected genes. Higher assembly quality could sometimes result in the merging of fragmented gene models, thereby yielding a more accurate depiction of gene structures but potentially reducing the overall gene count [44]. Additionally, natural genomic variation between cultivars, such as gene loss or segmental duplications, could also contribute to differences in gene counts [45]. Comparison of SVs present in “VF36” and “Heinz 1706” revealed several regions associated with the immune response, DNA integration, and metal ion binding.

Modern cultivated tomatoes are derived from the wild relative, *S. pimpinellifolium*. Wild tomatoes are recognized as SD plants, whereas most cultivated accessions have reduced photoperiodic sensitivity and are considered ND plants [9]. The selection of flowering time has been a major goal in tomato breeding efforts, which have spread cultivars worldwide from their origins. In this study, the core circadian oscillator SlPRR1 exhibited a circadian rhythm with peak expression at night, consistent with previous studies [46]. SlPRR1 was highly expressed in roots during vegetative growth when the tissues were in a dark environment.

The clock ran faster in *slprr1* mutants under LD conditions, whereas the *slprr1*1 mutants exhibited delayed flowering under SD conditions. These results indicate that SlPRR1 regulates flowering time in cultivated tomato plants. CO is a circadian clock-regulated gene that encodes a transcription factor required for flowering [47], which is modulated by day length. Thirteen *CO*/*COL* genes were identified in cultivated tomatoes, most of which were differentially expressed between the LD and SD conditions. A previous study suggested that *SlCOL, SlCOL4a*, and *SlCOL4b* may function as positive regulators of tomato flowering [48]. *CO* and *FT* are the 2 central integrators of the photoperiod pathway that control flowering time, with FT members being the final outputs of the photoperiodic response downstream of CO [49]. As previously reported, we observed several florigen genes involved in the regulation of flowering time. *SP5G* was highly expressed under LD conditions but barely expressed under SD conditions. As an *FT* paralog, *SP5G* is a major locus influencing day-length adaptation in tomatoes, and tomato cultivars contribute to the loss of day length–sensitive flowering by reducing their LD responses. Another *FT* paralog, FTL1, does not control LD flowering in tomatoes and responds specifically to SDs [5]. Consistent with our results, *FTL1* was specifically expressed under SD conditions. Therefore, we propose a model for the regulation of flowering time by SlPRR1 knockout in tomatoes cultivated under LD and SD conditions.

Flowering is an important developmental stage and a clear sign of plants’ transition from the vegetative to the reproductive stage. During the tomato growth cycle, the leaf chlorophyll content changes dynamically through chlorophyll accumulation and degradation. Chlorophyll, the most abundant pigment on Earth, is a key component of photosynthesis and is required for sunlight absorption [50]. Photoperiod regulates plant germination, growth, and flowering, but it affects chlorophyll biosynthesis. In addition to photoreceptors, chloroplasts act as plant light sensors in response to different photoperiods by altering their ultrastructures [51]. Few studies have reported on the regulatory mechanisms of chlorophyll biosynthesis in tomato leaves cultivated under different photoperiods. PRR1, a core member of the circadian oscillator in plants, has been shown to regulate the phase and amplitude of circadian rhythms. Disruption of PRR1 function (*prr1* mutants) results in circadian arrhythmia, which manifests chlorophyll homeostasis, attenuated biosynthesis, and delayed degradation. Concurrently, this circadian perturbation alters carbon partitioning, leading to aberrant sucrose accumulation in source leaves and reduced phloem loading efficiency, likely through misregulation of sucrose transporters [52]. Under LD conditions, the chloroplasts of the growing plants exhibited smaller grana stacks and the chlorophyll contents increased significantly. In the present study, tomato leaves exhibited higher chlorophyll content under LD conditions than under SD conditions. Chlorophyll biosynthesis can be divided into 4 parts: the formation of ALA, biosynthesis of protoporphyrin IX from eight ALA molecules, and biosynthesis of chlorophyll *a* and *b* in the magnesium branch [53]. Moreover, chlorophyll biosynthesis is influenced by several genes such as *GLKs*, which play vital roles in regulating chlorophyll accumulation and chloroplast development in tomato fruits [21]. BEL2 may directly bind to the *GLK2* promoter to repress its transcription in tomatoes [54]. In this study, *GLK2* was highly expressed under SD conditions compared with LD conditions. *BEL2* was highly expressed in *slprr1* mutants under LD conditions and showed a different expression pattern from that of *GLK2* in tomatoes. Chlorophyll synthesis and degradation are dynamic and complex processes influenced by environmental factors such as photoperiod and are regulated by multiple enzymes and regulatory genes.

## Conclusions

In summary, we present a T2T gap-free genome of the cultivated tomato *var*. VF36 using data from PacBio HiFi, ONT ultra-long, and Hi-C technologies. We identified and verified a core circadian oscillator, SlPRR1, and observed that *slprr1* mutant lines exhibited significantly early flowering under LD conditions and delayed flowering under SD conditions compared to WT. Based on these findings, we propose a hypothetical model illustrating how SlPRR1 regulates flowering time and chlorophyll biosynthesis in response to photoperiods. The study provides novel insights into the essential regulatory mechanisms of flowering time and offers potential avenues for manipulating and improving tomato yields.

## Data Description

### Plant materials, growth conditions, and photoperiod treatment

Tomato (*S. lycopersicum*) cultivars “VF36” and “Micro-Tom” were grown in soil in growth chambers at Nanjing Agricultural University. For flowering time assessment, seeds were sown under a light intensity of 20,000 lx (360 μmol m^−2^ s^−1^) at 25°C and 70% relative humidity with different photoperiods: LD (16L/8D), SD (8L/16D), and ND (12L/12D). Flowering time was evaluated as the number of days to reach the first observable flower opening, with at least 3 individual plants used for assessment.

### DNA extraction and sequencing

Leaf samples of the “VF36” inbred line were collected for genome sequencing. DNA extraction was performed using a modified cetyltrimethylammonium bromide (CTAB) method.

The ONT ultra-long library was obtained from the Nanopore sequencing platform and size-selected (>30 kb) using Filtlong (RRID:SCR_024020) (v.2.4) software. The pass reads were then filtered to obtain joint sequences with mean read quality scores above 90% using Porechop (RRID:SCR_016967) (v.2.4). A SMART cell sequencing library containing approximately 15- to 20-kb fragments was constructed and sequenced using PacBio according to the standard protocol [55].

For Illumina short-read sequencing, libraries were constructed using the Nextera DNA Flex Library Prep Kit (Illumina) and sequenced on an Illumina HiSeq 2000 platform (RRID:SCR_020130). Raw reads were filtered and polished using Fastp software (RRID:SCR_016962) (v.21.0). Simultaneously, a Hi-C library was established using an Illumina NovaSeq 6000 platform (RRID:SCR_016387), yielding 662,677,798 bp of clean data. Sequencing was performed at the Wuhan Benagen Technology Co., Ltd. [56].

### Genome initial assembly and assessment

Genome size was estimated based on *k*-mer distribution analysis using Jellyfish (RRID:SCR_005491) (v.2.2.10), and genome heterozygosity was determined using GCE (RRID:SCR_017332) (v.1.0) [57]. After removing low-quality sequences from the ONT ultra-long sequencing data, the initial assembly was performed using NextDenovo (RRID:SCR_025033) (v.2.5) with parameters of read_cutoff =1k, block size = 1 g, nextgraph_options = -a 1. The assembly was corrected for ONT reads using Racon (RRID:SCR_017642) (v.1.4.11) over 2 rounds and further improved using Pilon (RRID:SCR_014731) (v.1.23) with second-generation sequencing data. Additionally, a combined assembly strategy using PacBio HiFi reads was used to obtain a high-accuracy assembly. Hifiasm (RRID:SCR_021069) (v.0.16.1-r375) software was used to assemble the genome using PacBio HiFi reads alone and PacBio HiFi reads combined with ONT ultra-long reads [58]. Initial genome assembly completeness was assessed using the embryophyte_odb10 database of 1,614 single-copy orthologs in BUSCO (RRID:SCR_015008) (v.5.8.2) [59].

A total of 98.70 Gb Hi-C sequencing data assisted in genome assembly through clustering, ordering, orienting, and eliminating redundancy of the contigs using ALLHiC (RRID:SCR_022750) (v.0.9.8), 3D-DNA (RRID:SCR_017227) (v.180419), and Juicer (RRID:SCR_017226) (v.1.6) software. The gaps were filled with 100 N to obtain the final chromosome-level genome sequence. Finally, the accuracy of the Hi-C–based chromosomal assembly was assessed using the HiCExplorer (RRID:SCR_022111) (v.3.6) chromatin contact matrix [60].

Missed telomers were further filled using ONT ultra-long reads with Winnowmap (RRID:SCR_025349) (v.1.11), medaka_consensus (v.1.2.1), and Nucmer (v.3.1), as described by Wang et al. [27]. To further improve the scaffold building and fill these gaps, winnowmap (v.1.11) was used with corrected ONT ultra-long reads and HiFi reads. Genome assembly continuity was assessed based on gap location and number. The completeness of the gene regions was evaluated using BUSCO, as described above. The QV of the genome assembly was estimated using the *k*-mer database of Illumina short reads.

### Genome annotation

Repetitive sequences were identified using homology-based and *de novo* approaches. RepeatModeler (RRID:SCR_015027) (v.2.0.4) and LTR_FINDER were used to build a *de novo* TE library [61]. RepeatMasker (RRID:SCR_012954) (v.4.1.5) was applied to identify TEs from the RepBase TE library combined with the *de novo* TE library [62]. Finally, the nonredundant combined TE sets were generated.

Gene structure annotation was performed using *ab initio*, homology-based, and transcriptome-based prediction methods. Homologies from 5 species (*Solanum chmielewskii, Solanum galapagense, S. lycopersicum* var. “Heinz1706,” *S. pimpinellifolium*, and *A. thaliana*) were collected as protein evidence for the predicted gene sets using Exonerate (RRID:SCR_016088) (v.2.4). The TransDecoder (RRID:SCR_017647) (v.5.7) pipeline was used to assemble RNA sequencing reads into the transcripts. *Ab initio* gene prediction was performed using Augustus (v.3.5.0) and Glimmerhmm (RRID:SCR_002654) (v.3.0.4) [63]. All predictions were integrated into a comprehensive protein-coding gene set using Maker (RRID:SCR_005309) (v.3.01.03) [64]. Gene function annotation was performed using homology searches against the public databases, including NCBI NR, UniProt, InterPro, Pfam, GO, and KEGG databases.

The ncRNAs, including tRNAs, rRNAs, miRNAs, and snRNAs, were predicted. The tRNAscan-SE (RRID:SCR_008637) (v.2.0.12) was used to identify tRNAs with default parameters, RNAmmer (RRID:SCR_017075) (v.1.2) was used to search for rRNAs, and INFERNAL (RRID:SCR_011809) (v.1.1.4) was applied to identify miRNAs or snRNAs based on the Rfam database (RRID:SCR_007891) [65].

### Identification of centromeres and telomeric sequences

Centromeric regions consist of TRs, centromeric retrotransposons, and low-copy sequences. For telomere identification, the plant telomere sequence 5′-CCCTAAA-3′ was used. Furthermore, TRF software was used to search for TR sequences, and the denser regions of the TRCs tended to be in the centromere regions.

### Synteny analysis and identification of SNPs, indels, and SVs

MUMmer was used to perform genomic collinearity analysis between the “VF36” and “Heinz 1706” genomes [66]. SNPs, short indels, and SVs were analyzed using SyRI [67]. Annotations were obtained using the ANNOVAR software toolkit.

SVs were classified into 5 types: inversions, translocations, duplications, deletions, and insertions. Based on the overlapping regions in the genome, we calculated the number of SVs in the coding regions, introns, 2 kb upstream, 2 kb downstream, and intergenic regions. Compared with the “VF36” genome, the identified genes affected by SVs were subjected to GO and KEGG enrichment analyses.

### Phylogenetic, gene family expansion/contraction analysis

To infer the evolutionary history of the “VF36” tomato, we identified homologs and single-copy orthologous genes using OrthoFinder software in *S. lycopersicum* var. “Heinz1706,” *S. pimpinellifolium, S. pennellii, S. tuberosum, S. melongena, C. annuum, N. benthamiana, P. axillaris, C. campestris, D. carota, V. vinifera*, and the outgroup *O. sativa*. Single-copy genes were aligned using MUSCLE (RRID:SCR_011812) (v3.8.31). A phylogenetic tree was constructed using the maximum likelihood method in the RAxML software with JTT models for amino acid data [68]. MCMCTREE in PAML was then used to estimate the divergence time [55].

The gene family expansion and contraction were analyzed by computational analysis of gene family evolution (CAFE v.3.1) with default parameters in “VF36” tomato compared with that in other 12 species [69].

### Synteny and WGD analysis

Syntenic gene pairs between “VF36” tomato, *S. lycopersicum* var “Heinz1706,” *S. pimpinellifolium, S. pennellii, S. tuberosum, S. melongena, C. annuum, N. benthamiana, P. axillaris, C. campestris, D. carota, V. vinifera*, and *O. sativa* were identified using JCVI (RRID:SCR_021641) (v.0.9.13) [70].

WGDs or polyploidy were prevalent and estimated using synonymous substitutions per synonymous site (${K}_S$) values. ${K}_S$ estimates for pairwise comparisons (one-to-one orthologs between species) were obtained using the PAML package in yn00.

### CRISPR plasmid construction and stable tomato transformation

For the CRISPR/Cas9 construct, 2 specific sgRNAs in the exon of the tomato *SlPRR1* gene were designed using the CRISPR-GE online tool (http://skl.scau.edu.cn/targetdesign). Two sgRNA expression cassettes, combined with 2 target sites, were driven by *AtU6*, and assembled into the pHSbdcas9i vector [71]. The confirmed constructs were transformed into *Agrobacterium tumefaciens* GV3101. These constructs were introduced into the tomato cultivar Micro-Tom via *A. tumefaciens*–mediated transformation. All primers used are listed in Supplementary Table S17. Homozygous T_1_ transgenic plants were used for phenotypic characterization.

### Chlorophyll contents, sugar extraction, and measurement

Chlorophyll content was measured using a Plant Nutrition Tester (SPAD 502; Beijing Zhongke Weihe Technology Development Co., Ltd.). Soluble sugars were determined using a high-performance liquid chromatography (HPLC) system (Waters Corp.) equipped with an Acquity UPLC BEH amide column and evaporative light-scattering detector. Samples were collected from red ripe tomato fruits, immediately frozen in liquid nitrogen, and stored at −80°C. Soluble sugars were extracted and analyzed as previously described. Briefly, freeze-dried samples were added to 5 mL of distilled water, homogenized for 1 minute, and extracted in a water bath at 80°C for 30 minutes. The supernatant was collected by centrifugation and filtered through a 0.45-μm membrane to determine the soluble sugar content in the filtrate.

### Statistical analysis

Significance tests were performed using Prism 9 (GraphPad Software) based on Student’s *t*-test at *P* < 0.01 or *P* < 0.05. Data are presented as means ± standard deviations.

## Supplementary Material

giaf058_Supplemental_Files

giaf058_Authors_Response_To_Reviewer_Comments_original_submission

giaf058_GIGA-D-24-00568_original_submission

giaf058_GIGA-D-24-00568_Revision_1

giaf058_Reviewer_1_Report_Original_Submission Jose Jimenez-Gomez -- 2/3/2025

giaf058_Reviewer_2_Report_Original_Submission Tao Lin -- 2/8/2025

giaf058_Reviewer_3_Report_Original_Submission Alessandro Cestaro -- 2/26/2025

## Data Availability

The genome data are deposited in NCBI with BioProject number PRJNA1204391 and BioSample accession number SAMN46040925. All additional supporting data are available in the *GigaScience* repository, GigaDB [72].

## Additional Files

**Supplementary Table S1**. *K*-mer statistics of the genomic characteristics of “VF36” tomato obtained by genome.

**Supplementary Table S2**. Summary of sequencing data.

**Supplementary Table S3**. Summary of assembly data.

**Supplementary Table S4**. General statistics of the telomeres for the VF36 genome.

**Supplementary Table S5**. Predicted centromeric regions.

**Supplementary Table S6**. Statistics of repeat content in the VF36 genome.

**Supplementary Table S7**. General statistics of predicted protein-coding genes.

**Supplementary Table S8**. BUSCO analysis of VF36 genome completeness.

**Supplementary Table S9**. Functional annotation of the predicted genes.

**Supplementary Table S10**. The statistics of noncoding RNA in the VF36 genome.

**Supplementary Table S11**. The statistics of variation between the VF36 and Heinz1706 genomes.

**Supplementary Table S12**. Structure variations between the VF36 and Heinz1706 genomes.

**Supplementary Table S13**. The influenced genes by structure variations between the 'VF36' and the 'Heinz1706' genomes.

**Supplementary Table S14**. Gene sets used in this study.

**Supplementary Table S15**. Summary of clustered gene families by OrthoFinder among 13 species.

**Supplementary Table S16**. The information of collinear gene pairs.

**Supplementary Table S17**. The primers used in this study.

**Supplementary Fig. S1**. “VF36” tomato fruits of MG and RR stages.

**Supplementary Fig. S2**. *K*-mer = 19 depth and *k*-mer number frequency distribution map.

**Supplementary Fig. S3**. Twelve pseudomolecules scaffolding with Hi-C data.

**Supplementary Fig. S4**. Density of genes and TRs. Blue lines represent LTR/Copia, orange lines represent LTR/Gyps, dark red lines represent TRF, and green lines represent genes.

**Supplementary Fig. S5**. ${K}_S$ distribution from putative collinear homologous genes between the “VF36” and the “Heinz 1706” tomato.

**Supplementary Fig. S6**. GO enrichment of expanded gene families.

**Supplementary Fig. S7**. GO enrichment of unique genes.

**Supplementary Fig. S8**. Syntenic dot plot of *V. vinifera* versus “VF36” tomato.

**Supplementary Fig. S9**. Syntenic dot plot of *N. benthamiana* versus “VF36” tomato.

**Supplementary Fig. S10**. Sequence alignment of SlPRR1 from the “VF36” and the “Heinz 1706” tomatoes.

**Supplementary Fig. S11**. Relative expression levels of *LIN5, SUT1*, and *SWEET* genes in tomato.

## Abbreviations

ALA: 5-aminolevulinic acid; BUSCO: Benchmarking Universal Single-Copy Orthologs; Chl: chlorophyll; CTAB: cetyltrimethylammonium bromide; GO: Gene Ontology; Hi-C: high-throughput chromosome conformation capture; HiFi: high-fidelity; indel: insertion/deletion; KEGG: Kyoto Encyclopedia of Genes and Genomes; LAI: long terminal repeat assembly index; LD: long day; LTR: long terminal repeat; miRNA: microRNA; Mya: million years ago; ND: day-neutral; NR: nonredundant; ONT: Oxford Nanopore Technology; QTL: quantitative trait locus; QV: quality value; rRNA: ribosomal RNA; SD: short day; sgRNA: single-guide RNA; SNP: single nucleotide variation; snRNA: small nuclear RNA; SV: structural variation; SWEETs: sugar will eventually be exported transporters; T2T: telomere-to-telomere; TE: transposable element; TF: transcription factor; TR: tandem repeat; TRC: tandem repeat cluster; tRNA: transfer RNA; WGD: whole-genome duplication; WT: wild type.
