# Supplementary figures and images for: A telomere-to-telomere gapless genome reveals SlPRR1 control of circadian rhythm and photoperiodic flowering in tomato
Source: Gigascience. 2025 Jul 2;14:giaf058. doi: 10.1093/gigascience/giaf058 (PMC12218202; doi:10.1093/gigascience/giaf058)

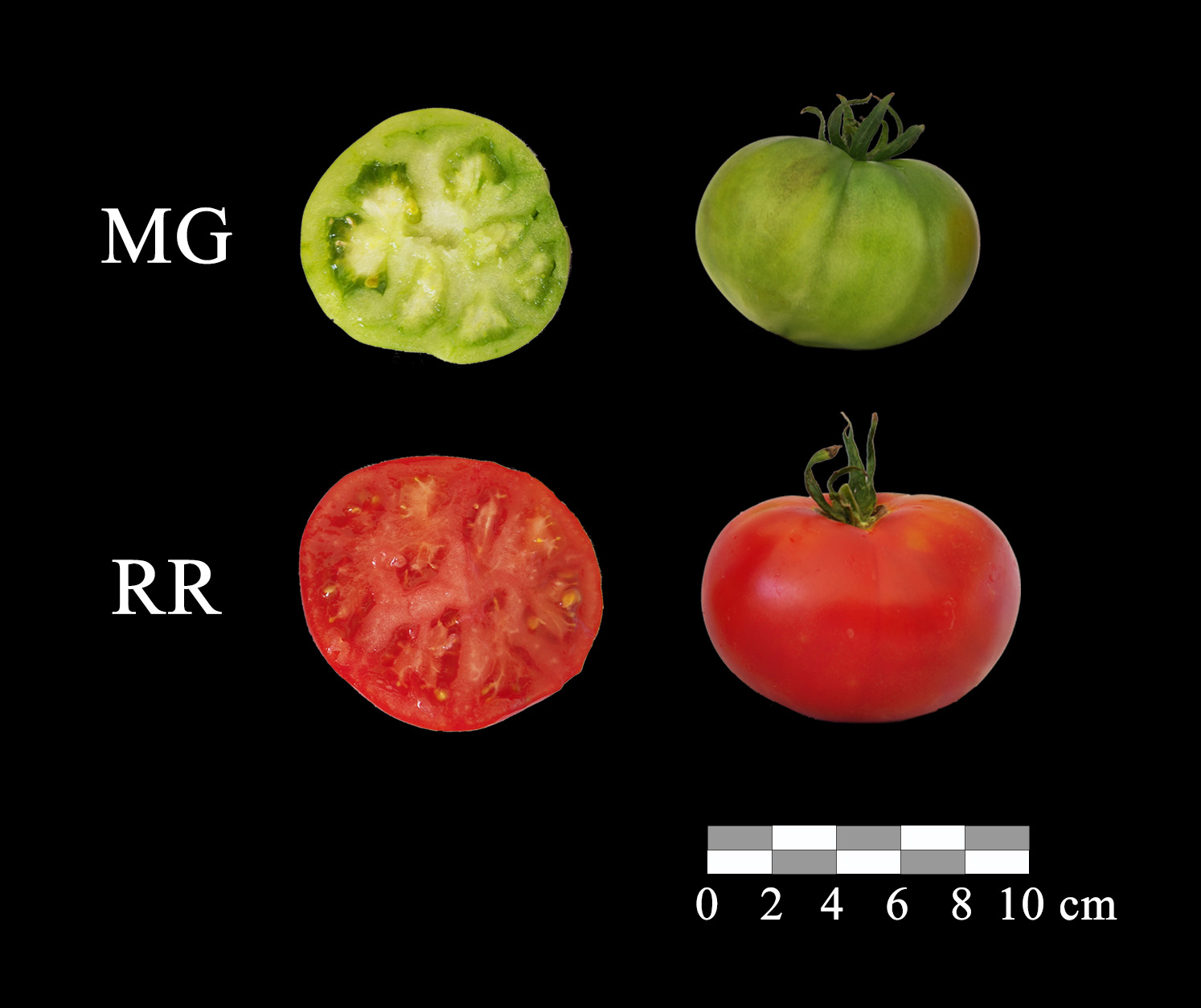

Supplement: giaf058_Supplemental_Files [file giaf058_supplemental_files.zip › Figure S1.jpg]

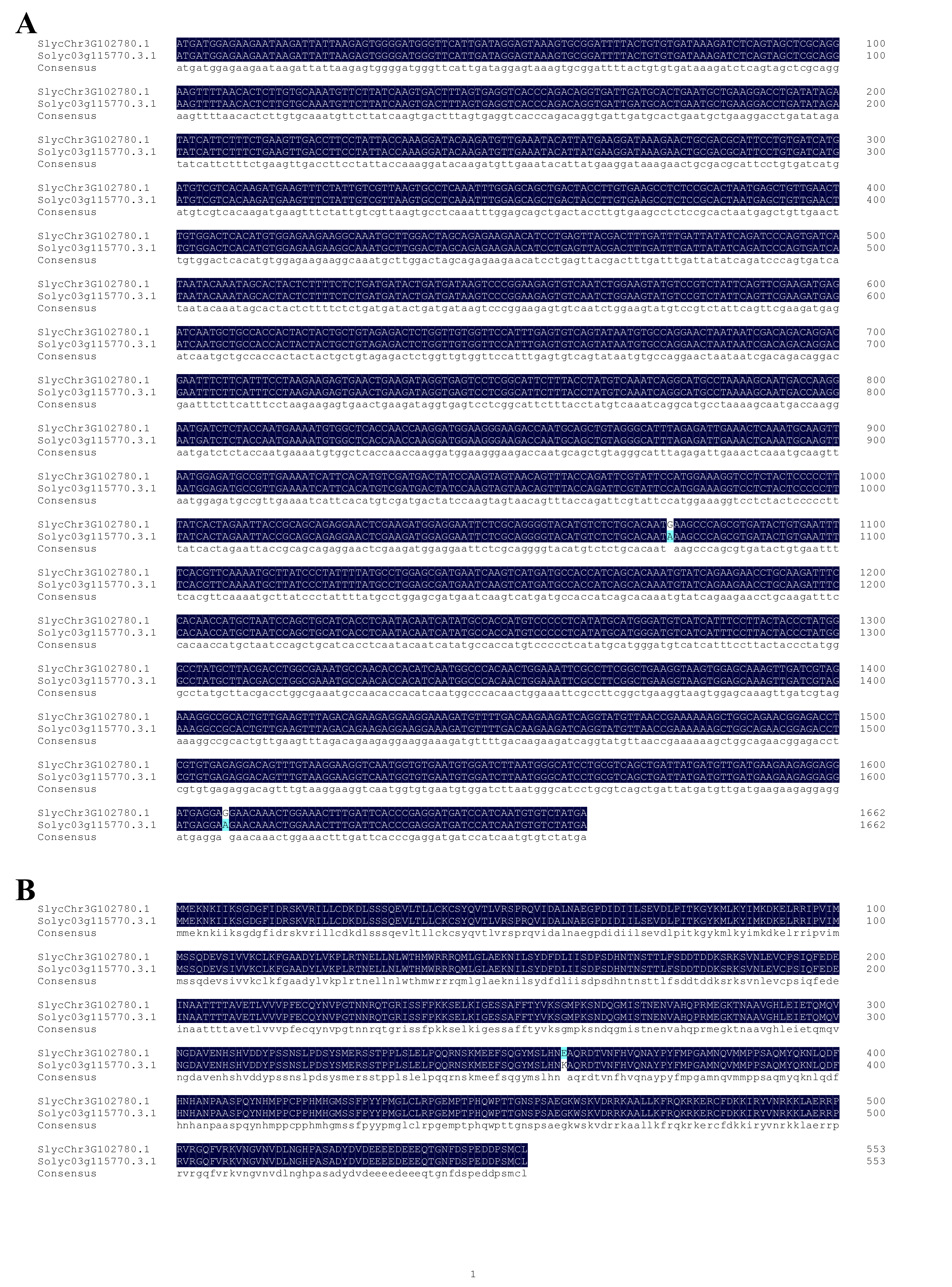

Supplement: giaf058_Supplemental_Files [file giaf058_supplemental_files.zip › Figure S10.jpg]

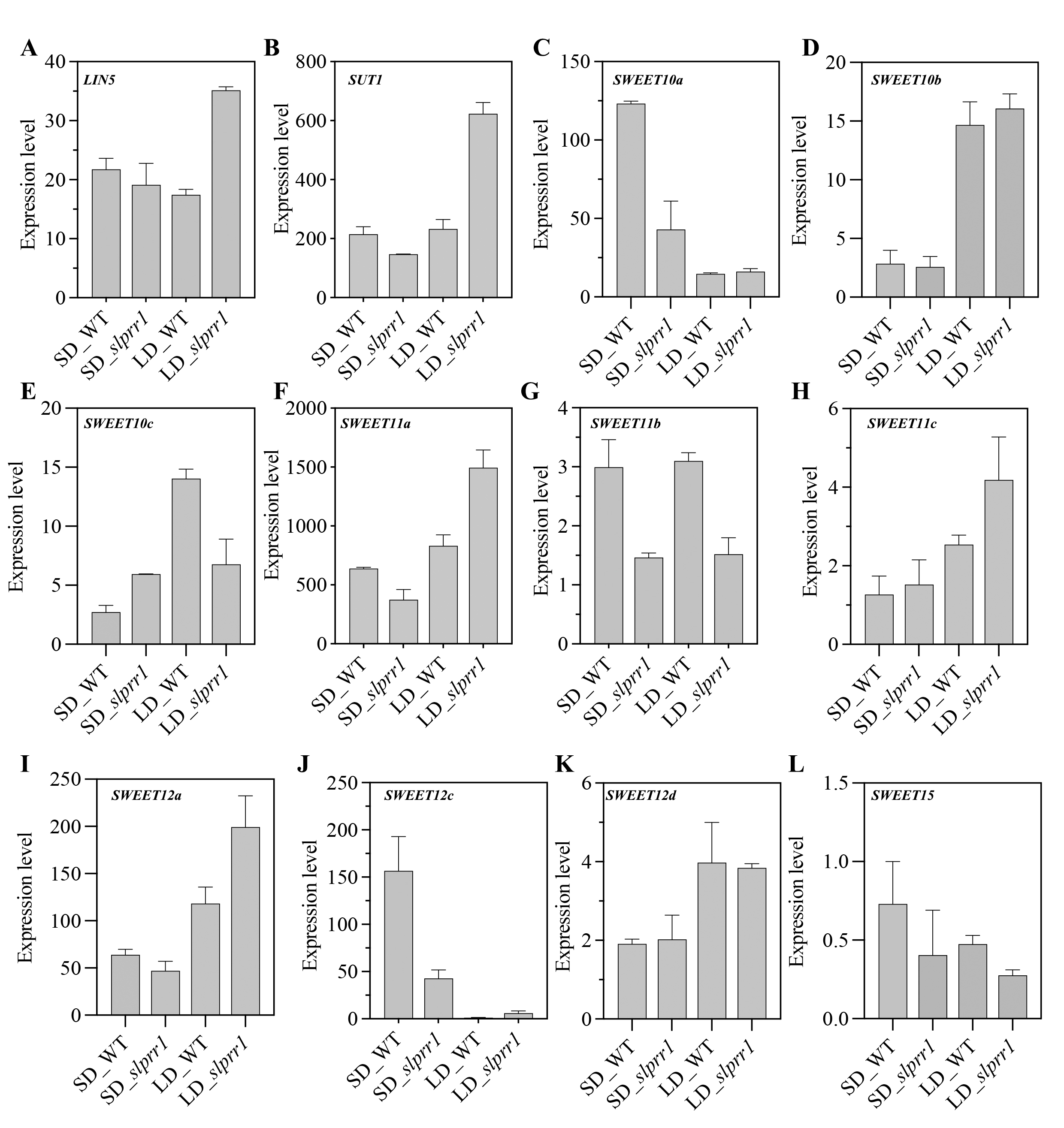

Supplement: giaf058_Supplemental_Files [file giaf058_supplemental_files.zip › Figure S11.jpg]

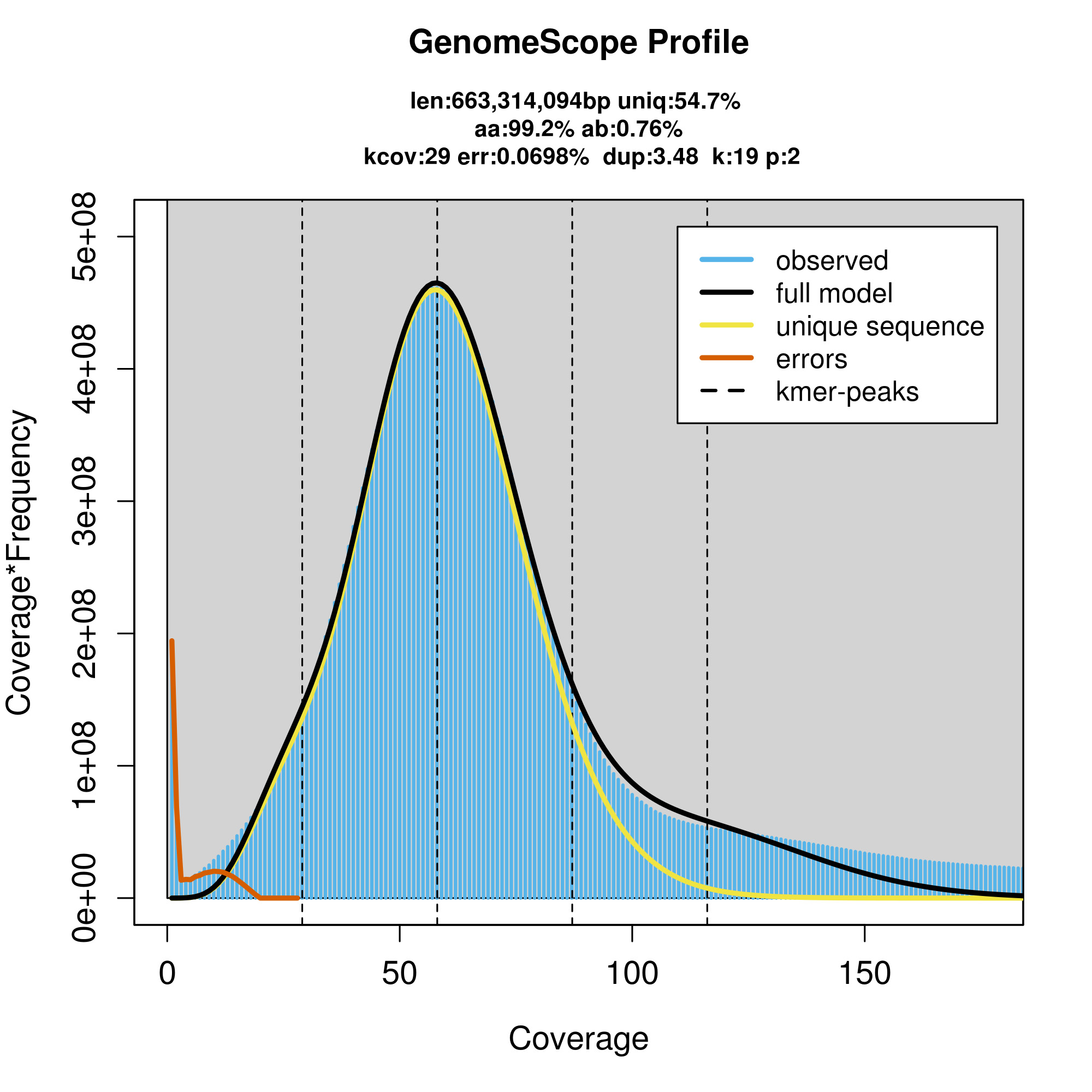

Supplement: giaf058_Supplemental_Files [file giaf058_supplemental_files.zip › Figure S2.jpg]

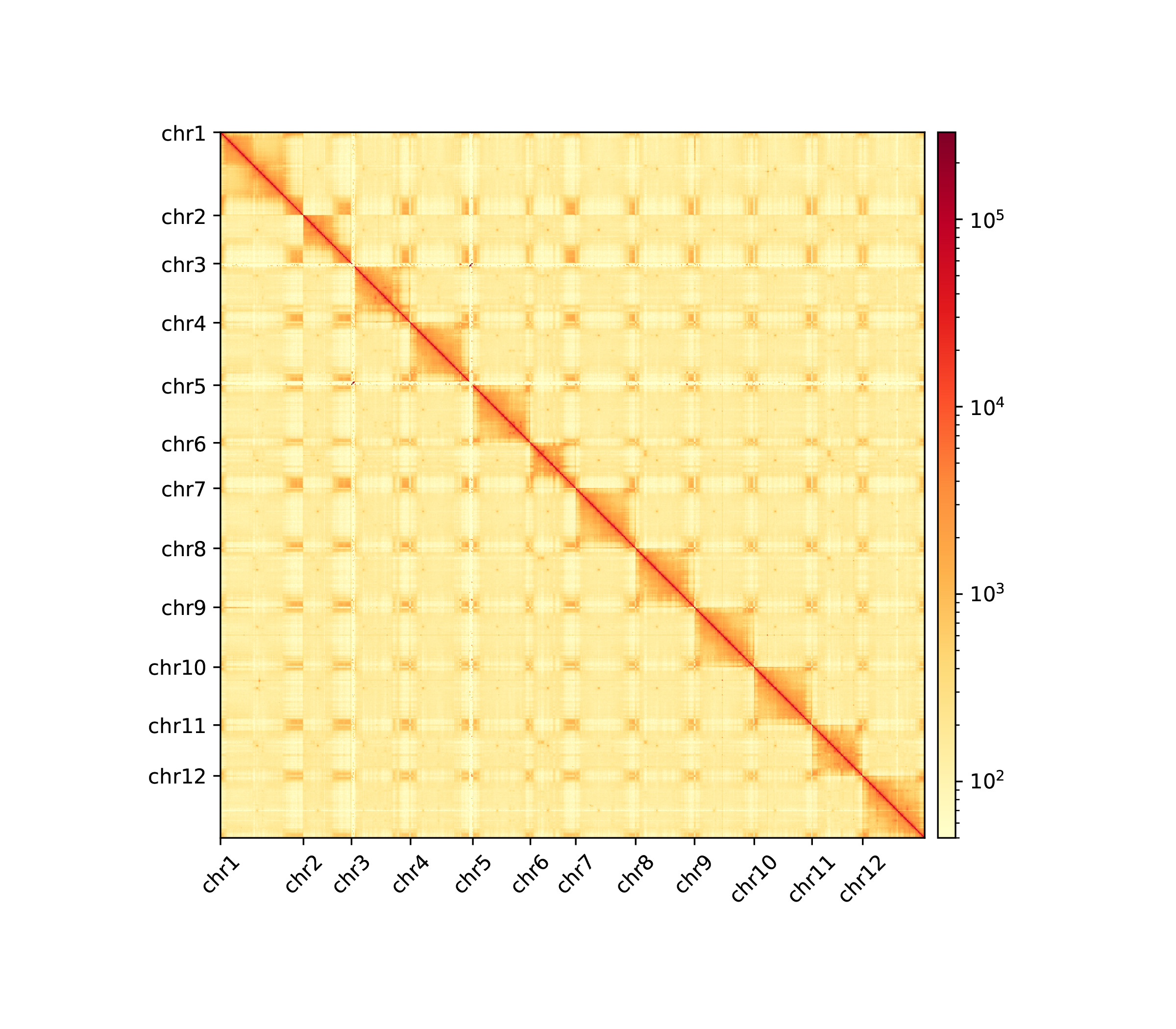

Supplement: giaf058_Supplemental_Files [file giaf058_supplemental_files.zip › Figure S3.jpg]

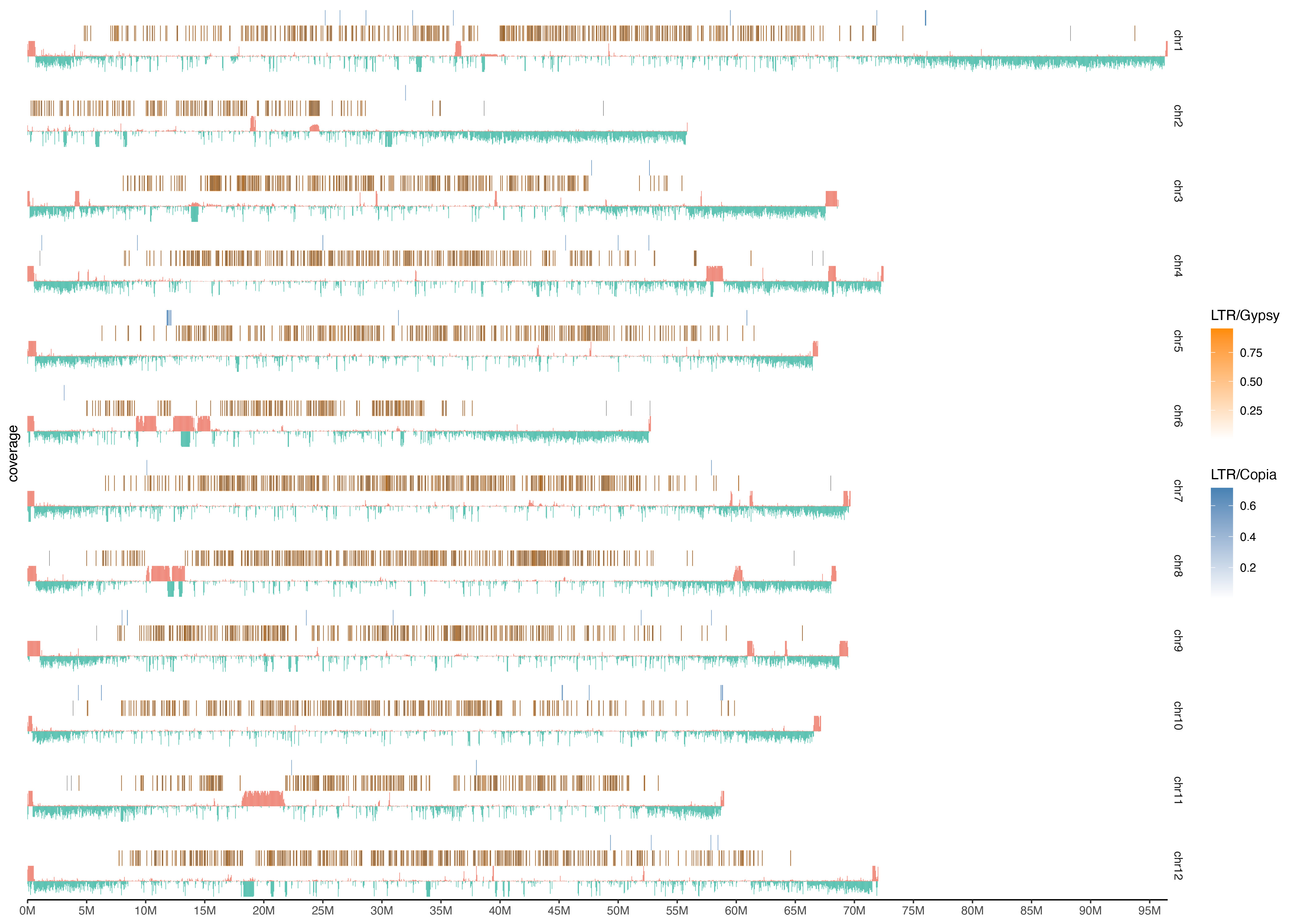

Supplement: giaf058_Supplemental_Files [file giaf058_supplemental_files.zip › Figure S4.jpg]

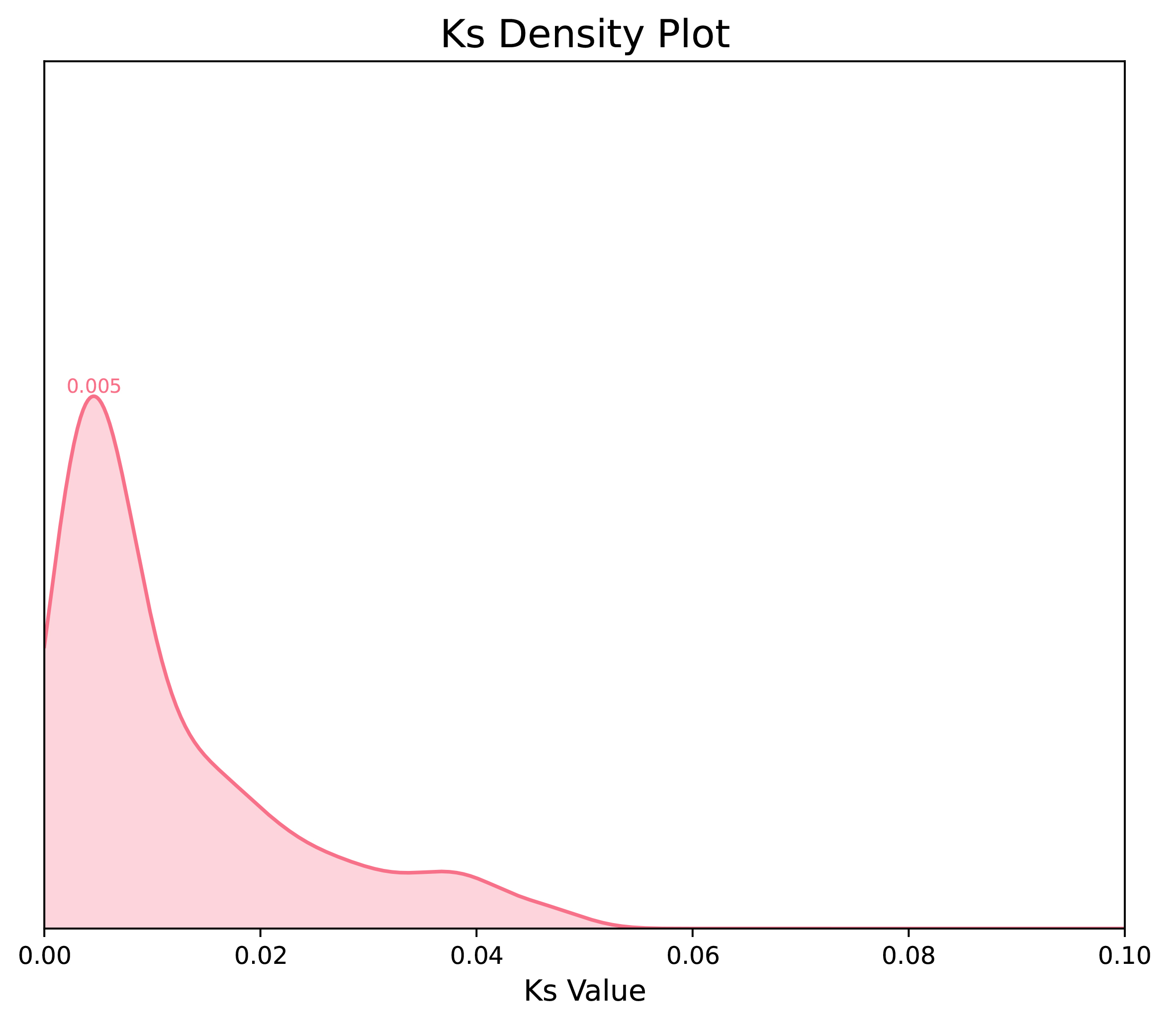

Supplement: giaf058_Supplemental_Files [file giaf058_supplemental_files.zip › Figure S5.jpg]

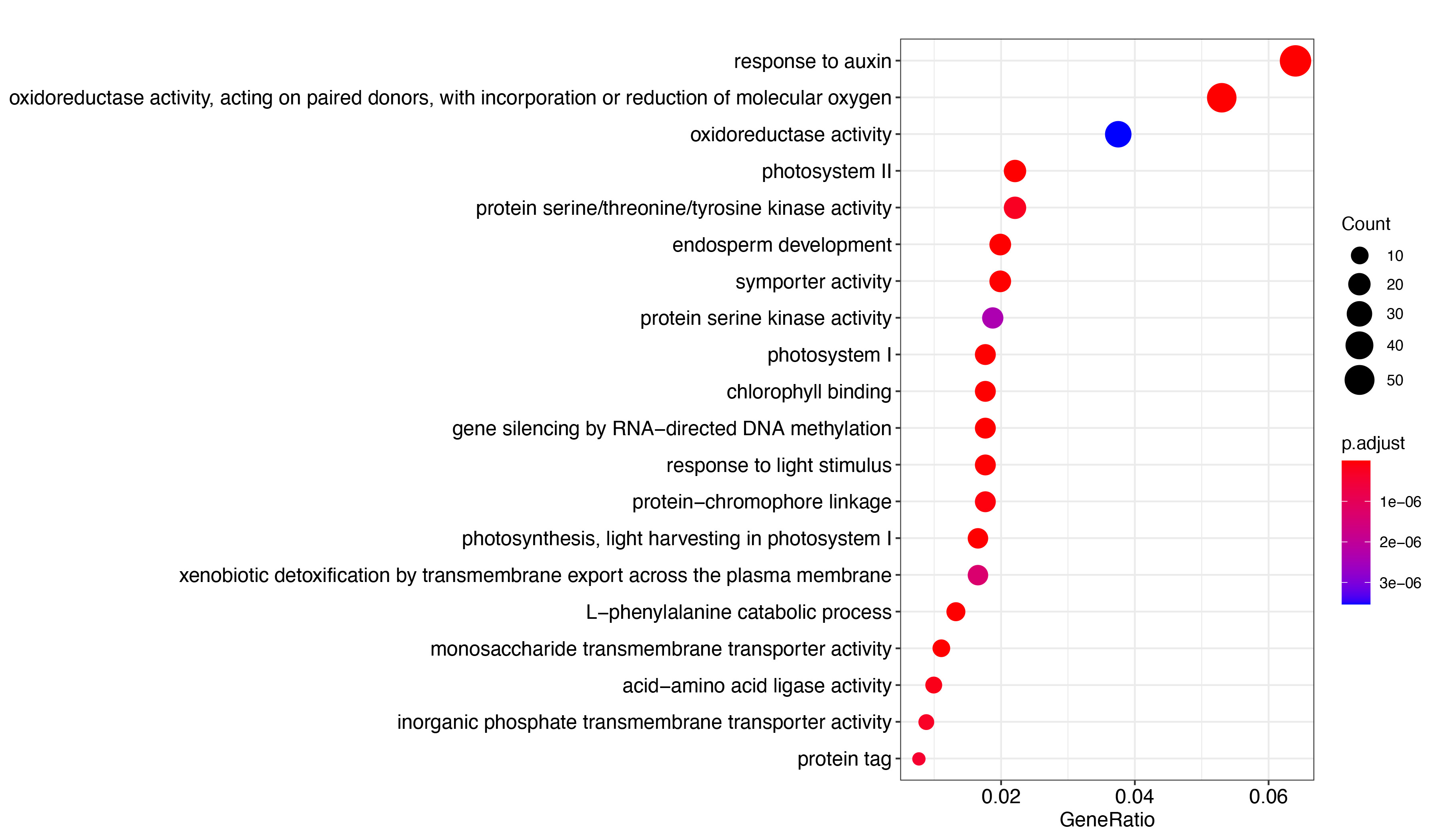

Supplement: giaf058_Supplemental_Files [file giaf058_supplemental_files.zip › Figure S6.jpg]

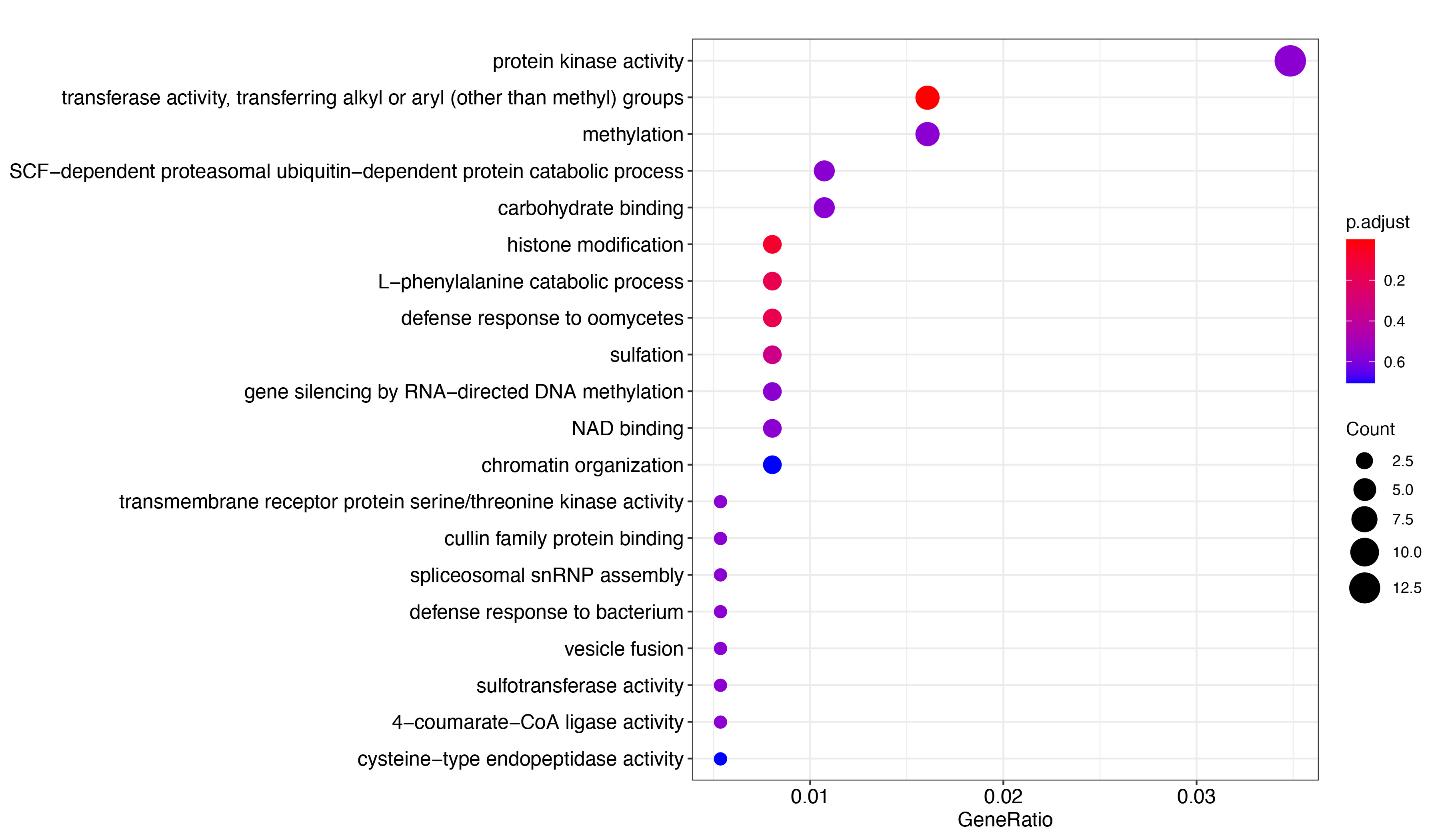

Supplement: giaf058_Supplemental_Files [file giaf058_supplemental_files.zip › Figure S7.jpg]

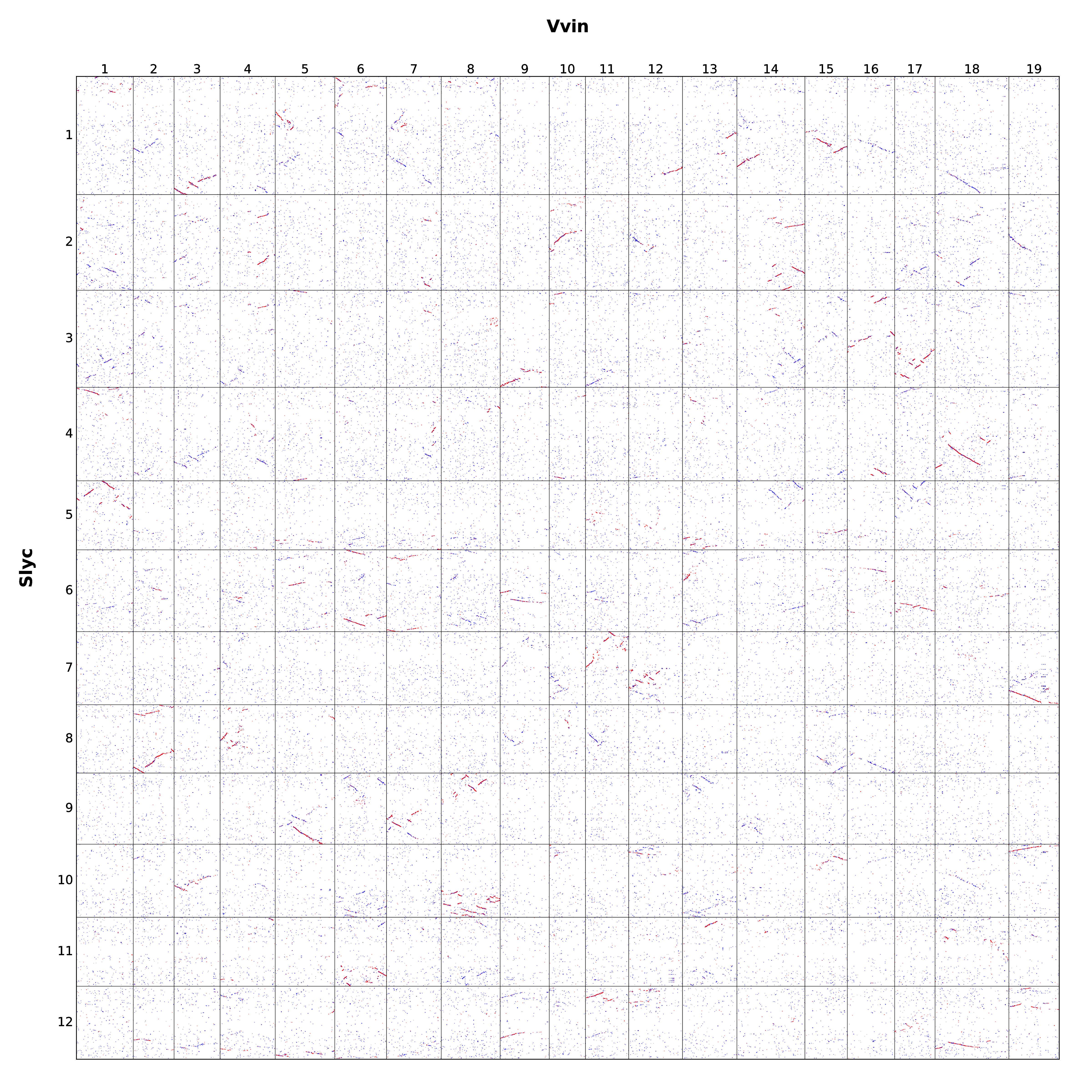

Supplement: giaf058_Supplemental_Files [file giaf058_supplemental_files.zip › Figure S8.jpg]

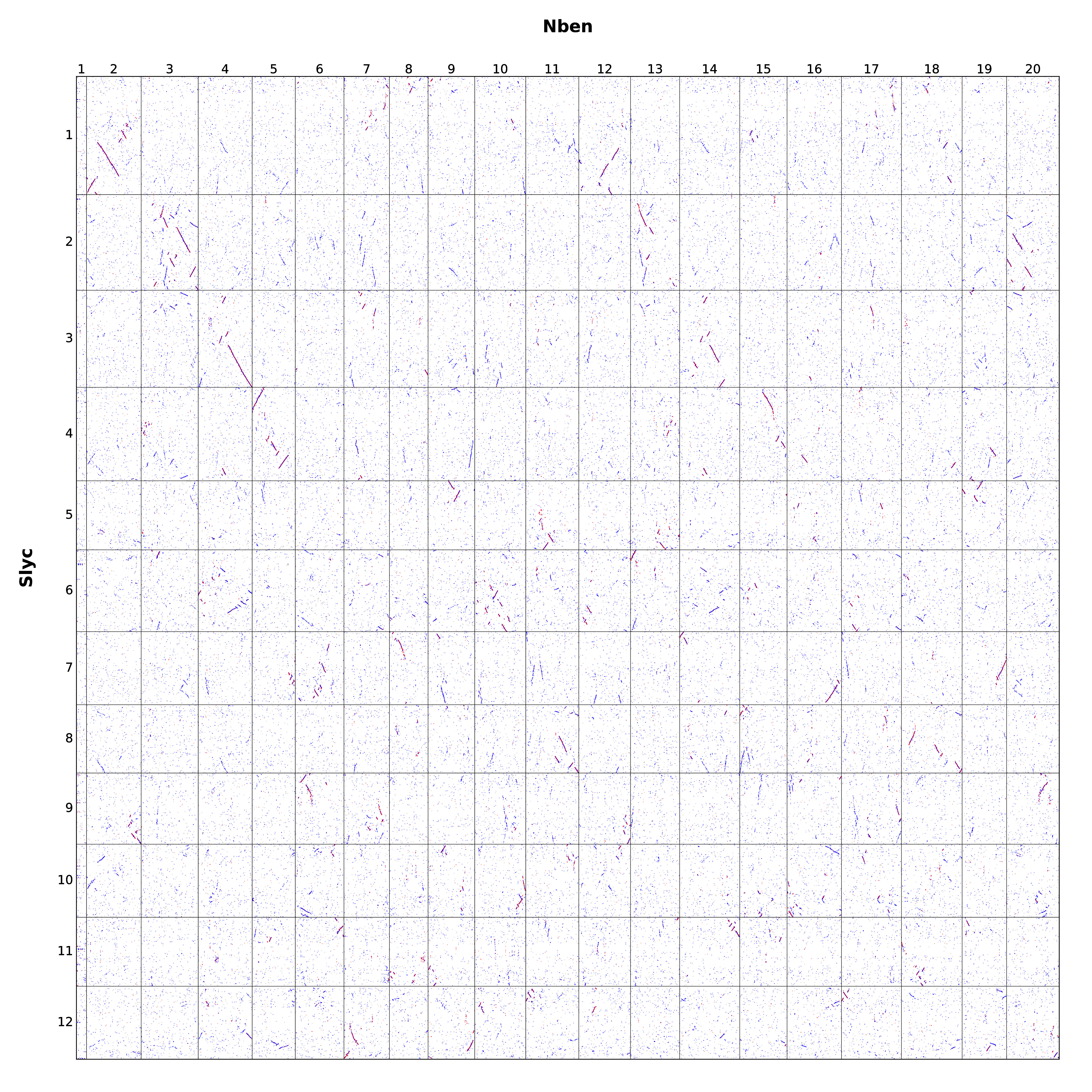

Supplement: giaf058_Supplemental_Files [file giaf058_supplemental_files.zip › Figure S9.jpg]
