# Supplementary material for: A telomere-to-telomere gapless genome reveals SlPRR1 control of circadian rhythm and photoperiodic flowering in tomato
Source: Gigascience. 2025 Jul 2;14:giaf058. doi: 10.1093/gigascience/giaf058 (PMC12218202; doi:10.1093/gigascience/giaf058)
Supplement: giaf058_GIGA-D-24-00568_original_submission [file giaf058_giga-d-24-00568_original_submission.pdf]

GigaScience

A telomere-to-telomere gapless genome reveals SlPRR1 control circadian rhythm and photoperiodic flowering in cultivated tomato

--Manuscript Draft--

**Manuscript Number:**	GIGA-D-24-00568
**Full Title:**	A telomere-to-telomere gapless genome reveals SlPRR1 control circadian rhythm and photoperiodic flowering in cultivated tomato
**Article Type:**	Research
**Funding Information:**	National Natural Science Foundation of China (32402552) — Dr. Hui Liu<br>Natural Science Research of Jiangsu Higher Education Institutions of China (BK20221009) — Dr. Hui Liu<br>Jiangsu Provincial Key Research and Development Program (BE2023350) — Dr. Jian-Ping Tao
**Abstract:**	The cultivated tomato (Solanum lycopersicum) is a major vegetable fruit with high economic values and plays role as an important model for the study of flowering time of day-neutral (ND) plants. Completely continuous and gap-less genome of cultivated tomato is needed to decipher the genetic research and breeding program. Here we reported a telomere-to-telomere (T2T) gap-free genome of S. lycopersicum cv. VF36 constructed by combined sequencing technologies. The 815.27 Mb T2T 'VF36' genome was presented with 600.23 Mb transposable elements (TEs) of the genome. Through comparative genomics and phylogenetic analysis, we revealed that structure variations (SVs) were predicted between 'VF36' and 'Heinz 1706' genome, and the recent WGD event was not occurred in 'VF36' tomato. Furthermore, a core circadian oscillator SlPRR1 was identified, which expressed peaked at nighttime with circadian rhythm. CRISPR/Cas9 knockdown SlPRR1 in tomato demonstrated that slprr1 mutant lines significantly early flowering under long-day (LD) condition. We presented the hypothetical model how SlPRR1 regulates flowering time and Chlorophyll biosynthesis to adjust to photoperiod. This T2T genomic resources will accelerate cultivated tomato genetic improvement and SlPRR1-related hypothetical model help to understand the photoperiodic response in cultivated tomatoes, uncovering a regulatory mechanism to manipulate flowering time in tomato.
**Corresponding Author:**	Ai-Sheng Xiong, Ph.D<br>Nanjing Agricultural University<br>nanjing, CHINA
**Corresponding Author Secondary Information:**	
**Corresponding Author's Institution:**	Nanjing Agricultural University
**Corresponding Author's Secondary Institution:**	
**First Author:**	Hui Liu
**First Author Secondary Information:**	
**Order of Authors:**	Hui Liu<br>Jia-Qi Zhang<br>Jian-Ping Tao<br>Chen Chen<br>Li-Yao Su<br>Jin-Song Xiong

Powered by Editorial Manager® and ProduXion Manager® from Aries Systems Corporation

	Ai-Sheng Xiong, Ph.D
**Order of Authors Secondary Information:**	
**Additional Information:**	
**Question**	**Response**
Are you submitting this manuscript to a special series or article collection?	No
**Experimental design and statistics**<br><br>Full details of the experimental design and statistical methods used should be given in the Methods section, as detailed in our Minimum Standards Reporting Checklist. Information essential to interpreting the data presented should be made available in the figure legends.<br><br>Have you included all the information requested in your manuscript?	Yes
**Resources**<br><br>A description of all resources used, including antibodies, cell lines, animals and software tools, with enough information to allow them to be uniquely identified, should be included in the Methods section. Authors are strongly encouraged to cite Research Resource Identifiers (RRIDs) for antibodies, model organisms and tools, where possible.<br><br>Have you included the information requested as detailed in our Minimum Standards Reporting Checklist?	Yes
**Availability of data and materials**<br><br>All datasets and code on which the conclusions of the paper rely must be either included in your submission or deposited in publicly available repositories (where available and ethically appropriate), referencing such data using a unique identifier in the references and in	Yes

Powered by Editorial Manager® and ProduXion Manager® from Aries Systems Corporation

the “Availability of Data and Materials” section of your manuscript.<br><br>Have you have met the above requirement as detailed in our [Minimum Standards Reporting Checklist]?	
GigaScience has policies and guidelines in place for the use of generative AI-writing tools such as ChatGPT. If you have used such writing tools to assist with writing the manuscript this must be declared and cited in the text. Authors should not list AI-writing tools and other AI-assisted technologies as an author or co-author and should acknowledge that they are fully responsible for text generated or refined by AI-writing tools.<p><br><br>A summary of use (particularly in the introduction or among methods) needs to be included at the end of the paper, and the outputs should also be included as a supplementary file hosted in GigaDB or other open repositories. Please read our guidelines for more information. <p><br><br>By submitting to GigaScience, you are aware of the journal's AI-writing tools policy, and if you have declared use of such tools below, you have acknowledged this where appropriate in your manuscript and have made a summary of use and outputs available. <p><br><b>AI-assisted writing tools have been used in the preparation of this manuscript?	No

Manuscript Click here to access/download;Manuscript;manuscript.docx

A telomere-to-telomere gapless genome reveals SlPRR1 control circadian rhythm and photoperiodic flowering in cultivated tomato

Hui Liu[1, #], Jia-Qi Zhang[1, #], Jian-Ping Tao[1], Chen Chen[1], Li-Yao Su[1], Jin-Song Xiong[1], Ai-Sheng Xiong[1, *]

[1] *State Key Laboratory of Crop Genetics & Germplasm Enhancement and Utilization, Ministry of Agriculture and Rural Affairs Key Laboratory of Biology and Germplasm Enhancement of Horticultural Crops in East China, College of Horticulture, Nanjing Agricultural University, Nanjing, Jiangsu 210095, China*

[#]These authors contributed equally to this work.

*Please address all correspondence to: Ai-Sheng Xiong (xiongaisheng@njau.edu.cn)

Dr. Ai-Sheng Xiong,

Professor,

State Key Laboratory of Crop Genetics & Germplasm Enhancement and Utilization,

College of Horticulture,

Nanjing Agricultural University,

1 Weigang, 210095, Nanjing, China

Email: xiongaisheng@njau.edu.cn

Abstract

The cultivated tomato (*Solanum lycopersicum*) is a major vegetable fruit with high economic values and plays role as an important model for the study of flowering time of day-neutral (ND) plants. Completely continuous and gap-less genome of cultivated tomato is needed to decipher the genetic research and breeding program. Here we reported a telomere-to-telomere (T2T) gap-free genome of *S. lycopersicum* cv. VF36 constructed by combined sequencing technologies. The 815.27 Mb T2T 'VF36' genome was presented with 600.23 Mb transposable elements (TEs) of the genome. Through comparative genomics and phylogenetic analysis, we revealed that structure variations (SVs) were predicted between 'VF36' and 'Heinz 1706' genome, and the recent WGD event was not occurred in 'VF36' tomato. Furthermore, a core circadian oscillator SlPRR1 was identified, which expressed peaked at nighttime with circadian rhythm. CRISPR/Cas9 knockdown *SlPRR1* in tomato demonstrated that *slprr1* mutant lines significantly early flowering under long-day (LD) condition. We presented the hypothetical model how SlPRR1 regulates flowering time and Chlorophyll biosynthesis to adjust to photoperiod. This T2T genomic resources will accelerate cultivated tomato genetic improvement and SlPRR1-related hypothetical model help to understand the photoperiodic response in cultivated tomatoes, uncovering a regulatory mechanism to manipulate flowering time in tomato.

Key Words: Cultivated tomato T2T genome; Photoperiod; Flowering time; Chlorophyll biosynthesis; SlPRR1

1 Introduction

Flowering is essential for plants which can transited from vegetative growth to reproductive growth, is usually regulated by daylength (or photoperiod). Long-day (LD) plants flower when the number of daylight hours exceeds a critical amount, whereas short-day (SD) plants require the opposite [1, 2]. In the LD plant *Arabidopsis thaliana*, the major photoperiodic flowering regulator *CONSTANS* (*CO*) expresses peaks before dusk in long days in response to light and the circadian clock [3]. CO has been sown to promote flowering by activating the expression of *FLOWERING LOCUS* (*FT*) and *SUPPRESSOR OF OVEREXPRESSION OF CO1* (*SOC1*) [4]. Photoperiod responses rely on crosstalk between light perception and the circadian clock, which together control the expression of the flowering hormone florigen [5]. The florigen gene *FT* is induced by CO protein in the light [6, 7]. As a day-neutral (ND) plant, flowering regardless of daylength was detected in tomato. Tomato (*Solanum lycopersicum*, 2n = 24) is an important cash vegetable cultivated throughout the world, which is a sympodial plant with a scorpioid cymose inflorescence [8]. As an important factor in tomato adaptability and genetic improvement, flowering time is regulated by various genes. *EARLY FLOWERING* (*ELF*) has been characterized to develop flowers much earlier than parental control [8]. Several classical genes involved in the control of flowering time in tomato, *SINGLE FLOWER TRUSS* (*SFT*), *JOINTLESS* (*J*), and *FALSIFLORA* (*FA*) exert the biological function to promote flowering, whereas *SELF PRUNING 5G* (*SP5G*) and *TERMINATING FLOWER* (*TMF*) have been shown to delay the flowering time in tomato [9]. The *SP5G*, one of *FT* paralogs, had been verified which contributed to the loss of day-length-sensitive flowering by reducing the LD response in tomato cultivars [5].

The metabolic daylength measurement system has been reported rely on the circadian clock-controlled balance in a photoperiodic manner [10]. The circadian clock is a molecular timing device that has been repeatedly characterized to regulate various physiological and developmental processes with and endogenous rhythm [11]. Flowering time is a phenological event which is regulated by the clock output pathways [5]. *EARLY FLOWERING 3* (*ELF3*) is identified as a circadian clock-associated gene and rhythmically inhibits the activity of the light input pathways around dusk by reducing clock sensitivity to light resetting at this phase, act as a transcriptional regulator to control the period of flowering time [12]. Lesions in *ELF3* cause early flowering, possibly by increased accumulation of transcripts of *CONSTANS* (*CO*), and arrhythmic expression of a morning-specific clock regulated gene *CHLOROPHYLL A/B BINDING 2* (*CAB2*) and an oscillator component *LATE ELONGATED HYPOCOTYL* (*LHY*) [13]. The *PSEUDO RESPONSE REGULATOR1* (*PRR1*) gene (also known as *TIMING OF CAB EXPRESSION1* (*TOC1*)) belongs to the PRR family, is the first member discovered to play a central role in the regulation of circadian rhythms [14, 15]. The clock runs faster in PRR1 mutant. misexpression of PRR1 can lead to hypocotyl growth and early flowering [16]. PRR1 represses the expression of *LHY* and *CIRCADIAN CLOCK ASSOCIATED1* (*CCA1*) by directly binding to their promoters in *Arabidopsis*. Comparing to *Arabidopsis*, cultivated tomato is less well studied in flowering regulatory pathways with circadian clock oscillator.

Chlorophyll (Chl) play central roles in harvesting light and transforming it into chemical energy through photosynthesis for the development of tomato fruit [17, 18]. Chl is the main photosynthetically active pigments. Chl metabolism in tomato leaves contribute to photosynthesis [19]. The Chl content of tomato modulates many metabolic processes and therefore affects the quality of fruit. However, it is unclear which

enzymes act to degrade Chl in the photosystems of tomato leaves in LD and SD condition. The Chl synthesis process consists with four main parts, including 5-aminoleculinic acid (ALA), protoporphyrin IX, chlorophyll a (Chl. a), and chlorophyll b (Chl. b) synthesis. Previous study reported *SlCHLH* and *SlCHLI* were important for Chl accumulation in tomato leaves [20]. Transcription factors such as GOLDEN2-LIKE 1 and 2 (GLK1& 2) have now been reported in many species, overexpression of *GLKs* increases chlorophyll and can lead to chloroplast development in tissues [21].

Cultivated tomato (*Solanum lycopersicum*) distributes worldwide with great economic significance in vegetable industry. Tomato is a good model for horticulture plants and especially for fleshy fruit biology [22]. Currently, although several tomato reference genomes have released [23, 24], a high-accuracy reference genome reported that 31 gaps still remained in SL5.0 [25]. A gapless reference genome of cultivated tomato is absence. Furthermore, centromeres are reported essential for maintaining the integrity of chromosomes during cell division and ensure the fidelity of their inheritance. Until now, centromeres in plants have remained largely underexplored [26]. A considerable fraction of the cultivated tomato genome and a number of gaps still remain to be resolved. Very recently, several horticulture species including carrot, grape, kiwifruit, lemon, and strawberry were sequenced with telomere-to-telomere (T2T) assemblies using PacBio high-fidelity (HiFi), Oxford Nanopore Technology (ONT) Ultra-long, and high-through chromosome conformation capture (Hi-C) technology [27-31]. However, no T2T gap-free genome has been reported in cultivated tomato.

Here, to improve the completeness of cultivated tomato reference genome, we assembled a telomere-to-telomere (T2T) gap-free genome sequence for ‘VF36’ using combining data from PacBio-HiFi, ONT ultra-long, and Hi-C technologies. ‘VF36’ tomato is an important variety [32]. It remains unclear whether the core circadian

oscillator SlPRR1 regulates the flowering time and Chlorophyll biosynthesis under different photoperiod. In this study, we demonstrated that knockdown *SlPRR1* in tomato caused early flowering under LD conditions and delayed flowering under SD conditions. Collectively, our study proposed a hypothetical model for the *SlPRR1* regulation of flowering time and Chlorophyll biosynthesis to adjust to photoperiod.

2 Results

2.1 A T2T gap-free tomato reference genome for 'VF36'

Based on *K*-mer analysis with 48.86 Gb Illumina reads, the genome size of 'VF36' tomato was estimated to be 774.22 Mb, with heterozygosity rate of 0.71% and duplication ratio of 38.84% (Figure S1-S2 and Table S1).

To develop a high-quality genome assembly for 'VF36' tomato, different sequencing platforms were employed. A total of 62 Gb PacBio HiFi reads and 169.51 Gb ONT Ultra-long reads were generated to pre-assemble the genome. The N_{50} length of HiFi reads was 17.74 kb, and the N_{50} length of ONT reads was 55.07 kb (Table S2). NextDenovo was applied to assembly the ONT data, forming 26 contigs with N_{50} size of 55.68 Mb. For the PacBio HiFi combined with ONT reads, we assembled a genome with contig N_{50} size of 68.44 Mb (Table S3), an increase of approximately 1.6-fold compared with the previous build SL5.0 [25] and 3.8- fold compared with 'Heinz 1706' build SLT1.0 [33]. Moreover, a total of 812.37 Mb Hi-C reads was anchored into 12 pseudochromosomes by assist the assembly correction (Figure 1A and Figure S3). After filling all remaining gaps, a gap-less reference genome of 'VF36' was generated, containing a total length of 815,269,421 bp (Table 1 and Table S4).

Using telomeric repeat (CCCTAAA at the 50 end or TTTAGGG at the 30 end) as

a sequence query, we identified 24 telomeric regions among 12 chromosomes. Plant centromeres were usually had high tandem repeats (TRs) density and low gene density. Based on the particular sequence structure, we identified nine denser regions of the tandem repeat clusters (TRCs) in each chromosome, which were continuous and occupied the majority (Figure S4). In total, 12 centromeric regions were estimated with lengths ranging from 0.48 to 3.63 Mb (Table S5).

Finally, a gap-free 'VF36' tomato genome consisted of 12 telomere-to-telomere (T2T) chromosomes with 12 centromeric regions was presented (Figure 1C). The BUSCO assessment indicated that 1592 of the core conserved plant genes (98.64% of 1614 BUSCOs) were found to be complete in 'VF36' tomato assembly. We assessed the base accuracy of the genome using *k*-mer quality estimation, the quality value (QV) ranged from 41.82 to 62.72 among each chromosome. The LTR assembly index (LAI) was used to evaluate genome assembly continuity, which was 12.71. These results indicated completeness, continuous, and accuracy of the genome assembly.

2.2 'VF36' tomato genome annotation

A total of 600,225,913 bp transposable elements (TEs) were identified, which accounted for 73.28% of the assembled 'VF36' tomato genome, long terminal repeat (LTR) elements being the major component with 429.64 Mb length. Gypsy-type LTRs (246.25 Mb) were much more abundant than Copia-type LTRs (68.01 Mb) (Figure 1B and Table S6). Moreover, we predicted protein-coding genes from the assembly, resulting in 'VF36' tomato annotation with 34,783 genes using a combination search method of *ab initio*, homology-based, and transcriptome-based. The average mRNA length was 5.12 kb and 4.8 exons per gene in 'VF36' genome (Table S7). The BUSCO assessment indicated that the completeness of the gene set of the annotated genome was

98.5% (Table S8).

In total, 33,540 genes (96.43%) could be functionally annotated through National Center for Biotechnology Information non-redundant (NR), Uniprot, InterPro, Pfam, gene ontology (GO), and Kyoto Encyclopedia of Genes and Genomes (KEGG) databases (Figure 1D and Table S9). The number of 413 micro RNAs (miRNAs), 1,049 transfer RNAs (tRNAs), 2,938 ribosomal RNAs (rRNAs), and 582 small nuclear RNAs (snRNAs) were found in ‘VF36’ tomato genome (Table S10).

2.3 Global comparison of ‘VF36’ and ‘Heinz 1706’ genomes

Comparing to ‘Heinz 1706’ tomato genome assembly, the ‘VF36’ genome assembly had longer length in size (Table 1). Especially, 12 T2T chromosomes with 12 centromeric regions were predicted on the ‘VF36’ genome, whereas none of the telomeres centromeric regions were identified in ‘Heinz 1706’ genome assemblies (SLT1.0 and SL5.0). The version of SLT1.0 genome had 210 gaps, a substantially higher completeness version of SL5.0 still had 31 gaps, whereas no gaps were left in ‘VF36’ genome. In addition, Furthermore, the BUSCO analysis showed an average of 98.6% single-copy genes completely assembled in ‘VF36’ genome, which slightly higher than in SLT1.0 (97.7%) and SL5.0 (96.2%). Taken together, ‘VF36’ genome assembly had higher completeness and accuracy than the ‘Heinz 1706’ assembly.

The collinearity analysis between ‘VF36’ and ‘Heinz 1706’ genome found 99.72% ‘Heinz 1706’ genome could mapped to ‘VF36’ genome (Figure 2A). We performed single nucleotide variation (SNP) and short insertion/deletion (InDel) analysis, which found 289,116 SNPs and 103,826 InDels through comparing analysis of ‘VF36’ and ‘Heinz 1706’ assembly. Among these, most SNPs (210,357) and InDels (74,011) were located at intergenic regions (Table S11). Abundant genetic variations, particular

structure variations (SVs) were detected between them. We predicted 1,807 SVs distributed throughout the genome, deletions (DELs) and insertions (INSs) accounted for 817 and 705 events, respectively. These DELs and INSs impacted 587 different functional genes, which contributed to the divergence of these two accessions. Furthermore, 60.55% SVs (1,076) were located at Intergenic regions, while only 4.76% SVs (86) were located at coding regions. Among these SVs, we observed two large (inversions) INVs (>400 kb) between the two genomes. INV299 with 2.78 Mb length (chr02: 37,961,912-40,734,631) was predicted between 'VF36' and 'Heinz 1706', which was annotated pectate lyase (Table S12). The GO enrichment analysis on the genes in SV regions indicated that the enriched terms were immune response, DNA integration, and metal ion binding (Figure 2D).

2.4 Phylogenetic analysis

Identification of homologous genes was critical for 'VF36' tomato evolutionary analysis. We predicted homologous genes among 13 genomes including 'VF36', 'Heinz 1706', *Solanum pimpinellifolium*, *Solanum pennellii*, *Solanum tuberosum*, *Solanum melongena*, *Capsicum annuum*, *Nicotiana benthamiana*, *Petunia axillaris*, *Cuscuta campestris*, *Daucus carota*, *Vitis vinifera*, and outgroup *Oryza sativa* (Table S13). As expected, 'VF36' clustered together with 'Heinz 1706' (Figure 2B). A total of 527,829 homologous genes were identified and classified into 86,939 gene families. Among these genes, 195,190 genes were distributed into 7,593 gene families which shared with 13 genomes (Table S14). According to characterized 52 single-copy gene families and low-copy gene families, we constructed a phylogenetic tree with divergence time. Our findings inferred that 'VF36' divergence from 'Heinz 1706' tomato to be 2.1 million years ago (Mya). *C. annuum* was the *Solanum* species sister

group, the divergence time of *C. annuum*- *Solanum* lineage approximately 33.3 Mya (Figure 2B).

The gene family expansion and contraction were examined using CAFE. Expanded and unique gene families in 'VF36' tomato were recognized with 456/1,786. These expanded genes were involved in responding to auxin, oxidoreductase activity, photosystem Il, and protein serine/threonine/tyrosine kinase activity through GO analysis (Figure S5). The unique genes were enriched in transferase activity, protein kinase activity, and methylation (Figure S6).

The distribution of synonymous substitutions per synonymous site (K_S) between collinear homologous genes was detected. We observed a K_S peak of approximately 0.65 in 'VF36' tomato, which was presented in the other Solanaceae species *S. tuberosum* and *N. benthamiana*, corresponding to the time of shared family-specific Solanaceae-α hexaploidy event [34] (Table S15). While a substantial recent K_S peak at approximately 0.15 was detected present in *N. benthamiana*, in agreement with previous studies [35, 36] (Figure 3). Furthermore, inter-genomic gene collinearity between *V. vinifera* and 'VF36' tomato was investigated with 6:1 syntenic depth ratio (Figure S7). A ratio of 2:1 between *N. benthamiana* - 'VF36' tomato and *N. benthamiana* - *S. tuberosum* was characterized (Figure S8). The dot plot of syntenic analysis showed each fragment in 'VF36' tomato could be identified with the two most related synteny fragments in *N. benthamiana*. Taken together, the results indicated that the recent WGD event was not occurred in 'VF36' tomato.

2.5 A core circadian oscillator SlPRR1 repressed flowering in tomato

Flowering time is a vital trait for the reproductive success of tomato. Photoperiod (daylength) has been well known to regulated plant growth and flowering. The PRR1

was identified the core circadian oscillator, which was essential for plant growth and development. Previous report showed the expression of SlPRR1 had a robust light-dependent circadian rhythm with night-peaking [37]. The expression profiles of *SlPRR1* were performed by RT-qPCR analysis which showed it highly expressed in roots and fruits (Figure 4A). We observed the expression of *SlPRR1* peaked at nighttime and had circadian rhythm (Figure 4B).

To determine the biological role of *SlPRR1* in flowering time, genetic evidence was provided by CRISPR/Cas9 gene-editing system. We designed two single-guide RNA (sgRNA) to target the third exon of *SlPRR1* (Figure 5A). Four homozygous mutant lines designated *slprr1-35*, *slprr1-5*, *slprr1-6*, and *slprr1-10* were used for further analysis (Figure 5B). To better investigate the role of *SlPRR1* in daylength responses, we measured flowering time of control plants (WT) and *slprr1* mutant lines under LD and SD conditions. Compared with WT tomato, *slprr1* mutant lines significantly early flowering under LD condition. While *slprr1* mutant lines delayed flowering compared to WT plants under SD condition (Figure 5C-E).

The *slprr1* mutation caused early flowering under LD and effected gene expression of *CO/COL*. We identified 13 *CO/COL* genes in tomato, the expression of these genes increased in *slprr1* mutations, except *SlCO1*, *SlCOL10b*, and *SlCOL16b* (Figure 8K). The COs were considered as mediates between the circadian clock and the control of flowering [38]. CO had been shown to promote flowering by activating the expression of *FT* and *SOC1*. We surveyed expression of a group of genes which had been implicated in flowering time control in tomato, including *FTL1, J, SFT, SP5G, BOP*, *TMF*, *SOC1*, and *FA* (Figure 8A-J). Among them, tomato *SP5G* gene was the *FT* paralog and significantly higher expressed in LD than in SD. However, another *FT* paralog *FTL1* gene transcription obviously decreased in LD. The result indicated that

SP5G acted to regulate flowering under LD, whereas FTL1 responded specifically to SD. Furthermore, the expression of *SOC1* gene was down-regulated significantly in the *slprr1* mutants both under LD and SD. The expression of *J* and *FUL2* genes had significantly changed between *slprr1* mutant and WT plants. These data proposed a model for the regulation of flowering time under LD or SD condition and provided evidence that the core circadian clock gene *SlPRR1* regulated flowering time in tomato (Figure 9).

2.6 Knockout of *SlPRR1* affected sugar accumulation in tomato fruit

The sugar contents of red ripening fruits were analyzed in WT and gene-edited fruits under LD and SD conditions, which played a decisive role for tomato quality [24]. The *slprr1* mutant fruits showed significantly fewer in fructose and glucose content under both LD and SD conditions. The sucrose contents were detected significantly higher in *slprr1* mutant fruits than in WT fruits under both LD and SD conditions, although much lower content of sucrose compared to fructose and glucose in tomato fruits (Figure 5F). The result indicated that knockout of *SlPRR1* promoted flowering in LD condition, effected on the fruit flavor in tomato.

2.7 Proposed Chlorophyll biosynthesis pathway in tomato leaves

To explore the Chlorophyll biosynthesis pathway in tomato leaves, we measured Chlorophyll contents in WT and gene-edited plants under LD or SD condition. Compared with both WT and gene-edited plants in SD, the tomato leaves exhibited significantly higher Chlorophyll contents in LD. The *slprr1* mutants showed slight lower Chlorophyll contents than in WT plants under both LD and SD conditions.

The simple Chlorophyll biosynthesis pathway had been elucidated and the

enzymes genes were surveyed (Figure 6A and Table S). Among these genes, the *SlGluTR_1*, *SlPPO*, *SlPOR1*, and *SlPOR2* was significantly higher expressed in WT plants under LD condition based on transcriptome analysis. To infer the transcription factors (TFs) modulate the transcription of genes related to Chlorophyll metabolism in tomato, we analyzed the expression of *SlH2A* and *SlNDF5* up-regulated and *SlGLK2*, *SlLHCB*, and *SlpsaH* down-regulated under LD (Figure 7A-K). It inferred that *SlH2A* and *SlNDF5* positively in regulatory activity of candidate enzymes, whereas *SlGLK2*, *SlLHCB*, and *SlpsaH* negatively regulated SlGluTR_1, SlPPO, SlPOR1, and SlPOR2 under LD (Figure 9).

3 Discussion

A high-quality reference genome is indispensable for uncovering traits and facilitating genetic enhancement. We have publicly released a T2T gap-free genome of cultivated tomato ‘VF36’, which included 815.27 Mb sequence and 34,783 protein-coding genes in this study. This complete genome sequence of cultivated tomato is the largest complete genome sequence reported thus far, although several tomato reference genomes have released [23-25, 39]. The combination of ONT ultra-long, PacBio-HiFi, and Hi-C sequencing technology, has overcome the assembly issues including remained 31 gaps in SL5.0 version and centromeres. In our version of cultivated tomato ‘VF36’, we have successfully corrected many misassembles and filled all chromosomal gaps. The comparison of SVs presents in ‘VF36’ and ‘Heinz 1706’ provides several regions associated with immune response, DNA integration, and metal ion binding.

Modern cultivated tomato is derived from the wild relative, *Solanum pimpinellifolium*. The wild tomatoes are recognized as SD plants, while most cultivated

accessions reduce photoperiodic sensitivity and are considered day-neutral plants [9]. Selection for flowering time is a major goal of tomato breeding efforts, which spread cultivars worldwide from their origin. In this study, a core circadian oscillator SlPRR1 has circadian rhythm and the expression been verified peaked at nighttime, which in agreement with previous studies [40]. We noted that SlPRR1 highly expressed in roots during vegetative growth, when tissues were in a dark environment.

The clock runs faster in *slprr1* mutants under LD condition, while the *slprr1*1 mutants delay flowering under SD condition. The result indicates that SlPRR1 functions in cultivated tomatoes, which regulates flowering time. CO is a circadian clock-regulated gene encoding a transcription factor required for flowering [41], and is modulated by day length. The number of 13 *CO*/*COL* genes are identified in cultivated tomato. Among these, most genes differential expressed between LD and SD conditions. Previous study suggested *SlCOL*, *SlCOL4a*, and *SlCOL4b* might function as positive regulators of tomato flowering [42]. *CO* and *FT* are two central integrators of the photoperiod pathway controlling flowering time, FT members are the final output of the photoperiodic response downstream of CO [43]. We observed several florigen genes involved in the control of flowering time identified before. *SP5G* gene highly expressed in LD conditions, whereas hardly expressed in SD conditions. *SP5G* as the *FT* paralog, is a major locus influencing daylength adaptation in tomato, has been verified in tomato cultivars contributes to the loss of day-length-sensitive flowering by reducing the LD response. While another *FT* paralog, FTL1 does not control LD flowering in tomato and responds specifically to SDs [5]. Consistent with our result in this study that *FTL1* gene was specifically expressed under SDs. So, we proposed the model for the regulation of flowering time by knockout SlPRR1 in cultivated tomato under LD and SD conditions.

Flowering is an important developmental stage and also a clear sign of plants transition from vegetative stage to the reproductive stage. During tomato growth cycle, the chlorophyll contents of leaves change dynamically with chlorophyll accumulation and degradation. Chlorophyll, as well-known the most abundant pigment on earth, is a key component of photosynthesis required for the absorption of sunlight [44]. Photoperiod regulates plant germination, growth, and flowering, while affects chlorophyll biosynthesis. In addition to photoreceptors, chloroplasts act as plant light sensors in response to different photoperiods by changing their ultrastructure [45]. Few studies have been reported that the regulatory mechanisms of Chlorophyll biosynthesis in cultivated tomato leaves under different photoperiods. Under LD conditions, the chloroplasts of growing plants exhibit smaller grana stacks, and the Chlorophyll contents increase significantly. In this study, the tomato leaves exhibited higher Chlorophyll contents in LD than in SD conditions. Chlorophyll biosynthesis can be divided into four parts, including formation of 5-aminolevulinic acid, biosynthesis of protoporphyrin IX from eight molecules of 5-aminolevulinic acid, and biosynthesis of chlorophyll *a* and *b* in the magnesium branch [46]. Moreover, Chlorophyll biosynthesis was influenced by several genes, like *GLKs*, which were reported played a vital role in regulating Chlorophyll accumulation and chloroplast development in tomato fruit [21]. The BEL2 might directly bind to the *GLK2* promoter to repress its transcription in tomato [47]. In this study, *GLK2* gene highly expressed in SD conditions than in LD conditions. While *BEL2* gene highly expressed in in *slprr1* mutants under LD condition, which showed different expression patter to that of *GLK2* in tomato. The process of Chlorophyll synthesis and degradation is dynamic and complex, which influenced by environment, such as photoperiod and regulated by multiple enzyme and regulatory genes.

4 Conclusions

In summary, we presented T2T gap-free genome of cultivated tomato *var*. VF36 using combining data from PacBio-HiFi, ONT ultra-long, and Hi-C technologies. A core circadian oscillator SlPRR1 was identified and verified which *slprr1* mutant lines significantly early flowering under LD and delayed flowering under SD conditions. We presented the hypothetical model how SlPRR1 regulates flowering time and Chlorophyll biosynthesis to adjust to photoperiod. These findings provided novel approaches for essential regulatory mechanism of flowering time and made facilitate the manipulation and improvement of yield in tomato crops.

5 Experimental Section

Plant materials, growth conditions, and photoperiod treatment

Tomato (*Solanum lycopersicum*) plants cultivars 'VF36' and 'Micro-Tom' were grown in soil in growth chambers at Nanjing Agricultural University. For flowering time assessment, the tomato seeds were sown in growth chambers under a light intensity of 20000 lx (360 μmol m^{-2} s^{-1}) at 25 °C and 70% relative humidity with different photoperiods, LD (16L:8D), SD (8L:16D), and ND (12L:12D). The flowering time was evaluated as the number of days to reach the first observable flower opening, at least three individual plants were used for assessment.

DNA extraction and sequencing

Leaf samples of cultivated tomato 'VF36' inbred line were collected for genome

sequencing. A modified cetyltrimethyl ammonium bromide (CTAB) method was performed for DNA extraction.

The ONT ultra-long library was obtained from Nanopore sequencing platform (Nanopore, Oxford, UK) and size-selected (>30 kb) using Filtlong (v.2.4) software. Then, the pass reads were filtered joint sequence with mean read quality scores more than 90% by Porechop (v.2.4). In addition, a SMART cell sequencing library containing about 15-20 kb cut fragment was constructed and sequenced using PacBio according to standard protocol (PacBio, CA, USA) [48].

For Illumina short-read sequencing, Nextera DNA Flex Library Prep Kit (Illumina, CA, USA) Sequencing libraries were constructed using a library construction kit (Illumina, CA, USA) and sequenced on Illumina HiSeq 2000 platform. The raw reads were filtered and polished using fastp (v.21.0). Simultaneously, a Hi-C library was established on Illumina NovaSeq 6000 platform (Illumina, CA, USA). A total of 662,677,798 bp clean data was obtained. Above sequencing was performed in the Wuhan Benagen Technology Co., Ltd (Wuhan, China) [49].

Genome initial assembly and assessment

The genome size was estimated based on *k*-mer distribution analysis by jellyfish (v.2.2.10), the heterozygosity rate of the genome was determined using GCE (v.1.0) [50]. After removing the low-quality sequences from ONT ultra-long sequencing data, NextDenovo (v.2.5) was used to initial assembly with parameters of read_cutoff =1k, blocksize = 1g, nextgraph_options = -a 1. The assembly was corrected with the ONT reads using Racon (v.1.4.11) with two rounds. We further improved the correction of the assembly by Pilon (v.1.23) with second-generation sequencing data. In addition, a combined assembly strategy based on PacBio HiFi reads was used to obtain high-

accuracy assembly. We performed Hifiasm (v.0.16.1-r375) software to assembly the genome by PacBio HiFi reads only and PacBio HiFi reads combined with ONT ultra-long reads [51]. Genome initial assembly completeness was assessed using the embryophyte_odb10 database of 1614 single-copy orthologues using BUSCO (v.4.1.3) [52].

A total of 98.70 Gb Hi-C sequencing data was used to assist assembly the genome through clustering, ordering, orienting, and eliminating redundancy of the contigs by ALLHiC (v.0.9.8), 3D-DNA (v.180419), and juicer (v.1.6) software. Then, 100 N were used to fill the gaps and obtain the final chromosome-level genome sequence. Finally, the accuracy of Hi-C based chromosomal assembly was assessed using HiCExplorer (v.3.6) chromatin contact matrix [53].

The missed telomers were further filled using ONT ultra-long reads by Winnowmap (v.1.11), medaka_consensu (v.1.2.1), and nucmer (v.3.1) as descripted by Wang [27]. To further improve the scaffold building and fill the gaps, winnowmap (v.1.11) was used with corrected ONT ultra-long reads and HiFi reads. Genome assembly continuum was assessed by the location and number of gaps on the genome. The completeness of gene regions was evaluated using BUSCO descripted above. In addition, the quality-value (QV) of the genome assembly was estimated based on *K*-mer database from Illumina short reads.

Genome annotation

Repetitive sequences were searched by homology-based and *de novo* approaches. RepeatModeler (v.2.0.4) and LTR_FINDER was employed to build *de novo* transposable element (TE) library [54]. RepeatMasker (v.4.1.5) was applied to identified TEs from Repbase TE library added with *de novo* TE library [55]. Finally,

the non-redundant combined TE sets were obtained.

Annotation of gene structure was conducted by ab initio, homology-based, and transcriptome-based prediction methods. Homologies from five species (*Solanum chmielewskii*, *Solanum galapagense*, *Solanum lycopersicum* var 'Heinz1706', *Solanum pimpinellifolium*, and *Arabidopsis thaliana*) were collected as protein evidence for predicted gene sets using Exonerate (v.2.4). TransDecoder (v.5.7) pipeline was used to assembly RNA-seq reads into transcripts. Ab initio gene prediction was performed with Augustus (v.3.5.0) and Glimmerhmm (v.3.0.4) [56]. The all predictions were generated to a comprehensive protein-coding gene set by Maker (v.3.01.03) [57]. Furthermore, the gene function annotation was carried out through homology searching against the public databases including NCBI non-redundant (NR), Uniprot, InterPro, Pfam, GO, and KEGG databases.

Non-coding RNAs (ncRNAs) including transfer RNAs (tRNAs), ribosome RNAs (rRNAs), microRNAs (miRNAs), and small nuclear RNAs (snRNAs) were predicted. The tRNAscan-SE (v.2.0.12) was performed to identify tRNAs with default parameters, RNAmmer (v.1.2) was used to search rRNAs, and INFERNAL (v.1.1.4) was applied to find miRNAs or snRNAs based on Rfam database [58].

Identification of centromeres and telomeric sequences

The regions of centromeres were consisted with tandem repeats (TRs), centromeric retrotransposon, and low-copy sequences. For telomere identification, the plant telomere sequences 5'-CCCTAAA-3' were identified. Furthermore, TRF software was used to search TR sequences, the denser regions of the tandem repeat clusters (TRCs) tended to be the centromere regions.

Synteny analysis and identification of SNPs, InDels, and SVs

MUMmer was applied to perform genomic collinearity analysis between 'VF36' and 'Heinz 1706' genomes [59]. Then, single nucleotide variations (SNPs) and short insertions/deletions (InDels), structure variations (SVs) were implemented by SyRI [60]. Annotations were obtained through the ANNOVAR software toolkit.

SVs were classified into five types: inversion, translocation, duplication, deletion, and insertion. Based on their overlapping regions in the genome, we calculated the number of SVs in coding regions, introns, 2K bp upstream, 2K bp downstream, and intergenic regions. Compared to the 'VF36' genome, the identified genes affected by SVs were subjected to GO and KEGG enrichment analysis.

Phylogenetic, gene family expansion/contraction analysis

Inferring the evolution history of 'VF36' tomato, we identified homologs and single-copy orthologous genes using OrthoFinder software in *S. lycopersicum* var 'Heinz1706', *Solanum pimpinellifolium*, *Solanum pennellii*, *Solanum tuberosum*, *Solanum melongena*, *Capsicum annuum*, *Nicotiana benthamiana*, *Petunia axillaris*, *Cuscuta campestris*, *Daucus carota*, *Vitis vinifera*, and outgroup *Oryza sativa*. The single-copy genes were aligned by MUSCLE (v3.8.31). Phylogenetic tree was constructed using maximum likelihood (ML) method in RAxML software with the JTT models for amino acid data [61]. Then, the MCMCTREE in PAML was performed to estimate the divergence time [48].

The gene family expansion and contraction were analyzed by computational analysis of gene family evolution (CAFE v.3.1) with default parameters in 'VF36' tomato compared with that in other 12 species [62].

Synteny and whole-genome duplication (WGD) analysis

Syntenic gene pairs between 'VF36' tomato, *S. lycopersicum* var 'Heinz1706', *S. pimpinellifolium*, *S. pennellii*, *S. tuberosum*, *S. melongena*, *C. annuum*, *N. benthamiana*, *P. axillaris*, *C. campestris*, *D. carota*, *V. vinifera*, and *O. sativa* were identified using JCVI (v.0.9.13) [63].

Whole-genome duplications (WGDs or polyploidy) were prevalent and estimated by synonymous substitutions per synonymous site (K_S) values. K_S estimates for pairwise comparisons (one-to-one orthologs between species) were obtained by PAML package implemented in the yn00 program.

CRISPR plasmid construction and stable tomato transformation

For the CRISPR/Cas9 construct, two specific sgRNAs at the exon of tomato *SlPRR1* gene were designed using CRISPR-GE online tool (http://skl.scau.edu.cn/targetdesign). Two sgRNAs expression cassettes, combined with two target sites, were driven by *AtU6*, and assembled into the pHSbdcas9i vector [64]. The confirmed constructs were transformed into *Agrobacterium tumefaciens* strain GV3101. The constructs were introduced into tomato cv Micro-Tom by *A. tumefaciens*-mediated transformation. All the primers were listed in Table S16. Homozygous T_1 transgenic plants were used for phenotypic characterization.

Chlorophyll contents, sugar extraction and measurement

Chlorophyll content was measured by Plant Nutrition Tester (SPAD 502, Beijing Zhongke Weihe Technology Development Co., Ltd, Beijing, China). Soluble sugars were determined by High-Performance Liquid Chromatography (HPLC) system (Waters Corp., MA, USA) equipped with an Acquity UPLC BEH amide column and an

Evaporative Light Scattering detector was used. Samples were collected from red ripe tomato fruits, then frozen in liquid nitrogen immediately and stored at -80 °C freezer. The soluble sugars were extracted and analyzed as described before. Briefly, freeze-dried samples were added to 5 ml of distilled water and homogenized for 1 min, then extracted in a water bath at 80 °C for 30 min. The supernatant was collected by centrifugation, filtered through 0.45 μm membrane to determine the soluble sugar content in the filtrate.

Statistical analysis

Significance tests were carried out by Prism 9 (GraphPad, CA, USA) based on Student's *t*-test at P <0.01 or $P < 0.05$. The data were presented as the means ± SDs (standard deviations).

Declarations

Ethics approval and consent to participate

Not applicable.

Availability of data and materials

The raw data of genome and transcriptome sequencing for this project were deposited in the CNGB Nucleotide Sequence Archive (https://db.cngb.org/cnsa) and are accessible with the accession ID CNP0006298.

Competing interests

The authors declare that they have no competing interests.

Funding

This research was supported by National Natural Science Foundation of China (32402552), Natural Science Foundation of Jiangsu (BK20221009), and Key Research and Development Program of Jiangsu (BE2023350).

Authors' contributions

A.-S.X. and H.L. designed and supervised the study. H.L. wrote the manuscript. H.L. and J.-Q.Z. performed the experiments with assistance from J.-P.T., C.C., and L.-Y.S. J.-S.X. and A.-S.X. contributed substantially to revisions. All authors commented on the manuscript.

Acknowledgements

Not applicable.

Reference

1. Andrés F and Coupland G. The genetic basis of flowering responses to seasonal cues. Nature Reviews Genetics. 2012;13 9:627-39.
2. Huang H and Nusinow DA. Into the Evening: Complex Interactions in the Arabidopsis Circadian Clock. Trends in Genetics. 2016;32 10:674-86. doi:10.1016/j.tig.2016.08.002.
3. Song YH, Shim JS, Kinmonth-Schultz HA and Imaizumi T. Photoperiodic Flowering: Time Measurement Mechanisms in Leaves. Annual Review of Plant Biology. 2015;66 1:441-64.
4. Samach A, Onouchi H, Gold SE, Ditta GS, Schwarz-Sommer Z, Yanofsky MF, et al. Distinct Roles of CONSTANS Target Genes in Reproductive Development of Arabidopsis. Science. 2000;288 5471:1613-6. doi:doi:10.1126/science.288.5471.1613.
5. Soyk S, Muller NA, Park SJ, Schmalenbach I, Jiang K, Hayama R, et al. Variation in the flowering gene SELF PRUNING 5G promotes day-neutrality and early yield in tomato. Nature Genetics. 2017;49 1:162-8. doi:10.1038/ng.3733.
6. Valverde F, Mouradov A, Soppe W, Ravenscroft D and Coupland G. Photoreceptor Regulation of CONSTANS Protein in Photoperiodic Flowering. Science. 2004;303

5660:1003-6.
7. The flowering time regulator CONSTANS is recruited to the FLOWERING LOCUS T promoter via a unique cis-element. New Phytologist. 2010;187 1:57-66.
8. Zhang D, Ai G, Ji K, Huang R, Chen C, Yang Z, et al. EARLY FLOWERING is a dominant gain-of-function allele of FANTASTIC FOUR 1/2c that promotes early flowering in tomato. Plant Biotechnology Journal. 2024;22 3:698-711. doi:10.1111/pbi.14217.
9. Zhang S, Jiao Z, Liu L, Wang K, Zhong D, Li S, et al. Enhancer-Promoter Interaction of SELF PRUNING 5G Shapes Photoperiod Adaptation. Plant Physiology. 2018;178 4:1631-42. doi:10.1104/pp.18.01137.
10. Wang Q, Liu W, Leung CC, Tarte DA and Gendron JM. Plants distinguish different photoperiods to independently control seasonal flowering and growth. Science. 2024;383 6683:eadg9196. doi:10.1126/science.adg9196.
11. Gil KE and Park CM. Thermal adaptation and plasticity of the plant circadian clock. New Phytologist. 2019;221 3:1215-29. doi:10.1111/nph.15518.
12. Liu XL, Covington MF, Fankhauser C, Chory J and Wagner DR. ELF3 Encodes a Circadian Clock–Regulated Nuclear Protein That Functions in an Arabidopsis PHYB Signal Transduction Pathway. The Plant Cell. 2001;13:1293-304.
13. Yu JW, Rubio V, Lee NY, Bai S, Lee SY, Kim SS, et al. COP1 and ELF3 control circadian function and photoperiodic flowering by regulating GI stability. Molecular Cell. 2008;32 5:617-30. doi:10.1016/j.molcel.2008.09.026.
14. Alabadi D, Oyama T, Yanovsky MJ, Harmon FG and Más PK, S.A. Reciprocal Regulation Between TOC1 and LHY/CCA1 Within the Arabidopsis Circadian Clock. Science. 2001;293 5531:880-3.
15. Du SX, Wang LL, Yu WP, Xu SX, Chen L and Huang W. Appropriate induction of TOC1 ensures optimal MYB44 expression in ABA signaling and stress response in Arabidopsis. Plant, Cell & Environment. 2024;14922 14922:1-17. doi:10.1111/pce.14922.
16. Fung-Uceda J, Lee K, Seo PJ, Polyn S, De Veylder L and Mas P. The Circadian Clock Sets the Time of DNA Replication Licensing to Regulate Growth in Arabidopsis. Developmental Cell. 2018;45 1:101-13. doi:10.1016/j.devcel.2018.02.022.
17. Pan X, Ma J, Su X, Cao P, Chang W, Liu Z, et al. Structure of the maize photosystem I supercomplex with light-harvesting complexes I and II. Science. 2018;360 6393:1109-13. doi:doi:10.1126/science.aat1156.
18. Tian Y-n, Zhong R-h, Wei J-b, Luo H-h, Eyal Y, Jin H-l, et al. Arabidopsis CHLOROPHYLLASE 1 protects young leaves from long-term photodamage by facilitating FtsH-mediated D1 degradation in photosystem II repair. Molecular Plant. 2021;14 7:1149-67. doi:https://doi.org/10.1016/j.molp.2021.04.006.
19. Chen Y, Cai X, Tang B, Xie Q, Chen G, Chen X, et al. SlERF.J2 reduces chlorophyll accumulation and inhibits chloroplast biogenesis and development in tomato leaves. Plant Science. 2023;328:111578. doi:10.1016/j.plantsci.2022.111578.
20. Yan HX, Fu DQ, Zhu BZ, Liu HP, Shen XY and Luo YB. Sprout vacuum-infiltration: a simple and efficient agroinoculation method for virus-induced gene silencing in diverse solanaceous species. Plant Cell Reports. 2012;31 9:1713-22.
21. Nguyen CV, Vrebalov JT, Gapper NE, Zheng Y, Zhong S, Fei Z, et al. Tomato GOLDEN2-LIKE Transcription Factors Reveal Molecular Gradients That Function during Fruit

Development and Ripening. Plant Cell. 2014;26 2:585-601.

22. Meissner R, Jacobson Y, Melamed S, Levyatuv S and Levy A. A new model system for tomato genetics. The Plant Journal. 2010;12 6:1465-72.

23. Lin T, Zhu G, Zhang J, Xu X, Yu Q, Zheng Z, et al. Genomic analyses provide insights into the history of tomato breeding. Nature Genetics. 2014;46 11:1220-6. doi:10.1038/ng.3117.

24. Gao L, Gonda I, Sun H, Ma Q, Bao K, Tieman DM, et al. The tomato pan-genome uncovers new genes and a rare allele regulating fruit flavor. Nature Genetics. 2019;51 6:1044-51. doi:10.1038/s41588-019-0410-2.

25. Zhou Y, Zhang Z, Bao Z, Li H, Lyu Y, Zan Y, et al. Graph pangenome captures missing heritability and empowers tomato breeding. Nature. 2022;606 7914:527-34. doi:10.1038/s41586-022-04808-9.

26. Perumal S, Koh CS, Jin L, Buchwaldt M, Higgins EE, Zheng C, et al. A high-contiguity Brassica nigra genome localizes active centromeres and defines the ancestral Brassica genome. Nature Plants. 2020;6 8:929-41. doi:10.1038/s41477-020-0735-y.

27. Wang Y-H, Liu P-Z, Liu H, Zhang R-R, Liang Y, Xu Z-S, et al. Telomere-to-telomere carrot (Daucus carota) genome assembly reveals carotenoid characteristics. Horticulture Research. 2023;10 7:uhad103. doi:10.1093/hr/uhad103.

28. Zhang K, Du M, Zhang H, Zhang X, Cao S, Wang X, et al. The haplotype-resolved T2T genome of teinturier cultivar Yan73 reveals the genetic basis of anthocyanin biosynthesis in grapes. Horticulture Research. 2023;10 11:uhad205. doi:10.1093/hr/uhad205.

29. Yue J, Chen Q, Wang Y, Zhang L, Ye C, Wang X, et al. Telomere-to-telomere and gap-free reference genome assembly of the kiwifruit Actinidia chinensis. Horticulture Research. 2022;10 2:uhac264. doi:10.1093/hr/uhac264.

30. Bao Y, Zeng Z, Yao W, Chen X, Jiang M, Sehrish A, et al. A gap-free and haplotype-resolved lemon genome provides insights into flavor synthesis and huanglongbing (HLB) tolerance. Horticulture Research. 2023;10 4:uhad020. doi:10.1093/hr/uhad020.

31. Zhou Y, Xiong J, Shu Z, Dong C, Gu T, Sun P, et al. The telomere-to-telomere genome of Fragaria vesca reveals the genomic evolution of Fragaria and the origin of cultivated octoploid strawberry. Horticulture Research. 2023;10 4:uhad027. doi:10.1093/hr/uhad027.

32. Canady MA, Meglic V and Chetelat RT. A library of Solanum lycopersicoides introgression lines in cultivated tomato. Genome. 2005;48 4:685-97. doi:10.1139/g05-032.

33. Su X, Wang B, Geng X, Du Y, Yang Q, Liang B, et al. A high-continuity and annotated tomato reference genome. BMC Genomics. 2021;22 898:1-12. doi:10.1186/s12864-021-08212-x.

34. Bombarely A, Moser M, Amrad A, Bao M, Bapaume L, Barry CS, et al. Insight into the evolution of the Solanaceae from the parental genomes of Petunia hybrida. Nat Plants. 2016;2 6:16074. doi:10.1038/nplants.2016.74.

35. Yang J, Wu Y, Zhang P, Ma J, Yao YJ, Ma YL, et al. Multiple independent losses of the biosynthetic pathway for two tropane alkaloids in the Solanaceae family. Nat Commun. 2023;14 1:8457. doi:10.1038/s41467-023-44246-3.

36. Xu S, Brockmller T, Navarro-Quezada A, Kuhl H and Baldwin IT. Wild tobacco genomes reveal the evolution of nicotine biosynthesis. Proceedings of the National Academy of Sciences of the United States of America. 2017; 114:6133-8.

37. Huang T, Liu H, Tao J-P, Zhang J-Q, Zhao T-M, Hou X-L, et al. Low light intensity

elongates period and defers peak time of photosynthesis: a computational approach to circadian-clock-controlled photosynthesis in tomato. Horticulture Research. 2023;10 6:uhad077. doi:10.1093/hr/uhad077.

38. Suárez-López P, Wheatley K, Robson F, Onouchi H, Valverde F and Coupland G. CONSTANS mediates between the circadian clock and the control of flowering in Arabidopsis. Nature. 2001;410 6832:1116-20. doi:10.1038/35074138.

39. van Rengs WMJ, Schmidt MH-W, Effgen S, Le DB, Wang Y, Zaidan MWAM, et al. A chromosome scale tomato genome built from complementary PacBio and Nanopore sequences alone reveals extensive linkage drag during breeding. The Plant Journal. 2022;110 2:572-88. doi:https://doi.org/10.1111/tpj.15690.

40. Legnaioli T, Cuevas J and Mas P. TOC1 functions as a molecular switch connecting the circadian clock with plant responses to drought. The EMBO Journal. 2009;28 23:3745-57.

41. Tokutsu R, Fujimura-Kamada K, Matsuo T, Yamasaki T and Minagawa J. The CONSTANS flowering complex controls the protective response of photosynthesis in the green alga Chlamydomonas. Nature Communications. 2019;10 1:4099. doi:10.1038/s41467-019-11989-x.

42. Yang T, He Y, Niu S, Yan S and Zhang Y. Identification and characterization of the CONSTANS (CO)/CONSTANS-like (COL) genes related to photoperiodic signaling and flowering in tomato. Plant Science. 2020;301:110653. doi:10.1016/j.plantsci.2020.110653.

43. Wigge PA, Kim MC, Jaeger KE, Busch W, Schmid M, Lohmann JU, et al. Integration of Spatial and Temporal Information During Floral Induction in Arabidopsis. Science. 2005;309 5737:1056-9.

44. Hortensteiner S. Stay-green regulates chlorophyll and chlorophyll-binding protein degradation during senescence. Trends in Plant Science. 2009;14 3:155-62. doi:10.1016/j.tplants.2009.01.002.

45. Lepisto A and Rintamaki E. Coordination of plastid and light signaling pathways upon development of Arabidopsis leaves under various photoperiods. Molecular Plant. 2012;5 4:799-816. doi:10.1093/mp/ssr106.

46. Wu M, Xu X, Hu X, Liu Y, Cao H, Chan H, et al. SlMYB72 Regulates the Metabolism of Chlorophylls, Carotenoids, and Flavonoids in Tomato Fruit. Plant physiology. 2020;183 3:854-68. doi:10.1104/pp.20.00156.

47. Niu XL, Li HL, Li R, Liu GS, Peng ZZ, Jia W, et al. Transcription factor SlBEL2 interferes with GOLDEN2-LIKE and influences green shoulder formation in tomato fruits. Plant Journal. 2022;112 4:982-97. doi:10.1111/tpj.15989.

48. Liu H, Zhang JQ, Zhang RR, Zhao QZ, Su LY, Xu ZS, et al. The high-quality genome of Cryptotaenia japonica and comparative genomics analysis reveals anthocyanin biosynthesis in Apiaceae. Plant Journal. 2024;118:717-30. doi:10.1111/tpj.16628.

49. Wang Z-H, Liu X, Cui Y, Wang Y-H, Lv Z-L, Cheng L, et al. Genomic, transcriptomic, and metabolomic analyses provide insights into the evolution and development of a medicinal plant Saposhnikovia divaricata (Apiaceae). Horticulture Research. 2024:uhae105. doi:10.1093/hr/uhae105.

50. Kingsford C. A fast, lock-free approach for efficient parallel counting of occurrences of k-mers. Bioinformatics. 2011;27 6:764-70.

51. Cheng H, Concepcion GT, Feng X, Zhang H and Li H. Haplotype-resolved de novo

assembly using phased assembly graphs with hifiasm. Nature Methods. 2021;18 2:170-5. doi:10.1038/s41592-020-01056-5.
52. A. SF, Waterhouse RM, Panagiotis I, Kriventseva EV and Zdobnov EM. BUSCO: assessing genome assembly and annotation completeness with single-copy orthologs. Bioinformatics. 2015; 19:3210-2.
53. Wolff J, Rabbani L, Gilsbach R, Richard G, Manke T, Backofen R, et al. Galaxy HiCExplorer 3: a web server for reproducible Hi-C, capture Hi-C and single-cell Hi-C data analysis, quality control and visualization. Nucleic Acids Research. 2020;48 1:177-84. doi:10.1093/nar/gkaa220.
54. Ou S and Jiang N. LTR_FINDER_parallel: parallelization of LTR_FINDER enabling rapid identification of long terminal repeat retrotransposons. Mobile DNA. 2019;10:48.
55. Graovac MT and Chen N. Using RepeatMasker to Identify Repetitive Elements in Genomic Sequences. Current Protocols in Bioinformatics. 2009;25 1:1-14.
56. Mario S, Oliver K, Irfan G, Alec H, Stephan W and Burkhard M. AUGUSTUS: ab initio prediction of alternative transcripts. Nucleic Acids Research. 2006;34:435-9.
57. Holt C and Yandell M. MAKER2: an annotation pipeline and genome-database management tool for second-generation genome projects. BMC Bioinformatics. 2011;12 1:491. doi:10.1186/1471-2105-12-491.
58. Nawrocki EP, Kolbe DL and Eddy SR. Infernal 1.0: inference of RNA alignments. Bioinformatics. 2009;25 10:1335-7.
59. Marçais G, Delcher AL, Phillippy AM, Coston R, Salzberg SL and Zimin A. MUMmer4: A fast and versatile genome alignment system. PLOS Computational Biology. 2018;14 1:e1005944. doi:10.1371/journal.pcbi.1005944.
60. Goel M, Sun H, Jiao W-B and Schneeberger K. SyRI: finding genomic rearrangements and local sequence differences from whole-genome assemblies. Genome Biology. 2019;20 1:277. doi:10.1186/s13059-019-1911-0.
61. Yang Z. PAML 4: Phylogenetic Analysis by Maximum Likelihood. Molecular Biology and Evolution. 2007;24 8:1586-91.
62. Bie TD, Cristianini N, Demuth JP, Hahn and W. M. CAFE: a computational tool for the study of gene family evolution. Bioinformatics. 2006;22 10:1269-71.
63. Wang YP, Tang HB, Jeremy D D, Tan X, Li JP, Wang XY, et al. MCScanX: a toolkit for detection and evolutionary analysis of gene synteny and collinearity. Nucleic Acids Research. 2012;40 7:e49.
64. Xu ZS, Yang QQ, Feng K, Yu X and Xiong AS. DcMYB113, a root-specific R2R3-MYB, conditions anthocyanin biosynthesis and modification in carrot. Plant Biotechnology Journal. 2020;18 7:1585-97. doi:10.1111/pbi.13325.

Table and figures

Table 1 Statistics for genome assembly and annotation of VF36 and Heinz 1706 genomes

**Genomic feature**	**VF36 (this study)**	**Heinz 1706 (SLT1.0)**	**Heinz 1706 (SL5.0)**
Total size of assembly contigs (Mb)	815.27	799.09	801.81
Number of contigs (gaps)	12 (0)	12 (210)	12 (31)
Number of telomeres	24	0	0
Number of centromeres	12	0	0
Number of gene models	34,783	34,384	36,648
Total size of TEs (Mb)	600.23	558.49	491.27
Annotation BUSCOs (%)	98.5	98.2	94.8
Genome BUSCOs (%)	98.64%	97.70%	97.60%

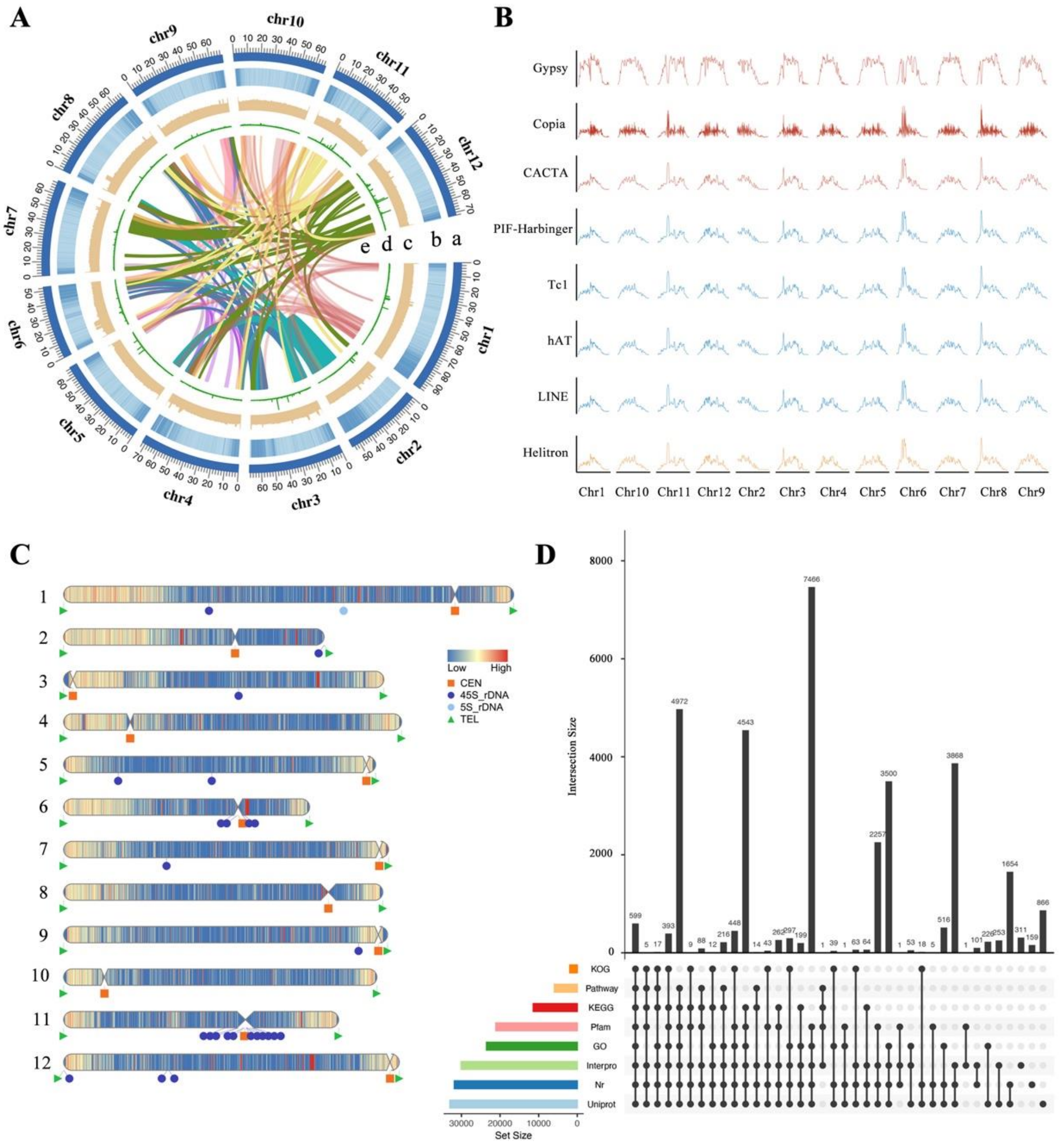

Figure 1 Complete genome assembly and annotation of VF36 tomato

(A) Circos plot of VF36 genome annotation. Quantitative tracks are aggregated in a 10-kb window. Track a, chromosomes information. Track b, gene density. Track c, GC content. Track d, repeat coverage. Track e, collinearity information.

(B) Track displaying density of *Gypsy*, *Copia*, *CACTA*, *PIF-Harbinger*, *Tc1*, *hAT*, *LINE*, and *Helitron* elements.

(C) Map of centromere prediction for VF36 genome.

(D) Distribution of VF36 genomic features.

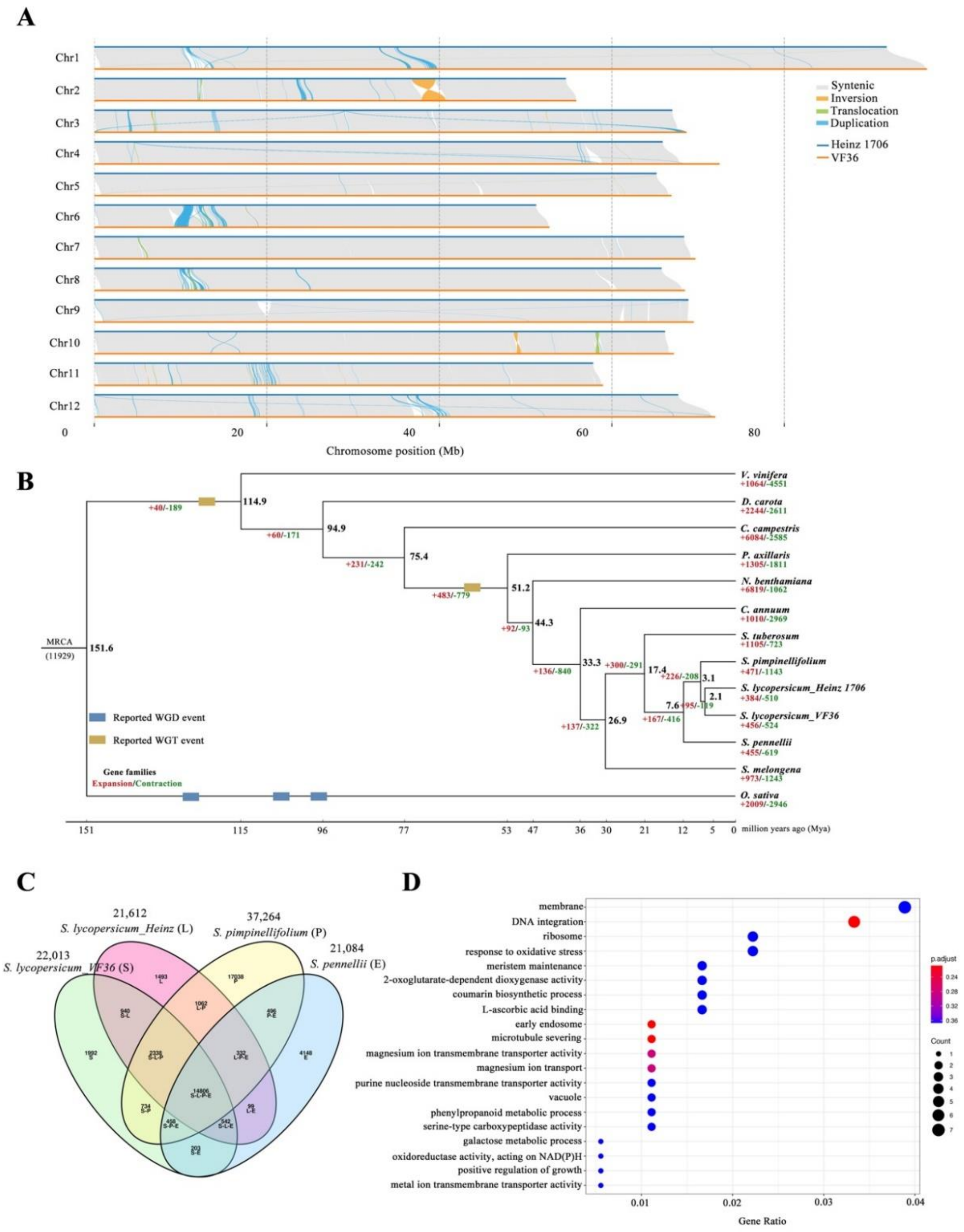

Figure 2 Comparative genomic analysis of VF36 genome.

(A) Structural variations between Heinz_1706 and VF36 genomes.

(B) Estimation of divergence time and gene family expansion/contraction. The blue blocks represent the published whole-genome duplication (WGD) events. The dark yellow blocks represent the published whole-genome triplication events.

(C) Venn diagram of gene family clustering.

(D) The GO enrichment on the genes in SV regions.

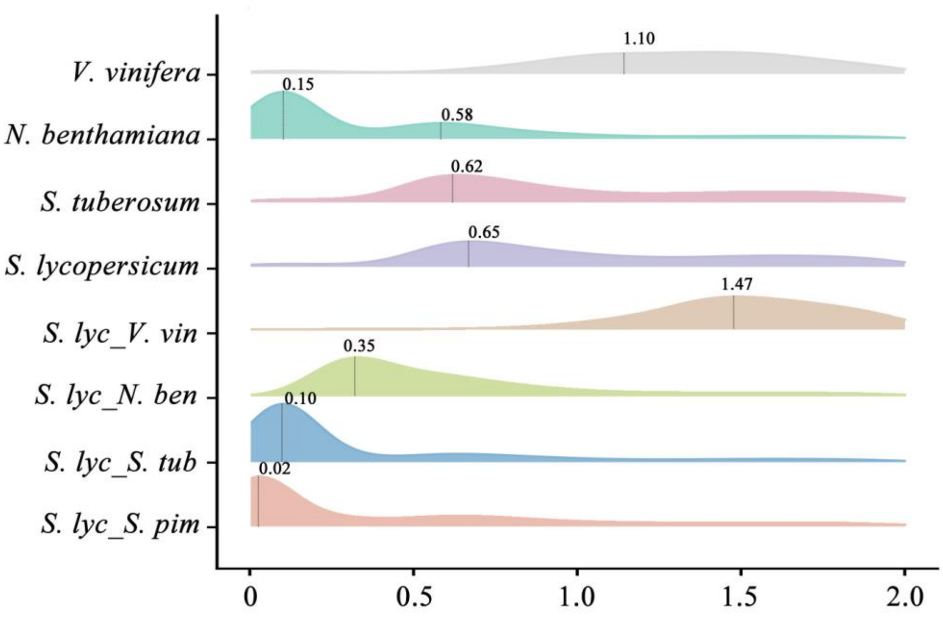

Figure 3 Gene duplication and evolution

K_S distribution from orthologs and paralogs among *S. pimpinellifolium*, *S. tuberosum*, *N. benthamiana*, *V. vinifera*, and *S. lycopersicum*.

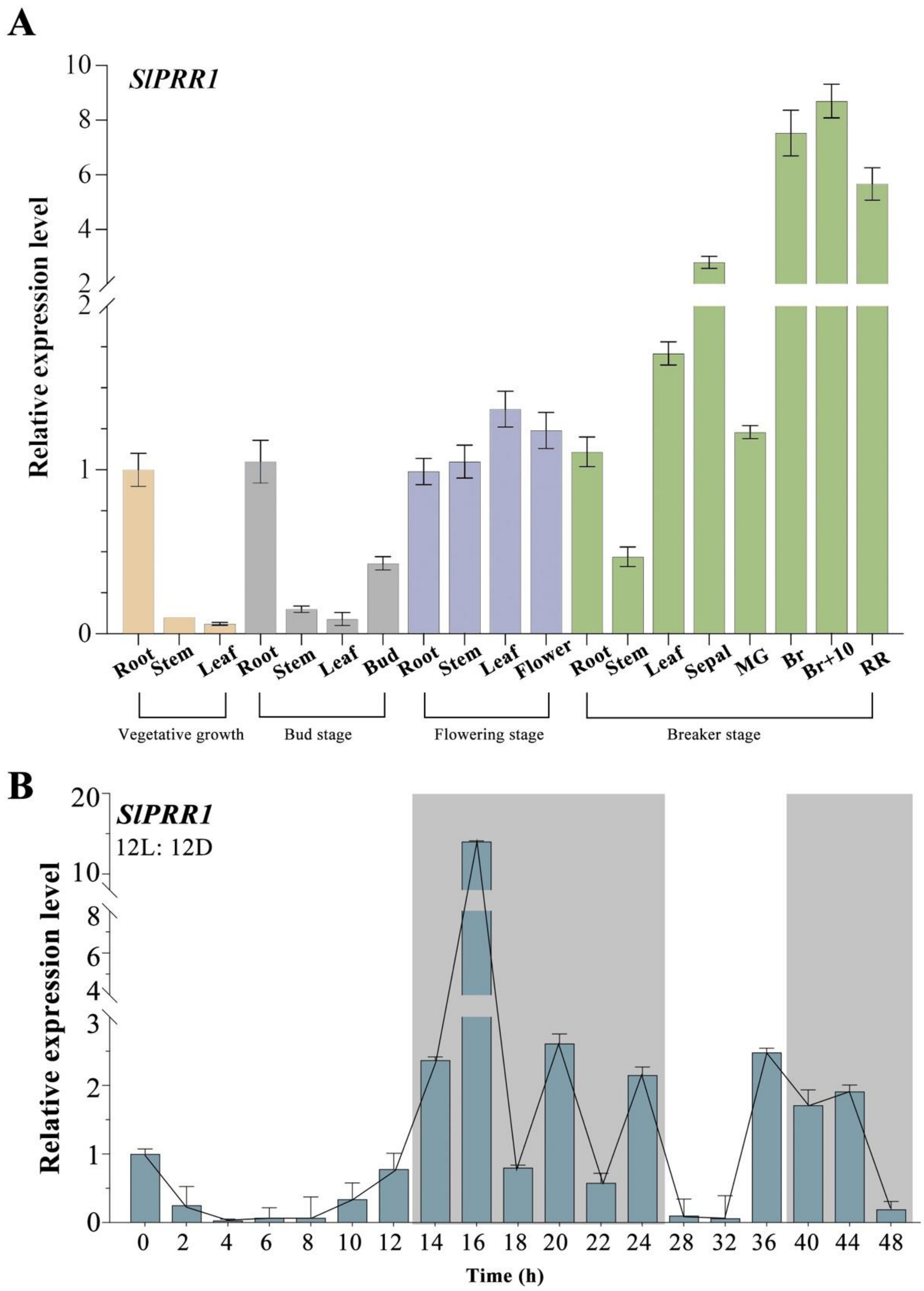

Figure 4 Expression and functional analysis of *SlPRR1* in circadian rhythm

(A) Expression of *SlPRR1* gene in various stages of tomato tissues.

(B) Expression of *SlPRR1* gene from tomato plants grown in 12L: 12D. Shading indicates the dark period.

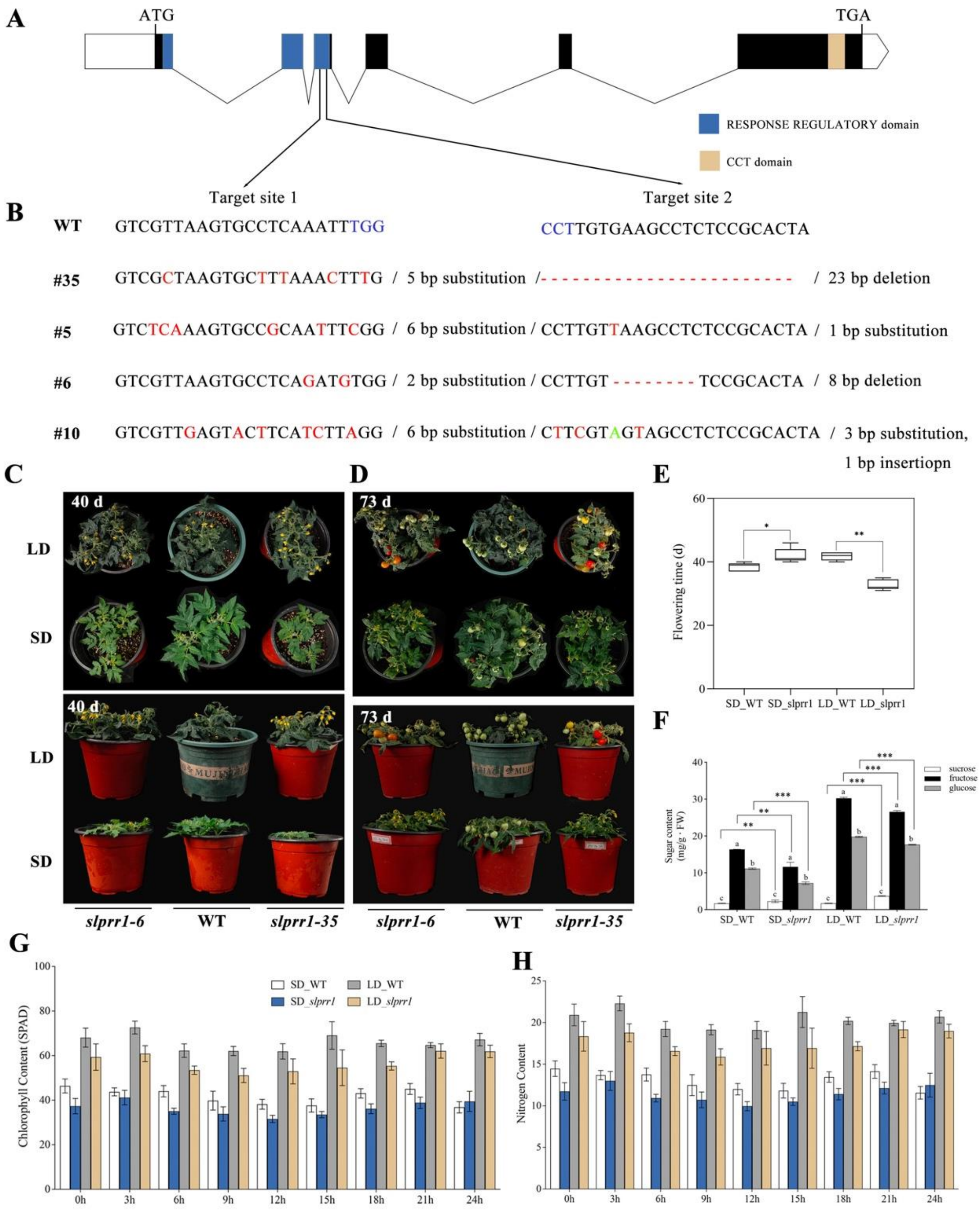

Figure 5 Functional analysis of *SlPRR1* in the regulation of tomato flowering time

(A) The two sgRNA target sites in *SlPRR1* locus used for CRISPR/Cas9 gene-editing system.

(B) The mutation types of *SlPRR1* in *slprr1*-35, *slprr1*-5, *slprr1*-6, and *slprr1*-10 line. Red letters indicated the substitution sites, green letters indicated the insertion sites, blue letters indicated the PAM.

(C-E) Flowering phenotype from 40-day-old (C), 73-day-old (D) tomato plants, and flowering time (E) of the WT, *slprr1*-6, and *slprr1*-35 lines under LD (16L: 8D) and SD (8L: 16D) conditions.

(F) The content of sucrose, fructose, and glucose of red ripening tomato fruits in WT and *slprr1* lines under SD or LD conditions.

(G-H) The Chhlorophyll (G) and Nitrogen (H) content of tomato leaves in WT and *slprr1* lines under SD or LD conditions during the photoperiod.

803

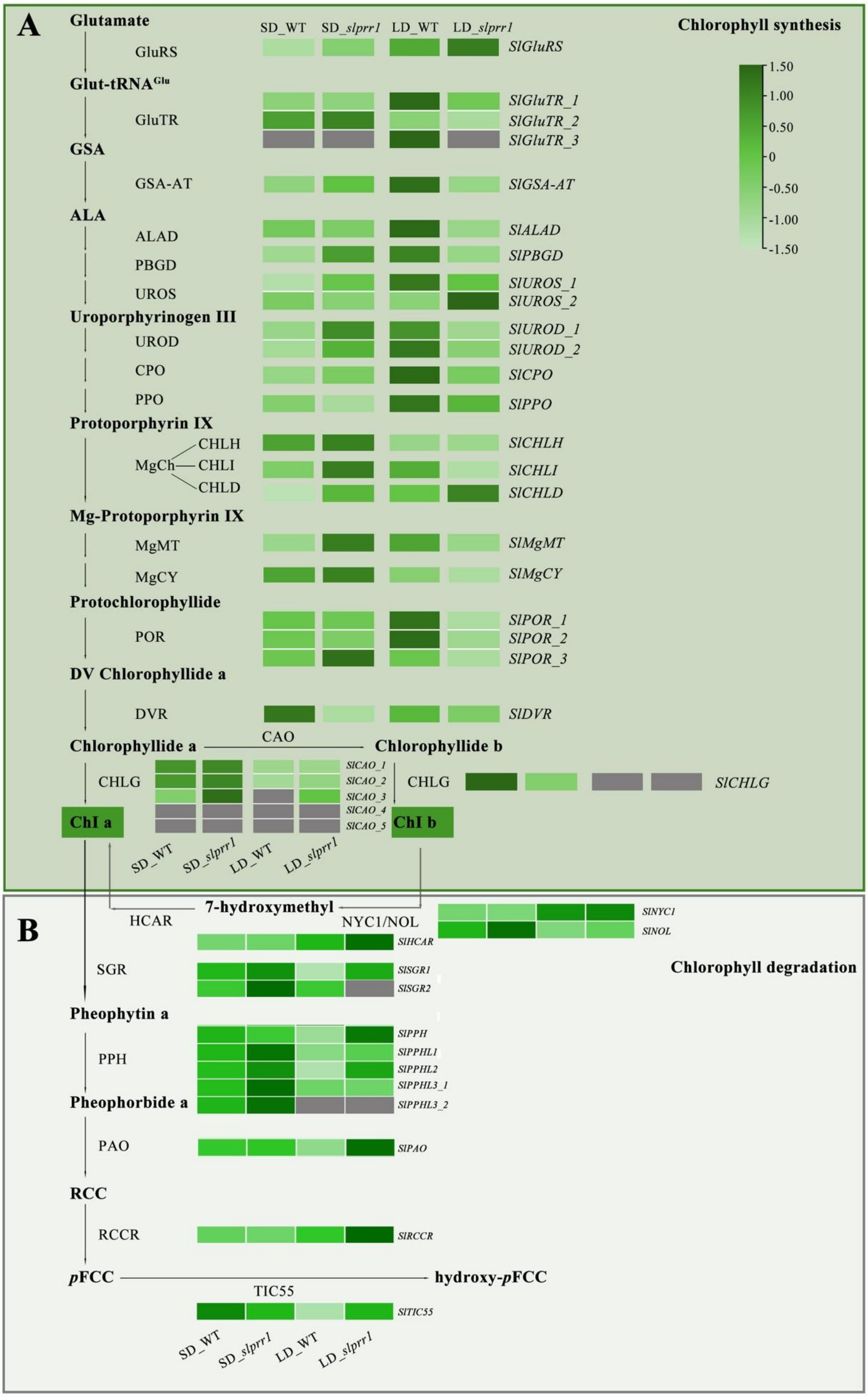

804 **Figure 6 Chlorophyll synthesis and degradation of tomato leaves in WT and *slprr1***

lines under SD or LD conditions

(A) The potential simplified Chlorophyll synthesis pathway in tomato leaves is depicted.

(B) The potential simplified Chlorophyll degradation pathway in tomato leaves is depicted.

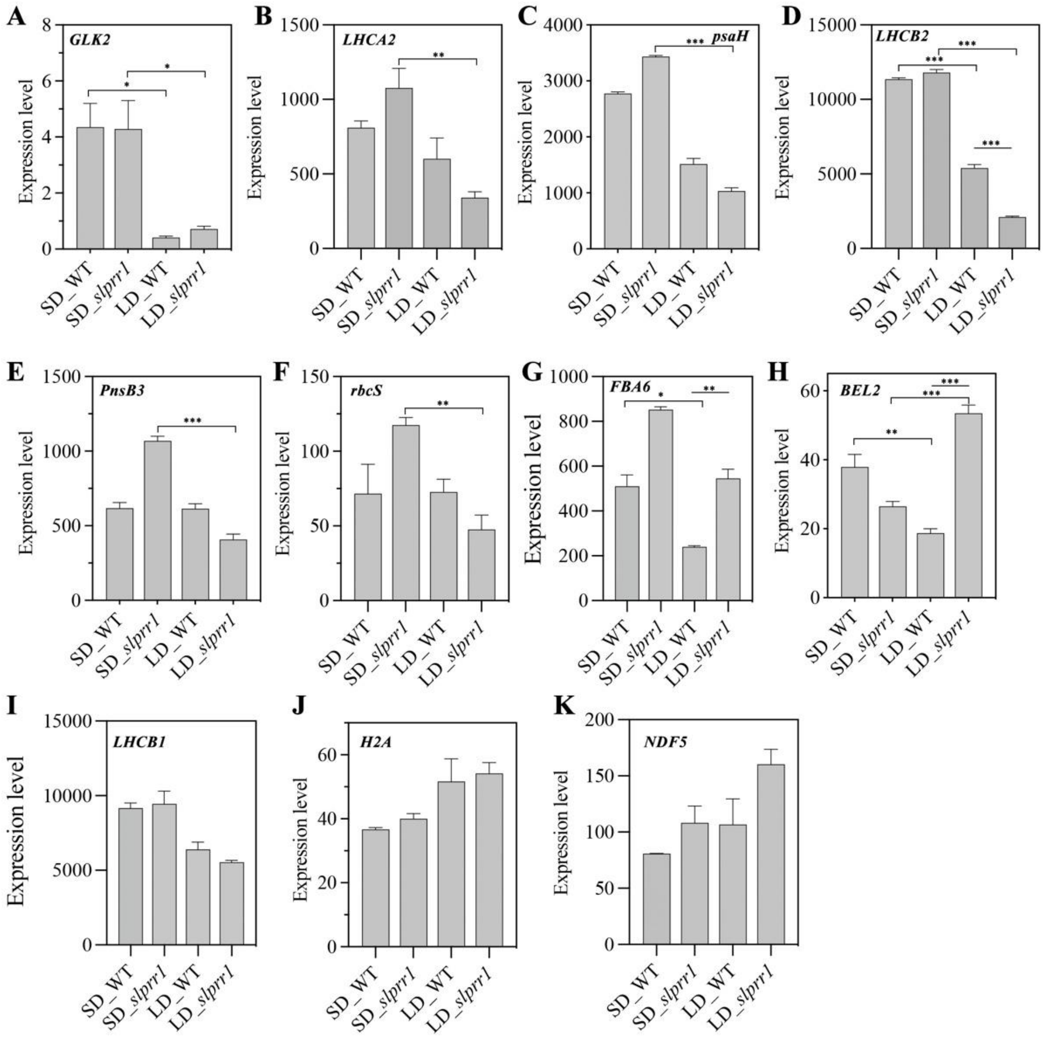

Figure 7 Relative expression levels of photosynthesis and Chlorophyll synthesis-related genes in tomato leaves. Error bars represent the averages of three biological replicates ± SD. Asterisks indicate statistical significance (***P* < 0.01, **P* < 0.05, Student's *t*-test).

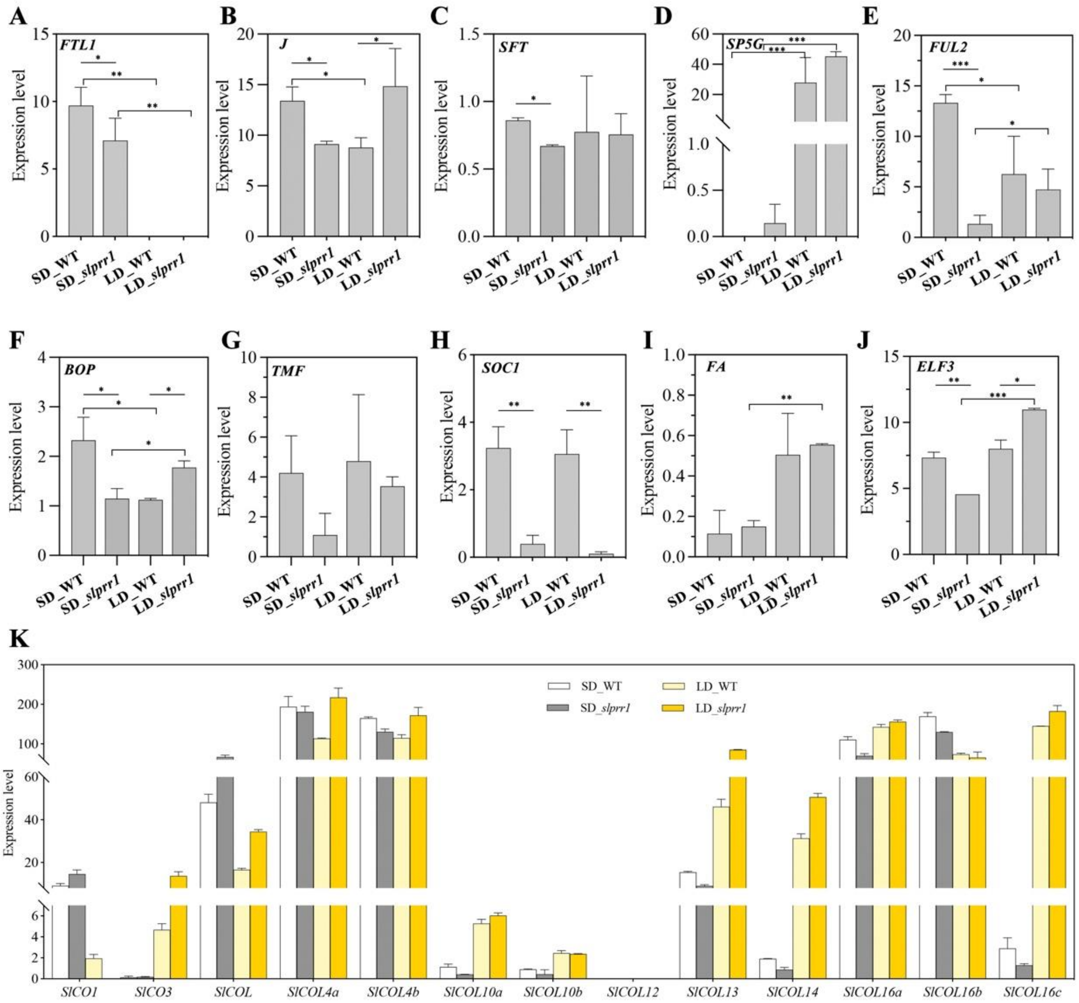

Figure 8 Relative expression levels of flowering-related genes in tomato. Error bars represent the averages of three biological replicates ± SD. Asterisks indicate statistical significance (***P* < 0.01, **P* < 0.05, Student's *t*-test).

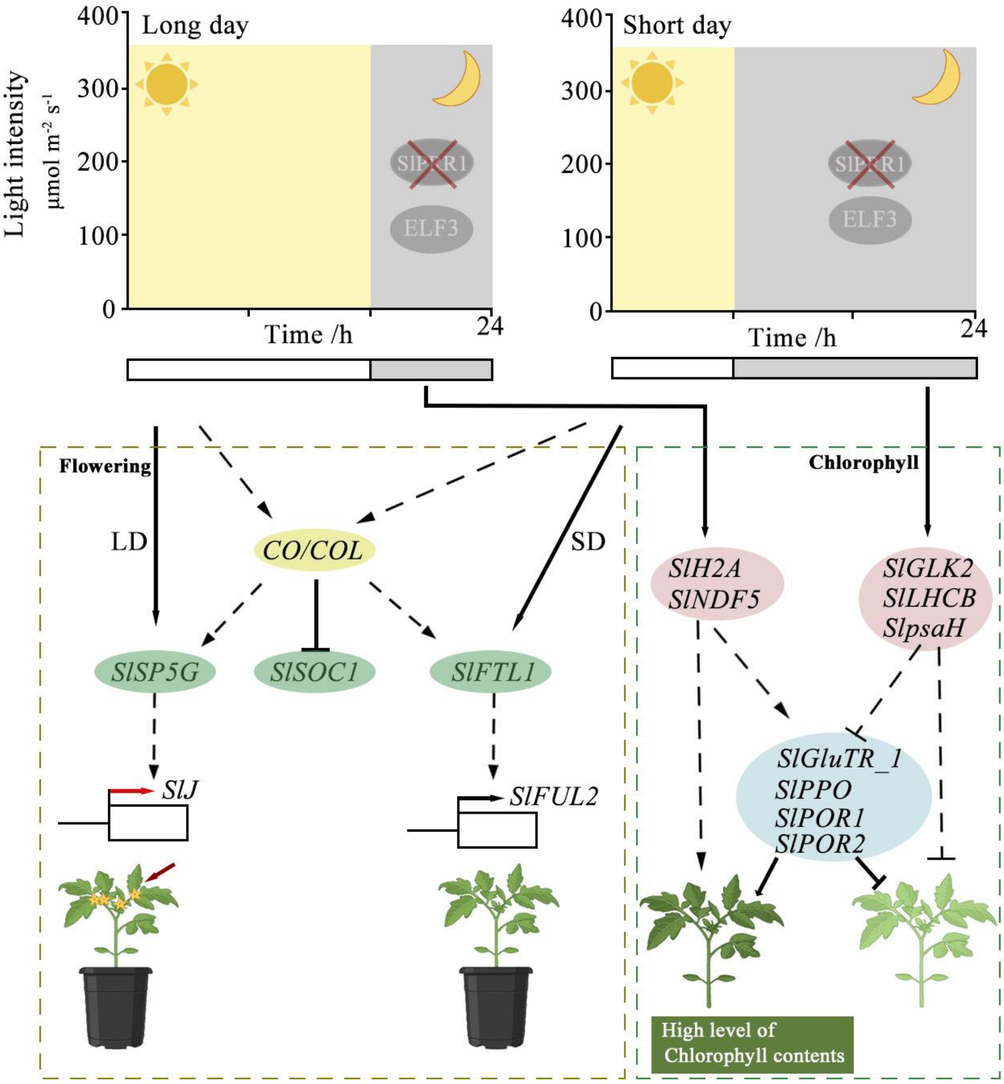

Figure 9 Model for the regulation of flowering time and Chlorophyll synthesis by knock-out *SlPRR1* in tomato under LD and SD conditions

Supplementary material

Supplementary Table 1. *K*-mer statistics of the genomic characteristics of 'VF36' tomato obtained by genome.

Supplementary Table 2. Summary of sequencing data.

Supplementary Table 3. Summary of assembly data.

Supplementary Table 4. General statistics of the telomeres for VF36 genome.

Supplementary Table 5. Predicted centromeric regions.

Supplementary Table 6. Statistics of repeat content in VF36 genome.

Supplementary Table 7. General statistics of predicted protein-coding genes.

Supplementary Table 8. BUSCOs analysis of VF36 genome completeness.

Supplementary Table 9. Functional annotation of the predicted genes.

Supplementary Table10. The statistics of non-coding RNA in VF36 genome.

Supplementary Table11. The statistics of variation between VF36 and Heinz1706 genome.

Supplementary Table12. Structure variations between VF36 and Heinz1706 genome.

Supplementary Table 13. Gene sets used in this study.

Supplementary Table 14. Summary of clustered gene families by OrthoFinder among 13 species.

Supplementary Table 15. The information of collinear gene pairs.

Supplementary Table 16. The primers used in this study.

Figure S1 'VF36' tomato fruits of MG and RR stages.

Figure S2 *K*-mer=19 Depth and *K*-mer number frequency distribution map.

Figure S3 Twelve pseudomolecules scaffolding with Hi-C data.

Figure S4 Density of genes and TRs, blue lines represent LTR/Copia, orange lines

represent LTR/Gyps, dark red lines represent TRF, and green lines represent genes.

Figure S5 GO enrichment of expanded gene families.

Figure S6 GO enrichment of unique genes.

Figure S7 Syntenic dot plot of *V. vinifera* versus ‘VF36’ tomato.

Figure S8 Syntenic dot plot of *N. benthamiana* versus ‘VF36’ tomato.

 Click here to access/download;Table;Table 1.xlsx

Table1 Statistics for genome assembly and annotation of VF36 and Heinz 1706 genom

Genomic feature	VF36 (this study)	Heinz 1706 (SLT1.0)
Total size of assembly contigs (Mb)	815.27	799.09
Number of contigs (gaps)	12 (0)	12 (210)
Number of telomeres	24	0
Number of centromeres	12	0
Number of gene models	34,783	34,384
Total size of TEs (Mb)	600.23	558.49
Annotation BUSCOs (%)	98.5	98.2
Genome BUSCOs (%)	98.64%	97.70%

es.

Heinz 1706 (SL5.0)
801.81
12 (31)
0
0
36,648
491.27
94.8
97.60%

Figure 1

Click here to access/download;Figure;Figure 1.jpg

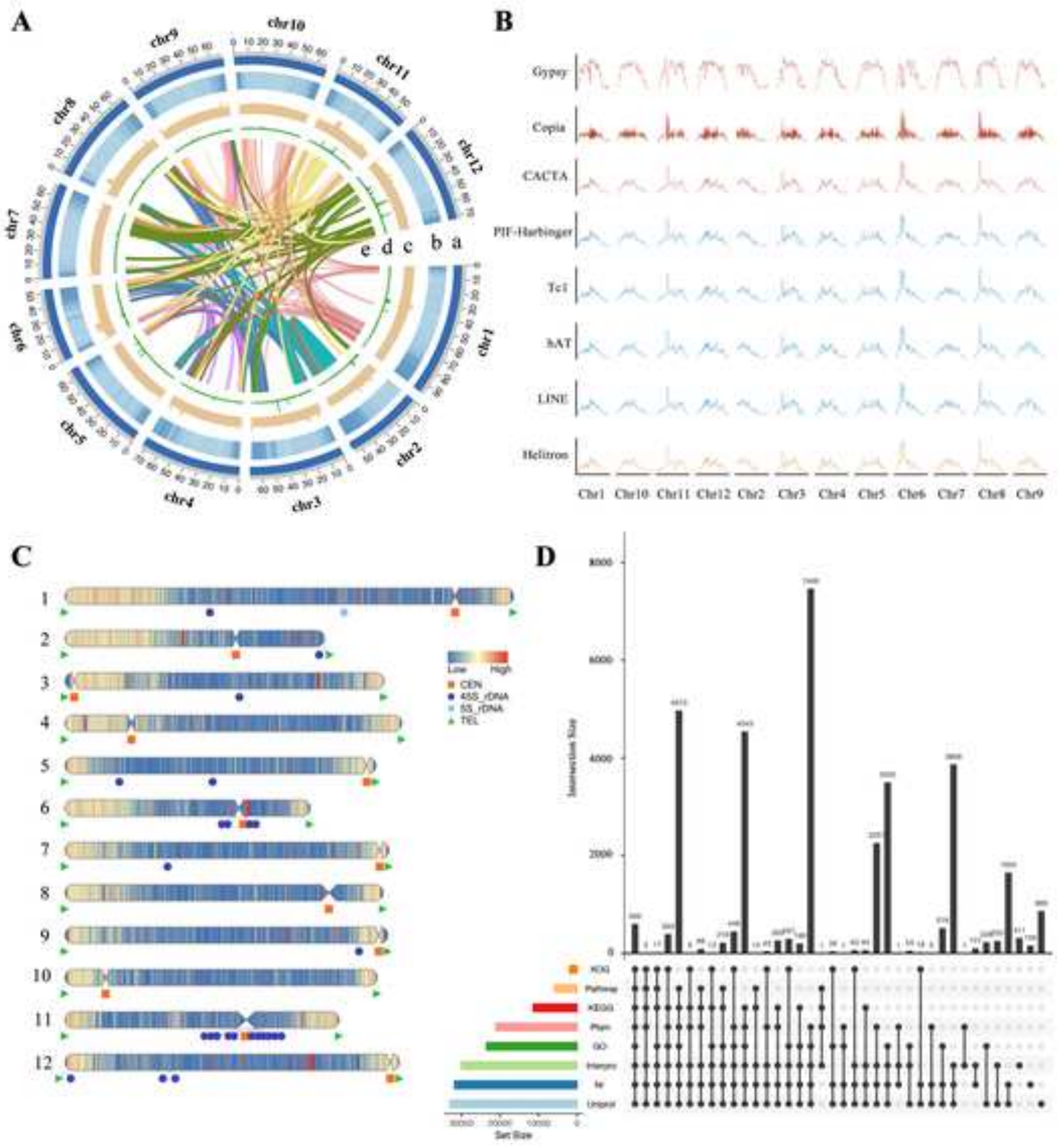

Figure 2

Click here to access/download;Figure;Figure 2.jpg

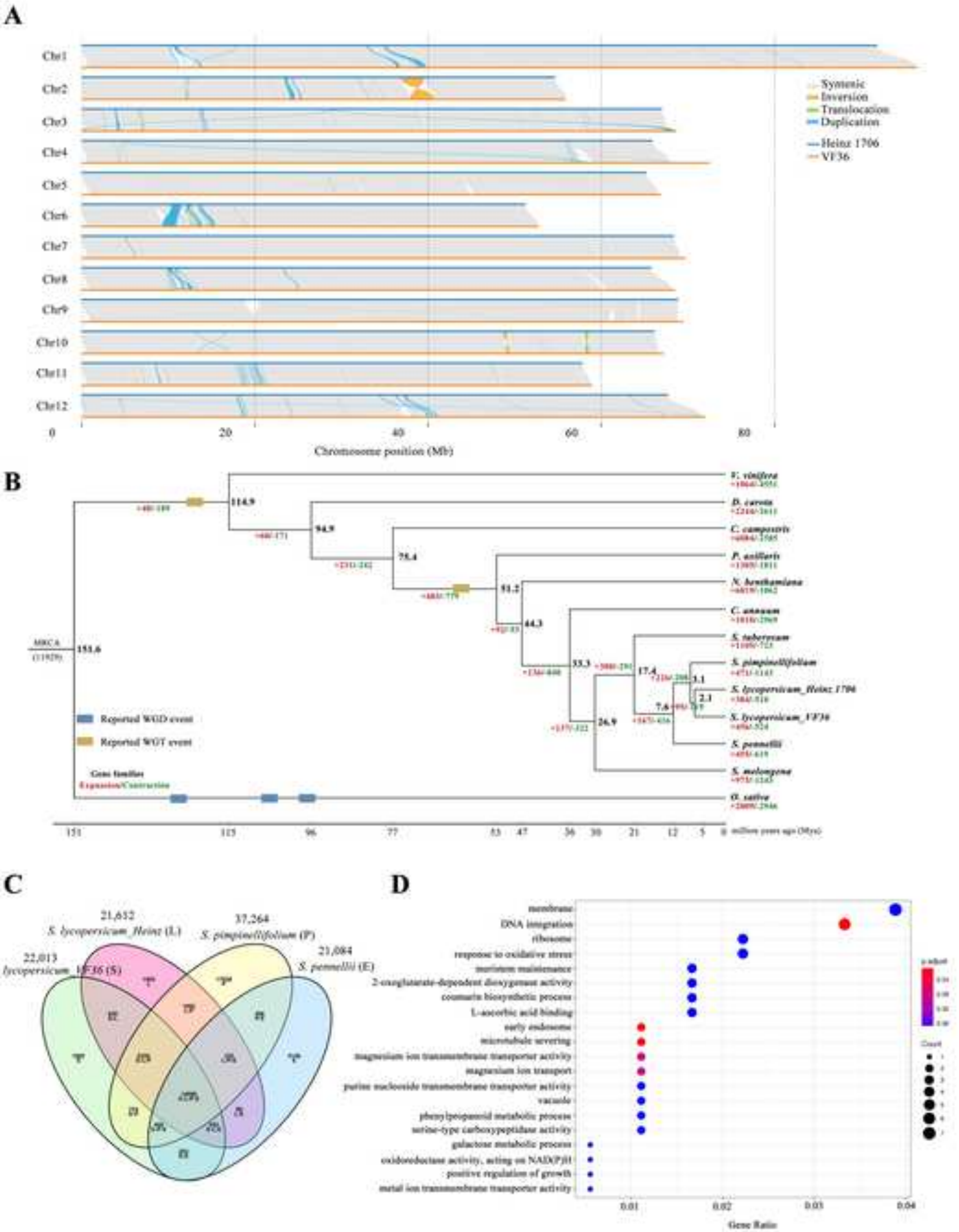

Figure 3

Click here to access/download;Figure;Figure 3.jpg

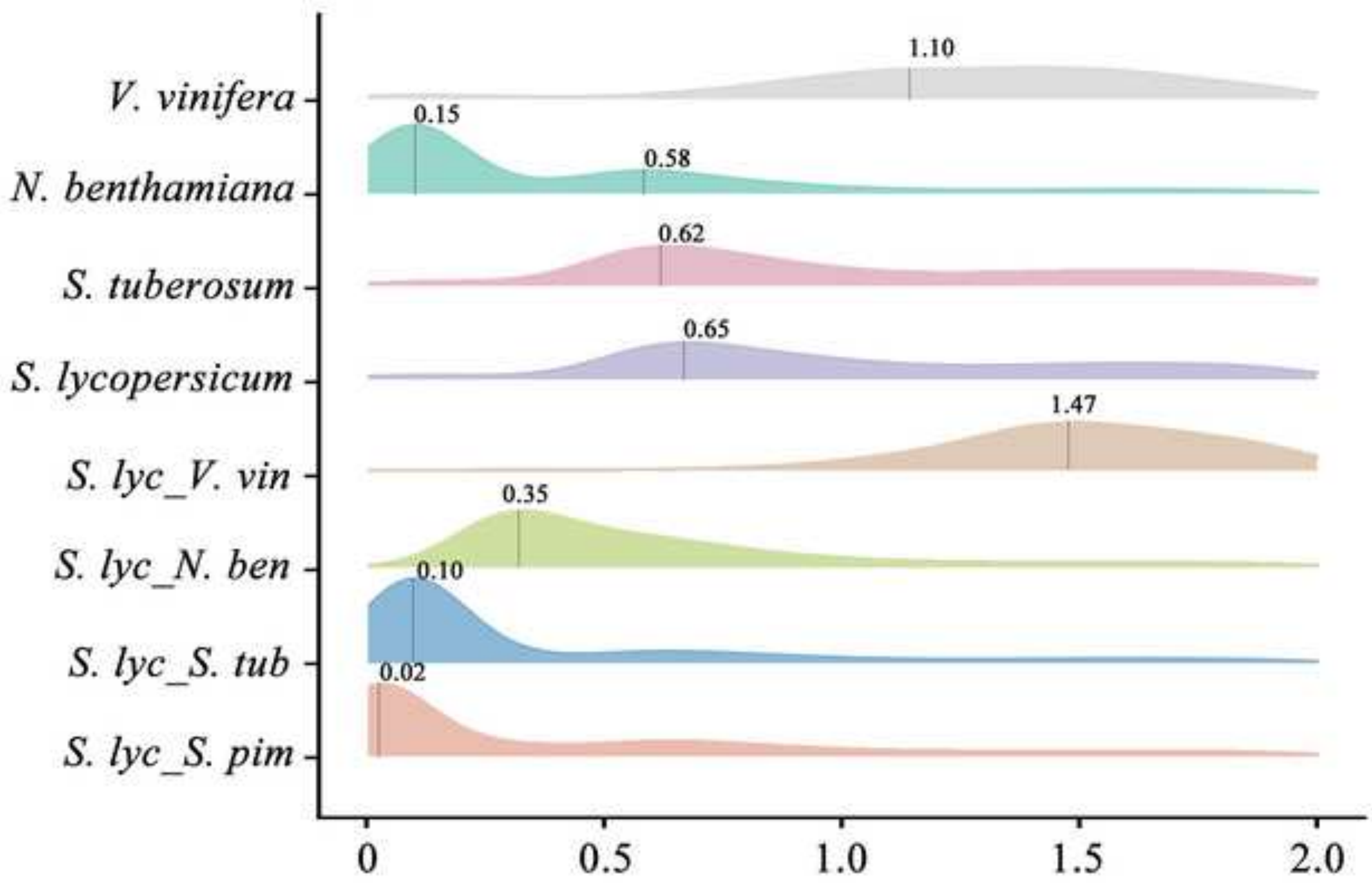

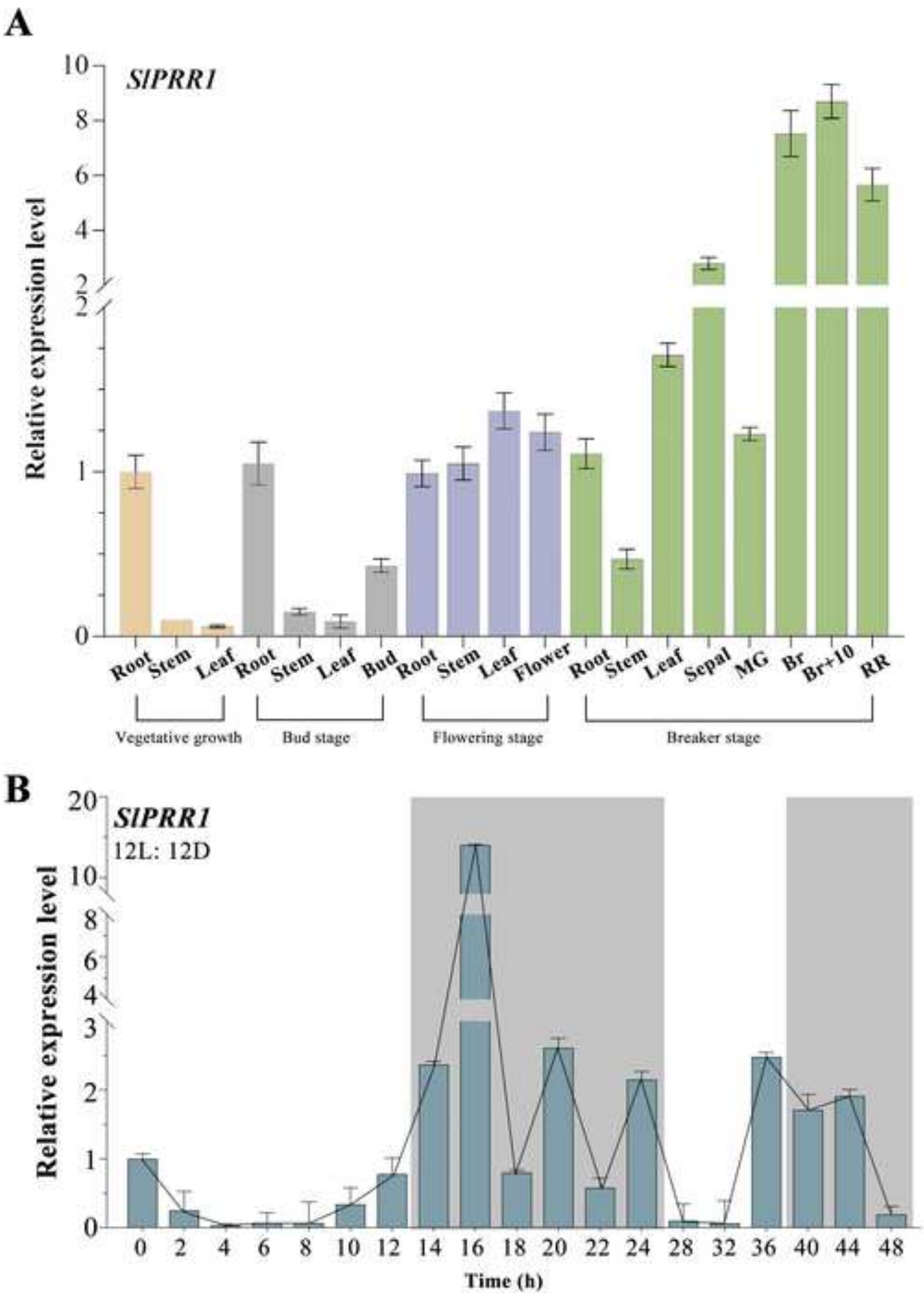
A
SlPRR1
Relative expression level
10
8
6
4
2
2
1
0
Root
Stem
Leaf
Root
Stem
Leaf
Bud
Root
Stem
Leaf
Flower
Root
Stem
Leaf
Sepal
MG
Br
Br+10
RR
Vegetative growth
Bud stage
Flowering stage
Breaker stage
B
SlPRR1
12L: 12D
Relative expression level
20
10
8
6
4
3
2
1
0
0
2
4
6
8
10
12
14
16
18
20
22
24
28
32
36
40
44
48
Time (h)

Figure 5

Click here to access/download;Figure;Figure 5.jpg

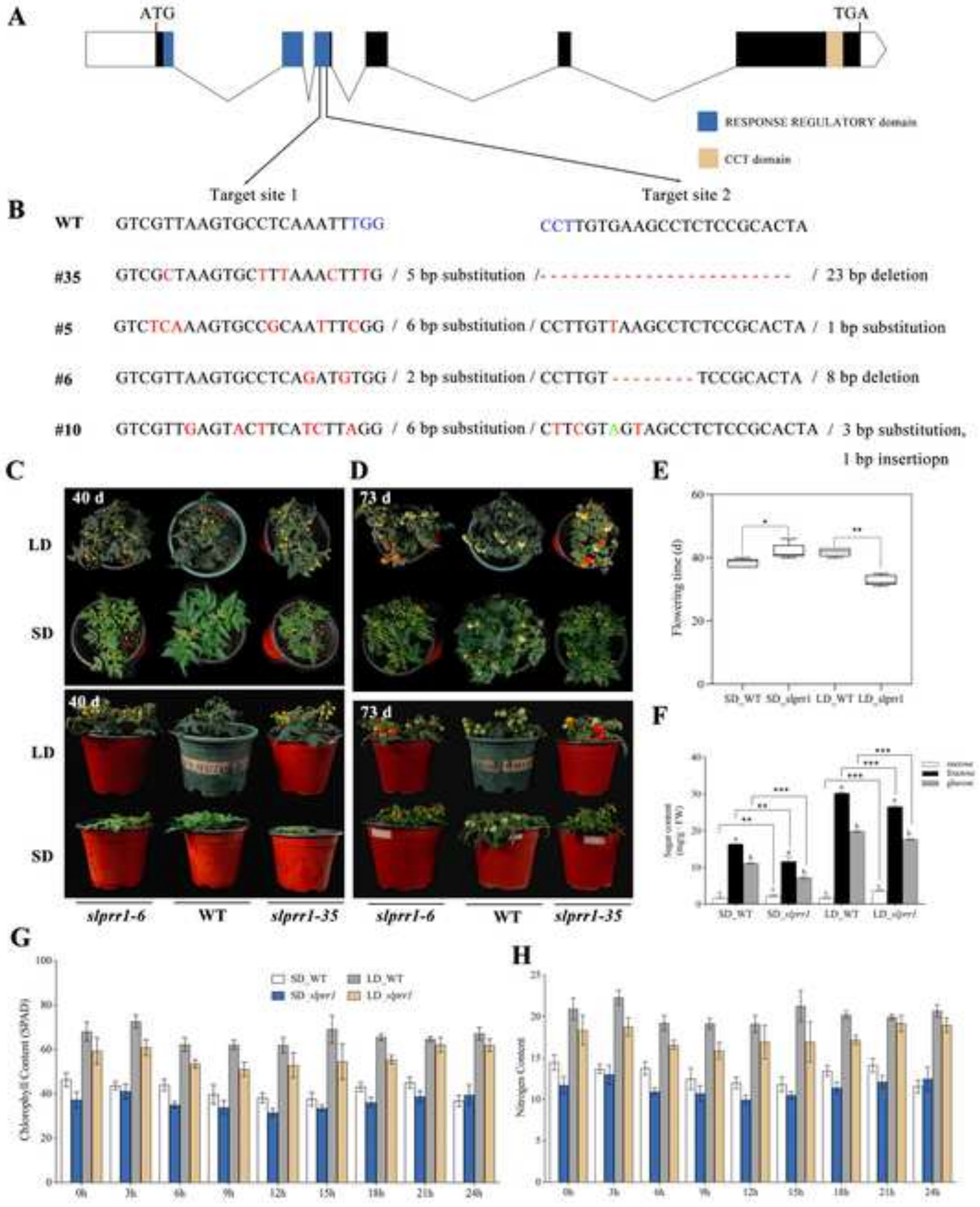

Figure 6

Click here to access/download;Figure;Figure 6.jpg

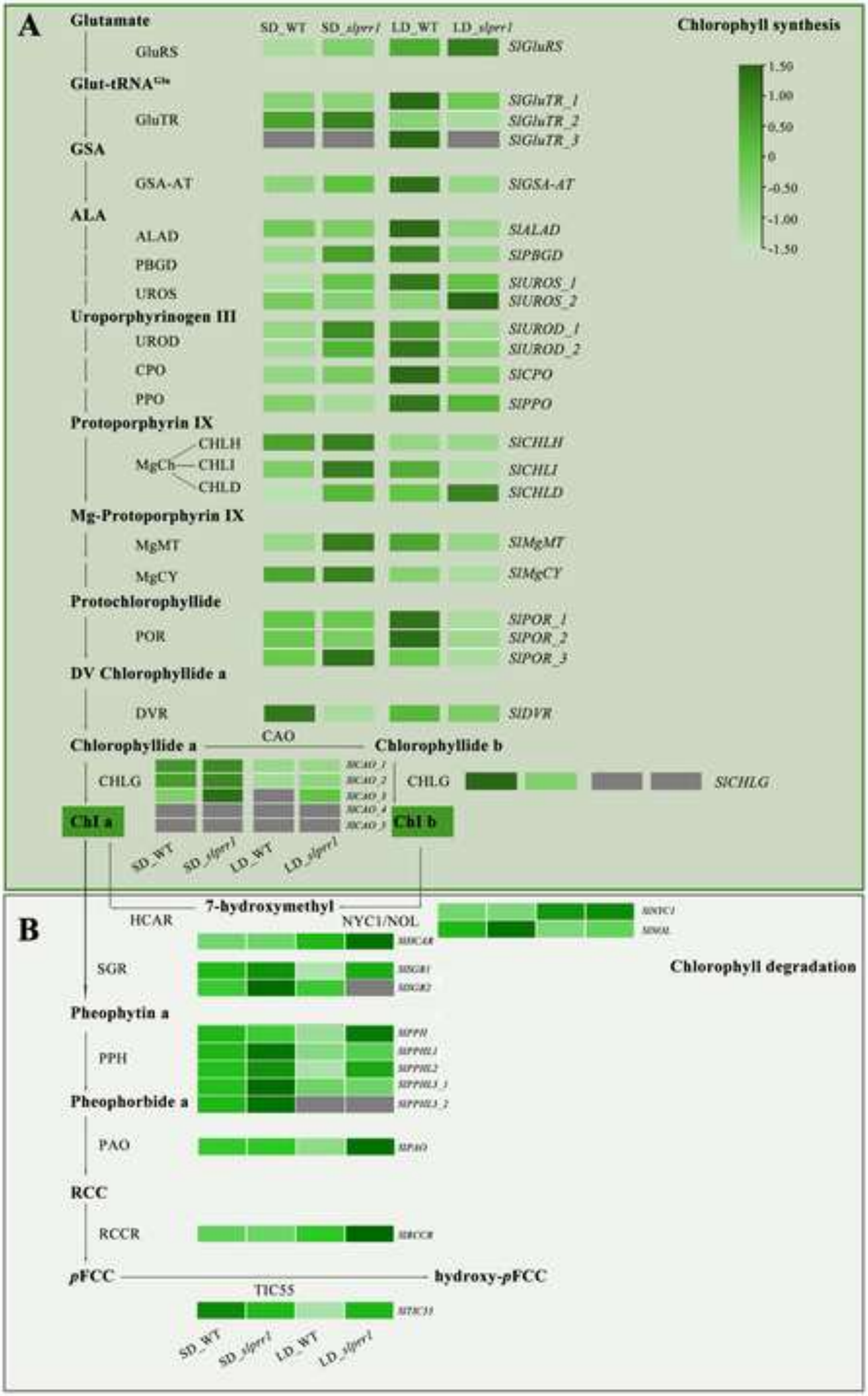

Figure 7

Click here to access/download;Figure;Figure 7.jpg

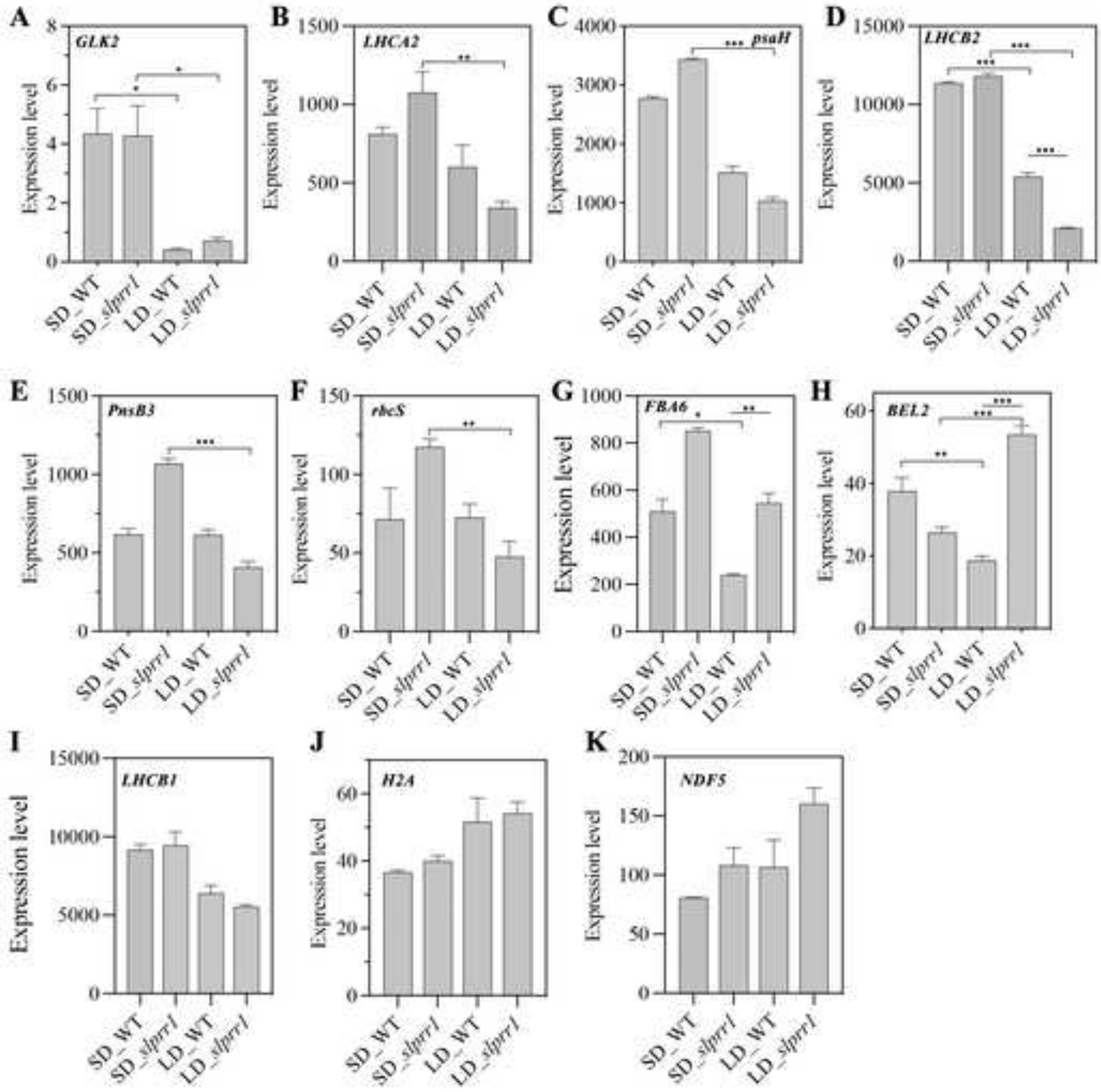

Click here to access/download;Figure;Figure 8.jpg

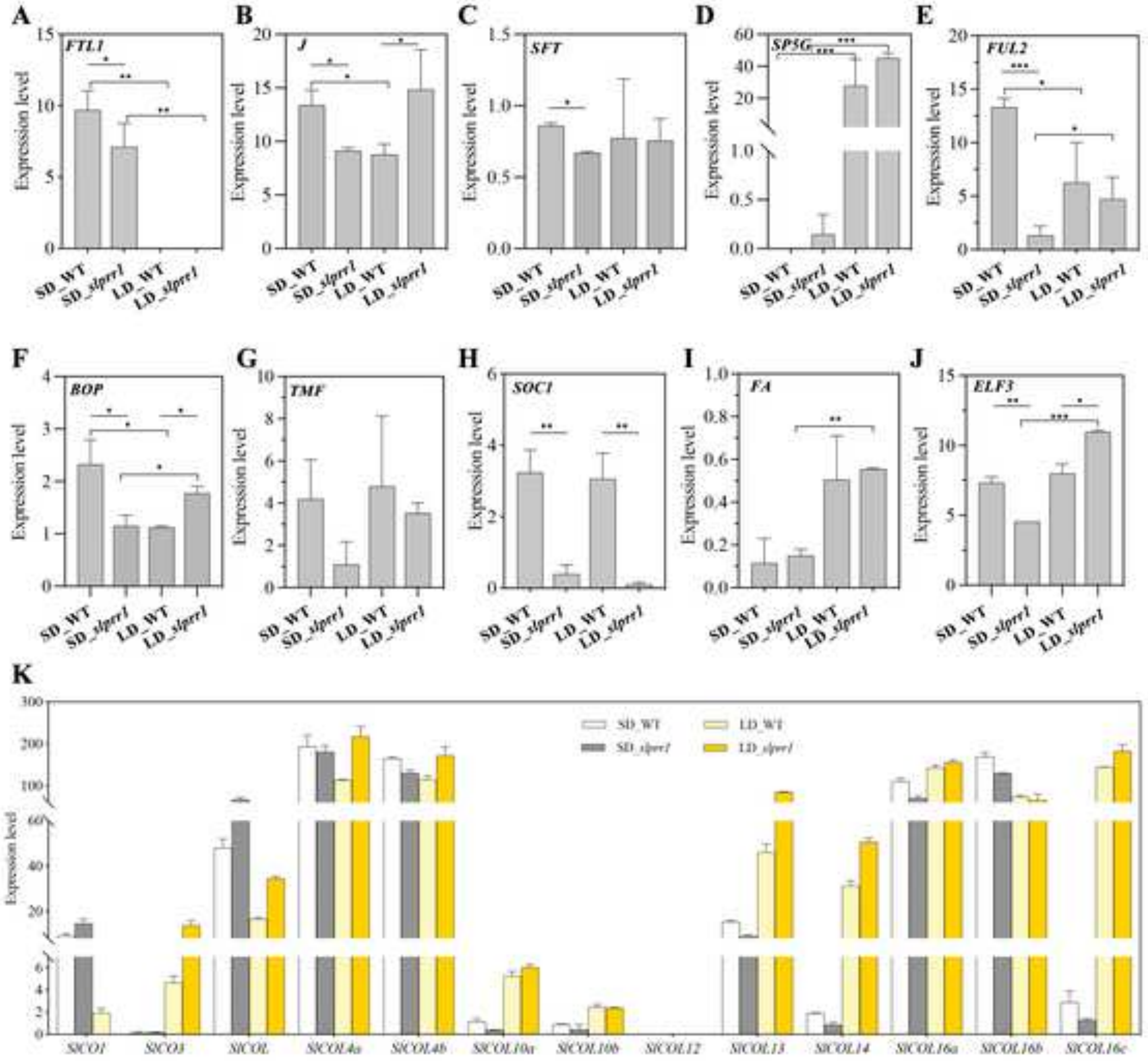

Figure 9

Click here to access/download;Figure;Figure 9.jpg

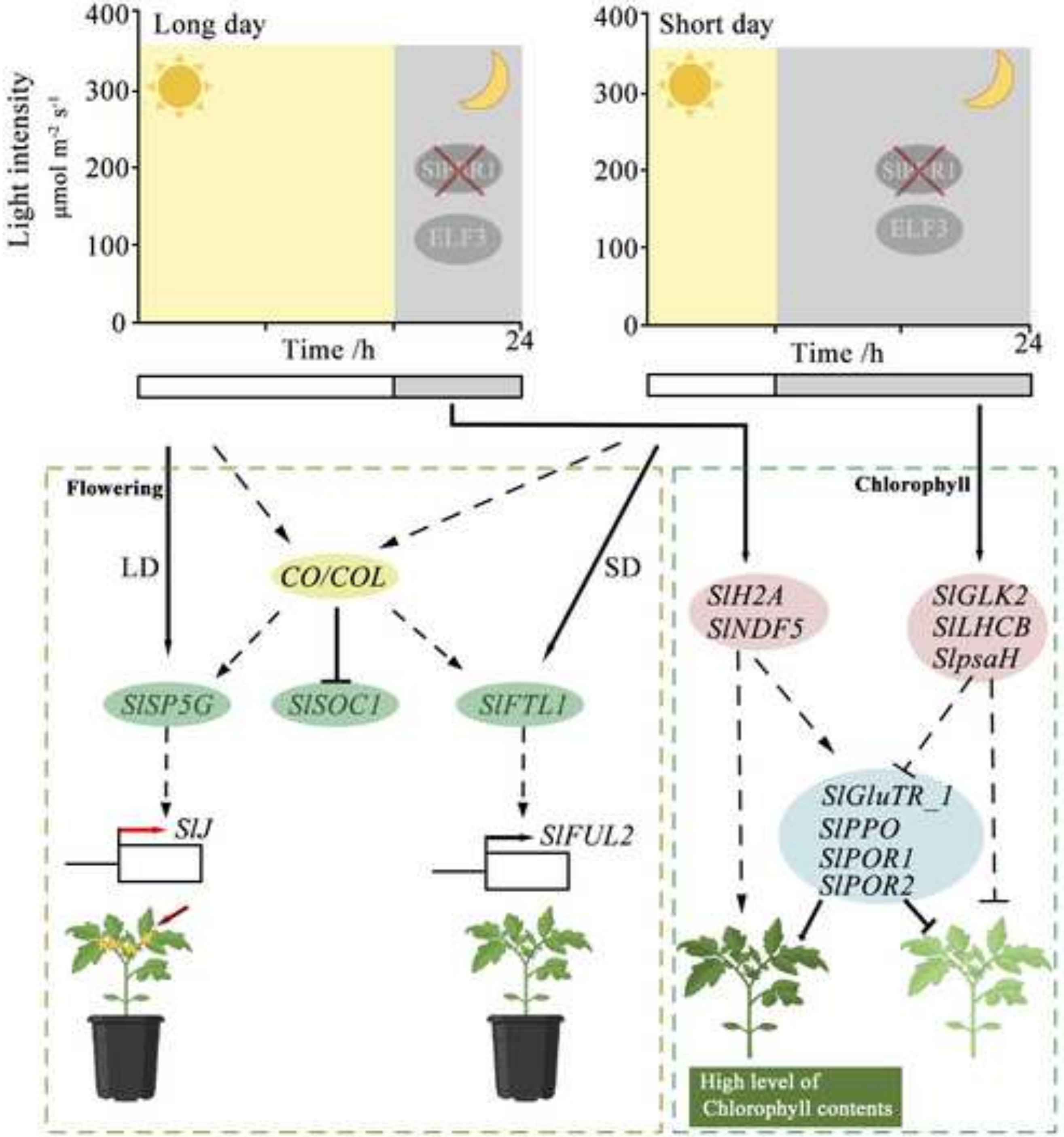

Supplementary figure 1

Click here to access/download
Supplementary Material
Figure S1.jpg

Supplementary figure 2

Click here to access/download
Supplementary Material
Figure S2.jpg

Supplementary figure 3

Click here to access/download
Supplementary Material
Figure S3.jpg

Supplementary figure 4

Click here to access/download
Supplementary Material
Figure S4.jpg

Supplementary figure 5

Click here to access/download
Supplementary Material
Figure S5.jpg

Supplementary figure 6

Click here to access/download
Supplementary Material
Figure S6.jpg

Supplementary figure 7

Click here to access/download
Supplementary Material
Figure S7.jpg

Supplementary figure 8

Click here to access/download
Supplementary Material
Figure S8.jpg

Supplementary tables

Click here to access/download
Supplementary Material
supplementary tables.xlsx

Personal Cover
Click here to access/download;Personal Cover;1coverletter.docx

GigaScience
13th Dec, 2024

A telomere-to-telomere gapless genome reveals SlPRR1 control circadian rhythm and photoperiodic flowering in cultivated tomato

Dear Editor,

Please find our manuscript entitled "**A telomere-to-telomere gapless genome reveals SlPRR1 control circadian rhythm and photoperiodic flowering in cultivated tomato**", which we would like to submit for publication in ***GigaScience***.

The cultivated tomato (*Solanum lycopersicum*) is a major vegetable fruit with high economic values and plays role as an important model for the study of flowering time of day-neutral (ND) plants. Completely continuous and gap-less genome of cultivated tomato is needed to decipher the genetic research and breeding program. We reported a telomere-to-telomere (T2T) gap-free genome of *S. lycopersicum* cv. VF36 constructed by combined sequencing technologies. Through comparative genomics and phylogenetic analysis, we predicted structure variations (SVs) between 'VF36' and 'Heinz 1706' genome. Furthermore, a core circadian oscillator SlPRR1 was identified, which expressed peaked at nighttime with circadian rhythm. CRISPR/Cas9 knockdown *SlPRR1* in tomato demonstrated that *slprr1* mutant lines significantly early flowering under long-day (LD) condition and delayed flowering under short-day (SD) condition. We surveyed expression of a group of genes which had been implicated in flowering time control in tomato, including *FTL1*, *J*, *SFT*, *SP5G*, *BOP*, *TMF*, *SOC1*, and *FA*. We presented the hypothetical model how SlPRR1 regulates flowering time and Chlorophyll biosynthesis to adjust to photoperiod.

We believe these findings will be of great interest to biologists, and particularly to researchers working on this field. As the international journal devoted to the rapid dissemination of scientific findings, ***GigaScience*** represents the perfect platform for us to share these results with the international research community. As the corresponding

author of this manuscript, I hereby certify that this manuscript has not been published elsewhere and is not under consideration by another journal. All authors support the contents of this manuscript and its submission to ***GigaScience.***

We shall look forward to hearing from you at your earliest convenience.

Yours sincerely,
Ai-Sheng Xiong

Dr. Ai-Sheng Xiong
Professor
State Key Laboratory of Crop Genetics & Germplasm Enhancement and Utilization,
College of Horticulture, Nanjing Agricultural University,
1 Weigang, 210095 Nanjing, China
Fax: 86 25 84396790
Email: xiongaisheng@njau.edu.cn
