# Supplementary material for: A telomere-to-telomere gapless genome reveals SlPRR1 control of circadian rhythm and photoperiodic flowering in tomato
Source: Gigascience. 2025 Jul 2;14:giaf058. doi: 10.1093/gigascience/giaf058 (PMC12218202; doi:10.1093/gigascience/giaf058)
Supplement: giaf058_GIGA-D-24-00568_Revision_1 [file giaf058_giga-d-24-00568_revision_1.pdf]

GigaScience

A telomere-to-telomere gapless genome reveals SlPRR1 control of circadian rhythm and photoperiodic flowering in tomato

--Manuscript Draft--

**Manuscript Number:**	GIGA-D-24-00568R1
**Full Title:**	A telomere-to-telomere gapless genome reveals SlPRR1 control of circadian rhythm and photoperiodic flowering in tomato
**Article Type:**	Research
**Funding Information:**	National Natural Science Foundation of China (32402552) — Dr. Hui Liu<br>Natural Science Research of Jiangsu Higher Education Institutions of China (BK20221009) — Dr. Hui Liu<br>Jiangsu Provincial Key Research and Development Program (BE2023350) — Dr. Jian-Ping Tao
**Abstract:**	Cultivated tomato (Solanum lycopersicum) is a major vegetable crop of high economic values that serves as an important model for studying flowering time in day-neutral (ND) plants. A complete, continuous, and gapless genome of cultivated tomato is essential for genetic research and breeding programs. Here, we reported the construction of a telomere-to-telomere (T2T) gap-free genome of S. lycopersicum cv. VF36 using a combination of sequencing technologies. The 815.27 Mb T2T 'VF36' genome contained 600.23 Mb of transposable elements (TEs). Through comparative genomics and phylogenetic analysis, we identified structural variations (SVs) between the 'VF36' and 'Heinz 1706' genomes and found no evidence of a recent species-specific whole-genome duplication (WGD) in the 'VF36' tomato. Furthermore, a core circadian oscillator, SlPRR1, was identified, which peaked at night in a circadian rhythm. CRISPR/Cas9-mediated knockdown of SlPRR1 in tomatoes demonstrated that slprr1 mutant lines exhibited significantly earlier flowering under long-day (LD) condition than wild type (WT). We present a hypothetical model of how SlPRR1 regulates flowering time and chlorophyll biosynthesis in response to photoperiod. This T2T genomic resources will accelerate the genetic improvement of large-fruited tomatoes, and the SlPRR1-related hypothetical model will enhance our understanding of the photoperiodic response in cultivated tomatoes, revealing a regulatory mechanism for manipulating flowering time.
**Corresponding Author:**	Ai-Sheng Xiong, Ph.D<br>Nanjing Agricultural University<br>nanjing, CHINA
**Corresponding Author Secondary Information:**	
**Corresponding Author's Institution:**	Nanjing Agricultural University
**Corresponding Author's Secondary Institution:**	
**First Author:**	Hui Liu
**First Author Secondary Information:**	
**Order of Authors:**	Hui Liu<br>Jia-Qi Zhang<br>Jian-Ping Tao<br>Chen Chen<br>Li-Yao Su<br>Jin-Song Xiong

Powered by Editorial Manager® and ProduXion Manager® from Aries Systems Corporation

	Ai-Sheng Xiong, Ph.D
**Order of Authors Secondary Information:**	
**Response to Reviewers:**	2 April 2025<br>GigaScience<br>Manuscript ID: GIGA-D-24-00568<br>A telomere-to-telomere gapless genome reveals SlPRR1 control circadian rhythm and photoperiodic flowering in cultivated tomato<br><br>Dear Prof. Zhang<br>Thank you very much for your assistance in handing our manuscript (A telomere-to-telomere gapless genome reveals SlPRR1 control circadian rhythm and photoperiodic flowering in cultivated tomato). Your effort and time spent on our manuscript are greatly appreciated by all of us. We are delighted to all suggestions and review comments, which you and the reviewers made. Your revisions/suggestions have definitely improved the quality of our manuscript.<br>The manuscript was edited extensively according to reviewer' comments. The language of the manuscript has been improved by a copy-editing company. Please find the revised manuscript in 'GigaScience' manuscript center. The changes were made directly in the text with RED marked. The responses to the reviewers are highlighted below.<br>Thank you again for your kind help and excellent suggestions for our manuscript. We hope these revisions will be satisfactory and will lead to acceptance for publication. We are looking forward to hearing from you soon.<br><br>Yours sincerely<br>Ai-Sheng Xiong<br>--------------------------<br>Dr. Ai-Sheng Xiong<br>Professor<br>State Key Laboratory of Crop Genetics & Germplasm Enhancement and Utilization,<br>College of Horticulture,<br>Nanjing Agricultural University<br>1 Weigang, 210095, Nanjing, China<br>Fax: 86 25 84396790<br>Email: xiongaisheng@njau.edu.cn<br><br>-----------------------------<br>Editor's comments:<br>We suggest you find a copy-editing company or friendly native English speaker to polish the grammar.<br>Response:<br>--We thank the editor and the reviewers for this suggestion.<br>--As suggested by the editor and both reviewers, the manuscript has been carefully checked and the language of the manuscript has been improved by the Wiley editing services. All the errors have been corrected. (See revised manuscript, English editing certificate)<br><br>-----------------------------<br>Reviewers' comments:<br>Reviewer #1:<br>Major Concerns:<br>1. Looks very interesting. I'd be happy to review if once the english language is readable.<br>Response:<br>--We thank the reviewer for this suggestion.<br>--As suggested by the reviewer, the manuscript has been carefully checked and the language of the manuscript has been improved by the Wiley editing services. All the errors have been corrected. (See revised manuscript, English editing certificate)

Powered by Editorial Manager® and ProduXion Manager® from Aries Systems Corporation

Reviewer #2:
Major Concerns:
1. A recent triplication event has been identified in tomato genome (2012, Nature). However, no recent WGD event has been detected in the 'VF36' tomato genome.
Response:
--We thank the reviewer for this suggestion.
--As the reviewer mentioned, there was a triplication event in the tomato genome, which occurred in the Solanum lineage. In this study, our results indicate that the 'VF36' tomato genome does not exhibit species-specific WGD. The ambiguity in our original phrasing led to a misunderstanding. We have revised this sentence. Please see lines 32-35 and 250-252.

Lines 32-35: Through comparative genomics and phylogenetic analysis, we identified structural variations (SVs) between the 'VF36' and 'Heinz 1706' genomes and found no evidence of a recent species-specific whole-genome duplication (WGD) in the 'VF36' tomato.

Lines 250-252: Taken together, these results indicated that the recent species-specific whole-genome duplication (WGD) event did not occur in the 'VF36' tomato.

2. Line51, delete 'is' before 'usually regulated'. Line188, remove 'Furthermore,'.
Response:
--We thank the reviewer for this suggestion.
--We have deleted 'is' before 'usually regulated'. We have removed 'Furthermore' in Line 184. Please see lines 51-52 and 187-190.

Lines 51-52: Flowering is essential for the transition of plants from vegetative to reproductive growth and usually regulated by day-length (or photoperiod).

Lines 187-190: In addition, BUSCO analysis showed that an average of 98.3% of single-copy genes were completely assembled in the 'VF36' genome, which was slightly higher than that in SLT1.0 (97.7%) and SL5.0 (96.2%).

3. Line118, A gap-free MicroTOM genome has been reported in Plant Biotechnology in 2024. I suggest that authors provide information on MircroTOM in whole genome comparison. This will contribute to a more comprehensive understanding of differences among tomato genomes.
Response:
--We thank the reviewer for this suggestion.
--We have added the comparative genomics analysis between the 'MicroTom' and the 'VF36' genome. Please see lines 180-192.

Lines180-192: In comparison to the 'Heinz 1706' and the 'MicroTom' tomato genome assemblies, the 'VF36' genome assembly displayed a greater length than the 'Heinz 1706' but was shorter than the 'MicroTom' (Table 1). Notably, 12 T2T chromosomes with 12 centromeric regions were predicted in the 'VF36' genome, whereas no telomeres or centromeric regions were identified in the 'Heinz 1706' (SLT1.0 and SL5.0) and 'MicroTom' (SLM_r2.0) genome assemblies. The SLM_r2.0 genome had 16,700 gaps, SLT1.0 genome version had 210 gaps, and even the substantially more complete SL5.0 version still had 31 gaps, whereas no gaps remained in the 'VF36' genome. In addition, BUSCO analysis showed that an average of 98.3% of single-copy genes were completely assembled in the 'VF36' genome, which was slightly higher than that in SLT1.0 (97.7%) and SL5.0 (96.2%). Taken together, the 'VF36' genome assembly demonstrated higher completeness and accuracy than the 'Heinz 1706' and the 'MicroTom' assemblies.

4. Authors detected 34783 genes in the 'VF36' genome, while 36648 genes were found in SL5.0. I wonder why less gene number in the 'VF36' genome that had a more completeness, continuous genome.
Response:
--We thank the reviewer for this suggestion.

Powered by Editorial Manager® and ProduXion Manager® from Aries Systems Corporation

--The observed discrepancy in gene numbers between the ‘VF36’ and SL5.0 genomes may be attributed to the annotation process rather than being solely dependent on completeness and continuity of the genome assembly. Several factors could underlie this variation: Differences in the gene annotation methods may lead to variations in the number of detected genes. Higher assembly quality can sometimes lead to the merging of fragmented gene models, resulting in a more accurate representation of gene structures but potentially fewer overall gene counts, like overlapping genes or transposable element-related pseudogenes. Natural genomic variation between cultivars (e.g., gene loss, segmental duplications) can contribute to gene count differences.
We have updated the discussion section to describe the discrepancy in gene numbers between the ‘VF36’ and SL5.0 genomes. Please see lines 342-350.

Lines 342-350: The ‘VF36’ displayed more completeness and continuity of the genome assembly than ‘Heinz 1706’, despite the annotation of a greater number of genes in SL5.0 version. This discrepancy might be attributed to differences in gene annotation methods, which could lead to variations in the number of detected genes. Higher assembly quality could sometimes result in the merging of fragmented gene models, thereby yielding a more accurate depiction of gene structures but potentially reducing the overall gene count [40]. Additionally, natural genomic variation between cultivars, such as gene loss or segmental duplications, could also contribute to differences in gene counts [41].

5. Line207, among these SV regions, authors should present the important features, such as functional genes or traits, between the 'VF36' and 'Heinz 1706' genomes. I suggest that authors delete the results of the GO enrichment analysis.
Response:
--We thank the reviewer for this insightful suggestion.
--We agree that highlighting important features such as functional genes or traits within the SV regions is crucial for understanding the differences between the ‘VF36’ and the ‘Heinz 1706’ genomes. We acknowledge that while GO enrichment analysis cannot directly provide the key features within the SV regions, it does provide a broad functional overview of the gene involved. We have revised Line 207 and the surrounding text to better reflect these important features of the genomic differences between the ‘VF36’ and the ‘Heinz 1706’. Please see lines 208-212 and Table S13.

Lines 208-212: Among the identified SV regions, a total of 259 genes were functionally annotated. These genes were involved in a variety of biological processes, including metabolism, environmental information processing, and genetic information processing (Table S13). GO enrichment analysis of the genes in the SV regions indicated that the enriched terms were immune response, DNA integration, and metal ion binding (Figure 2D).

Supplementary Table13. The influenced genes by structure variations between VF36 and Heinz1706 genomes.

6. A total of 289116 SNPs were identified between the 'VF36' and 'Heinz 1706' genomes. The Ks value could be estimated to infer the divergence time, providing additional evidence for the 2.1 Mya obtained from gene family analysis.
Response:
--We thank the reviewer for this suggestion.
-- As the reviewer’s suggestion, we have estimated the divergence time between the ‘VF36’ and the ‘Heinz 1706’ tomato genomes by analyzing the SNPs identified between these two genomic sequences. Please see lines 226-231 and Figure S5.

Lines 226-231: To provide additional evidence, we analyzed the synonymous substitutions per synonymous site (K_S) of collinear homologous genes between the two tomato varieties by analyzing SNP-identified genomic regions. It showed a K_S peak of approximately 0.005 between the genome of the ‘VF36’ and the ‘Heinz 1706’ (Figure S5). Using the formula T=K_S/2r, we estimated the divergence time between the two tomato varieties to be approximately at 1.55 Mya.

Powered by Editorial Manager® and ProduXion Manager® from Aries Systems Corporation

Figure S5 K_S distribution from putative collinear homologous genes between the 'VF36' and the 'Heinz 1706' tomato.

7. Are the sequences of SlPRR1 different between 'VF36' and other genomes? Also, it is worth exploring whether gene expansion, contraction, or specific gene family?
Response:
--We thank the reviewer for this suggestion.
--We have conducted a detailed sequence comparison of the SlPRR1 between the 'VF36' and the 'Heinz 1706'. We observed the two sequences exhibited very few variations. We identified a non-synonymous substitution at position 1074, resulting in an amino acid alteration, and a synonymous substitution at position 1608 located in the C-terminal regulatory region. Please see lines 258-262 and Figure S10.

Lines 258-262: We have conducted a sequence comparison of the SlPRR1 between the 'VF36' and the 'Heinz 1706', it exhibited very few variations. We identified a non-synonymous substitution at position 1074, resulting in an amino acid alteration, and a synonymous substitution at position 1608 located in the C-terminal regulatory region (Figure S10).

Figure S10 Sequences alignment of SlPRR1 from the 'VF36' and the 'Heinz 1706' tomatoes.

8. LIN5, SWEET genes are important factors affecting sugar content. Authors may consider providing the expression levels of these genes in the ppr1 mutant lines.
Response:
--We thank the reviewer for this suggestion.
--We have provided the expression analysis of LIN5, SUT1, and SWEET genes in the slprr1 mutant lines. Please see lines 302-312 and Figure S11.

Lines 302-312: LIN5, a tomato cell-wall-invertase gene (CWIN) was mapped to a major quantitative trait locus (QTL) determining fruit sugar level [39]. Additionally, SUCROSE TRANSPORTER 1 (SUT1) gene, which was responsible for loading and transporting sucrose from source-to-sink organs, encoding an enzyme involved in tomato sucrose metabolism [40]. The expression levels of both LIN5 and SUT1 were significantly higher in slprr1 mutant lines compared to WT lines under LD condition (Figure S11A-S11B). Sugar will eventually be exported transporters (SWEETs) have been verified to mediate sugar transport, with subfamily III members being preferentially explored to transport sucrose in tomato [41]. We surveyed SWEETs of subfamily III in tomato, the expression of SWEET10b, 11a, 11c, and 12a increased in slprr1 mutations under LD condition (Figure S11C-S11L).

Figure S11 Relative expression levels of LIN5, SUT1, and SWEET genes in tomato.

9. What is the relationship among these genes regulating leaf chlorophyll content and sugar content in the prr1 mutant? Further analysis or experiments are needed to clarify this relationship.
Response:
--We thank the reviewer for this suggestion.
--PRR1, a core member of the circadian oscillator in plant, has been shown to regulate the phase and amplitude of circadian rhythms. Disruption of PRR1 function (prr1 mutants) results in circadian arrhythmia, which manifests of chlorophyll homeostasis, attenuated biosynthesis, and delayed degradation. Concurrently, this circadian perturbation alters carbon partitioning, leading to aberrant sucrose accumulation in source leaves and reduced phloem loading efficiency, likely through misregulation of sucrose transporters. While the experiments are indeed an important approach to elucidate the regulatory relationship between circadian clock genes and chlorophyll metabolism as well as sugar transport, we will focus on them in our future research. As the reviewer's suggestion, we have added that to Discussion section. Please see lines 389-395.

Powered by Editorial Manager® and ProduXion Manager® from Aries Systems Corporation

	Lines 389-395: PRR1, a core member of the circadian oscillator in plant, has been shown to regulate the phase and amplitude of circadian rhythms. Disruption of PRR1 function (prr1 mutants) results in circadian arrhythmia, which manifests of chlorophyll homeostasis, attenuated biosynthesis, and delayed degradation. Concurrently, this circadian perturbation alters carbon partitioning, leading to aberrant sucrose accumulation in source leaves and reduced phloem loading efficiency, likely through mis-regulation of sucrose transporters [51].<br><br>Reviewer #3:<br>I was unable to locate the data or any general information about the project. Perhaps the data are under embargo? I kindly ask the authors to clarify this or to indicate the correct location. On a related note, the paper does not specify when the genome assembly and annotation will be made publicly available. Given GigaScience journal's policy on data availability, I believe it is important for the authors to address these points.<br>Response:<br>--We thank the reviewer for this suggestion.<br>--We have double checked and revised the 'Availability of data and materials' section. Please see lines 571-574.<br><br>Lines 571-574:<br>Availability of data and materials<br>Genome Data NCBI BioProject number: PRJNA1204391, BioSample accession: SAMN46040925. All additional supporting data are available in the GigaScience repository, GigaDB.
**Additional Information:**	
**Question**	**Response**
Are you submitting this manuscript to a special series or article collection?	No
**Experimental design and statistics**<br><br>Full details of the experimental design and statistical methods used should be given in the Methods section, as detailed in our Minimum Standards Reporting Checklist. Information essential to interpreting the data presented should be made available in the figure legends.<br><br>Have you included all the information requested in your manuscript?	Yes
**Resources**<br><br>A description of all resources used, including antibodies, cell lines, animals and software tools, with enough information to allow them to be uniquely identified, should be included in the Methods section. Authors are strongly encouraged to cite Research Resource	Yes

Identifiers (RRIDs) for antibodies, model organisms and tools, where possible.<br><br>Have you included the information requested as detailed in our Minimum Standards Reporting Checklist?	
**Availability of data and materials**<br><br>All datasets and code on which the conclusions of the paper rely must be either included in your submission or deposited in publicly available repositories (where available and ethically appropriate), referencing such data using a unique identifier in the references and in the “Availability of Data and Materials” section of your manuscript.<br><br>Have you have met the above requirement as detailed in our Minimum Standards Reporting Checklist?	Yes
GigaScience has policies and guidelines in place for the use of generative AI-writing tools such as ChatGPT. If you have used such writing tools to assist with writing the manuscript this must be declared and cited in the text. Authors should not list AI-writing tools and other AI-assisted technologies as an author or co-author and should acknowledge that they are fully responsible for text generated or refined by AI-writing tools.<p><br><br>A summary of use (particularly in the introduction or among methods) needs to be included at the end of the paper, and the outputs should also be included as a supplementary file hosted in GigaDB or other open repositories. Please read our guidelines for more information. <p><br><br>By submitting to GigaScience, you are aware of the journal's AI-writing tools policy, and if you have declared use of such tools below, you have acknowledged this where appropriate in your manuscript and have made a summary of use and outputs available. <p>	No

Powered by Editorial Manager® and ProduXion Manager® from Aries Systems Corporation

<b>AI-assisted writing tools have been used in the preparation of this manuscript?	

Powered by Editorial Manager® and ProduXion Manager® from Aries Systems Corporation

Manuscript

Click here to access/download;Manuscript;Manuscript-Apr 23.docx

A telomere-to-telomere gapless genome reveals SlPRR1 control of circadian rhythm and photoperiodic flowering in tomato

Hui Liu[1,#], Jia-Qi Zhang[1,#], Jian-Ping Tao[1], Chen Chen[1], Li-Yao Su[1], Jin-Song Xiong[1], Ai-Sheng Xiong[1,*]

[1] *State Key Laboratory of Crop Genetics & Germplasm Enhancement and Utilization, Ministry of Agriculture and Rural Affairs Key Laboratory of Biology and Germplasm Enhancement of Horticultural Crops in East China, College of Horticulture, Nanjing Agricultural University, Nanjing, Jiangsu 210095, China*

[#]These authors contributed equally to this work.

*Please address all correspondence to: Ai-Sheng Xiong (xiongaisheng@njau.edu.cn)

Dr. Ai-Sheng Xiong,

Professor,

State Key Laboratory of Crop Genetics & Germplasm Enhancement and Utilization,

College of Horticulture,

Nanjing Agricultural University,

1 Weigang, 210095, Nanjing, China

Email: xiongaisheng@njau.edu.cn

Hui Liu [0000-0002-0415-722X]; Jia-Qi Zhang [0009-0005-6527-7478]; Chen Chen [0009-0004-8438-8563]; Li-Yao Su [0000-0001-8071-4158]; Jin-Song Xiong [0000-0002-0209-8596]; Ai-Sheng Xiong [0000-0002-7900-5001];

Abstract

Cultivated tomato (*Solanum lycopersicum*) is a major vegetable crop of high economic values that serves as an important model for studying flowering time in day-neutral (ND) plants. A complete, continuous, and gapless genome of cultivated tomato is essential for genetic research and breeding programs. Here, we report the construction of a telomere-to-telomere (T2T) gap-free genome of *S. lycopersicum* cv. VF36 using a combination of sequencing technologies. The 815.27 Mb T2T 'VF36' genome contained 600.23 Mb of transposable elements (TEs). Through comparative genomics and phylogenetic analysis, we identified structural variations (SVs) between the 'VF36' and 'Heinz 1706' genomes and found no evidence of a recent species-specific whole-genome duplication (WGD) in the 'VF36' tomato. Furthermore, a core circadian oscillator, SlPRR1, was identified, which peaked at night in a circadian rhythm. CRISPR/Cas9-mediated knockdown of *SlPRR1* in tomatoes demonstrated that *slprr1* mutant lines exhibited significantly earlier flowering under long-day (LD) condition than wild type (WT). We present a hypothetical model of how SlPRR1 regulates flowering time and chlorophyll biosynthesis in response to photoperiod. This T2T genomic resources will accelerate the genetic improvement of large-fruited tomatoes, and the SlPRR1-related hypothetical model will enhance our understanding of the photoperiodic response in cultivated tomatoes, revealing a regulatory mechanism for manipulating flowering time.

Key Words: Cultivated tomato T2T genome; Photoperiod; Flowering time; Chlorophyll biosynthesis; SlPRR1

Introduction

Flowering is essential for the transition of plants from vegetative to reproductive growth and usually regulated by day-length (or photoperiod). Long-day (LD) plants flower when the number of daylight hours exceeds a critical value, whereas short-day (SD) plants exhibit the opposite effect [1, 2]. In the LD plant *Arabidopsis thaliana*, the major photoperiodic flowering regulator *CONSTANS* (*CO*) peaks before dusk on long days in response to light and the circadian clock [3]. CO promotes flowering by activating the expression of *FLOWERING LOCUS T* (*FT*) and *SUPPRESSOR OF OVEREXPRESSION OF CO1* (*SOC1*) [4]. Photoperiod responses rely on crosstalk between light perception and the circadian clock, which together control the expression of the flowering hormone florigen [5]. The florigen gene *FT* is induced by the CO protein in the light [6, 7]. In contrast, tomato (*Solanum lycopersicum*, 2n = 24; NCBI:txid4081) is a day-neutral (ND) plant that flowers regardless of the day-length. Tomatoes are an important cash crop cultivated worldwide. It is characterized by a sympodial growth habit with scorpioid cymose inflorescence [8]. Flowering time in tomatoes is regulated by several genes and is an important factor for tomato adaptability and genetic improvement. *EARLY FLOWERING* (*ELF*) has been characterized to develop flowers much earlier than parental controls [8]. Several classical genes involved in the control of flowering time in tomatoes, such as *SINGLE FLOWER TRUSS* (*SFT*), *JOINTLESS* (*J*), and *FALSIFLORA* (*FA*), promote flowering, whereas *SELF PRUNING 5G* (*SP5G*) and *TERMINATING FLOWER* (*TMF*) delay flowering [9]. *SP5G*, an *FT* paralog, contributes to the loss of day-length-sensitive flowering by reducing the LD response in tomato cultivars [5].

Metabolic day-length measurement systems have been reported to rely on the

circadian clock-controlled balance in a photoperiodic manner [10]. The circadian clock is a molecular timing device that regulates various physiological and developmental processes via endogenous rhythm [11]. Flowering time is a phenological event regulated by clock output pathways [5]. *EARLY FLOWERING 3* (*ELF3*) is a circadian clock-associated gene that rhythmically inhibits the activity of the light input pathways around dusk by reducing clock sensitivity to light during this phase. It acts as a transcriptional regulator that controls the period of flowering time [12]. Mutations in *ELF3* cause early flowering, possibly because of the increased accumulation of *CONSTANS* (*CO*) transcripts and arrhythmic expression of the morning-specific clock-regulated gene *CHLOROPHYLL A/B BINDING 2* (*CAB2*) and the oscillator component *LATE ELONGATED HYPOCOTYL* (*LHY*) [13]. The *PSEUDO RESPONSE REGULATOR1* (*PRR1*) gene, also known as *TIMING OF CAB EXPRESSION1* (*TOC1*), belongs to the PRR family and was the first member discovered to play a central role in the regulation of circadian rhythms [14, 15]. *PRR1* mutants exhibit a faster-running clock, and misexpression of *PRR1* can lead to hypocotyl growth and early flowering [16]. PRR1 represses *LHY* and *CIRCADIAN CLOCK ASSOCIATED1* (*CCA1*) expression in *Arabidopsis* by directly binding to their promoters. However, the flowering regulatory pathways associated with the circadian clock oscillator are less well-studied in cultivated tomatoes than in *Arabidopsis*.

Chlorophyll (Chl) plays a central role in harvesting light and transforming it into chemical energy via through photosynthesis during tomato fruit development [17, 18]. Chl is the primary photosynthetically active pigments in plants. Chl metabolism in tomato leaves contributes to photosynthesis [19], and Chl content modulates many metabolic processes that affect fruit quality of fruit. However, it remains unclear which enzymes degrade Chl in the tomato leaf photosystems under LD and SD conditions.

Chl synthesis involves four main steps: 5-aminoleculinic acid (ALA), protoporphyrin IX, chlorophyll a (Chl a), and chlorophyll b (Chl b) synthesis. Previous studies have reported that *SlCHLH* and *SlCHLI* are important for Chl accumulation in tomato leaves [20]. Transcription factors such as GOLDEN2-LIKE 1 and 2 (GLK1 and 2) have been reported in many species, and their overexpression increases chlorophyll content and can lead to chloroplast development in tissues [21].

Cultivated tomatoes (*Solanum lycopersicum*) are distributed worldwide and are economically significant in the vegetable industry. Tomatoes are good model for horticultural plants, especially for studying fleshy fruit biology [22]. Although several tomato reference genomes have been published [23, 24], a high-accuracy reference genome has reported 31 gaps in SL5.0 [25]. However, a gapless reference genome is unavailable for the large-fruited tomato. Furthermore, centromeres, which are essential for maintaining chromosomal integrity during cell division and ensuring the fidelity of inheritance, remain largely underexplored in plants [26]. Recently, several horticultural species, including carrot, grape, kiwifruit, lemon, and strawberry, have been sequenced with telomere-to-telomere (T2T) assemblies using PacBio high-fidelity (HiFi), Oxford Nanopore Technology (ONT) ultra-long, and high-throughput chromosome conformation capture (Hi-C) technology [27-31]. However, T2T gap-free genomes have not been reported in large-fruited tomatoes.

To improve the completeness of the cultivated tomato reference genome, we assembled a T2T gap-free genome sequence for large-fruited tomato ‘VF36’ using a combination of PacBio-HiFi, ONT ultra-long, and Hi-C technologies. ‘VF36’ tomato is an important variety [32], and it remains unclear whether the core circadian oscillator SlPRR1 regulates flowering time and chlorophyll biosynthesis during different photoperiods. In the present study, we demonstrated that the knockdown of *SlPRR1* in

tomatoes caused early flowering under LD conditions and delayed flowering under SD conditions. In this study, we proposed a hypothetical model for the *SlPRR1* regulation of flowering time and chlorophyll biosynthesis in response photoperiod.

Results

A T2T gap-free tomato reference genome for 'VF36'

Based on *K*-mer analysis with 48.86 Gb Illumina reads, the genome size of 'VF36' tomato was estimated to be 774.22 Mb, with a heterozygosity rate of 0.71% and a duplication ratio of 38.84% (Figure S1-S2 and Table S1).

To develop a high-quality genome assembly for the 'VF36' tomato, different sequencing platforms were employed. A total of 62 Gb of PacBio HiFi reads and 169.51 Gb of ONT ultra-long reads were generated to pre-assemble the genome. The N_{50} length of the HiFi reads was 17.74 kb, and the N_{50} length of the ONT reads was 55.07 kb (Table S2). NextDenovo was used to assemble the ONT data, forming 26 contigs with an N_{50} size of 55.68 Mb. For the PacBio HiFi combined with ONT reads, a genome with a contig N_{50} size of 68.44 Mb was assembled (Table S3), an increase of approximately 1.6-fold compared with the previous build SL5.0 [25] and 3.8- fold compared with the 'Heinz 1706' build SLT1.0 [33]. Moreover, a total of 812.37 Mb Hi-C reads were anchored into 12 pseudochromosomes by assistance with assembly correction (Figure 1A and Figure S3). After filling all remaining gaps, a gap-less reference genome of 'VF36' was generated, with a total length of 815,269,421 bp (Table 1 and Table S4).

Using telomeric repeats (CCCTAAA at the 5' end or TTTAGGG at the 3' end) as sequence queries, we identified 24 telomeric regions among the 12 chromosomes. Plant centromeres typically have a high tandem repeats (TRs) density and low gene density.

Based on this sequence structure, we identified nine regions of the tandem repeat clusters (TRCs) on each chromosome that were continuous and occupied the majority of the chromosome (Figure S4). In total, 12 centromeric regions were estimated, with lengths ranging from 0.48 to 3.63 Mb (Table S5).

Finally, a gap-free 'VF36' tomato genome consisting of 12 T2T chromosomes with 12 centromeric regions was generated (Figure 1C). BUSCO assessment indicated that 1586 of the core conserved plant genes (98.27% of 1614 BUSCOs) were complete in the 'VF36' tomato assembly. We assessed the base accuracy of the genome using *k*-mer quality estimation, and the quality values (QV) ranged from 41.82 to 62.72 for each chromosome. The long terminal repeat (LTR) assembly index (LAI) was used to evaluate genome assembly continuity, which was found to be 12.71. These results indicated the completeness, continuity, and accuracy of the genome assembly.

'VF36' tomato genome annotation

A total of 600,225,913 bp transposable elements (TEs) were identified, accounting for 73.28% of the assembled 'VF36' tomato genome, with LTR elements being the major component at 429.64 Mb. Gypsy-type LTRs (246.25 Mb) were much more abundant than Copia-type LTRs (68.01 Mb) (Figure 1B and Table S6). Moreover, we predicted protein-coding genes from the assembly, resulting in 'VF36' tomato annotation with 34,783 genes using a combination of *ab initio*, homology-based, and transcriptome-based search methods. The average mRNA length was 5.12 kb with 4.8 exons per gene in the 'VF36' genome (Table S7). The BUSCO assessment indicated that the completeness of the gene set in the annotated genome was 98.2% (Table S8).

In total, 33,540 genes (96.43%) were functionally annotated using the National Center for Biotechnology Information non-redundant (NR), UniProt, InterPro, Pfam,

Gene Ontology (GO), and Kyoto Encyclopedia of Genes and Genomes (KEGG) databases (Figure 1D and Table S9). We identified 413 micro RNAs (miRNAs), 1,049 transfer RNAs (tRNAs), 2,938 ribosomal RNAs (rRNAs), and 582 small nuclear RNAs (snRNAs) were found in the 'VF36' tomato genome (Table S10).

Global comparison of 'VF36' and 'Heinz 1706' genomes

In comparison to the 'Heinz 1706' and the 'MicroTom' tomato genome assemblies, the 'VF36' genome assembly displayed a greater length than the 'Heinz 1706' but was shorter than the 'MicroTom' (Table 1) [34]. Notably, 12 T2T chromosomes with 12 centromeric regions were predicted in the 'VF36' genome, whereas no telomeres or centromeric regions were identified in the 'Heinz 1706' (SLT1.0 and SL5.0) and 'MicroTom' (SLM_r2.0) genome assemblies. The SLM_r2.0 genome had 16,700 gaps, SLT1.0 genome version had 210 gaps, and even the substantially more complete SL5.0 version still had 31 gaps, whereas no gaps remained in the 'VF36' genome. In addition, BUSCO analysis showed that an average of 98.3% of single-copy genes were completely assembled in the 'VF36' genome, which was slightly higher than that in SLT1.0 (97.7%) and SL5.0 (96.2%). Taken together, the 'VF36' genome assembly demonstrated higher completeness and accuracy than the 'Heinz 1706' and the 'MicroTom' assemblies.

Collinearity analysis between the 'VF36' and 'Heinz 1706' genomes revealed that 99.72% of the 'Heinz 1706' genome could be mapped to the 'VF36' genome (Figure 2A). We performed single nucleotide variation (SNP) and short insertion/deletion (InDel) analyses, which identified 289,116 SNPs and 103,826 InDels through a comparison of the 'VF36' and 'Heinz 1706' assemblies. Most SNPs (210,357) and InDels (74,011) were located in the intergenic regions (Table S11). Abundant genetic

variations, particularly structural variations (SVs), was detected between the two genomes. We predicted 1,807 SVs distributed throughout the genome, with deletions (DELs) and insertions (INSs) accounting for 817 and 705 events, respectively. These DELs and INSs affected 587 functional genes and contributed to the divergence of these two accessions. Furthermore, 60.55% of the SVs (1,076) were located in Intergenic regions, whereas only 4.76% (86) were located in the coding regions. Among these SVs, two large inversions (INVs) (> 400 kb) were observed between the two genomes. INV299, with a length of 2.78 Mb (chr02: 37,961,912-40,734,631), was predicted between 'VF36' and 'Heinz 1706' and was annotated as pectate lyase (Table S12). Among the identified SV regions, a total of 259 genes were functionally annotated. These genes were involved in a variety of biological processes, including metabolism, environmental information processing, and genetic information processing (Table S13). GO enrichment analysis of the genes in the SV regions indicated that the enriched terms were immune response, DNA integration, and metal ion binding (Figure 2D).

Phylogenetic analysis

The identification of homologous genes was critical for 'VF36' tomato evolutionary analysis. We predicted homologous genes among 13 genomes, including 'VF36', 'Heinz 1706', *Solanum pimpinellifolium*, *Solanum pennellii*, *Solanum tuberosum*, *Solanum melongena*, *Capsicum annuum*, *Nicotiana benthamiana*, *Petunia axillaris*, *Cuscuta campestris*, *Daucus carota*, *Vitis vinifera*, and the outgroup *Oryza sativa* (Table S14). As expected, 'VF36' clustered together with 'Heinz 1706' (Figure 2B). A total of 527,829 homologous genes were identified and classified into 86,939 gene families. Among these genes, 195,190 were distributed among the 7,593 gene families shared by all 13 genomes (Table S15). Based on 52 characterized single-copy and low-

copy gene families, we constructed a phylogenetic tree with divergence times. Our findings inferred that ‘VF36’ diverged from ‘Heinz 1706’ tomato 2.1 million years ago (Mya). To provide additional evidence, we analyzed the synonymous substitutions per synonymous site (K_S) between collinear homologous genes between the two tomato varieties by analyzing SNP-identified genomic regions. It showed a K_S peak of approximately 0.005 between the genome of the ‘VF36’ and the ‘Heinz 1706’ (Figure S5). Using the formula $T=K_S/2r$, we estimated the divergence time between the two tomato varieties to be approximately at 1.55 Mya. *C. annuum* was the sister group to the *Solanum* species, and the divergence time of the *C. annuum*- *Solanum* lineage was approximately 33.3 Mya (Figure 2B).

Gene family expansion and contraction were examined using CAFE. The expanded and unique gene families in the ‘VF36’ tomato were identified as 456/1,786. GO analysis revealed that these expanded genes were involved in the response to auxin, oxidoreductase activity, photosystem II, and protein serine/threonine/tyrosine kinase activity (Figure S6). Unique genes were enriched in transferase activity, protein kinase activity, and methylation (Figure S7).

The distribution of K_S between collinear homologous genes was determined. We observed a K_S peak of approximately 0.65 in ‘VF36’ tomato, which was also present in other Solanaceae species *S. tuberosum* and *N. benthamiana*, corresponding to the time of the shared family-specific Solanaceae-α hexaploidy event [35] (Table S16). A substantial recent K_S peak at approximately 0.15 was observed in *N. benthamiana*, consistent with previous studies [36, 37] (Figure 3). Furthermore, inter-genomic gene collinearity between *V. vinifera* and the ‘VF36’ tomato was investigated using a 6:1 syntenic depth ratio (Figure S8). A ratio of 2:1 between *N. benthamiana* - ‘VF36’ tomato and *N. benthamiana* - *S. tuberosum* was characterized (Figure S9). The dot plot

of syntenic analysis showed that each fragment in the 'VF36' tomato could be identified with the two most related syntenic fragments in *N. benthamiana*. Taken together, these results indicated that the recent species-specific whole-genome duplication (WGD) event did not occur in the 'VF36' tomato.

A core circadian oscillator SlPRR1 repressed flowering in tomato

Flowering time is a vital trait in the reproductive success of tomato plants. Photoperiod (day-length) regulates plant growth and flowering. PRR1 is a core circadian oscillator essential for plant growth and development. A previous study has shown that SlPRR1 expression has a robust light-dependent circadian rhythm with night-peaking [38]. We have conducted a sequence comparison of the SlPRR1 between the 'VF36' and the 'Heinz 1706', it exhibited very few variations. We identified a non-synonymous substitution at position 1074, resulting in an amino acid alteration, and a synonymous substitution at position 1608 located in the C-terminal regulatory region (Figure S10). In this study, the expression profiles of *SlPRR1* were analyzed using RT-qPCR, which showed that it was highly expressed in roots and fruits (Figure 4A). We observed that the expression of *SlPRR1* peaked at night and exhibited a circadian rhythm (Figure 4B).

To determine the biological role of *SlPRR1* in flowering time, genetic evidence was obtained using the CRISPR/Cas9 gene-editing system. We designed two single-guide RNAs (sgRNAs) targeting the third exon of *SlPRR1* (Figure 5A). Four homozygous mutant lines, designated as *slprr1-35, slprr1-5, slprr1-6,* and *slprr1-10*, were used for further analysis (Figure 5B). To better investigate the role of *SlPRR1* in day-length responses, we measured the flowering time of control plants (WT) and *slprr1* mutant lines under LD and SD conditions. Compared to WT tomatoes, *slprr1* mutant lines showed significantly earlier flowering under LD conditions. In contrast,

the *slprr1* mutant lines showed delayed flowering compared to WT plants under SD conditions (Figure 5C-E).

The *slprr1* mutation caused early flowering under LD conditions and affected the expression of *CO/COL*. We identified 13 *CO/COL* genes in tomatoes, the expression of which increased in *slprr1* mutations, except for *SlCO1*, *SlCOL10b*, and *SlCOL16b* (Figure 8K). COs are thought to mediate the circadian clock and control flowering [39]. CO promoted flowering by activating *FT* and *SOC1* expression. We surveyed the expression of a group of genes implicated in the control of flowering time in tomatoes, including *FTL1*, *J*, *SFT*, *SP5G*, *BOP*, *TMF*, *SOC1*, and *FA* (Figure 8A-J). Among them, the tomato *SP5G* gene, an *FT* paralog, was significantly more highly expressed in LD than in SD. However, transcription of another *FT* paralog, *FTL1*, decreased under LD conditions. These results indicate that SP5G regulates flowering under LD conditions, whereas FTL1 specifically responds to SD. Furthermore, the expression of *SOC1* was significantly downregulated in the *slprr1* mutants under both LD and SD conditions. The expression of *J* and *FUL2* was significantly different between the *slprr1* mutant and WT plants. These data suggest a model for the regulation of flowering time under LD or SD conditions and provided evidence that the core circadian clock gene, *SlPRR1*, regulates flowering time in tomatoes (Figure 9).

Knockout of *SlPRR1* affected sugar accumulation in tomato fruit

The sugar content of red ripening fruits, which plays a decisive role in tomato quality, was analyzed in WT and gene-edited fruits under LD and SD conditions [24]. The *slprr1* mutant fruits showed significantly lower fructose and glucose contents under both LD and SD conditions. The sucrose content was significantly higher in *slprr1* mutant fruits than in WT fruits under both LD and SD conditions, although the sucrose

content was much lower than that of fructose and glucose in the tomato fruits (Figure 5F). These results indicated that the knockout of *SlPRR1* promoted flowering under LD conditions and affected fruit flavor in tomatoes.

LIN5, a tomato *cell-wall-invertase* gene (*CWIN*) was mapped to a major quantitative trait locus (QTL) determining fruit sugar level [40]. Additionally, *SUCROSE TRANSPORTER 1* (*SUT1*) gene, which was responsible for loading and transporting sucrose from source-to-sink organs, encoding an enzyme involved in tomato sucrose metabolism [41]. The expression levels of both *LIN5* and *SUT1* were significantly higher in *slprr1* mutant lines compared to WT lines under LD condition (Figure S11A-S11B). Sugar will eventually be exported transporters (SWEETs) have been verified to mediate sugar transport, with subfamily III members being preferentially explored to transport sucrose in tomato [42]. We surveyed *SWEET*s of subfamily III in tomato, the expression of *SWEET10b*, *11a*, *11c*, and *12a* increased in *slprr1* mutations under LD condition (Figure S11C-S11L).

Proposed Chlorophyll biosynthesis pathway in tomato leaves

To explore the chlorophyll biosynthesis pathway in tomato leaves, we measured the chlorophyll content in WT and gene-edited plants under LD and SD conditions. Compared to both WT and gene-edited plants grown under SD conditions, tomato leaves exhibited significantly higher chlorophyll contents under LD conditions. The *slprr1* mutants showed slightly lower chlorophyll content than WT plants under both LD and SD conditions.

The chlorophyll biosynthesis pathway was elucidated, and the enzymes genes were surveyed (Figure 6A). Among these genes, the *SlGluTR_1*, *SlPPO*, *SlPOR1*, and *SlPOR2* were expressed at significantly higher levels in WT plants under LD conditions

according to transcriptome analysis. To infer the transcription factors (TFs) that modulate the transcription of genes related to chlorophyll metabolism in tomatoes, we analyzed the expression of *SlH2A* and *SlNDF5* (upregulated) and *SlGLK2*, *SlLHCB*, and *SlpsaH* (downregulated) under LD conditions (Figure 7A-K). This suggests that *SlH2A* and *SlNDF5* positively regulate the activity of candidate enzymes, whereas *SlGLK2*, *SlLHCB*, and *SlpsaH* negatively regulated SlGluTR_1, SlPPO, SlPOR1, and SlPOR2 under LD conditions (Figure 9).

Discussion

A high-quality reference genome is indispensable for identifying traits and facilitating genetic improvements. In this study, we publicly released a T2T gap-free genome of cultivated tomato 'VF36', comprising 815.27 Mb of sequence and 34,783 protein-coding genes. The complete genome sequence of cultivated tomato is the largest reported to date, surpassing several previously released tomato reference genomes [23-25, 43]. The combination of ONT ultra-long, PacBio-HiFi, and Hi-C sequencing technologies has overcome the assembly challenges, including the 31 gaps remaining in the SL5.0 version and centromeres. In our cultivated tomato 'VF36', we have successfully corrected numerous misassembles and filled all chromosomal gaps. The 'VF36' displayed more completeness and continuity of the genome assembly than 'Heinz 1706', despite the annotation of a greater number of genes in SL5.0 version. This discrepancy might be attributed to differences in gene annotation methods, which could lead to variations in the number of detected genes. Higher assembly quality could sometimes result in the merging of fragmented gene models, thereby yielding a more accurate depiction of gene structures but potentially reducing the overall gene count

[44]. Additionally, natural genomic variation between cultivars, such as gene loss or segmental duplications, could also contribute to differences in gene counts [45]. Comparison of SVs present in ‘VF36’ and ‘Heinz 1706’ revealed several regions associated with the immune response, DNA integration, and metal ion binding.

Modern cultivated tomatoes are derived from the wild relative, *Solanum pimpinellifolium*. Wild tomatoes are recognized as SD plants, whereas most cultivated accessions have reduced photoperiodic sensitivity and are considered ND plants [9]. The selection of flowering time has been a major goal in tomato breeding efforts, which have spread cultivars worldwide from their origins. In this study, the core circadian oscillator SlPRR1 exhibited a circadian rhythm with peak expression at night, consistent with previous studies [46]. SlPRR1 was highly expressed in roots during vegetative growth when the tissues were in a dark environment.

The clock ran faster in *slprr1* mutants under LD conditions, whereas the *slprr1*1 mutants exhibited delayed flowering under SD conditions. These results indicate that SlPRR1 regulates flowering time in cultivated tomato plants. CO is a circadian clock-regulated gene that encodes a transcription factor required for flowering [47], which is modulated by day-length. The number of 13 *CO*/*COL* genes were identified in cultivated tomatoes, most of which were differentially expressed between the LD and SD conditions. A previous study suggested that *SlCOL*, *SlCOL4a*, and *SlCOL4b* may function as positive regulators of tomato flowering [48]. *CO* and *FT* are the two central integrators of the photoperiod pathway that control flowering time, with FT members being the final outputs of the photoperiodic response downstream of CO [49]. As previously reported, we observed several florigen genes involved in the regulation of flowering time. *SP5G* was highly expressed under LD conditions, but barely expressed under SD conditions. As an *FT* paralog, *SP5G* is a major locus influencing day-length

adaptation in tomatoes, and tomato cultivars contribute to the loss of daylength-sensitive flowering by reducing their LD responses. Another *FT* paralog, FTL1, does not control LD flowering in tomatoes and responds specifically to SDs [5]. Consistent with our results, *FTL1* was specifically expressed under SD conditions. Therefore, we propose a model for the regulation of flowering time by SlPRR1 knockout in tomatoes cultivated under LD and SD conditions.

Flowering is an important developmental stage and a clear sign of plants transition from the vegetative to the reproductive stage. During the tomato growth cycle, the leaf chlorophyll content changes dynamically through chlorophyll accumulation and degradation. Chlorophyll, the most abundant pigment on Earth, is a key component of photosynthesis and is required for sunlight absorption [50]. Photoperiod regulates plant germination, growth, and flowering, while affects chlorophyll biosynthesis. In addition to photoreceptors, chloroplasts act as plant light sensors in response to different photoperiods by altering their ultrastructures [51]. Few studies have reported on the regulatory mechanisms of chlorophyll biosynthesis in tomato leaves cultivated under different photoperiods. PRR1, a core member of the circadian oscillator in plant, has been shown to regulate the phase and amplitude of circadian rhythms. Disruption of PRR1 function (*prr1* mutants) results in circadian arrhythmia, which manifests of chlorophyll homeostasis, attenuated biosynthesis, and delayed degradation. Concurrently, this circadian perturbation alters carbon partitioning, leading to aberrant sucrose accumulation in source leaves and reduced phloem loading efficiency, likely through mis-regulation of sucrose transporters [52]. Under LD conditions, the chloroplasts of the growing plants exhibited smaller grana stacks and the chlorophyll contents increased significantly. In the present study, tomato leaves exhibited higher chlorophyll content under LD conditions than under SD conditions. Chlorophyll

biosynthesis can be divided into four parts: the formation of 5-aminolevulinic acid, biosynthesis of protoporphyrin IX from eight 5-aminolevulinic acid molecules, and biosynthesis of chlorophyll *a* and *b* in the magnesium branch [53]. Moreover, Chlorophyll biosynthesis is influenced by several genes such as *GLKs*, which play vital roles in regulating chlorophyll accumulation and chloroplast development in tomato fruits [21]. BEL2 may directly bind to the *GLK2* promoter to repress its transcription in tomatoes [54]. In this study, *GLK2* was highly expressed under SD conditions compared with LD conditions. *BEL2* was highly expressed in *slprr1* mutants under LD conditions, showed a different expression pattern than that of *GLK2* in tomatoes. Chlorophyll synthesis and degradation are dynamic and complex processes influenced by environmental factors such as photoperiod and are regulated by multiple enzymes and regulatory genes.

Conclusions

In summary, we present a T2T gap-free genome of the cultivated tomato *var*. VF36 using data from PacBio-HiFi, ONT ultra-long, and Hi-C technologies. We identified and verified a core circadian oscillator, SlPRR1, and observed that *slprr1* mutant lines exhibited significantly early flowering under LD conditions and delayed flowering under SD conditions compared to WT. Based on these findings, we propose a hypothetical model illustrating how SlPRR1 regulates flowering time and chlorophyll biosynthesis in response to photoperiods. The study provides novel insights into the essential regulatory mechanisms of flowering time and offers potential avenues for manipulating and improving tomato yields.

Data Description

Plant materials, growth conditions, and photoperiod treatment

Tomato (*Solanum lycopersicum*) cultivars 'VF36' and 'Micro-Tom' were grown in soil in growth chambers at Nanjing Agricultural University. For flowering time assessment, seeds were sown under a light intensity of 20000 lx (360 μmol m^{-2} s^{-1}) at 25 °C and 70% relative humidity with different photoperiods: LD (16L:8D), SD (8L:16D), and ND (12L:12D). Flowering time was evaluated as the number of days to reach the first observable flower opening, with at least three individual plants used for assessment.

DNA extraction and sequencing

Leaf samples of the 'VF36' inbred line were collected for genome sequencing. DNA extraction was performed using a modified cetyltrimethylammonium bromide (CTAB) method.

The ONT ultra-long library was obtained from the Nanopore sequencing platform (Nanopore, Oxford, UK) and size-selected (>30 kb) using Filtlong (RRID:SCR_024020) (v.2.4) software. The pass reads were then filtered to obtain joint sequences with mean read quality scores above 90% using Porechop (RRID:SCR_016967) (v.2.4). A SMART cell sequencing library containing approximately 15-20 kb fragments was constructed and sequenced using PacBio according to the standard protocol (PacBio, CA, USA) [55].

For Illumina short-read sequencing, libraries were constructed using the Nextera DNA Flex Library Prep Kit (Illumina, San Diego, CA, USA) and Sequenced on an Illumina HiSeq 2000 platform (RRID:SCR_020130). Raw reads were filtered and polished using Fastp software (RRID:SCR_016962) (v.21.0). Simultaneously, a Hi-C

library was established using an Illumina NovaSeq 6000 platform (RRID:SCR_016387) (Illumina, CA, USA), yielding 662,677,798 bp of clean data. Sequencing was performed at the Wuhan Benagen Technology Co., Ltd (Wuhan, China) [56].

Genome initial assembly and assessment

Genome size was estimated based on *k*-mer distribution analysis using Jellyfish (RRID:SCR_005491) (v.2.2.10), and genome heterozygosity was determined using GCE (RRID:SCR_017332) (v.1.0) [57]. After removing low-quality sequences from the ONT ultra-long sequencing data, the initial assembly was performed using NextDenovo (RRID:SCR_025033) (v.2.5) with parameters of read_cutoff =1k, block size = 1 g, nextgraph_options = -a 1. The assembly was corrected for ONT reads using Racon (RRID:SCR_017642) (v.1.4.11) over two rounds, and further improved using Pilon (RRID:SCR_014731) (v.1.23) with second-generation sequencing data. Additionally, a combined assembly strategy using PacBio HiFi reads was used to obtain a high-accuracy assembly. Hifiasm (RRID:SCR_021069) (v.0.16.1-r375) software was used to assemble the genome using PacBio HiFi reads alone and PacBio HiFi reads combined with ONT ultra-long reads [58]. Initial genome assembly completeness was assessed using the embryophyte_odb10 database of 1614 single-copy orthologues in BUSCO (RRID:SCR_015008) (v.5.8.2) [59].

A total of 98.70 Gb Hi-C sequencing data assisted in genome assembly through clustering, ordering, orienting, and eliminating redundancy of the contigs using ALLHiC (RRID:SCR_022750) (v.0.9.8), 3D-DNA (RRID:SCR_017227) (v.180419), and Juicer (RRID:SCR_017226) (v.1.6) software. The gaps were filled with 100 N to obtain the final chromosome-level genome sequence. Finally, the accuracy of the Hi-C-based chromosomal assembly was assessed using the HiCExplorer

(RRID:SCR_022111) (v.3.6) chromatin contact matrix [60].

Missed telomers were further filled using ONT ultra-long reads with Winnowmap (RRID:SCR_025349) (v.1.11), medaka_consensus (v.1.2.1), and Nucmer (v.3.1), as described by Wang [27]. To further improve the scaffold building and fill these gaps, winnowmap (v.1.11) was used with corrected ONT ultra-long reads and HiFi reads. Genome assembly continuity as assessed based on gap location and number. The completeness of the gene regions was evaluated using BUSCO, as described above. The quality value (QV) of the genome assembly was estimated using the *K*-mer database of Illumina short reads.

Genome annotation

Repetitive sequences were identified using homology-based and *de novo* approaches. RepeatModeler (RRID:SCR_015027) (v.2.0.4) and LTR_FINDER were used to build a *de novo* TE library [61]. RepeatMasker (RRID:SCR_012954) (v.4.1.5) was applied to identify TEs from the RepBase TE library combined with the *de novo* TE library [62]. Finally, the non-redundant combined TE sets were generated.

Gene structure annotation was performed using ab initio, homology-based, and transcriptome-based prediction methods. Homologies from five species (*Solanum chmielewskii*, *Solanum galapagense*, *Solanum lycopersicum* var. 'Heinz1706', *Solanum pimpinellifolium*, and *Arabidopsis thaliana*) were collected as protein evidence for the predicted gene sets using Exonerate (RRID:SCR_016088) (v.2.4). The TransDecoder (RRID:SCR_017647) (v.5.7) pipeline was used to assemble RNA-seq reads into the transcripts. Ab initio gene prediction was performed using Augustus (v.3.5.0) and Glimmerhmm (RRID:SCR_002654) (v.3.0.4) [63]. All predictions were integrated into a comprehensive protein-coding gene set using Maker

(RRID:SCR_005309) (v.3.01.03) [64]. Gene function annotation was performed using homology searches against the public databases, including NCBI non-redundant (NR), UniProt, InterPro, Pfam, GO, and KEGG databases.

Non-coding RNAs (ncRNAs) including transfer RNAs (tRNAs), ribosomal RNAs (rRNAs), microRNAs (miRNAs), and small nuclear RNAs (snRNAs), were predicted. The tRNAscan-SE (RRID:SCR_008637) (v.2.0.12) was used to identify tRNAs with default parameters, RNAmmer (RRID:SCR_017075) (v.1.2) was used to search for rRNAs, and INFERNAL (RRID:SCR_011809) (v.1.1.4) was applied to identify miRNAs or snRNAs based on the Rfam database (RRID:SCR_007891) [65].

Identification of centromeres and telomeric sequences

Centromeric regions consist of tandem repeats (TRs), centromeric retrotransposons, and low-copy sequences. For telomere identification, the plant telomere sequences 5'-CCCTAAA-3' was used. Furthermore, TRF software was used to search for TR sequences, and the denser regions of the tandem repeat clusters (TRCs) tended to be in the centromere regions.

Synteny analysis and identification of SNPs, InDels, and SVs

MUMmer was used to perform genomic collinearity analysis between the 'VF36' and 'Heinz 1706' genomes [66]. Single nucleotide variations (SNPs), short insertions/deletions (indels), and structural variations (SVs) were analyzed using SyRI [67]. Annotations were obtained using ANNOVAR software toolkit.

SVs were classified into five types: inversions, translocations, duplications, deletions, and insertions. Based on the overlapping regions in the genome, we calculated the number of SVs in the coding regions, introns, 2 kb upstream, 2 kb

downstream, and intergenic regions. Compared with the 'VF36' genome, the identified genes affected by SVs were subjected to GO and KEGG enrichment analyses.

Phylogenetic, gene family expansion/contraction analysis

To infer the evolutionary history of the 'VF36' tomato, we identified homologs and single-copy orthologous genes using OrthoFinder software in *S. lycopersicum* var. 'Heinz1706', *Solanum pimpinellifolium*, *Solanum pennellii*, *Solanum tuberosum*, *Solanum melongena*, *Capsicum annuum*, *Nicotiana benthamiana*, *Petunia axillaris*, *Cuscuta campestris*, *Daucus carota*, *Vitis vinifera*, and the outgroup *Oryza sativa*. Single-copy genes were aligned using MUSCLE (RRID:SCR_011812) (v3.8.31). A phylogenetic tree was constructed using the maximum likelihood (ML) method in the RAxML software with JTT models for amino acid data [68]. MCMCTREE in PAML was then used to estimate the divergence time [55].

The gene family expansion and contraction were analyzed by computational analysis of gene family evolution (CAFE v.3.1) with default parameters in 'VF36' tomato compared with that in other 12 species [69].

Synteny and whole-genome duplication (WGD) analysis

Syntenic gene pairs between 'VF36' tomato, *S. lycopersicum* var 'Heinz1706', *S. pimpinellifolium*, *S. pennellii*, *S. tuberosum*, *S. melongena*, *C. annuum*, *N. benthamiana*, *P. axillaris*, *C. campestris*, *D. carota*, *V. vinifera*, and *O. sativa* were identified using JCVI (RRID:SCR_021641) (v.0.9.13) [70].

WGDs or polyploidy were prevalent and estimated using synonymous substitutions per synonymous site (K_S) values. K_S estimates for pairwise comparisons (one-to-one orthologs between species) were obtained using the PAML package in

yn00.

CRISPR plasmid construction and stable tomato transformation

For the CRISPR/Cas9 construct, two specific sgRNAs in the exon of the tomato *SlPRR1* gene were designed using the CRISPR-GE online tool (http://skl.scau.edu.cn/targetdesign). Two sgRNAs expression cassettes, combined with two target sites, were driven by *AtU6*, and assembled into the pHSbdcas9i vector [71]. The confirmed constructs were transformed into *Agrobacterium tumefaciens* GV3101. These constructs were introduced into the tomato cultivar Micro-Tom via *A. tumefaciens*-mediated transformation. All primers used are listed in Table S17. Homozygous T_1 transgenic plants were used for phenotypic characterization.

Chlorophyll contents, sugar extraction and measurement

Chlorophyll content was measured using a Plant Nutrition Tester (SPAD 502; Beijing Zhongke Weihe Technology Development Co., Ltd., Beijing, China). Soluble sugars were determined using a High-Performance Liquid Chromatography (HPLC) system (Waters Corp., MA, USA) equipped with an Acquity UPLC BEH amide column and Evaporative Light Scattering detector. Samples were collected from red ripe tomato fruits, immediately frozen in liquid nitrogen, and stored at -80 °C freezer. Soluble sugars were extracted and analyzed as previously described. Briefly, freeze-dried samples were added to 5 ml of distilled water, homogenized for 1 min, and extracted in a water bath at 80 °C for 30 min. The supernatant was collected by centrifugation and filtered through a 0.45 μm membrane to determine the soluble sugar content in the filtrate.

Statistical analysis

Significance tests were performed using Prism 9 (GraphPad, CA, USA) based on Student's *t*-test at P <0.01 or P < 0.05. Data are presented as means ± SDs (standard deviations).

Data Availability

The genome data is deposited in NCBI with BioProject number: PRJNA1204391 and BioSample accession: SAMN46040925. All additional supporting data are available in the *GigaScience* repository, GigaDB [72].

Competing interests

The authors declare that they have no competing interests.

Acknowledgements

This research was supported by National Natural Science Foundation of China (32402552), Natural Science Foundation of Jiangsu (BK20221009), and Key Research and Development Program of Jiangsu (BE2023350). The research was supported by the high-performance computing platform of Bioinformatics Center, Nanjing Agricultural University. We thank Professor Xue-Dong Yang from Shanghai Academy of Agricultural Science for providing the 'VF36' tomato materials.

Authors' contributions

A.-S.X. and H.L. designed and supervised the study. H.L. wrote the manuscript. H.L. and J.-Q.Z. performed the experiments with assistance from J.-P.T., C.C., and L.-Y.S. J.-S.X. and A.-S.X. contributed substantially to revisions. All authors commented on the manuscript.

Reference

1. Andrés F and Coupland G. The genetic basis of flowering responses to seasonal cues. Nature Reviews Genetics. 2012;13 9:627-39.
2. Huang H and Nusinow DA. Into the Evening: Complex Interactions in the Arabidopsis Circadian Clock. Trends in Genetics. 2016;32 10:674-86. doi:10.1016/j.tig.2016.08.002.
3. Song YH, Shim JS, Kinmonth-Schultz HA and Imaizumi T. Photoperiodic Flowering: Time Measurement Mechanisms in Leaves. Annual Review of Plant Biology. 2015;66 1:441-64.
4. Samach A, Onouchi H, Gold SE, Ditta GS, Schwarz-Sommer Z, Yanofsky MF, et al. Distinct Roles of CONSTANS Target Genes in Reproductive Development of Arabidopsis. Science. 2000;288 5471:1613-6. doi:doi:10.1126/science.288.5471.1613.
5. Soyk S, Muller NA, Park SJ, Schmalenbach I, Jiang K, Hayama R, et al. Variation in the flowering gene SELF PRUNING 5G promotes day-neutrality and early yield in tomato. Nature Genetics. 2017;49 1:162-8. doi:10.1038/ng.3733.
6. Valverde F, Mouradov A, Soppe W, Ravenscroft D and Coupland G. Photoreceptor Regulation of CONSTANS Protein in Photoperiodic Flowering. Science. 2004;303 5660:1003-6.
7. The flowering time regulator CONSTANS is recruited to the FLOWERING LOCUS T promoter via a unique cis-element. New Phytologist. 2010;187 1:57-66.
8. Zhang D, Ai G, Ji K, Huang R, Chen C, Yang Z, et al. EARLY FLOWERING is a dominant gain-of-function allele of FANTASTIC FOUR 1/2c that promotes early flowering in tomato. Plant Biotechnology Journal. 2024;22 3:698-711. doi:10.1111/pbi.14217.
9. Zhang S, Jiao Z, Liu L, Wang K, Zhong D, Li S, et al. Enhancer-Promoter Interaction of SELF PRUNING 5G Shapes Photoperiod Adaptation. Plant Physiology. 2018;178 4:1631-42. doi:10.1104/pp.18.01137.
10. Wang Q, Liu W, Leung CC, Tarte DA and Gendron JM. Plants distinguish different photoperiods to independently control seasonal flowering and growth. Science. 2024;383 6683:eadg9196. doi:10.1126/science.adg9196.
11. Gil KE and Park CM. Thermal adaptation and plasticity of the plant circadian clock. New Phytologist. 2019;221 3:1215-29. doi:10.1111/nph.15518.
12. Liu XL, Covington MF, Fankhauser C, Chory J and Wagner DR. ELF3 Encodes a Circadian Clock–Regulated Nuclear Protein That Functions in an Arabidopsis PHYB Signal Transduction Pathway. The Plant Cell. 2001;13:1293-304.
13. Yu JW, Rubio V, Lee NY, Bai S, Lee SY, Kim SS, et al. COP1 and ELF3 control circadian function and photoperiodic flowering by regulating GI stability. Molecular Cell. 2008;32 5:617-30. doi:10.1016/j.molcel.2008.09.026.
14. Alabadi D, Oyama T, Yanovsky MJ, Harmon FG and Más PK, S.A. Reciprocal Regulation Between TOC1 and LHY/CCA1 Within the Arabidopsis Circadian Clock. Science. 2001;293 5531:880-3.

15. Du SX, Wang LL, Yu WP, Xu SX, Chen L and Huang W. Appropriate induction of TOC1 ensures optimal MYB44 expression in ABA signaling and stress response in Arabidopsis. Plant, Cell & Environment. 2024;14922 14922:1-17. doi:10.1111/pce.14922.
16. Fung-Uceda J, Lee K, Seo PJ, Polyn S, De Veylder L and Mas P. The Circadian Clock Sets the Time of DNA Replication Licensing to Regulate Growth in Arabidopsis. Developmental Cell. 2018;45 1:101-13. doi:10.1016/j.devcel.2018.02.022.
17. Pan X, Ma J, Su X, Cao P, Chang W, Liu Z, et al. Structure of the maize photosystem I supercomplex with light-harvesting complexes I and II. Science. 2018;360 6393:1109-13. doi:doi:10.1126/science.aat1156.
18. Tian Y-n, Zhong R-h, Wei J-b, Luo H-h, Eyal Y, Jin H-l, et al. Arabidopsis CHLOROPHYLLASE 1 protects young leaves from long-term photodamage by facilitating FtsH-mediated D1 degradation in photosystem II repair. Molecular Plant. 2021;14 7:1149-67. doi:https://doi.org/10.1016/j.molp.2021.04.006.
19. Chen Y, Cai X, Tang B, Xie Q, Chen G, Chen X, et al. SlERF.J2 reduces chlorophyll accumulation and inhibits chloroplast biogenesis and development in tomato leaves. Plant Science. 2023;328:111578. doi:10.1016/j.plantsci.2022.111578.
20. Yan HX, Fu DQ, Zhu BZ, Liu HP, Shen XY and Luo YB. Sprout vacuum-infiltration: a simple and efficient agroinoculation method for virus-induced gene silencing in diverse solanaceous species. Plant Cell Reports. 2012;31 9:1713-22.
21. Nguyen CV, Vrebalov JT, Gapper NE, Zheng Y, Zhong S, Fei Z, et al. Tomato GOLDEN2-LIKE Transcription Factors Reveal Molecular Gradients That Function during Fruit Development and Ripening. Plant Cell. 2014;26 2:585-601.
22. Meissner R, Jacobson Y, Melamed S, Levyatuv S and Levy A. A new model system for tomato genetics. The Plant Journal. 2010;12 6:1465-72.
23. Lin T, Zhu G, Zhang J, Xu X, Yu Q, Zheng Z, et al. Genomic analyses provide insights into the history of tomato breeding. Nature Genetics. 2014;46 11:1220-6. doi:10.1038/ng.3117.
24. Gao L, Gonda I, Sun H, Ma Q, Bao K, Tieman DM, et al. The tomato pan-genome uncovers new genes and a rare allele regulating fruit flavor. Nature Genetics. 2019;51 6:1044-51. doi:10.1038/s41588-019-0410-2.
25. Zhou Y, Zhang Z, Bao Z, Li H, Lyu Y, Zan Y, et al. Graph pangenome captures missing heritability and empowers tomato breeding. Nature. 2022;606 7914:527-34. doi:10.1038/s41586-022-04808-9.
26. Perumal S, Koh CS, Jin L, Buchwaldt M, Higgins EE, Zheng C, et al. A high-contiguity Brassica nigra genome localizes active centromeres and defines the ancestral Brassica genome. Nature Plants. 2020;6 8:929-41. doi:10.1038/s41477-020-0735-y.
27. Wang Y-H, Liu P-Z, Liu H, Zhang R-R, Liang Y, Xu Z-S, et al. Telomere-to-telomere carrot (Daucus carota) genome assembly reveals carotenoid characteristics. Horticulture Research. 2023;10 7:uhad103. doi:10.1093/hr/uhad103.
28. Zhang K, Du M, Zhang H, Zhang X, Cao S, Wang X, et al. The haplotype-resolved T2T genome of teinturier cultivar Yan73 reveals the genetic basis of anthocyanin biosynthesis in grapes. Horticulture Research. 2023;10 11:uhad205. doi:10.1093/hr/uhad205.
29. Yue J, Chen Q, Wang Y, Zhang L, Ye C, Wang X, et al. Telomere-to-telomere and gap-free reference genome assembly of the kiwifruit Actinidia chinensis. Horticulture Research. 2022;10 2:uhac264. doi:10.1093/hr/uhac264.

30. Bao Y, Zeng Z, Yao W, Chen X, Jiang M, Sehrish A, et al. A gap-free and haplotype-resolved lemon genome provides insights into flavor synthesis and huanglongbing (HLB) tolerance. Horticulture Research. 2023;10 4:uhad020. doi:10.1093/hr/uhad020.
31. Zhou Y, Xiong J, Shu Z, Dong C, Gu T, Sun P, et al. The telomere-to-telomere genome of Fragaria vesca reveals the genomic evolution of Fragaria and the origin of cultivated octoploid strawberry. Horticulture Research. 2023;10 4:uhad027. doi:10.1093/hr/uhad027.
32. Canady MA, Meglic V and Chetelat RT. A library of Solanum lycopersicoides introgression lines in cultivated tomato. Genome. 2005;48 4:685-97. doi:10.1139/g05-032.
33. Su X, Wang B, Geng X, Du Y, Yang Q, Liang B, et al. A high-continuity and annotated tomato reference genome. BMC Genomics. 2021;22 898:1-12. doi:10.1186/s12864-021-08212-x.
34. Shirasawa K and Ariizumi T. Near-complete genome assembly of tomato (Solanum lycopersicum) cultivar Micro-Tom. Plant Biotechnol (Tokyo). 2024;41 4:367-74. doi:10.5511/plantbiotechnology.24.0522a.
35. Bombarely A, Moser M, Amrad A, Bao M, Bapaume L, Barry CS, et al. Insight into the evolution of the Solanaceae from the parental genomes of Petunia hybrida. Nat Plants. 2016;2 6:16074. doi:10.1038/nplants.2016.74.
36. Yang J, Wu Y, Zhang P, Ma J, Yao YJ, Ma YL, et al. Multiple independent losses of the biosynthetic pathway for two tropane alkaloids in the Solanaceae family. Nat Commun. 2023;14 1:8457. doi:10.1038/s41467-023-44246-3.
37. Xu S, Brockmller T, Navarro-Quezada A, Kuhl H and Baldwin IT. Wild tobacco genomes reveal the evolution of nicotine biosynthesis. Proceedings of the National Academy of Sciences of the United States of America. 2017; 114:6133-8.
38. Huang T, Liu H, Tao J-P, Zhang J-Q, Zhao T-M, Hou X-L, et al. Low light intensity elongates period and defers peak time of photosynthesis: a computational approach to circadian-clock-controlled photosynthesis in tomato. Horticulture Research. 2023;10 6:uhad077. doi:10.1093/hr/uhad077.
39. Suárez-López P, Wheatley K, Robson F, Onouchi H, Valverde F and Coupland G. CONSTANS mediates between the circadian clock and the control of flowering in Arabidopsis. Nature. 2001;410 6832:1116-20. doi:10.1038/35074138.
40. Lou H, Li S, Shi Z, Zou Y, Zhang Y, Huang X, et al. Engineering source-sink relations by prime editing confers heat-stress resilience in tomato and rice. Cell. 2025;188 2:530-49. doi:10.1016/j.cell.2024.11.005.
41. Chourey PS, Taliercio EW, Carlson SJ and Ruan YL. Genetic evidence that the two isozymes of sucrose synthase present in developing maize endosperm are critical, one for cell wall integrity and the other for starch biosynthesis. Molecular and General Genetics MGG. 1998;259 1:88-96. doi:10.1007/s004380050792.
42. Liu H, Zhang J-Q, Zhang R-R, Chen C, Tao J-P, Xiong J-S, et al. SlMYB1R1- module synergistically promotes sugar accumulation in tomato fruits. The Plant Journal. 2025;121 4:e70062. doi:https://doi.org/10.1111/tpj.70062.
43. van Rengs WMJ, Schmidt MH-W, Effgen S, Le DB, Wang Y, Zaidan MWAM, et al. A chromosome scale tomato genome built from complementary PacBio and Nanopore sequences alone reveals extensive linkage drag during breeding. The Plant Journal. 2022;110 2:572-88. doi:https://doi.org/10.1111/tpj.15690.

44. Parks DH, Imelfort M, Skennerton CT, Hugenholtz P and Tyson GW. CheckM: assessing the quality of microbial genomes recovered from isolates, single cells, and metagenomes. Genome Research. 2015;25 7:1043-55. doi:10.1101/gr.186072.114.

45. Haas BJ, Salzberg SL, Zhu W, Pertea M, Allen JE, Orvis J, et al. Automated eukaryotic gene structure annotation using EVidenceModeler and the Program to Assemble Spliced Alignments. Genome Biology. 2008;9 1:R7. doi:10.1186/gb-2008-9-1-r7.

46. Legnaioli T, Cuevas J and Mas P. TOC1 functions as a molecular switch connecting the circadian clock with plant responses to drought. The EMBO Journal. 2009;28 23:3745-57.

47. Tokutsu R, Fujimura-Kamada K, Matsuo T, Yamasaki T and Minagawa J. The CONSTANS flowering complex controls the protective response of photosynthesis in the green alga Chlamydomonas. Nature Communications. 2019;10 1:4099. doi:10.1038/s41467-019-11989-x.

48. Yang T, He Y, Niu S, Yan S and Zhang Y. Identification and characterization of the CONSTANS (CO)/CONSTANS-like (COL) genes related to photoperiodic signaling and flowering in tomato. Plant Science. 2020;301:110653. doi:10.1016/j.plantsci.2020.110653.

49. Wigge PA, Kim MC, Jaeger KE, Busch W, Schmid M, Lohmann JU, et al. Integration of Spatial and Temporal Information During Floral Induction in Arabidopsis. Science. 2005;309 5737:1056-9.

50. Hortensteiner S. Stay-green regulates chlorophyll and chlorophyll-binding protein degradation during senescence. Trends in Plant Science. 2009;14 3:155-62. doi:10.1016/j.tplants.2009.01.002.

51. Lepisto A and Rintamaki E. Coordination of plastid and light signaling pathways upon development of Arabidopsis leaves under various photoperiods. Molecular Plant. 2012;5 4:799-816. doi:10.1093/mp/ssr106.

52. Wang F, Han T, Song Q, Ye W, Song X, Chu J, et al. The Rice Circadian Clock Regulates Tiller Growth and Panicle Development Through Strigolactone Signaling and Sugar Sensing. Plant Cell. 2020;32 10:3124-38. doi:10.1105/tpc.20.00289.

53. Wu M, Xu X, Hu X, Liu Y, Cao H, Chan H, et al. SlMYB72 Regulates the Metabolism of Chlorophylls, Carotenoids, and Flavonoids in Tomato Fruit. Plant physiology. 2020;183 3:854-68. doi:10.1104/pp.20.00156.

54. Niu XL, Li HL, Li R, Liu GS, Peng ZZ, Jia W, et al. Transcription factor SlBEL2 interferes with GOLDEN2-LIKE and influences green shoulder formation in tomato fruits. Plant Journal. 2022;112 4:982-97. doi:10.1111/tpj.15989.

55. Liu H, Zhang JQ, Zhang RR, Zhao QZ, Su LY, Xu ZS, et al. The high-quality genome of Cryptotaenia japonica and comparative genomics analysis reveals anthocyanin biosynthesis in Apiaceae. Plant Journal. 2024;118:717-30. doi:10.1111/tpj.16628.

56. Wang Z-H, Liu X, Cui Y, Wang Y-H, Lv Z-L, Cheng L, et al. Genomic, transcriptomic, and metabolomic analyses provide insights into the evolution and development of a medicinal plant Saposhnikovia divaricata (Apiaceae). Horticulture Research. 2024:uhae105. doi:10.1093/hr/uhae105.

57. Kingsford C. A fast, lock-free approach for efficient parallel counting of occurrences of k-mers. Bioinformatics. 2011;27 6:764-70.

58. Cheng H, Concepcion GT, Feng X, Zhang H and Li H. Haplotype-resolved de novo assembly using phased assembly graphs with hifiasm. Nature Methods. 2021;18 2:170-5.

doi:10.1038/s41592-020-01056-5.

59. A. SF, Waterhouse RM, Panagiotis I, Kriventseva EV and Zdobnov EM. BUSCO: assessing genome assembly and annotation completeness with single-copy orthologs. Bioinformatics. 2015; 19:3210-2.

60. Wolff J, Rabbani L, Gilsbach R, Richard G, Manke T, Backofen R, et al. Galaxy HiCExplorer 3: a web server for reproducible Hi-C, capture Hi-C and single-cell Hi-C data analysis, quality control and visualization. Nucleic Acids Research. 2020;48 1:177-84. doi:10.1093/nar/gkaa220.

61. Ou S and Jiang N. LTR_FINDER_parallel: parallelization of LTR_FINDER enabling rapid identification of long terminal repeat retrotransposons. Mobile DNA. 2019;10:48.

62. Graovac MT and Chen N. Using RepeatMasker to Identify Repetitive Elements in Genomic Sequences. Current Protocols in Bioinformatics. 2009;25 1:1-14.

63. Mario S, Oliver K, Irfan G, Alec H, Stephan W and Burkhard M. AUGUSTUS: ab initio prediction of alternative transcripts. Nucleic Acids Research. 2006;34:435-9.

64. Holt C and Yandell M. MAKER2: an annotation pipeline and genome-database management tool for second-generation genome projects. BMC Bioinformatics. 2011;12 1:491. doi:10.1186/1471-2105-12-491.

65. Nawrocki EP, Kolbe DL and Eddy SR. Infernal 1.0: inference of RNA alignments. Bioinformatics. 2009;25 10:1335-7.

66. Marçais G, Delcher AL, Phillippy AM, Coston R, Salzberg SL and Zimin A. MUMmer4: A fast and versatile genome alignment system. PLOS Computational Biology. 2018;14 1:e1005944. doi:10.1371/journal.pcbi.1005944.

67. Goel M, Sun H, Jiao W-B and Schneeberger K. SyRI: finding genomic rearrangements and local sequence differences from whole-genome assemblies. Genome Biology. 2019;20 1:277. doi:10.1186/s13059-019-1911-0.

68. Yang Z. PAML 4: Phylogenetic Analysis by Maximum Likelihood. Molecular Biology and Evolution. 2007;24 8:1586-91.

69. Bie TD, Cristianini N, Demuth JP, Hahn and W. M. CAFE: a computational tool for the study of gene family evolution. Bioinformatics. 2006;22 10:1269-71.

70. Wang YP, Tang HB, Jeremy D D, Tan X, Li JP, Wang XY, et al. MCScanX: a toolkit for detection and evolutionary analysis of gene synteny and collinearity. Nucleic Acids Research. 2012;40 7:e49.

71. Xu ZS, Yang QQ, Feng K, Yu X and Xiong AS. DcMYB113, a root-specific R2R3-MYB, conditions anthocyanin biosynthesis and modification in carrot. Plant Biotechnology Journal. 2020;18 7:1585-97. doi:10.1111/pbi.13325.

72. Liu H, Zhang JQ, Tao JP, Chen C, Su LY, Xiong JS, et al. Supporting data for "A telomere-to-telomere gapless genome reveals SlPRR1 control of circadian rhythm and photoperiodic flowering in tomato" GigaScience Database. 2025. https://doi.org/10.5524/102698

Table and figures

Table 1 Statistics for genome assembly and annotation of VF36 and Heinz 1706 genomes

Genomic feature	VF36 (this study)	Heinz 1706 (SLT1.0)	Heinz 1706 (SL5.0)
Total size of assembly contigs (Mb)	815.27	799.09	801.81
Number of contigs (gaps)	12 (0)	12 (210)	12 (31)
Number of telomeres	24	0	0
Number of centromeres	12	0	0
Number of gene models	34,783	34,384	36,648
Total size of TEs (Mb)	600.23	558.49	491.27
Annotation BUSCOs (%)	98.2	98.2	94.8
Genome BUSCOs (%)	98.27%	97.70%	97.60%

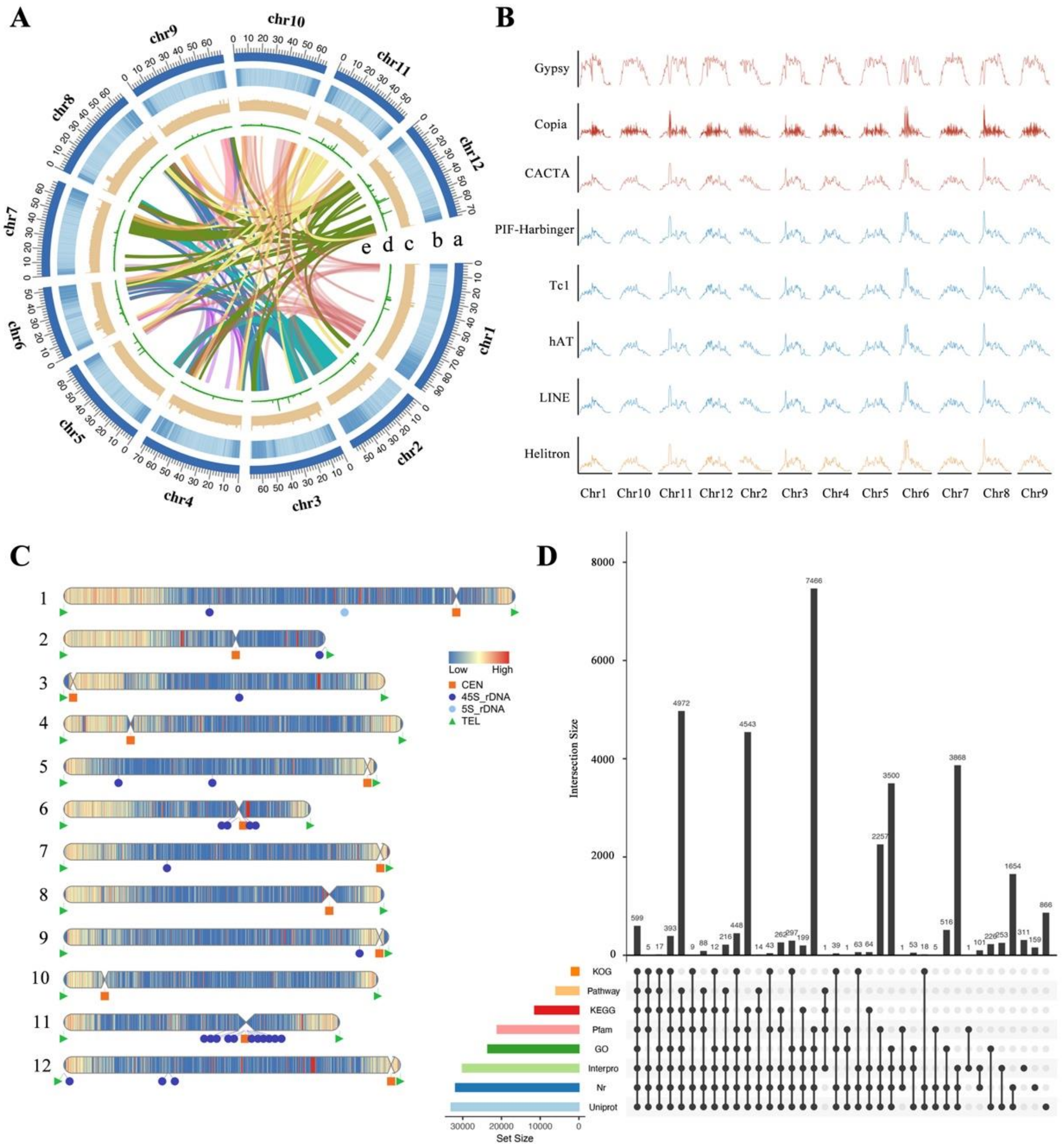

Figure 1 Complete genome assembly and annotation of VF36 tomato

(A) Circos plot of VF36 genome annotation. Quantitative tracks are aggregated in a 10-kb window. Track a, chromosomes information. Track b, gene density. Track c, GC content. Track d, repeat coverage. Track e, collinearity information.

(B) Track displaying density of *Gypsy*, *Copia*, *CACTA*, *PIF-Harbinger*, *Tc1*, *hAT*, *LINE*, and *Helitron* elements.

(C) Map of centromere prediction for VF36 genome.

(D) Distribution of VF36 genomic features.

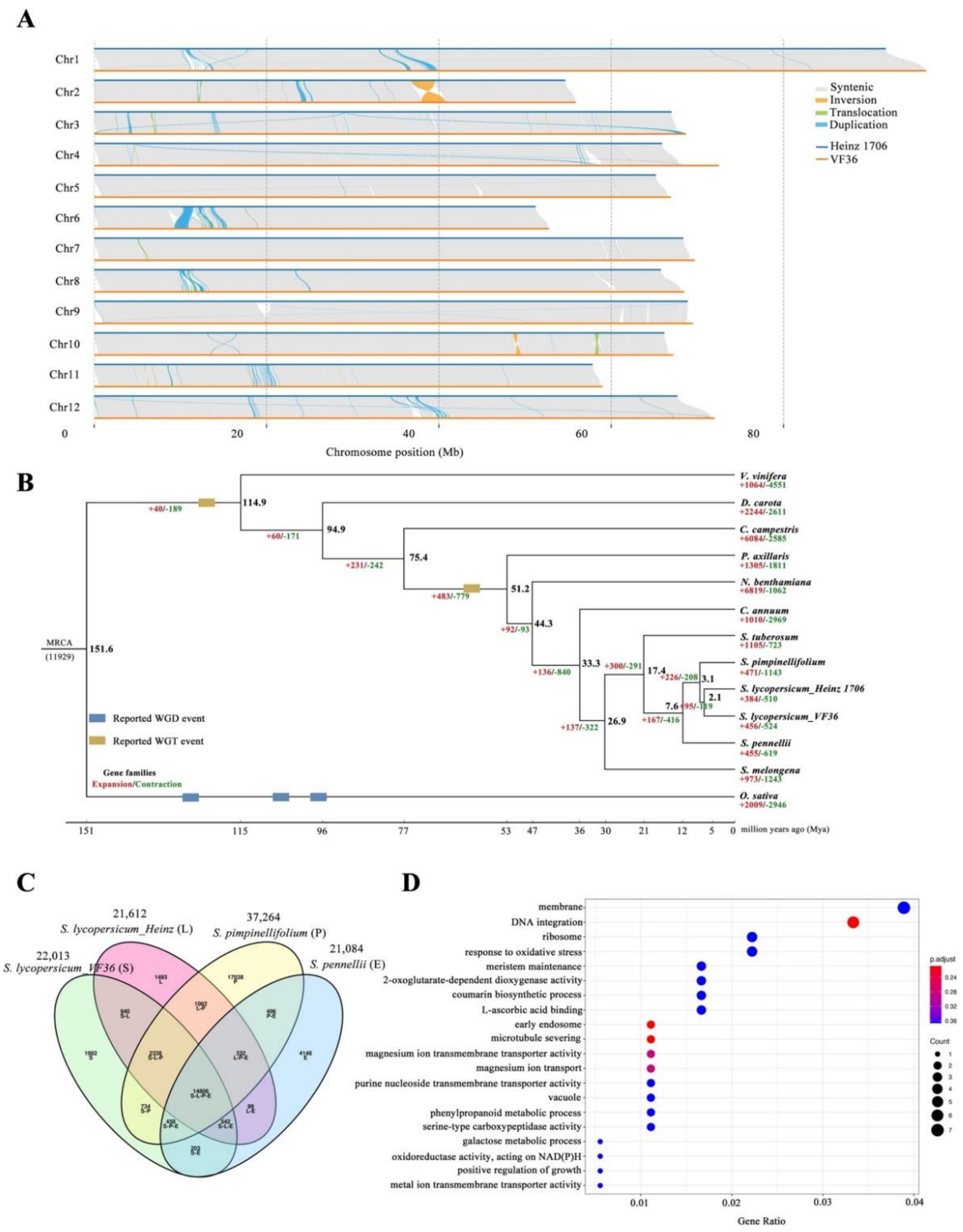

Figure 2 Comparative genomic analysis of VF36 genome.

(A) Structural variations between Heinz_1706 and VF36 genomes.

(B) Estimation of divergence time and gene family expansion/contraction. The blue blocks represent the published whole-genome duplication (WGD) events. The dark yellow blocks represent the published whole-genome triplication events.

(C) Venn diagram of gene family clustering.

(D) The GO enrichment on the genes in SV regions.

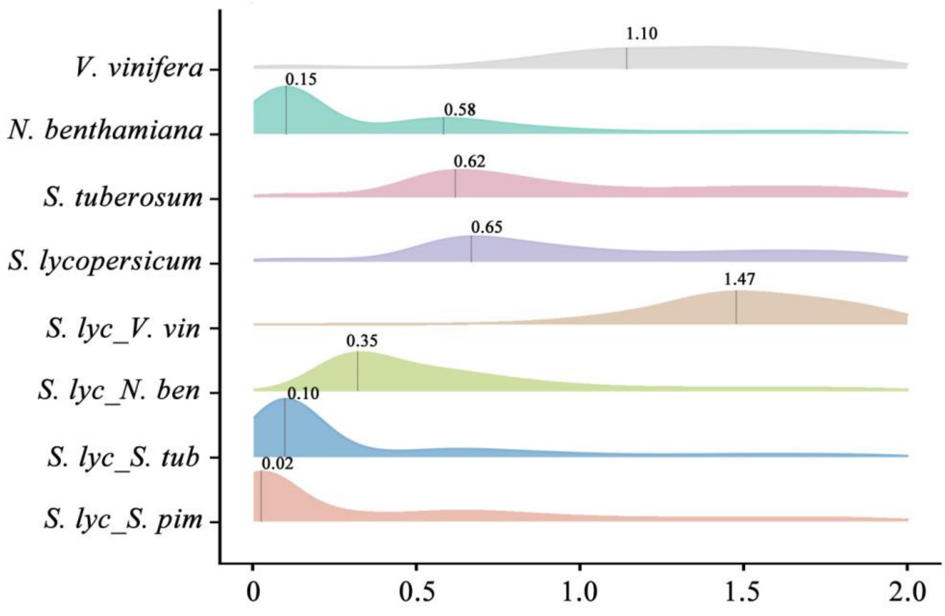

Figure 3 Gene duplication and evolution

K_S distribution from orthologs and paralogs among *S. pimpinellifolium*, *S. tuberosum*, *N. benthamiana*, *V. vinifera*, and *S. lycopersicum*.

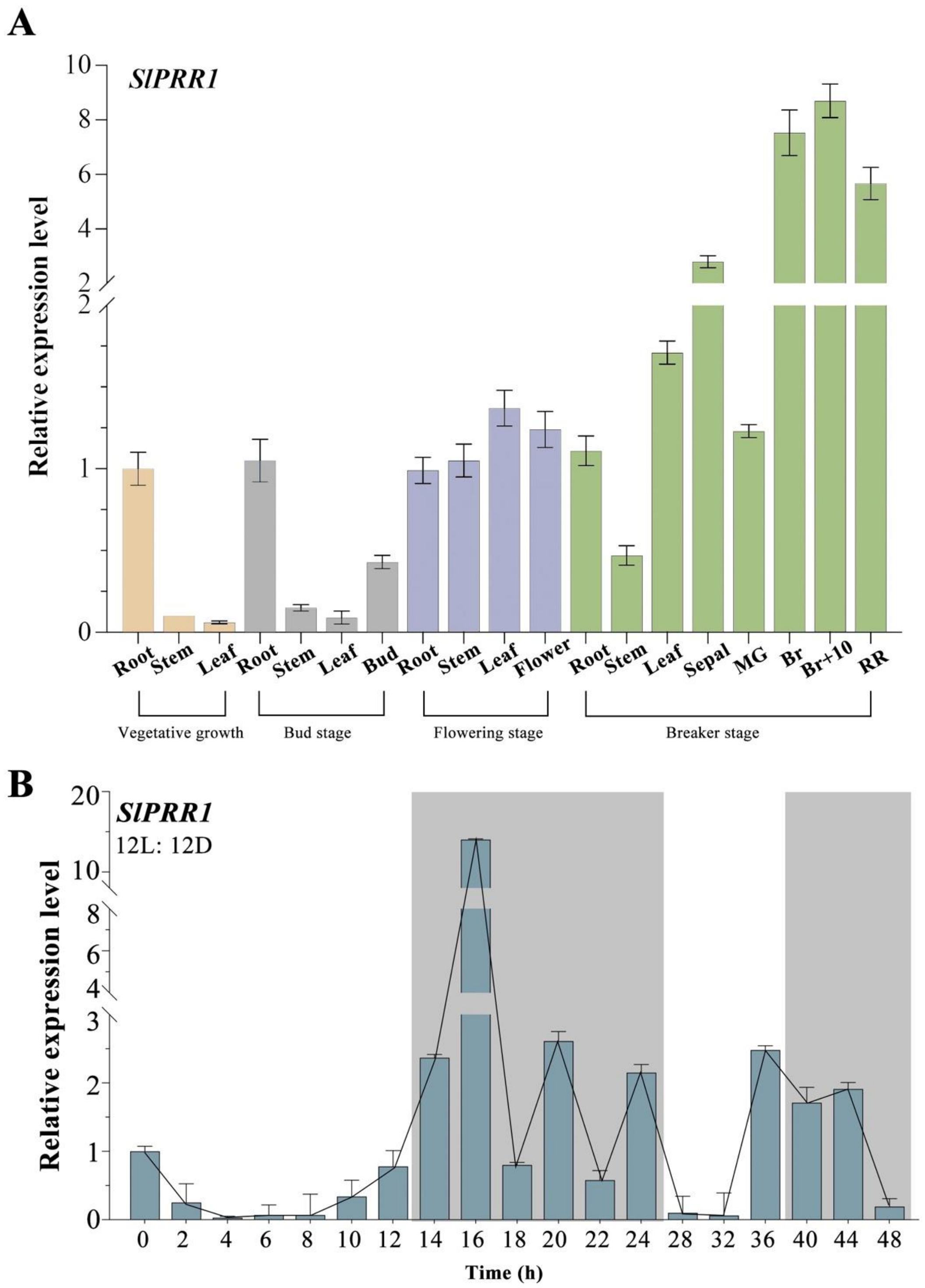

Figure 4 Expression and functional analysis of *SlPRR1* in circadian rhythm

(A) Expression of *SlPRR1* gene in various stages of tomato tissues.

(B) Expression of *SlPRR1* gene from tomato plants grown in 12L: 12D. Shading indicates the dark period.

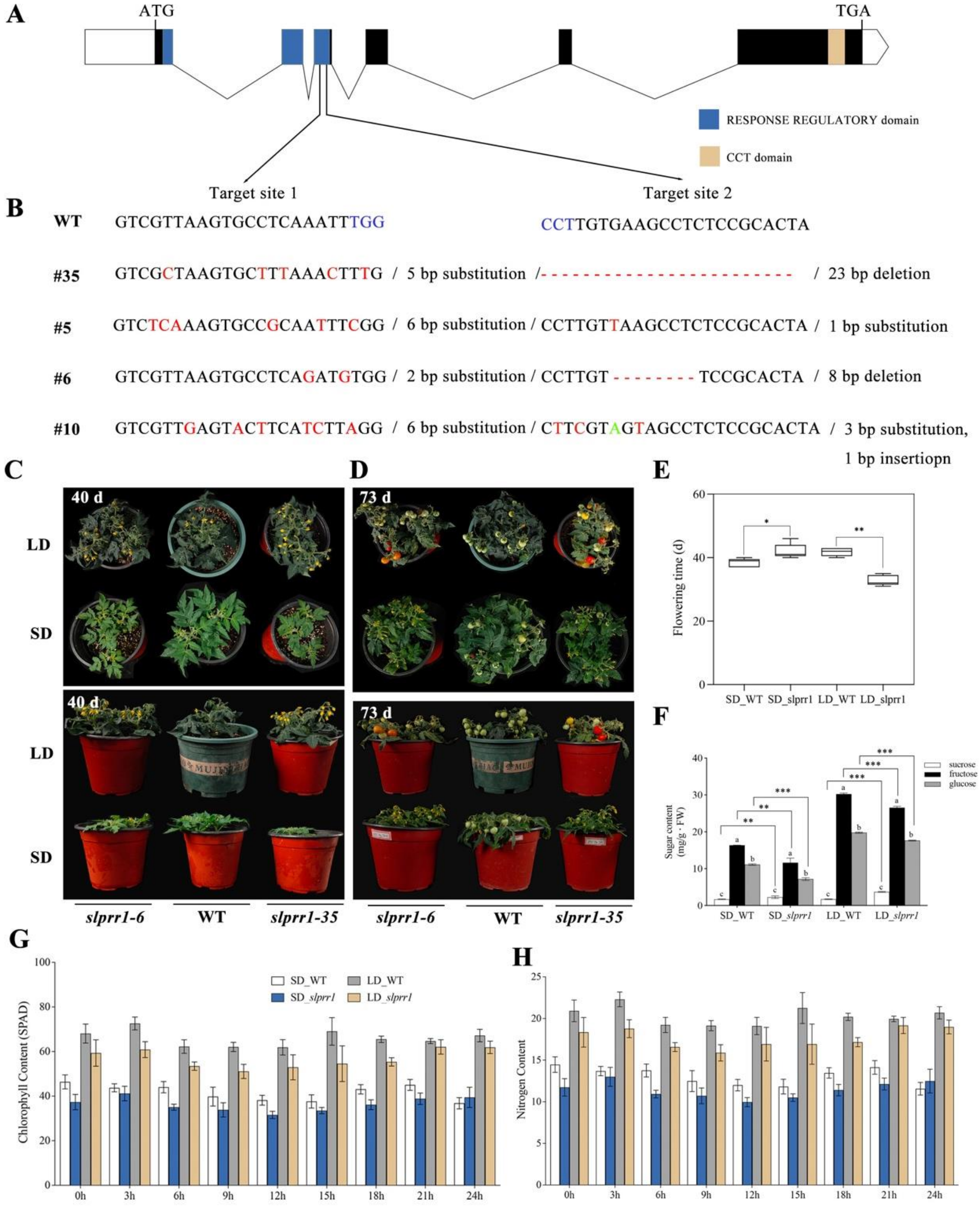

Figure 5 Functional analysis of *SlPRR1* in the regulation of tomato flowering time

(A) The two sgRNA target sites in *SlPRR1* locus used for CRISPR/Cas9 gene-editing system.

(B) The mutation types of *SlPRR1* in *slprr1*-35, *slprr1*-5, *slprr1*-6, and *slprr1*-10 line. Red letters indicated the substitution sites, green letters indicated the insertion sites, blue letters indicated the PAM.

(C-E) Flowering phenotype from 40-day-old (C), 73-day-old (D) tomato plants, and flowering time (E) of the WT, *slprr1*-6, and *slprr1*-35 lines under LD (16L: 8D) and SD (8L: 16D) conditions.

(F) The content of sucrose, fructose, and glucose of red ripening tomato fruits in WT and *slprr1* lines under SD or LD conditions.

(G-H) The Chhlorophyll (G) and Nitrogen (H) content of tomato leaves in WT and *slprr1* lines under SD or LD conditions during the photoperiod.

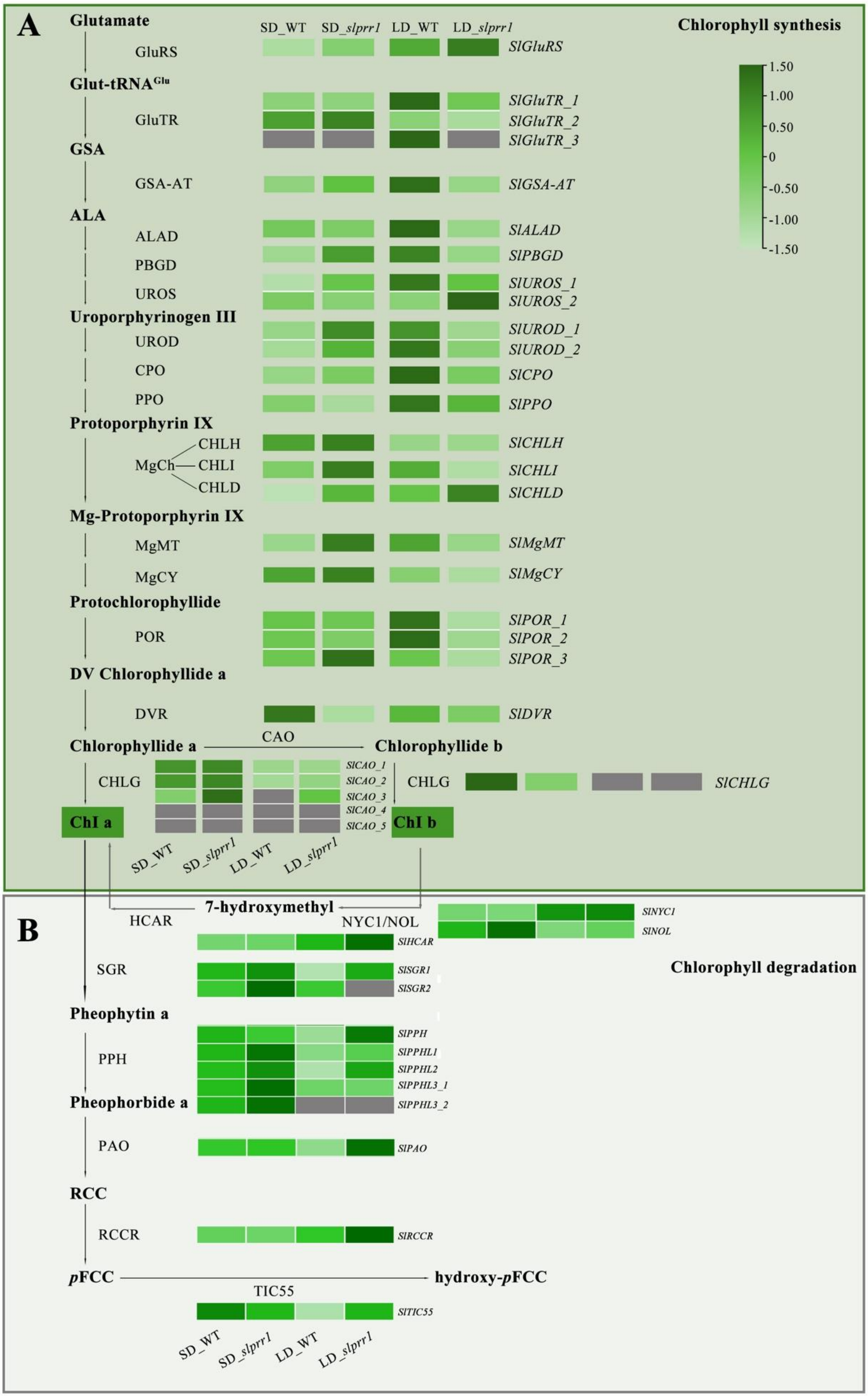

Figure 6 Chlorophyll synthesis and degradation of tomato leaves in WT and *slprr1*

lines under SD or LD conditions

(A) The potential simplified Chlorophyll synthesis pathway in tomato leaves is depicted.

(B) The potential simplified Chlorophyll degradation pathway in tomato leaves is depicted.

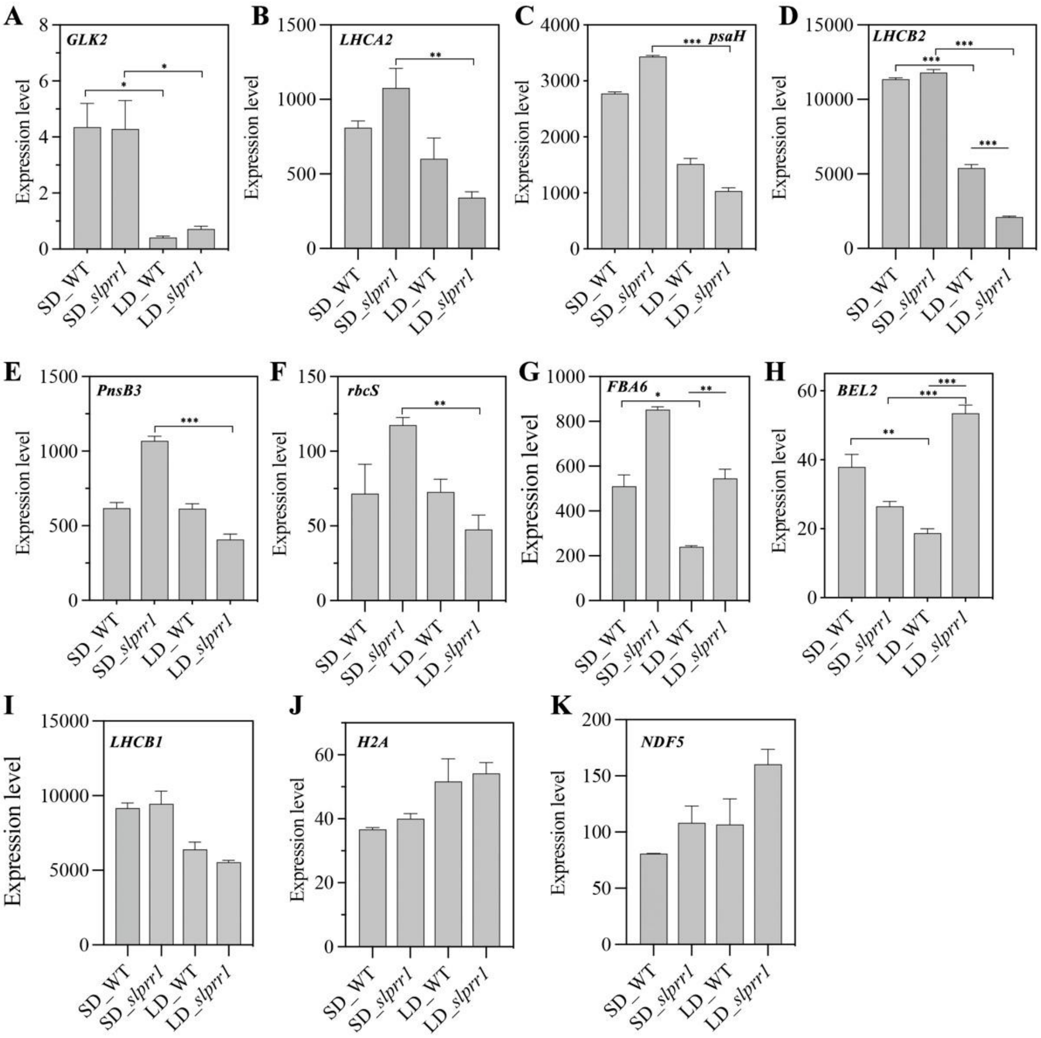

Figure 7 Relative expression levels of photosynthesis and Chlorophyll synthesis-related genes in tomato leaves. Error bars represent the averages of three biological replicates ± SD. Asterisks indicate statistical significance (***P* < 0.01, **P* < 0.05, Student's *t*-test).

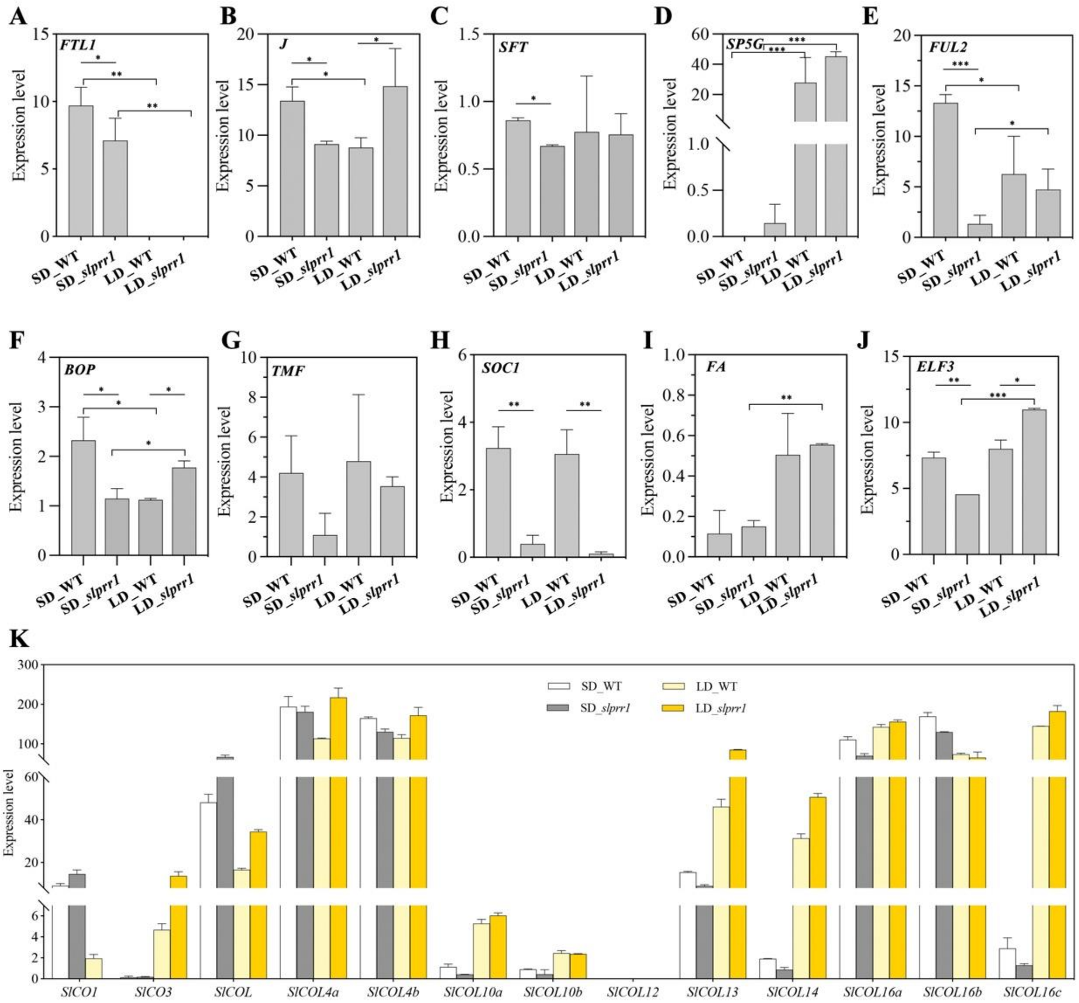

Figure 8 Relative expression levels of flowering-related genes in tomato. Error bars represent the averages of three biological replicates ± SD. Asterisks indicate statistical significance (***P* < 0.01, **P* < 0.05, Student's *t*-test).

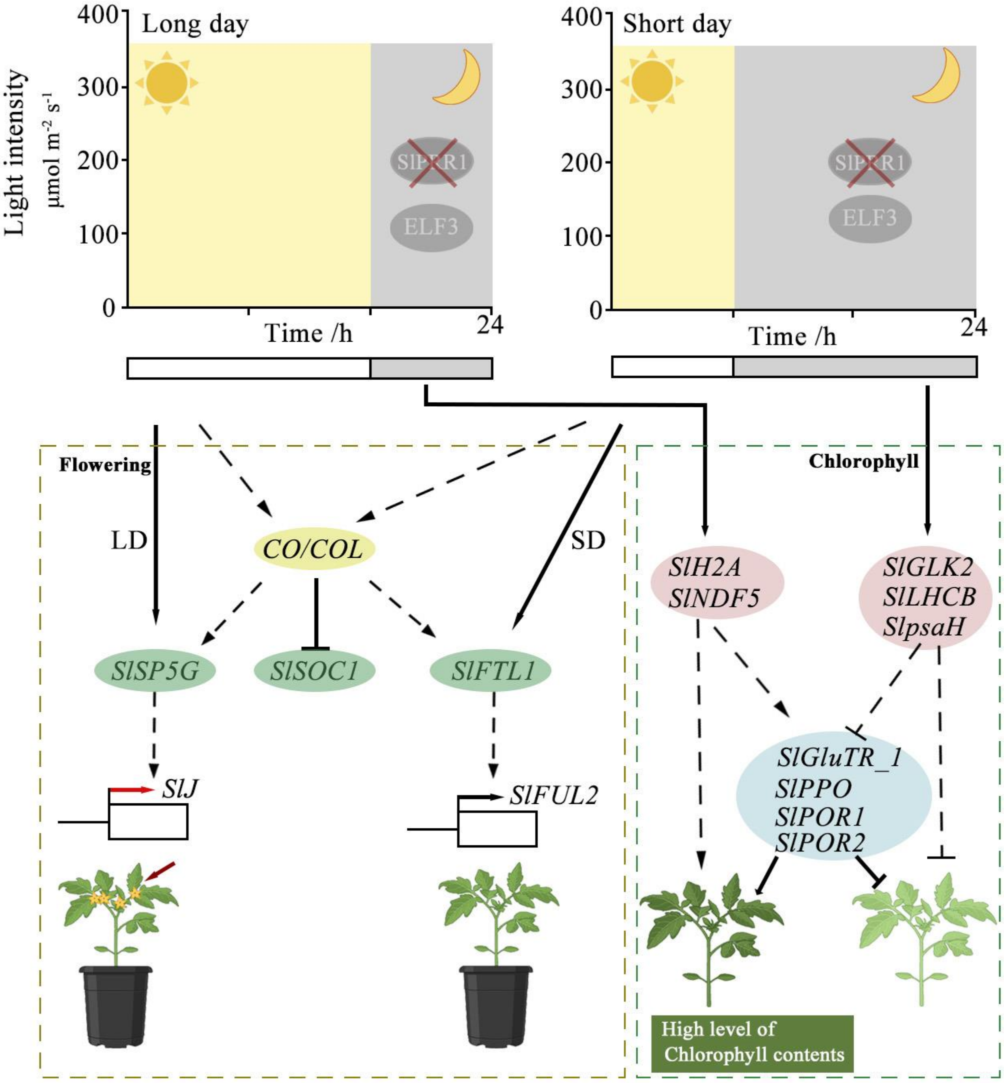

Figure 9 Model for the regulation of flowering time and Chlorophyll synthesis by knock-out *SlPRR1* in tomato under LD and SD conditions

Supplementary material

Supplementary Table 1. *K*-mer statistics of the genomic characteristics of 'VF36' tomato obtained by genome.

Supplementary Table 2. Summary of sequencing data.

Supplementary Table 3. Summary of assembly data.

Supplementary Table 4. General statistics of the telomeres for VF36 genome.

Supplementary Table 5. Predicted centromeric regions.

Supplementary Table 6. Statistics of repeat content in VF36 genome.

Supplementary Table 7. General statistics of predicted protein-coding genes.

Supplementary Table 8. BUSCOs analysis of VF36 genome completeness.

Supplementary Table 9. Functional annotation of the predicted genes.

Supplementary Table10. The statistics of non-coding RNA in VF36 genome.

Supplementary Table11. The statistics of variation between VF36 and Heinz1706 genome.

Supplementary Table12. Structure variations between VF36 and Heinz1706 genome.

Supplementary Table 13. Gene sets used in this study.

Supplementary Table 14. Summary of clustered gene families by OrthoFinder among 13 species.

Supplementary Table 15. The information of collinear gene pairs.

Supplementary Table 16. The primers used in this study.

Figure S1 'VF36' tomato fruits of MG and RR stages.

Figure S2 *K*-mer=19 Depth and *K*-mer number frequency distribution map.

Figure S3 Twelve pseudomolecules scaffolding with Hi-C data.

Figure S4 Density of genes and TRs, blue lines represent LTR/Copia, orange lines

represent LTR/Gyps, dark red lines represent TRF, and green lines represent genes.

Figure S5 K_S distribution from putative collinear homologous genes between the 'VF36' and the 'Heinz 1706' tomato.

Figure S6 GO enrichment of expanded gene families.

Figure S7 GO enrichment of unique genes.

Figure S8 Syntenic dot plot of *V. vinifera* versus 'VF36' tomato.

Figure S9 Syntenic dot plot of *N. benthamiana* versus 'VF36' tomato.

Figure S10 Sequences alignment of SlPRR1 from the 'VF36' and the 'Heinz 1706' tomatoes.

Figure S11 Relative expression levels of *LIN5*, *SUT1*, and *SWEET* genes in tomato.

 Click here to access/download;Table;Table 1-revised.xlsx

Table1 Statistics for genome assembly and annotation of VF36 and Heinz 1706 genom

Genomic feature	VF36 (this study)	Heinz 1706 (SLT1.0)
Total size of assembly contigs (Mb)	815.27	799.09
Number of contigs (gaps)	12 (0)	12 (210)
Number of telomeres	24	0
Number of centromeres	12	0
Number of gene models	34,783	34,384
Total size of TEs (Mb)	600.23	558.49
Annotation BUSCOs (%)	98.5	98.2
Genome BUSCOs (%)	98.64%	97.70%

es.

Heinz 1706 (SL5.0)	MicroTom (SLM_r2.0)
801.81	832.77
12 (31)	12 (16,700)
0	0
0	0
36,648	31,429
491.27	610.8
94.8	98.5
97.60%	98.5

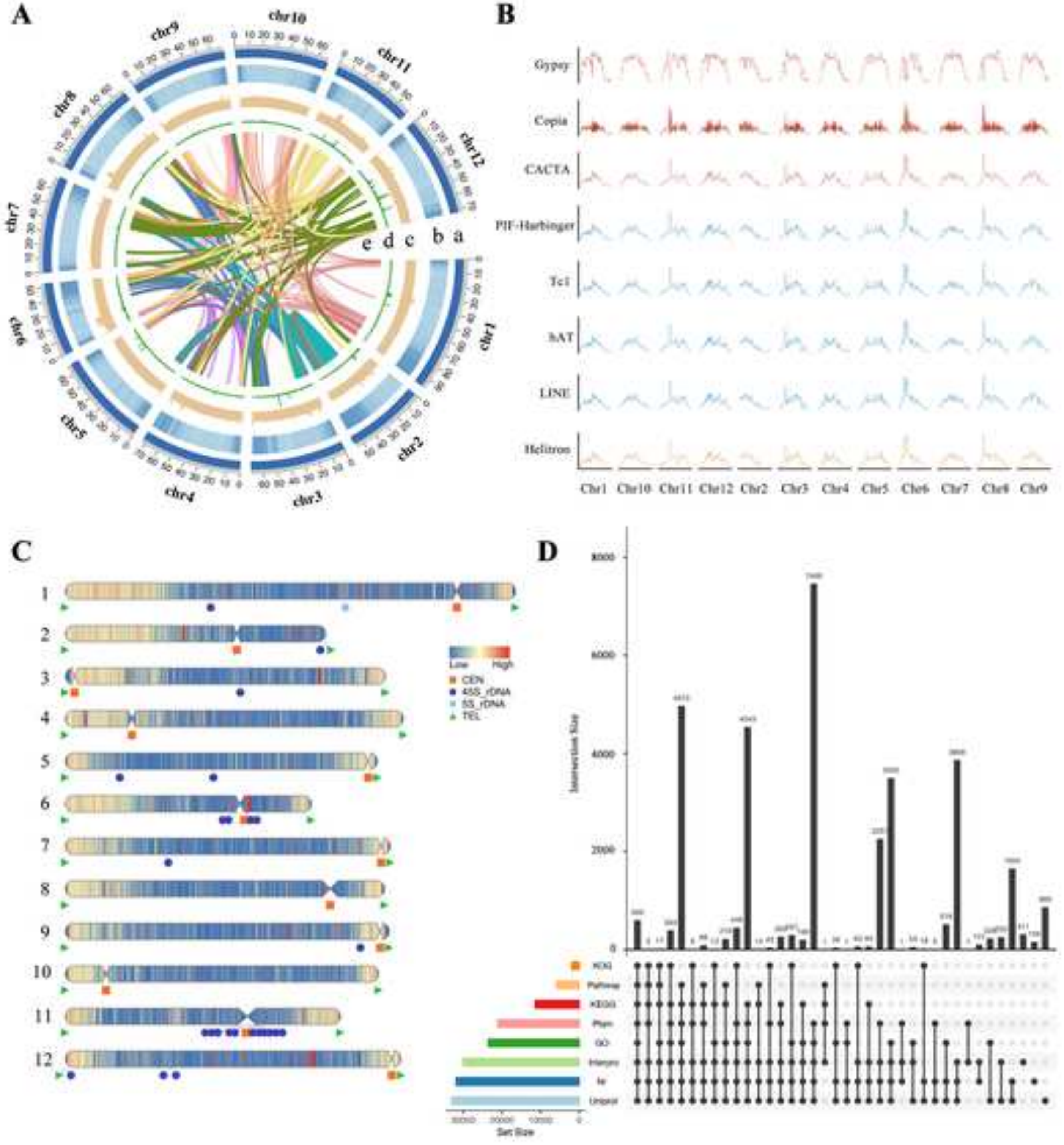
A
B
C
D
chr1
chr2
chr3
chr4
chr5
chr6
chr7
chr8
chr9
chr10
chr11
chr12
a
b
c
d
e
Gypsy
Copia
CACTA
PIF-Harbinger
Tc1
hAT
LINE
Helitron
Chr1 Chr10 Chr11 Chr12 Chr2 Chr3 Chr4 Chr5 Chr6 Chr7 Chr8 Chr9
Low
High
CEN
45S_rDNA
5S_rDNA
TEL
Intersection Size
Set Size

Figure 2

Click here to access/download;Figure;Figure 2.jpg

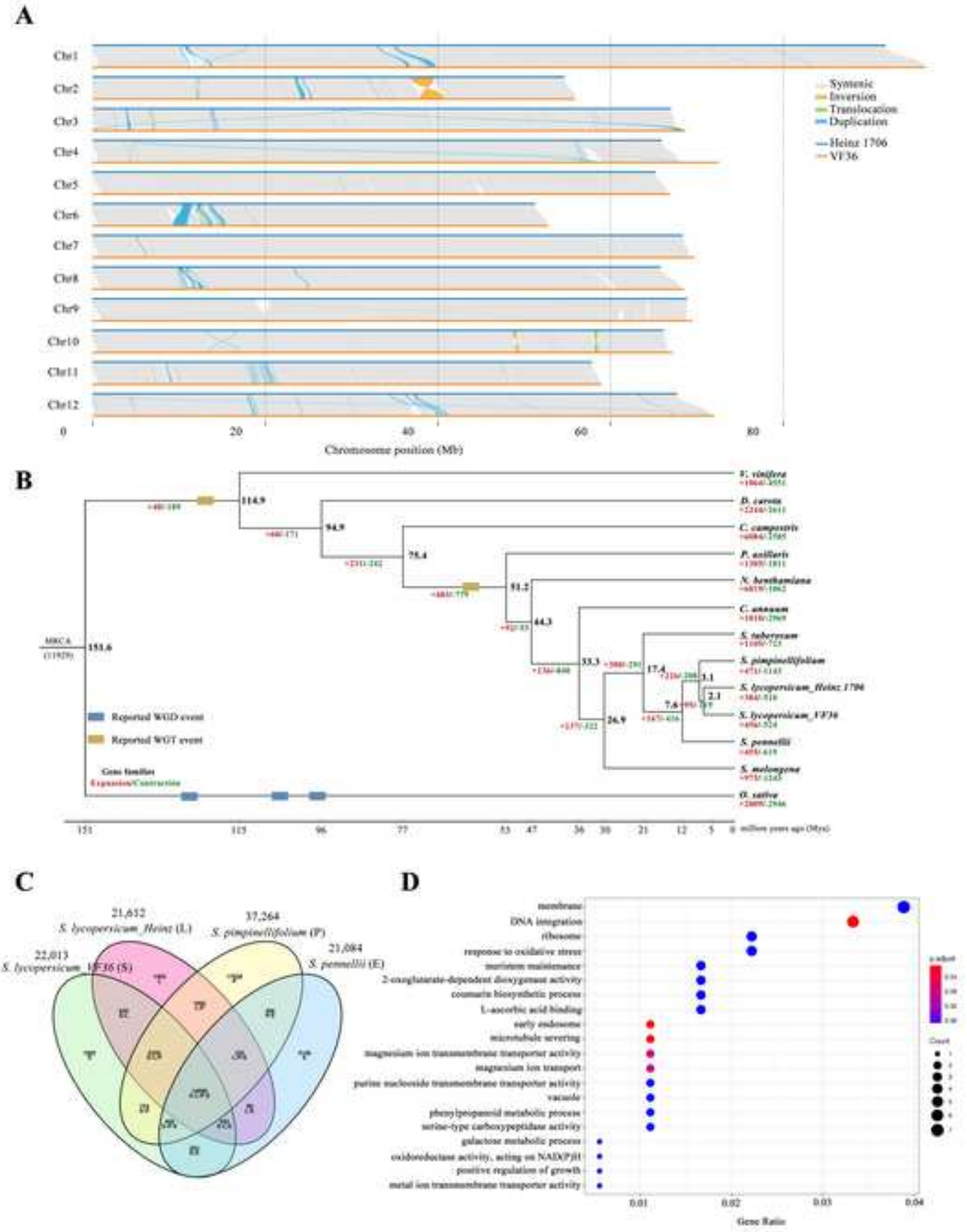

Figure 3

Click here to access/download;Figure;Figure 3.jpg

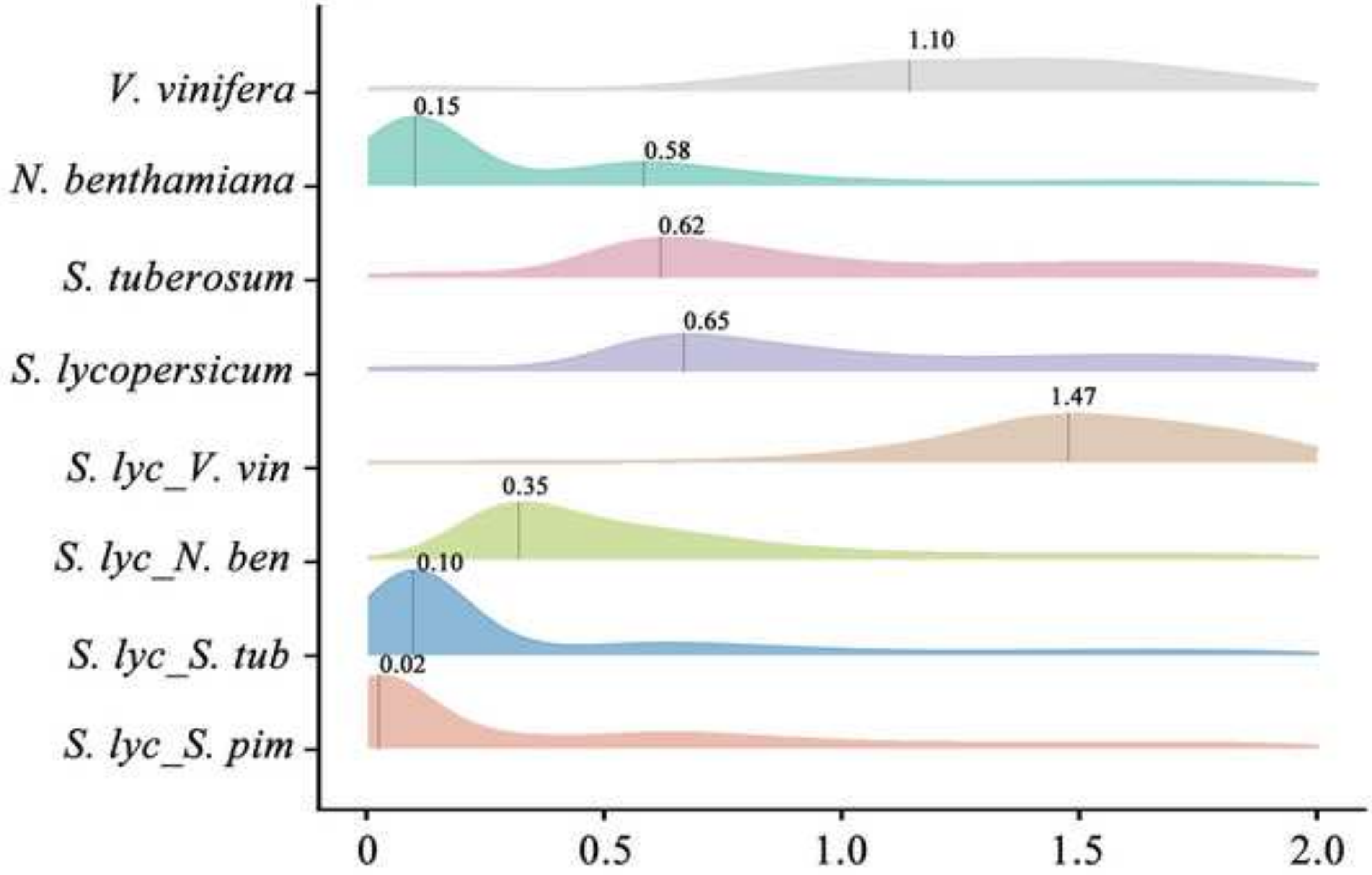

Figure 4

Click here to access/download;Figure;Figure 4.jpg

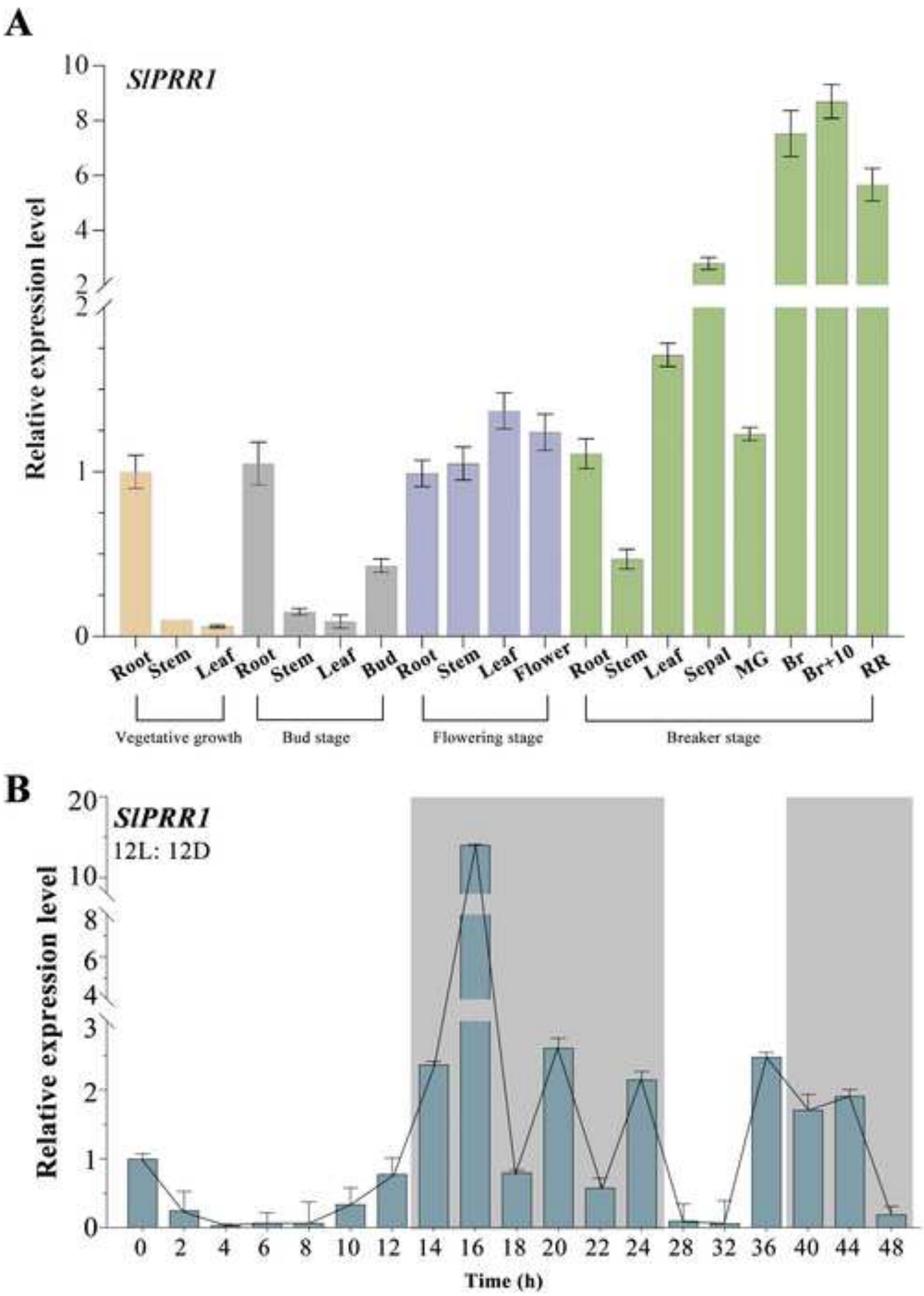

Figure 5

Click here to access/download;Figure;Figure 5.jpg

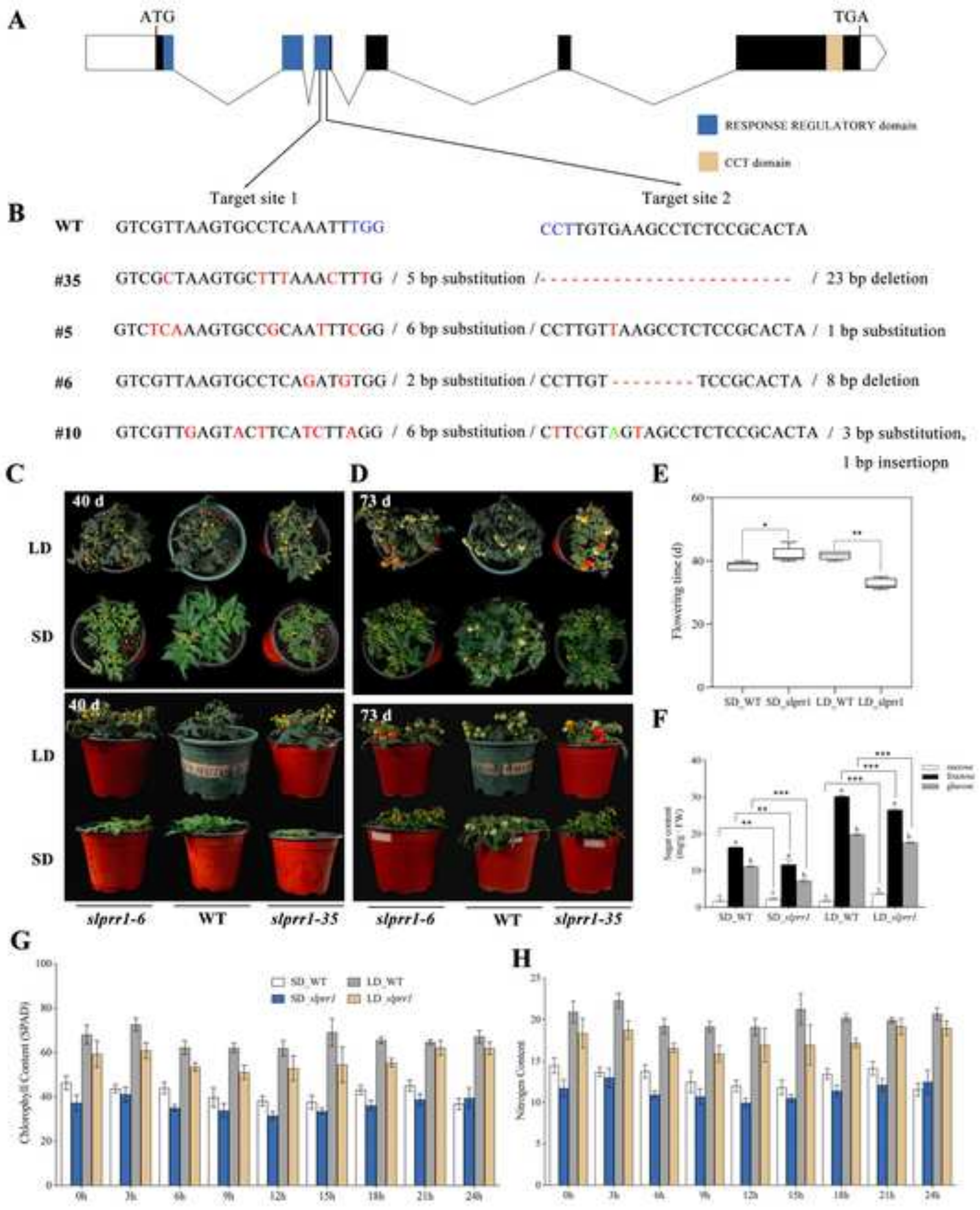

Click here to access/download;Figure;Figure 6.jpg

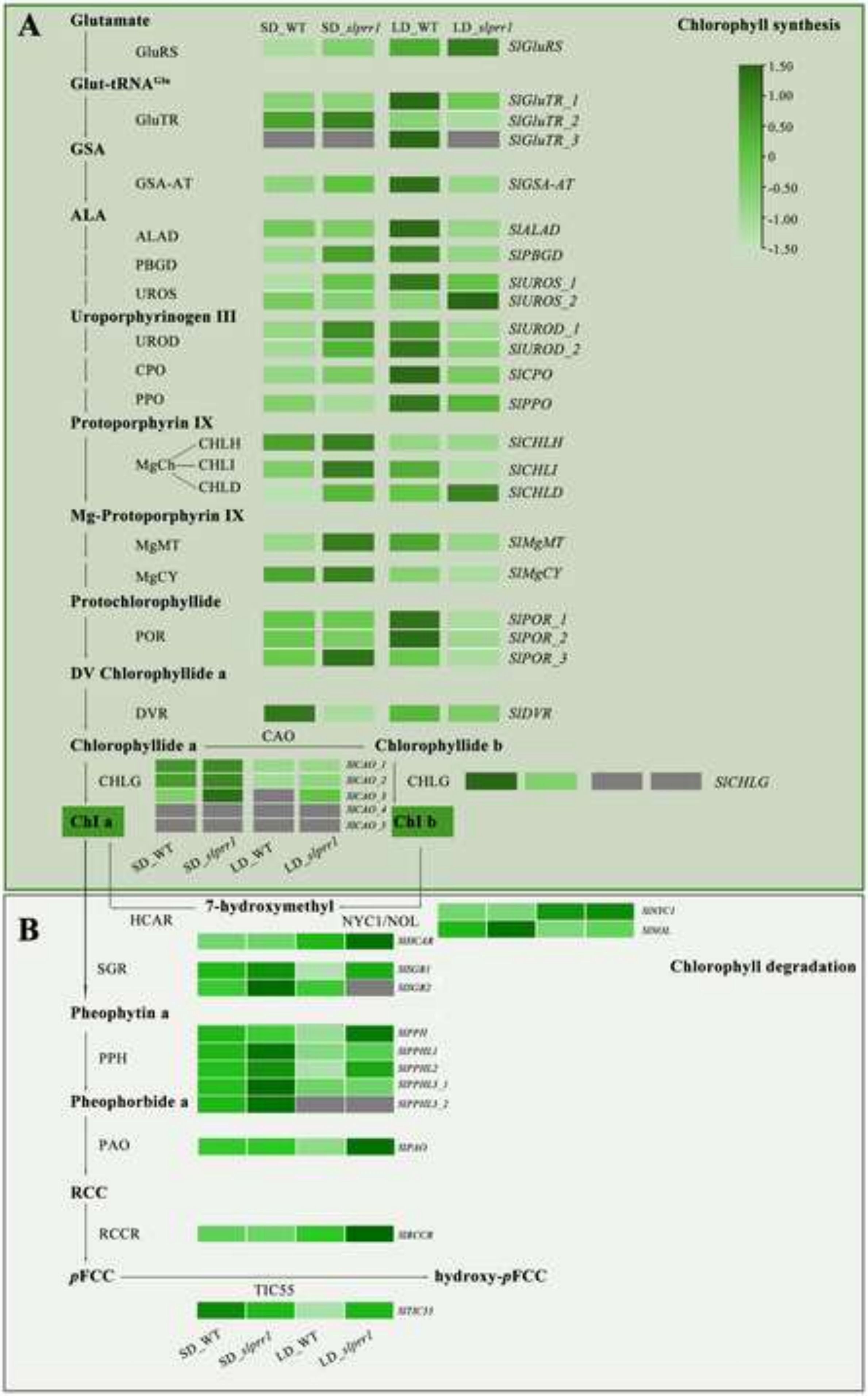

Figure 7 Click here to access/download;Figure;Figure 7.jpg

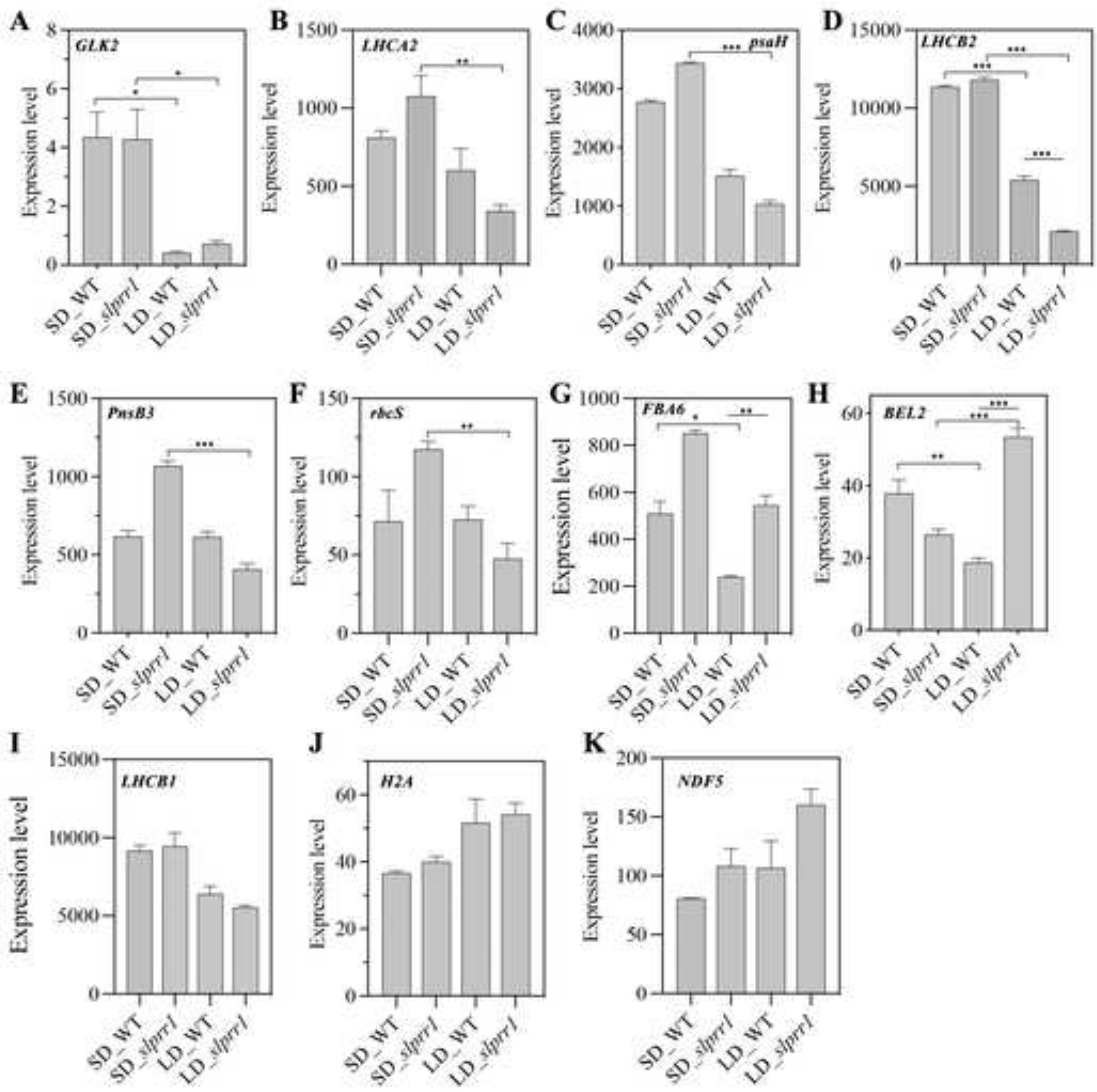

Click here to access/download;Figure;Figure 8.jpg

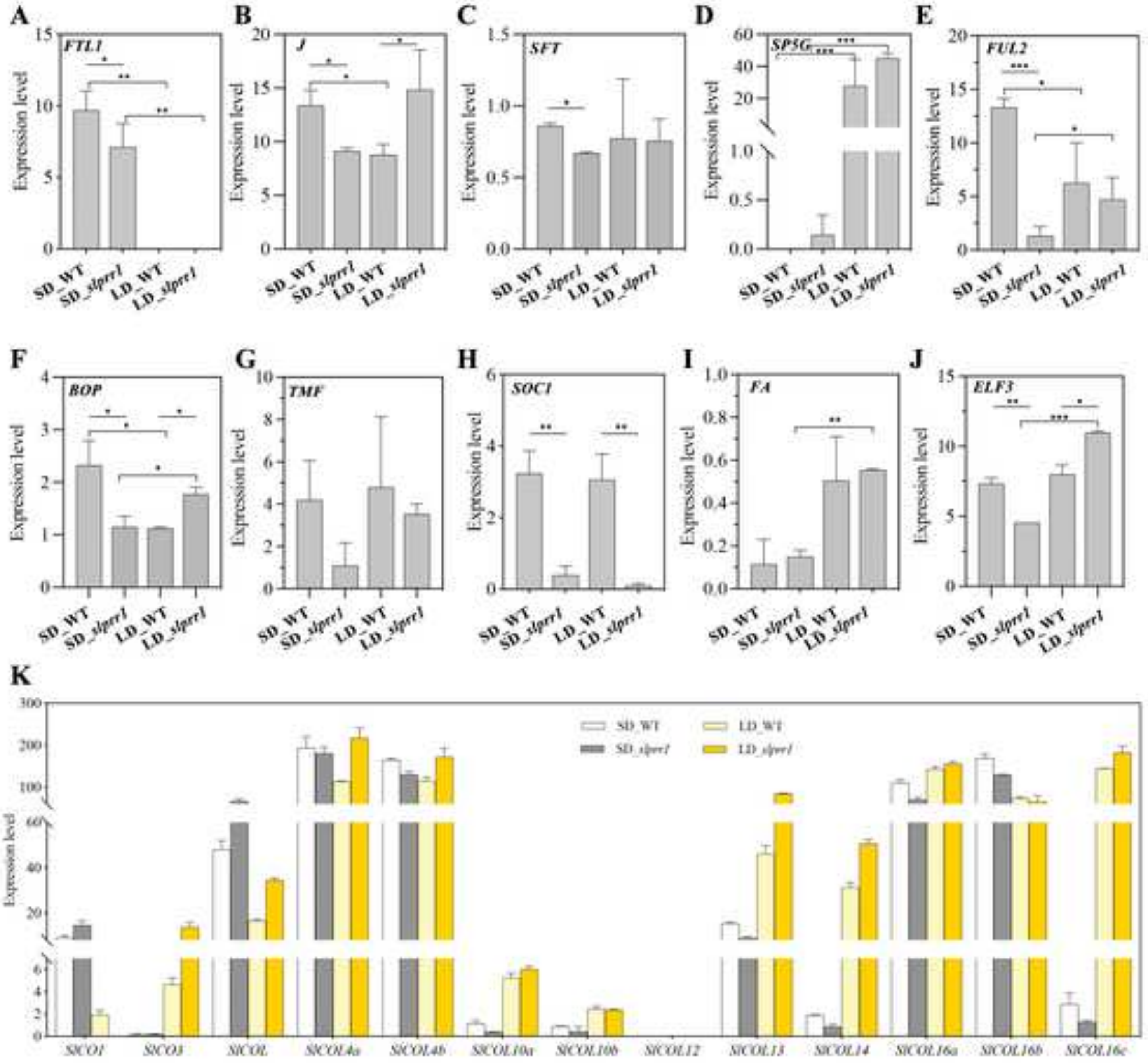

Figure 9

Click here to access/download;Figure;Figure 9.jpg

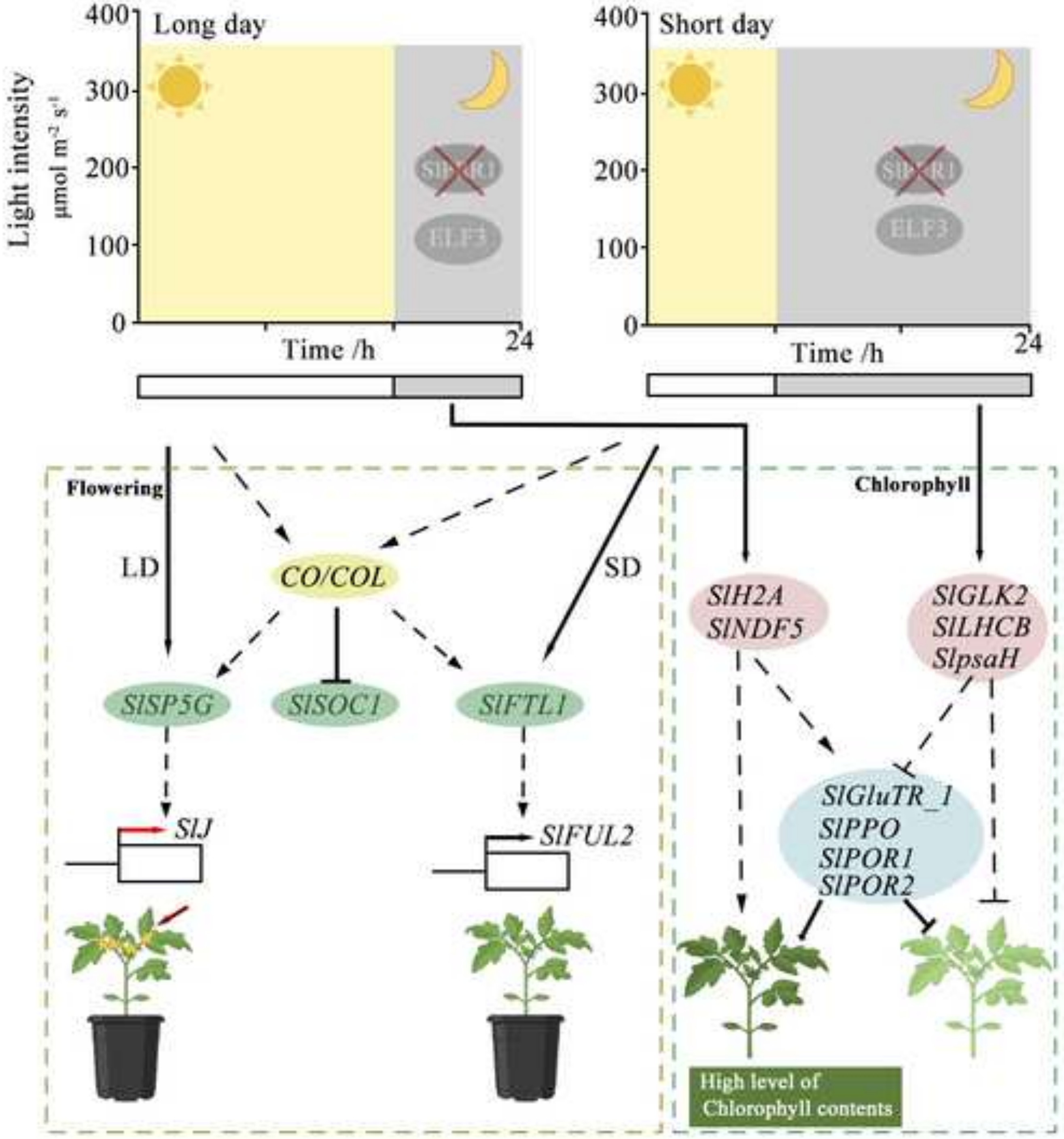

Supplementary tables

Click here to access/download
Supplementary Material
supplementary tables-revised.xlsx

Supplementary Figure 5

Click here to access/download

Supplementary Material

Figure S5.jpg

Supplementary Figure 6

Click here to access/download
Supplementary Material
Figure S6.jpg

Supplementary Figure 7

Click here to access/download
Supplementary Material
Figure S7.jpg

Supplementary Figure 8

Click here to access/download
Supplementary Material
Figure S8.jpg

Supplementary Figure 9

Click here to access/download
Supplementary Material
Figure S9.jpg

Supplementary Figure 10

Click here to access/download
Supplementary Material
Figure S10.jpg

Supplementary Figure 11

Click here to access/download
Supplementary Material
Figure S11.jpg

Personal Cover Click here to access/download;Personal Cover;response to reviewers.docx

2 April 2025

GigaScience

Manuscript ID: GIGA-D-24-00568

A telomere-to-telomere gapless genome reveals SlPRR1 control circadian rhythm and photoperiodic flowering in cultivated tomato

Dear Prof. Zhang

Thank you very much for your assistance in handing our manuscript (A telomere-to-telomere gapless genome reveals SlPRR1 control circadian rhythm and photoperiodic flowering in cultivated tomato). Your effort and time spent on our manuscript are greatly appreciated by all of us. We are delighted to all suggestions and review comments, which you and the reviewers made. Your revisions/suggestions have definitely improved the quality of our manuscript.

The manuscript was edited extensively according to reviewer' comments. The language of the manuscript has been improved by a copy-editing company. Please find the revised manuscript in '**GigaScience**' manuscript center. The changes were made directly in the text with **RED** marked. The responses to the reviewers are highlighted below.

Thank you again for your kind help and excellent suggestions for our manuscript. We hope these revisions will be satisfactory and will lead to acceptance for publication. We are looking forward to hearing from you soon.

Yours sincerely

Ai-Sheng Xiong

Dr. Ai-Sheng Xiong

Professor

State Key Laboratory of Crop Genetics & Germplasm Enhancement and Utilization,

College of Horticulture,

Nanjing Agricultural University

1 Weigang, 210095, Nanjing, China

Fax: 86 25 84396790

Email: xiongaisheng@njau.edu.cn

Editor's comments:

We suggest you find a copy-editing company or friendly native English speaker to polish the grammar.

Response:

--We thank the editor and the reviewers for this suggestion.

--As suggested by the editor and both reviewers, the manuscript has been carefully checked and the language of the manuscript has been improved by the Wiley editing services. All the errors have been corrected. (See revised manuscript, English editing certificate)

Wiley Editing Services

ENGLISH EDITING CERTIFICATE

This document certifies that the manuscript listed below was edited for proper English language, grammar, punctuation, spelling, and overall style by one or more of the highly qualified native English speaking editors at Wiley Editing Services

Manuscript title
A telomere-to-telomere gapless genome reveals SlPRR1 control of circadian rhythm and photoperiodic flowering in cultivated tomato

Authors
Hui Liu

Order No
VUQZC_1

Date Issued
March 08, 2025

This document certifies that the manuscript listed above was edited for proper English language, grammar, punctuation, spelling, and overall style. Neither the research content nor the authors' intentions were altered in any way during the editing process. Documents receiving this certification should be English-ready for publication; however, the author has the ability to accept or reject our suggestions and changes. If you have any questions or concerns about this document or certification, please contact help-cn@wileyeditingservices.com.

Wiley Publishing Services is a service of Wiley Publishing. Wiley's Scientific, Technical, Medical, and Scholarly (STMS) business serves the world's research and scholarly communities, and is the largest publisher for professional and scholarly societies. Wiley is committed to providing high quality services for researchers. To find out more about Wiley Editing Services, visit http://wileyeditingservices.com. To learn more about our other author services provided by Wiley

WILEY

Reviewers' comments:

Reviewer #1:

Major Concerns:

1. Looks very interesting. I'd be happy to review if once the english language is readable.

Response:

--We thank the reviewer for this suggestion.

--As suggested by the reviewer, the manuscript has been carefully checked and the language of the manuscript has been improved by the Wiley editing services. All the errors have been corrected. (See revised manuscript, English editing certificate)

Wiley Editing Services

ENGLISH EDITING CERTIFICATE

This document certifies that the manuscript listed below was edited for proper English language, grammar, punctuation, spelling, and overall style by one or more of the highly qualified native English speaking editors at Wiley Editing Services

Manuscript title

A telomere-to-telomere gapless genome reveals SlPRR1 control of circadian rhythm and photoperiodic flowering in cultivated tomato

Authors

Hui Liu

Order No

VUQZC_1

Date Issued

March 08, 2025

This document certifies that the manuscript listed above was edited for proper English language, grammar, punctuation, spelling, and overall style. Neither the research content nor the authors' intentions were altered in any way during the editing process. Documents receiving this certification should be English-ready for publication; however, the author has the ability to accept or reject our suggestions and changes. If you have any questions or concerns about this document or certification, please contact help-cn@wileyeditingservices.com.

Wiley Publishing Services is a service of Wiley Publishing. Wiley's Scientific, Technical, Medical, and Scholarly (STMS) business serves the world's research and scholarly communities, and is the largest publisher for professional and scholarly societies. Wiley is committed to providing high quality services for researchers. To find out more about Wiley Editing Services, visit http://wileyeditingservices.com. To learn more about our other author services provided by Wiley

WILEY

Reviewer #2:

Major Concerns:

1. A recent triplication event has been identified in tomato genome (2012, Nature). However, no recent WGD event has been detected in the 'VF36' tomato genome.

Response:

--We thank the reviewer for this suggestion.

--As the reviewer mentioned, there was a triplication event in the tomato genome, which occurred in the *Solanum* lineage. In this study, our results indicate that the ‘VF36’ tomato genome does not exhibit species-specific WGD. The ambiguity in our original phrasing led to a misunderstanding. We have revised this sentence. Please see lines 32-35 and 250-252.

Lines 32-35: Through comparative genomics and phylogenetic analysis, we identified structural variations (SVs) between the ‘VF36’ and ‘Heinz 1706’ genomes and found no evidence of a recent species-specific whole-genome duplication (WGD) in the ‘VF36’ tomato.

Lines 250-252: Taken together, these results indicated that the recent species-specific whole-genome duplication (WGD) event did not occur in the ‘VF36’ tomato.

2. Line51, delete 'is' before 'usually regulated'. Line188, remove 'Furthermore,'.

Response:

--We thank the reviewer for this suggestion.

--We have deleted ‘is’ before ‘usually regulated’. We have removed ‘Furthermore’ in Line 184. Please see lines 51-52 and 187-190.

Lines 51-52: Flowering is essential for the transition of plants from vegetative to reproductive growth and usually regulated by day-length (or photoperiod).

Lines 187-190: In addition, BUSCO analysis showed that an average of 98.3% of single-copy genes were completely assembled in the ‘VF36’ genome, which was

slightly higher than that in SLT1.0 (97.7%) and SL5.0 (96.2%).

3. Line118, A gap-free MicroTOM genome has been reported in Plant Biotechnology in 2024. I suggest that authors provide information on MircroTOM in whole genome comparison. This will contribute to a more comprehensive understanding of differences among tomato genomes.

Response:

--We thank the reviewer for this suggestion.

--We have added the comparative genomics analysis between the 'MicroTom' and the 'VF36' genome. Please see lines 180-192.

Lines180-192: In comparison to the 'Heinz 1706' and the 'MicroTom' tomato genome assemblies, the 'VF36' genome assembly displayed a greater length than the 'Heinz 1706' but was shorter than the 'MicroTom' (Table 1). Notably, 12 T2T chromosomes with 12 centromeric regions were predicted in the 'VF36' genome, whereas no telomeres or centromeric regions were identified in the 'Heinz 1706' (SLT1.0 and SL5.0) and 'MicroTom' (SLM_r2.0) genome assemblies. The SLM_r2.0 genome had 16,700 gaps, SLT1.0 genome version had 210 gaps, and even the substantially more complete SL5.0 version still had 31 gaps, whereas no gaps remained in the 'VF36' genome. In addition, BUSCO analysis showed that an average of 98.3% of single-copy genes were completely assembled in the 'VF36' genome, which was slightly higher than that in SLT1.0 (97.7%) and SL5.0 (96.2%). Taken together, the 'VF36' genome assembly demonstrated higher completeness and accuracy than the 'Heinz 1706' and the 'MicroTom' assemblies.

4. Authors detected 34783 genes in the 'VF36' genome, while 36648 genes were found in SL5.0. I wonder why less gene number in the 'VF36' genome that had a more completeness, continuous genome.

Response:

--We thank the reviewer for this suggestion.

--The observed discrepancy in gene numbers between the 'VF36' and SL5.0 genomes may be attributed to the annotation process rather than being solely dependent on

completeness and continuity of the genome assembly. Several factors could underlie this variation: Differences in the gene annotation methods may lead to variations in the number of detected genes. Higher assembly quality can sometimes lead to the merging of fragmented gene models, resulting in a more accurate representation of gene structures but potentially fewer overall gene counts, like overlapping genes or transposable element-related pseudogenes. Natural genomic variation between cultivars (e.g., gene loss, segmental duplications) can contribute to gene count differences.

We have updated the discussion section to describe the discrepancy in gene numbers between the 'VF36' and SL5.0 genomes. Please see lines 342-350.

Lines 342-350: The 'VF36' displayed more completeness and continuity of the genome assembly than 'Heinz 1706', despite the annotation of a greater number of genes in SL5.0 version. This discrepancy might be attributed to differences in gene annotation methods, which could lead to variations in the number of detected genes. Higher assembly quality could sometimes result in the merging of fragmented gene models, thereby yielding a more accurate depiction of gene structures but potentially reducing the overall gene count [40]. Additionally, natural genomic variation between cultivars, such as gene loss or segmental duplications, could also contribute to differences in gene counts [41].

5. Line207, among these SV regions, authors should present the important features, such as functional genes or traits, between the 'VF36' and 'Heinz 1706' genomes. I suggest that authors delete the results of the GO enrichment analysis.

Response:

--We thank the reviewer for this insightful suggestion.

--We agree that highlighting important features such as functional genes or traits within the SV regions is crucial for understanding the differences between the 'VF36' and the 'Heinz 1706' genomes. We acknowledge that while GO enrichment analysis cannot directly provide the key features within the SV regions, it does provide a broad functional overview of the gene involved. We have revised Line 207 and the surrounding text to better reflect these important features of the genomic differences between the 'VF36' and the 'Heinz 1706'. Please see lines 208-212 and Table S13.

Lines 208-212: Among the identified SV regions, a total of 259 genes were functionally annotated. These genes were involved in a variety of biological processes, including metabolism, environmental information processing, and genetic information processing (Table S13). GO enrichment analysis of the genes in the SV regions indicated that the enriched terms were immune response, DNA integration, and metal ion binding (Figure 2D).

Supplementary Table13. The influenced genes by structure variations between VF36 and Heinz1706 genomes.

6. A total of 289116 SNPs were identified between the 'VF36' and 'Heinz 1706' genomes. The Ks value could be estimated to infer the divergence time, providing additional evidence for the 2.1 Mya obtained from gene family analysis.

Response:

--We thank the reviewer for this suggestion.

-- As the reviewer's suggestion, we have estimated the divergence time between the 'VF36' and the 'Heinz 1706' tomato genomes by analyzing the SNPs identified between these two genomic sequences. Please see lines 226-231 and Figure S5.

Lines 226-231: To provide additional evidence, we analyzed the synonymous substitutions per synonymous site (K_S) of collinear homologous genes between the two tomato varieties by analyzing SNP-identified genomic regions. It showed a K_S peak of approximately 0.005 between the genome of the 'VF36' and the 'Heinz 1706' (Figure S5). Using the formula $T=K_S/2r$, we estimated the divergence time between the two tomato varieties to be approximately at 1.55 Mya.

Figure S5 K_S distribution from putative collinear homologous genes between the 'VF36' and the 'Heinz 1706' tomato.

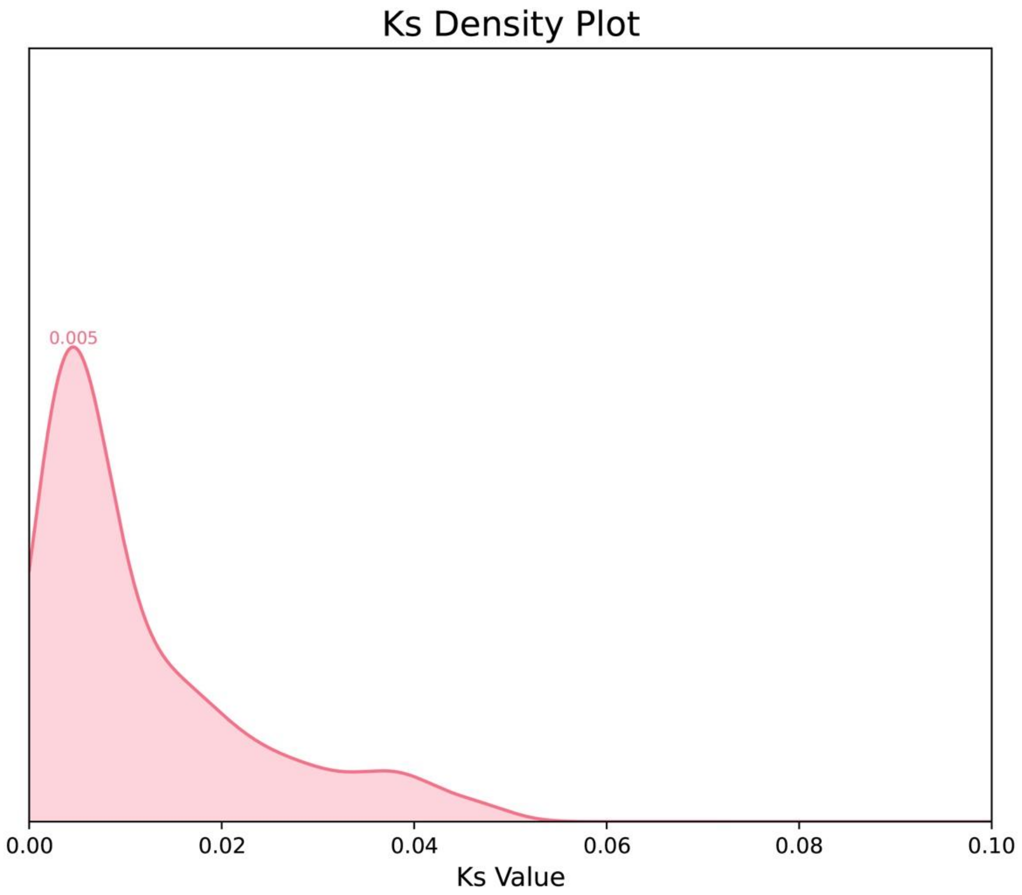

7. Are the sequences of SlPRR1 different between 'VF36' and other genomes? Also, it is worth exploring whether gene expansion, contraction, or specific gene family?

Response:

--We thank the reviewer for this suggestion.

--We have conducted a detailed sequence comparison of the SlPRR1 between the 'VF36' and the 'Heinz 1706'. We observed the two sequences exhibited very few variations. We identified a non-synonymous substitution at position 1074, resulting in an amino acid alteration, and a synonymous substitution at position 1608 located in the C-terminal regulatory region. Please see lines 258-262 and Figure S10.

Lines 258-262: We have conducted a sequence comparison of the SlPRR1 between the 'VF36' and the 'Heinz 1706', it exhibited very few variations. We identified a non-synonymous substitution at position 1074, resulting in an amino acid alteration,

and a synonymous substitution at position 1608 located in the C-terminal regulatory region (Figure S10).

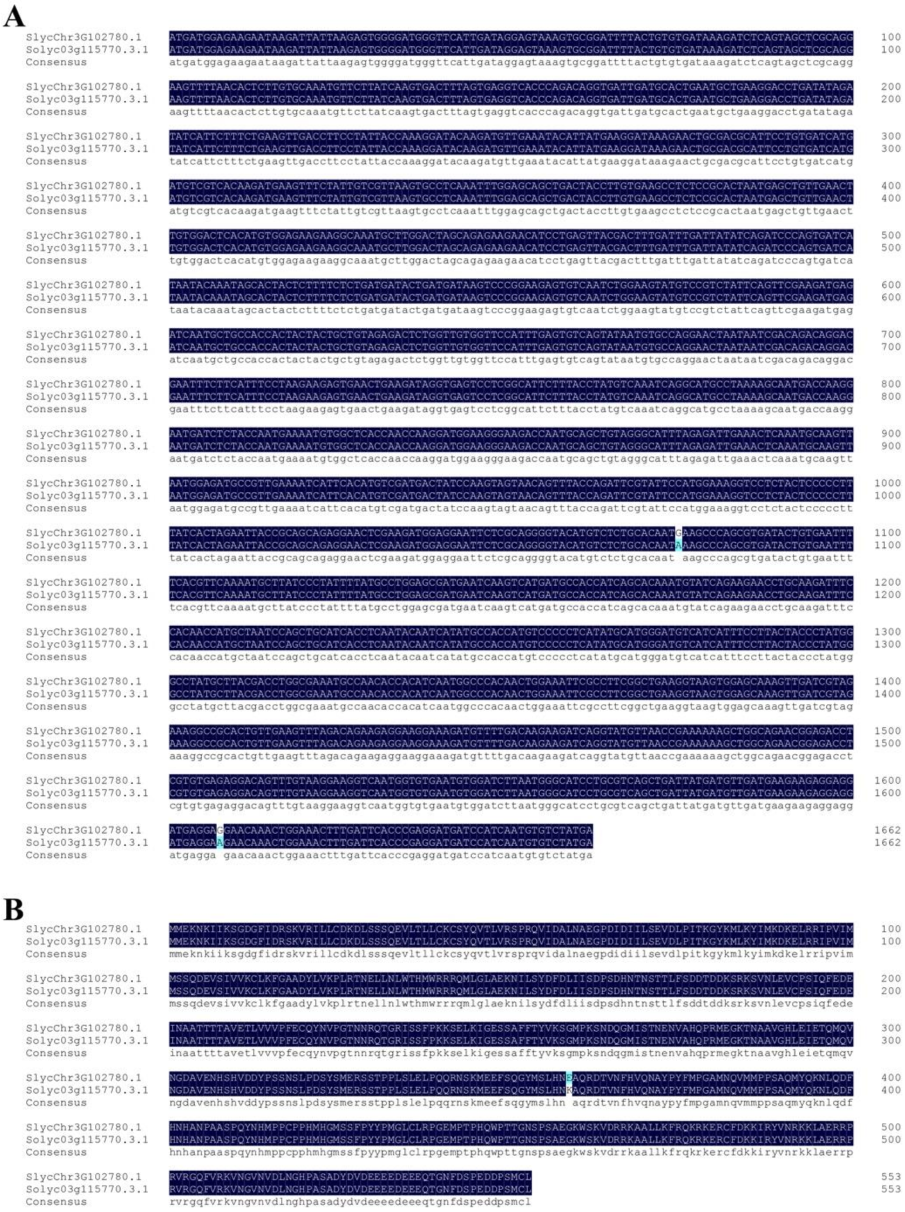

Figure S10 Sequences alignment of SlPRR1 from the ‘VF36’ and the ‘Heinz 1706’ tomatoes.

8. LIN5, SWEET genes are important factors affecting sugar content. Authors may consider providing the expression levels of these genes in the ppr1 mutant lines.

Response:

--We thank the reviewer for this suggestion.

--We have provided the expression analysis of *LIN5*, *SUT1*, and *SWEET* genes in the *slprr1* mutant lines. Please see lines 302-312 and Figure S11.

Lines 302-312: *LIN5*, a tomato *cell-wall-invertase* gene (*CWIN*) was mapped to a major quantitative trait locus (QTL) determining fruit sugar level [39]. Additionally, *SUCROSE TRANSPORTER 1* (*SUT1*) gene, which was responsible for loading and transporting sucrose from source-to-sink organs, encoding an enzyme involved in tomato sucrose metabolism [40]. The expression levels of both *LIN5* and *SUT1* were significantly higher in *slprr1* mutant lines compared to WT lines under LD condition (Figure S11A-S11B). Sugar will eventually be exported transporters (SWEETs) have been verified to mediate sugar transport, with subfamily III members being preferentially explored to transport sucrose in tomato [41]. We surveyed *SWEET*s of subfamily III in tomato, the expression of *SWEET10b*, *11a*, *11c*, and *12a* increased in *slprr1* mutations under LD condition (Figure S11C-S11L).

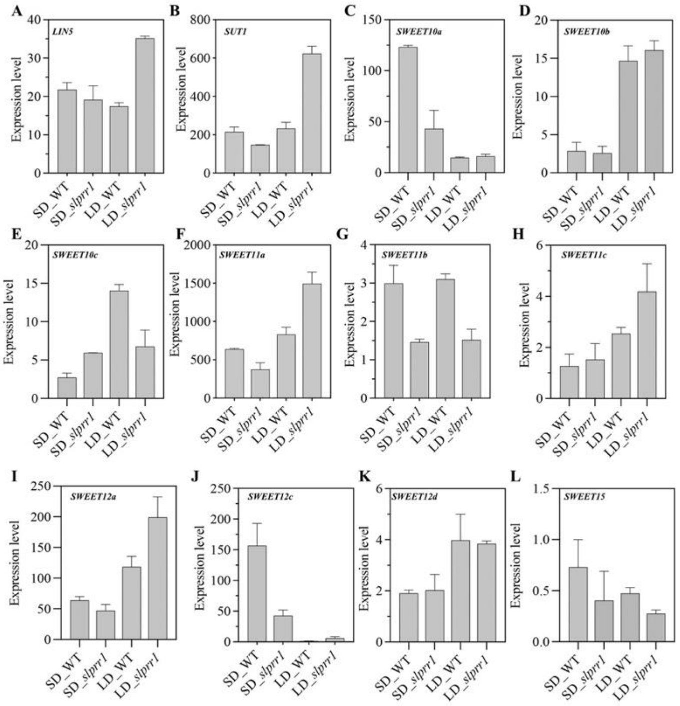

Figure S11 Relative expression levels of *LIN5*, *SUT1*, and *SWEET* genes in tomato.

9. What is the relationship among these genes regulating leaf chlorophyll content and sugar content in the prr1 mutant? Further analysis or experiments are needed to clarify this relationship.

Response:

--We thank the reviewer for this suggestion.

--PRR1, a core member of the circadian oscillator in plant, has been shown to regulate the phase and amplitude of circadian rhythms. Disruption of PRR1 function (*prr1* mutants) results in circadian arrhythmia, which manifests of chlorophyll homeostasis, attenuated biosynthesis, and delayed degradation. Concurrently, this circadian perturbation alters carbon partitioning, leading to aberrant sucrose accumulation in source leaves and reduced phloem loading efficiency, likely through misregulation of sucrose transporters. While the experiments are indeed an important approach to elucidate the regulatory relationship between circadian clock genes and chlorophyll metabolism as well as sugar transport, we will focus on them in our future research. As the reviewer's suggestion, we have added that to Discussion section. Please see lines 389-395.

Lines 389-395: PRR1, a core member of the circadian oscillator in plant, has been shown to regulate the phase and amplitude of circadian rhythms. Disruption of PRR1 function (*prr1* mutants) results in circadian arrhythmia, which manifests of chlorophyll homeostasis, attenuated biosynthesis, and delayed degradation. Concurrently, this circadian perturbation alters carbon partitioning, leading to aberrant sucrose accumulation in source leaves and reduced phloem loading efficiency, likely through mis-regulation of sucrose transporters [51].

Reviewer #3:

I was unable to locate the data or any general information about the project. Perhaps the data are under embargo? I kindly ask the authors to clarify this or to indicate the correct location. On a related note, the paper does not specify when the genome assembly and annotation will be made publicly available. Given GigaScience journal's

policy on data availability, I believe it is important for the authors to address these points.

Response:

--We thank the reviewer for this suggestion.

--We have double checked and revised the 'Availability of data and materials' section. Please see lines 571-574.

Lines 571-574:

Availability of data and materials

Genome Data NCBI BioProject number: PRJNA1204391, BioSample accession: SAMN46040925. All additional supporting data are available in the *GigaScience* repository, GigaDB.
