# Supplementary material for: A telomere-to-telomere gapless genome reveals SlPRR1 control of circadian rhythm and photoperiodic flowering in tomato
Source: Gigascience. 2025 Jul 2;14:giaf058. doi: 10.1093/gigascience/giaf058 (PMC12218202; doi:10.1093/gigascience/giaf058)
Supplement: giaf058_Authors_Response_To_Reviewer_Comments_original_submission [file giaf058_authors_response_to_reviewer_comments_original_submission.pdf]

Author's Response To Reviewer Comments

2 April 2025
GigaScience
Manuscript ID: GIGA-D-24-00568
A telomere-to-telomere gapless genome reveals SlPRR1 control circadian rhythm and photoperiodic flowering in cultivated tomato

Dear Prof. Zhang
Thank you very much for your assistance in handing our manuscript (A telomere-to-telomere gapless genome reveals SlPRR1 control circadian rhythm and photoperiodic flowering in cultivated tomato). Your effort and time spent on our manuscript are greatly appreciated by all of us. We are delighted to all suggestions and review comments, which you and the reviewers made. Your revisions/suggestions have definitely improved the quality of our manuscript.
The manuscript was edited extensively according to reviewer' comments. The language of the manuscript has been improved by a copy-editing company. Please find the revised manuscript in 'GigaScience' manuscript center. The changes were made directly in the text with RED marked. The responses to the reviewers are highlighted below.
Thank you again for your kind help and excellent suggestions for our manuscript. We hope these revisions will be satisfactory and will lead to acceptance for publication. We are looking forward to hearing from you soon.

Yours sincerely
Ai-Sheng Xiong

Dr. Ai-Sheng Xiong
Professor
State Key Laboratory of Crop Genetics & Germplasm Enhancement and Utilization,
College of Horticulture,
Nanjing Agricultural University
1 Weigang, 210095, Nanjing, China
Fax: 86 25 84396790
Email: xiongaisheng@njau.edu.cn

Editor's comments:
We suggest you find a copy-editing company or friendly native English speaker to polish the grammar.
Response:
--We thank the editor and the reviewers for this suggestion.
--As suggested by the editor and both reviewers, the manuscript has been carefully checked and the language of the manuscript has been improved by the Wiley editing services. All the errors have been corrected. (See revised manuscript, English editing certificate)

Reviewers' comments:
Reviewer #1:
Major Concerns:
1. Looks very interesting. I'd be happy to review if once the english language is readable.
Response:
--We thank the reviewer for this suggestion.
--As suggested by the reviewer, the manuscript has been carefully checked and the language of the manuscript has been improved by the Wiley editing services. All the errors have been corrected. (See revised manuscript, English editing certificate)

Reviewer #2:
Major Concerns:
1. A recent triplication event has been identified in tomato genome (2012, Nature). However, no recent WGD event has been detected in the 'VF36' tomato genome.
Response:
--We thank the reviewer for this suggestion.
--As the reviewer mentioned, there was a triplication event in the tomato genome, which occurred in the Solanum lineage. In this study, our results indicate that the ‘VF36’ tomato genome does not exhibit species-specific WGD. The ambiguity in our original phrasing led to a misunderstanding. We have revised this sentence. Please see lines 32-35 and 250-252.

Lines 32-35: Through comparative genomics and phylogenetic analysis, we identified structural variations (SVs) between the ‘VF36’ and ‘Heinz 1706’ genomes and found no evidence of a recent species-specific whole-genome duplication (WGD) in the ‘VF36’ tomato.

Lines 250-252: Taken together, these results indicated that the recent species-specific whole-genome duplication (WGD) event did not occur in the ‘VF36’ tomato.

2. Line51, delete 'is' before 'usually regulated'. Line188, remove 'Furthermore,'.
Response:
--We thank the reviewer for this suggestion.
--We have deleted ‘is’ before ‘usually regulated’. We have removed ‘Furthermore’ in Line 184. Please see lines 51-52 and 187-190.

Lines 51-52: Flowering is essential for the transition of plants from vegetative to reproductive growth and usually regulated by day-length (or photoperiod).

Lines 187-190: In addition, BUSCO analysis showed that an average of 98.3% of single-copy genes were completely assembled in the ‘VF36’ genome, which was slightly higher than that in SLT1.0 (97.7%) and SL5.0 (96.2%).

3. Line118, A gap-free MicroTOM genome has been reported in Plant Biotechnology in 2024. I suggest that authors provide information on MircroTOM in whole genome comparison. This will contribute to a more comprehensive understanding of differences among tomato genomes.
Response:
--We thank the reviewer for this suggestion.
--We have added the comparative genomics analysis between the ‘MicroTom’ and the ‘VF36’ genome. Please see lines 180-192.

Lines180-192: In comparison to the ‘Heinz 1706’ and the ‘MicroTom’ tomato genome assemblies, the ‘VF36’ genome assembly displayed a greater length than the ‘Heinz 1706’ but was shorter than the ‘MicroTom’ (Table 1). Notably, 12 T2T chromosomes with 12 centromeric regions were predicted in the ‘VF36’ genome, whereas no telomeres or centromeric regions were identified in the ‘Heinz 1706’ (SLT1.0 and SL5.0) and ‘MicroTom’ (SLM_r2.0) genome assemblies. The SLM_r2.0 genome had 16,700 gaps, SLT1.0 genome version had 210 gaps, and even the substantially more complete SL5.0 version still had 31 gaps, whereas no gaps remained in the ‘VF36’ genome. In addition, BUSCO analysis showed that an average of 98.3% of single-copy genes were completely assembled in the ‘VF36’ genome, which was slightly higher than that in SLT1.0 (97.7%) and SL5.0 (96.2%). Taken together, the ‘VF36’ genome assembly demonstrated higher completeness and accuracy than the ‘Heinz 1706’ and the ‘MicroTom’ assemblies.

4. Authors detected 34783 genes in the 'VF36' genome, while 36648 genes were found in SL5.0. I wonder why less gene number in the 'VF36' genome that had a more completeness, continuous genome.
Response:
--We thank the reviewer for this suggestion.
--The observed discrepancy in gene numbers between the ‘VF36’ and SL5.0 genomes may be attributed to the annotation process rather than being solely dependent on completeness and continuity of the genome assembly. Several factors could underlie this variation: Differences in the gene annotation methods may lead to variations in the number of detected genes. Higher assembly quality can sometimes lead to the merging of fragmented gene models, resulting in a more accurate representation of gene structures but potentially fewer overall gene counts, like overlapping genes or transposable element-related pseudogenes. Natural genomic variation between cultivars (e.g., gene loss, segmental duplications) can contribute to gene count differences.
We have updated the discussion section to describe the discrepancy in gene numbers between the ‘VF36’ and SL5.0 genomes. Please see lines 342-350.

Lines 342-350: The ‘VF36’ displayed more completeness and continuity of the genome assembly than ‘Heinz 1706’, despite the annotation of a greater number of genes in SL5.0 version. This discrepancy might be attributed to differences in gene annotation methods, which could lead to variations in the number of detected genes. Higher assembly quality could sometimes result in the merging of fragmented gene models, thereby yielding a more accurate depiction of gene structures but potentially reducing the overall gene count [40]. Additionally, natural genomic variation between cultivars, such as gene loss or segmental duplications, could also contribute to differences in gene counts [41].

5. Line207, among these SV regions, authors should present the important features, such as functional genes or traits, between the 'VF36' and 'Heinz 1706' genomes. I suggest that authors delete the results of the GO enrichment analysis.
Response:
--We thank the reviewer for this insightful suggestion.
--We agree that highlighting important features such as functional genes or traits within the SV regions is crucial for understanding the differences between the ‘VF36’ and the ‘Heinz 1706’ genomes. We acknowledge that while GO enrichment analysis cannot directly provide the key features within the SV regions, it does provide a broad functional overview of the gene involved. We have revised Line 207 and the surrounding text to better reflect these important features of the genomic differences between the ‘VF36’ and the ‘Heinz 1706’. Please see lines 208-212 and Table S13.

Lines 208-212: Among the identified SV regions, a total of 259 genes were functionally annotated. These genes were involved in a variety of biological processes, including metabolism, environmental information processing, and genetic information processing (Table S13). GO enrichment analysis of the genes in the SV regions indicated that the enriched terms were immune response, DNA integration, and metal ion binding (Figure 2D).

Supplementary Table13. The influenced genes by structure variations between VF36 and Heinz1706 genomes.

6. A total of 289116 SNPs were identified between the 'VF36' and 'Heinz 1706' genomes. The Ks value could be estimated to infer the divergence time, providing additional evidence for the 2.1 Mya obtained from gene family analysis.
Response:
--We thank the reviewer for this suggestion.
-- As the reviewer’s suggestion, we have estimated the divergence time between the ‘VF36’ and the ‘Heinz 1706’ tomato genomes by analyzing the SNPs identified between these two genomic sequences. Please see lines 226-231 and Figure S5.

Lines 226-231: To provide additional evidence, we analyzed the synonymous substitutions per synonymous site (K_S) of collinear homologous genes between the two tomato varieties by analyzing SNP-identified

genomic regions. It showed a K_S peak of approximately 0.005 between the genome of the ‘VF36’ and the ‘Heinz 1706’ (Figure S5). Using the formula T=K_S/2r, we estimated the divergence time between the two tomato varieties to be approximately at 1.55 Mya.

Figure S5 K_S distribution from putative collinear homologous genes between the ‘VF36’ and the ‘Heinz 1706’ tomato.

7. Are the sequences of SlPRR1 different between 'VF36' and other genomes? Also, it is worth exploring whether gene expansion, contraction, or specific gene family?
Response:
--We thank the reviewer for this suggestion.
--We have conducted a detailed sequence comparison of the SlPRR1 between the ‘VF36’ and the ‘Heinz 1706’. We observed the two sequences exhibited very few variations. We identified a non-synonymous substitution at position 1074, resulting in an amino acid alteration, and a synonymous substitution at position 1608 located in the C-terminal regulatory region. Please see lines 258-262 and Figure S10.

Lines 258-262: We have conducted a sequence comparison of the SlPRR1 between the ‘VF36’ and the ‘Heinz 1706’, it exhibited very few variations. We identified a non-synonymous substitution at position 1074, resulting in an amino acid alteration, and a synonymous substitution at position 1608 located in the C-terminal regulatory region (Figure S10).

Figure S10 Sequences alignment of SlPRR1 from the ‘VF36’ and the ‘Heinz 1706’ tomatoes.

8. LIN5, SWEET genes are important factors affecting sugar content. Authors may consider providing the expression levels of these genes in the ppr1 mutant lines.
Response:
--We thank the reviewer for this suggestion.
--We have provided the expression analysis of LIN5, SUT1, and SWEET genes in the slprr1 mutant lines. Please see lines 302-312 and Figure S11.

Lines 302-312: LIN5, a tomato cell-wall-invertase gene (CWIN) was mapped to a major quantitative trait locus (QTL) determining fruit sugar level [39]. Additionally, SUCROSE TRANSPORTER 1 (SUT1) gene, which was responsible for loading and transporting sucrose from source-to-sink organs, encoding an enzyme involved in tomato sucrose metabolism [40]. The expression levels of both LIN5 and SUT1 were significantly higher in slprr1 mutant lines compared to WT lines under LD condition (Figure S11A-S11B). Sugar will eventually be exported transporters (SWEETs) have been verified to mediate sugar transport, with subfamily III members being preferentially explored to transport sucrose in tomato [41]. We surveyed SWEETs of subfamily III in tomato, the expression of SWEET10b, 11a, 11c, and 12a increased in slprr1 mutations under LD condition (Figure S11C-S11L).

Figure S11 Relative expression levels of LIN5, SUT1, and SWEET genes in tomato.

9. What is the relationship among these genes regulating leaf chlorophyll content and sugar content in the prr1 mutant? Further analysis or experiments are needed to clarify this relationship.
Response:
--We thank the reviewer for this suggestion.
--PRR1, a core member of the circadian oscillator in plant, has been shown to regulate the phase and amplitude of circadian rhythms. Disruption of PRR1 function (prr1 mutants) results in circadian arrhythmia, which manifests of chlorophyll homeostasis, attenuated biosynthesis, and delayed degradation. Concurrently, this circadian perturbation alters carbon partitioning, leading to aberrant sucrose accumulation in source leaves and reduced phloem loading efficiency, likely through misregulation of sucrose transporters. While the experiments are indeed an important approach to elucidate the regulatory relationship between circadian clock genes and chlorophyll metabolism as well as sugar transport, we will

focus on them in our future research. As the reviewer's suggestion, we have added that to Discussion section. Please see lines 389-395.

Lines 389-395: PRR1, a core member of the circadian oscillator in plant, has been shown to regulate the phase and amplitude of circadian rhythms. Disruption of PRR1 function (prr1 mutants) results in circadian arrhythmia, which manifests of chlorophyll homeostasis, attenuated biosynthesis, and delayed degradation. Concurrently, this circadian perturbation alters carbon partitioning, leading to aberrant sucrose accumulation in source leaves and reduced phloem loading efficiency, likely through mis-regulation of sucrose transporters [51].

Reviewer #3:
I was unable to locate the data or any general information about the project. Perhaps the data are under embargo? I kindly ask the authors to clarify this or to indicate the correct location. On a related note, the paper does not specify when the genome assembly and annotation will be made publicly available. Given GigaScience journal's policy on data availability, I believe it is important for the authors to address these points.
Response:
--We thank the reviewer for this suggestion.
--We have double checked and revised the 'Availability of data and materials' section. Please see lines 571-574.

Lines 571-574:
Availability of data and materials
Genome Data NCBI BioProject number: PRJNA1204391, BioSample accession: SAMN46040925. All additional supporting data are available in the GigaScience repository, GigaDB.

em Editorial Manager®

Cookie Preference Center

We use cookies which are necessary to make our site work. We may also use additional cookies to analyze, improve and personalize our content and your digital experience. For more information, see our Cookie Policy.

You may choose not to allow some types of cookies. However, blocking some types may impact your experience of our site and the services we are able to offer. See the different category headings below to find out more or change your settings.

Allow all
Manage Consent Preferences

Strictly Necessary Cookies

Always active

These cookies are necessary for the website to function and cannot be switched off in our systems. They are usually only set in response to actions made by you which amount to a request for services, such as setting your privacy preferences, logging in or filling in forms. You can set your browser to block or alert you about these cookies, but some parts of the site will not then work. These cookies do not store any personally identifiable information.

Functional Cookies

These cookies enable the website to provide enhanced functionality and personalisation. They may be set by us or by third party providers whose services we have added to our pages. If you do not allow these cookies then some or all of these services may not function properly.

Performance Cookies

These cookies allow us to count visits and traffic sources so we can measure and improve the performance of our site. They help us to know which pages are the most and least popular and see how visitors move around the site.

Targeting Cookies

These cookies may be set through our site by our advertising partners. They may be used by those companies to build a profile of your interests and show you relevant adverts on other sites. If you do not allow these cookies, you will experience less targeted advertising.

Cookie List

Clear

Apply Cancel
Consent Leg.Interest

Confirm my choices

Powered by onetrust
