# Supplementary material for: A telomere-to-telomere gapless genome reveals SlPRR1 control of circadian rhythm and photoperiodic flowering in tomato
Source: Gigascience. 2025 Jul 2;14:giaf058. doi: 10.1093/gigascience/giaf058 (PMC12218202; doi:10.1093/gigascience/giaf058)
Supplement: giaf058_Reviewer_2_Report_Original_Submission — Tao Lin -- 2/8/2025 [file giaf058_reviewer_2_report_original_submission.pdf]

Reviewer Report

Title: A telomere-to-telomere gapless genome reveals SlPRR1 control of circadian rhythm and photoperiodic flowering in tomato

Version: Original Submission **Date:** 2/8/2025

Reviewer name: Tao Lin

Reviewer Comments to Author:

Gapless genome is crucial to explore functional mechanism, evolutionary, etc. A gap free reference genome is absence for tomato. Liu et al. constructed a gapless genome and explored a core circadian oscillator SlPRR1 using CRISP/Cas9 technology. Some flaws need to be revised.
A recent triplication event has been identified in tomato genome (2012, Nature). However, no recent WGD event has been detected in the 'VF36' tomato genome.
Line51, delete 'is' before 'usually regulated'. Line188, remove 'Furthermore,'.
Line118, A gap-free MicroTOM genome has been reported in Plant Biotechnology in 2024. I suggest that authors provide information on MircroTOM in whole genome comparison. This will contribute to a more comprehensive understanding of differences among tomato genomes.
Authors detected 34783 genes in the 'VF36' genome, while 36648 genes were found in SL5.0. I wonder why less gene number in the 'VF36' genome that had a more completeness, continuous genome.
Line207, Among these SV regions, authors should present the important features, such as functional genes or traits, between the 'VF36' and 'Heinz 1706' genomes. I suggest that authors delete the results of the GO enrichment analysis.
A total of 289116 SNPs were identified between the 'VF36' and 'Heinz 1706' genomes. The Ks value could be estimated to infer the divergence time, providing additional evidence for the 2.1 Mya obtained from gene family analysis.
Are the sequences of SlPRR1 different between 'VF36' and other genomes? Also, it is worth exploring whether gene expansion, contraction, or specific gene family?
LIN5, SWEET genes are important factors affecting sugar content. Authors may consider providing the expression levels of these genes in the ppr1 mutant lines.
What is the relationship among these genes regulating leaf chlorophyll content and sugar content in the prr1 mutant? Further analysis or experiments are needed to clarify this relationship.

Methods

Are the methods appropriate to the aims of the study, are they well described, and are necessary controls included? Choose an item.

Conclusions

Are the conclusions adequately supported by the data shown? Choose an item.

Reporting Standards

Does the manuscript adhere to the journal's guidelines on [minimum standards of reporting?]() Choose an item.

Choose an item.

Statistics

Are you able to assess all statistics in the manuscript, including the appropriateness of statistical tests used? Choose an item.

Quality of Written English

Please indicate the quality of language in the manuscript: Choose an item.

Declaration of Competing Interests

Please complete a declaration of competing interests, considering the following questions:

- Have you in the past five years received reimbursements, fees, funding, or salary from an organisation that may in any way gain or lose financially from the publication of this manuscript, either now or in the future?
- Do you hold any stocks or shares in an organisation that may in any way gain or lose financially from the publication of this manuscript, either now or in the future?
- Do you hold or are you currently applying for any patents relating to the content of the manuscript?
- Have you received reimbursements, fees, funding, or salary from an organization that holds or has applied for patents relating to the content of the manuscript?
- Do you have any other financial competing interests?
- Do you have any non-financial competing interests in relation to this paper?

If you can answer no to all of the above, write 'I declare that I have no competing interests' below. If your reply is yes to any, please give details below.

I declare that I have no competing interests.

I agree to the open peer review policy of the journal. I understand that my name will be included on my report to the authors and, if the manuscript is accepted for publication, my named report including any attachments I upload will be posted on the website along with the authors' responses. I agree for my report to be made available under an Open Access Creative Commons CC-BY license (http://creativecommons.org/licenses/by/4.0/). I understand that any comments which I do not wish to be included in my named report can be included as confidential comments to the editors, which will not be published.

Choose an item.

To further support our reviewers, we have joined with Publons, where you can gain additional credit to further highlight your hard work (see: https://publons.com/journal/530/gigascience). On publication of this paper, your review will be automatically added to Publons, you can then choose whether or not to claim your Publons credit. I understand this statement.

Yes Choose an item.
