# Supplementary material for: A telomere-to-telomere gapless genome reveals SlPRR1 control of circadian rhythm and photoperiodic flowering in tomato
Source: Gigascience. 2025 Jul 2;14:giaf058. doi: 10.1093/gigascience/giaf058 (PMC12218202; doi:10.1093/gigascience/giaf058)
Supplement: giaf058_Reviewer_3_Report_Original_Submission — Alessandro Cestaro -- 2/26/2025 [file giaf058_reviewer_3_report_original_submission.pdf]

Reviewer Report

Title: A telomere-to-telomere gapless genome reveals SlPRR1 control of circadian rhythm and photoperiodic flowering in tomato

Version: Original Submission **Date:** 2/26/2025

Reviewer name: Alessandro Cestaro

Reviewer Comments to Author:

Hui Liu and colleagues present in this paper the genome of the tomato cultivar VF36, focusing on its flowering response to photoperiod. In my opinion, they have done an excellent job. The experimental design is solid, the materials and methods are more than adequate, and all software used is state-of-the-art and properly specified. The quality of the assembly is high, and their analysis of flowering time is convincing. Considering all these aspects, I believe the paper is worthy of publication in its present form.
So, why the minor revision? The authors state that the raw data are available at the CNGB Nucleotide Sequence Archive (https://db.cngb.org/cnsa) under the accession ID CNP0006298. However, I was unable to locate the data or any general information about the project. Perhaps the data are under embargo? I kindly ask the authors to clarify this or to indicate the correct location.
On a related note, the paper does not specify when the genome assembly and annotation will be made publicly available. Given GigaScience journal's policy on data availability, I believe it is important for the authors to address these points.

Methods

Are the methods appropriate to the aims of the study, are they well described, and are necessary controls included? Choose an item.

Conclusions

Are the conclusions adequately supported by the data shown? Choose an item.

Reporting Standards

Does the manuscript adhere to the journal's guidelines on [minimum standards of reporting?](#) Choose an item.

Choose an item.

Statistics

Are you able to assess all statistics in the manuscript, including the appropriateness of statistical tests used? Choose an item.

Quality of Written English

Please indicate the quality of language in the manuscript: Choose an item.

Declaration of Competing Interests

Please complete a declaration of competing interests, considering the following questions:

- Have you in the past five years received reimbursements, fees, funding, or salary from an organisation that may in any way gain or lose financially from the publication of this manuscript, either now or in the future?
- Do you hold any stocks or shares in an organisation that may in any way gain or lose financially from the publication of this manuscript, either now or in the future?
- Do you hold or are you currently applying for any patents relating to the content of the manuscript?
- Have you received reimbursements, fees, funding, or salary from an organization that holds or has applied for patents relating to the content of the manuscript?
- Do you have any other financial competing interests?
- Do you have any non-financial competing interests in relation to this paper?

If you can answer no to all of the above, write 'I declare that I have no competing interests' below. If your reply is yes to any, please give details below.

I have no competing interests

I agree to the open peer review policy of the journal. I understand that my name will be included on my report to the authors and, if the manuscript is accepted for publication, my named report including any attachments I upload will be posted on the website along with the authors' responses. I agree for my report to be made available under an Open Access Creative Commons CC-BY license (http://creativecommons.org/licenses/by/4.0/). I understand that any comments which I do not wish to be included in my named report can be included as confidential comments to the editors, which will not be published.

Choose an item.

To further support our reviewers, we have joined with Publons, where you can gain additional credit to further highlight your hard work (see: https://publons.com/journal/530/gigascience). On publication of this paper, your review will be automatically added to Publons, you can then choose whether or not to claim your Publons credit. I understand this statement.

Yes Choose an item.
